# Nickel-Catalyzed Enantioselective Electrochemical Reductive Cross-Coupling of Aryl Aziridines with Alkenyl Bromides

**DOI:** 10.1021/jacs.2c12869

**Published:** 2023-03-07

**Authors:** Xia Hu, Iván Cheng-Sánchez, Sergio Cuesta-Galisteo, Cristina Nevado

**Affiliations:** Department of Chemistry, University of Zurich, Winterthurerstrasse 190, CH 8057 Zurich, Switzerland

## Abstract

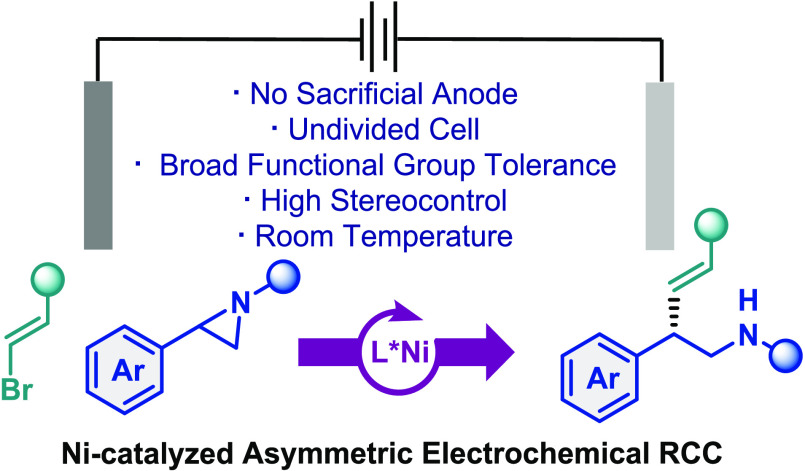

An electrochemically driven nickel-catalyzed enantioselective reductive cross-coupling of aryl aziridines with alkenyl bromides has been developed, affording enantioenriched β-aryl homoallylic amines with excellent *E*-selectivity. This electroreductive strategy proceeds in the absence of heterogeneous metal reductants and sacrificial anodes by employing constant current electrolysis in an undivided cell with triethylamine as a terminal reductant. The reaction features mild conditions, remarkable stereocontrol, broad substrate scope, and excellent functional group compatibility, which was illustrated by the late-stage functionalization of bioactive molecules. Mechanistic studies indicate that this transformation conforms with a stereoconvergent mechanism in which the aziridine is activated through a nucleophilic halide ring-opening process.

## Introduction

**Scheme 1:**
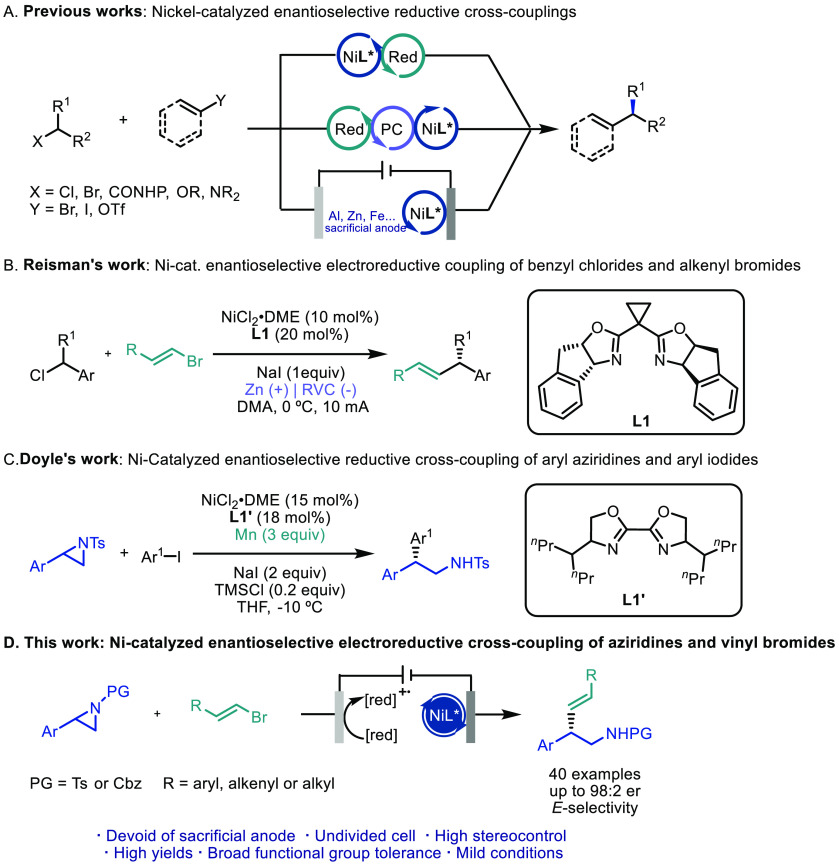
Strategies for Nickel-Catalyzed Enantioselective Cross-Electrophile Couplings

Nickel-catalyzed enantioselective cross-electrophile couplings represent a powerful strategy for the construction of stereogenic carbon centers.^1^ Compared to traditional asymmetric cross-coupling reactions,^2^ the direct coupling of two electrophiles precludes the preparation of sensitive organometallic species, thus enhancing both the operability and functional group compatibility of the overall process. A super stoichiometric amount of metal reductants such as manganese or zinc is typically required to turn over the nickel catalyst,^3,4^ which not only can lead to unpredictable results depending on stirring methods but also generates additional waste. Significant efforts have been undertaken to circumvent these challenges, including the use of organic reductants such as tetrakis(dimethylamino)ethylene (TDAE) or bis(pinacolato)diboron (B_2_Pin_2_) among several others (Scheme 1A, top).^5^ Further, with the advent of photoredox/nickel dual catalysis,^6^ organic reducing reagents, including amines and Hantzsch esters (HEH), have also been successfully employed in asymmetric metallaphotoredox cross-electrophile couplings (Scheme 1A, middle).^7^

In parallel to these developments, the past years have witnessed the renaissance of electrochemistry as a sustainable tool to replace chemical oxidants and reductants.^8^ The combination of cathodic reduction and nickel catalysis has proven to be an effective strategy for cross-couplings.^9^ Still, considerable limitations need to be addressed for these methodologies to attain their full potential. First and foremost, the use of metal sacrificial anodes (*e.g.*, aluminum, zinc, iron, *etc.*) complicates the scalability of the processes. Second, the control of stereochemistry still represents a significant challenge^10^ and, despite a few examples,^11^ most nickel-catalyzed electrochemically mediated processes deliver the corresponding products in racemic form (Scheme 1A, bottom). Notably, Reisman’s group has reported a Ni-catalyzed enantioselective cross-coupling of benzylic chlorides and alkenyl bromides using zinc as a sacrificial anode (Scheme 1B).^11c^

Intrigued by these limitations, we set out to develop a nickel-catalyzed asymmetric reductive cross-coupling devoid of sacrificial anodes that would explore electrophiles beyond the well-studied C(sp^2^)–X and C(sp^3^)–X systems. Aziridines are versatile building blocks^12^ that have been successfully incorporated in Ni-catalyzed enantioselective cross-coupling processes.^13^ An elegant study from Doyle and co-workers reported the enantioselective reductive cross-coupling between aryl aziridines and aryl iodides by employing manganese as a stoichiometric reductant (Scheme 1C).^13c^ Inspired by these precedents, we present the first example of a nickel-catalyzed asymmetric cross-electrophile coupling between aryl aziridines and alkenyl bromides, merging a constant current electrolysis process in a single cell with triethyl amine as a sustainable electron donor (Scheme 1D). Both the regio- and enantioselectivity of the reaction are controlled by a chiral bis(oxazoline) ligand. The obtained enantioenriched β-aryl homoallylic amines are not only important structural motifs found in pharmacologically and biologically active molecules^14^ but also useful synthetic intermediates to access a variety of valuable N-containing secondary metabolites.

## Results and Discussion

We began our investigations into this nickel-catalyzed asymmetric electroreductive cross-coupling with racemic 2-phenyl-1-tosylaziridine and β-bromostyrene as model reactants.^15^ After systematic evaluation of the reaction parameters (see the Tables S-1–S-9, Supporting Information), we were delighted to find that, in the presence of 10 mol % NiBr_2_·DME, 12 mol % chiral bis(oxazoline) **L1**, 25 mol % MgCl_2_, 5.0 equiv of Et_3_N, and 1.0 equiv of ^*n*^Bu_4_NBF_4_ in dimethylacetamide (DMA), the desired product (*S*,*E*)-*N*-(2,4-diphenylbut-3-en-1-yl)-4-methylbenzenesulfonamide **1** could be obtained in 70% isolated yield. Gratifyingly, the reaction proceeded with excellent stereocontrol (96:4 er) by using a graphite anode and a nickel foam cathode in an undivided cell under 10 mA constant current electrolysis (Table 1, entry 1). The reaction showed excellent stereoselectivity, since only *E*-product **1** was obtained even when *Z*- or *E*/*Z*-mixed β-bromostyrenes were used as starting materials (see Table S-11 in the Supporting Information).^3b,15,16^ A screening of chiral indanyl-substituted bis(oxazoline) ligands with different central linkers revealed the cyclopropyl-substituted one (**L1**) as the best compromise between reactivity and enantioselectivity (**L2**–**L4**). In contrast, pyridine-oxazoline ligand **L5** led to a low enantiomeric ratio, whereas chiral bioxazoline and bisimidazoline ligands (**L6**–**L9**) delivered the product in lower yields with moderate enantioselectivity. A slightly decreased yield was observed when the graphite anode was replaced with RVC foam or carbon felt (Table 1, entries 2 and 3). The choice of cathode material was essential: nickel or platinum plate cathodes resulted in near-complete failure of the reaction (Table 1, entry 4). This result could be attributed to the electrode surface area effect, as the large surface area of the Ni foam electrode might enhance the rate of surface reaction, thus increasing the overall efficiency of the system.^17^ Different nickel catalysts such as NiCl_2_·DME and NiBr_2_·diglyme afforded the product with lower yields but comparable er (Table 1, entries 5 and 6). Other electrolytes such as ^*n*^Bu_4_NPF_6_ or NaBF_4_ also provided good reactivity (Table 1, entries 7 and 8), while LiBF_4_ delivered **1** in lower yield (Table 1, entry 9). The reaction proceeded smoothly when the current was adjusted to 5 or 15 mA, albeit with lower yields (Table 1, entries 10 and 11). The yield decreased to 55% in the absence of MgCl_2_ (Table 1, entry 12), and MgBr_2_ had a weaker promoting effect compared with MgCl_2_ (Table 1, entry 13). Reducing the number of equivalents of Et_3_N or alkenyl bromide decreased the reaction efficiency (Table 1, entries 14 and 15). To our delight, a slight increase in yield was achieved when the reaction was performed on a 0.2 mmol scale (Table 1, entry 16). Control experiments indicated that the nickel source, the ligand, and the electrical current were all necessary for this transformation (Table 1, entry 17). It is worth noting that the use of stoichiometric amounts of Mn or Zn powder significantly reduced the yield and enantioselectivity of the process, likely as a result of unproductive pathways involving organometallic intermediates generated in the reaction media under these conditions (Table 1, entries 18 and 19).

**Table 1:**
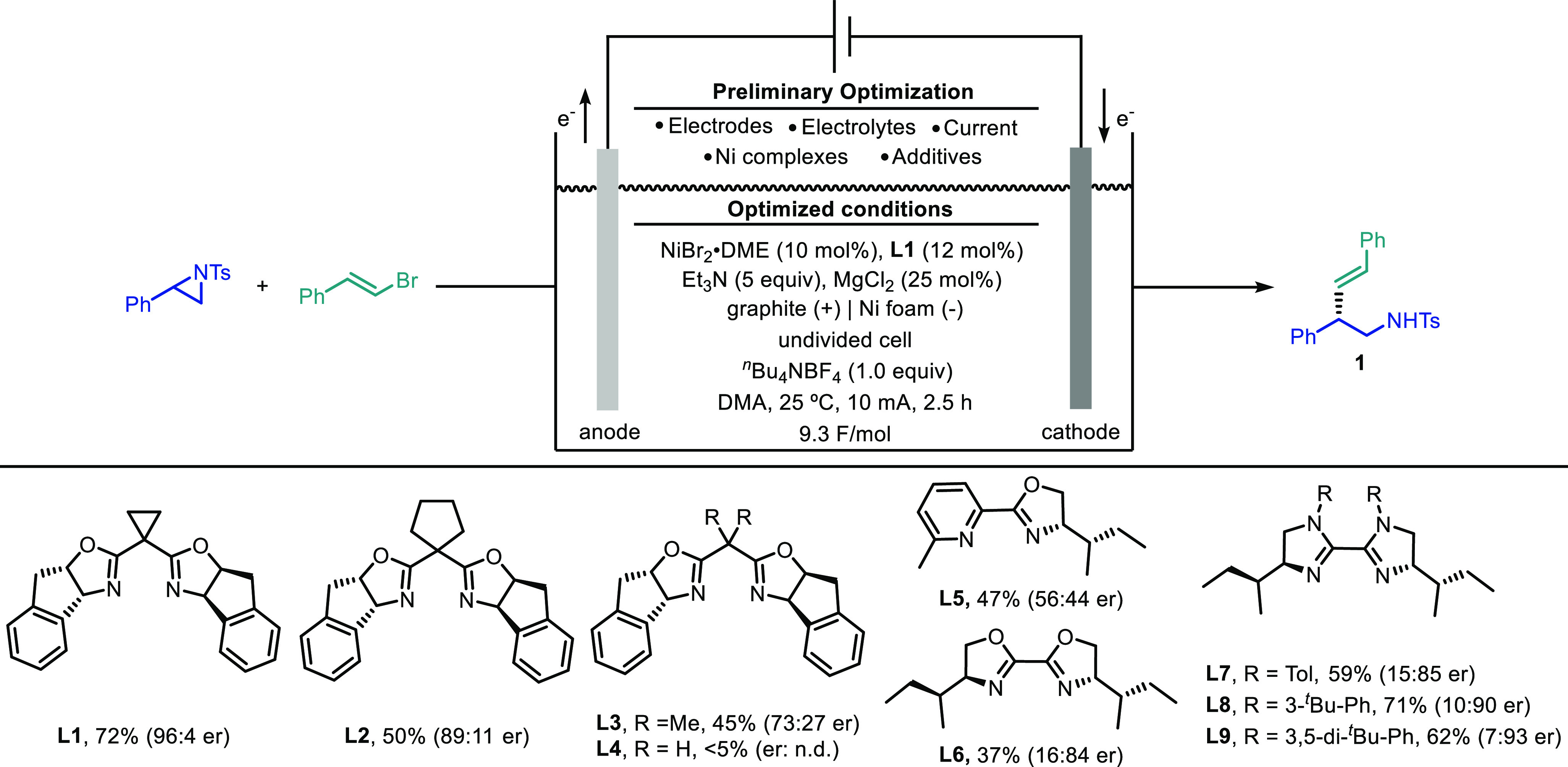
Optimization of the Reaction Conditions^a^

entry	deviation from standard conditions	**1** yield (%)^b^	er^c^
1	none	72 (70)	96:4
2	RVC (+) instead of graphite (+)	63	96:4
3	carbon felt (+) instead of graphite (+)	65	96:4
4	Ni (−) or Pt (−) instead of Ni foam (−)	<5	n.d.^f^
5	NiCl_2_·DME instead of NiBr_2_·DME	53	95:5
6	NiBr_2_·diglyme instead of NiBr_2_·DME	50	96:4
7	^*n*^Bu_4_NPF_6_ instead of ^*n*^Bu_4_NBF_4_	66	96:4
8	NaBF_4_ instead of ^*n*^Bu_4_NBF_4_	70	95:5
9	LiBF_4_ instead of ^*n*^Bu_4_NBF_4_	46	95:5
10	5 mA, 5 h instead of 10 mA, 2.5 h	55	96:4
11	15 mA, 1.67 h instead of 10 mA, 2.5 h	60	96:4
12	Without MgCl_2_	55	96:4
13	MgBr_2_ instead of MgCl_2_	61	96:4
14	3 equiv Et_3_N instead of 5 equiv Et_3_N	53	96:4
15	2 equiv β-bromostyrene was used	59	96:4
16^d^	0.2 mmol reaction scale	75 (73)	96:4
17	w/o electric current, Ni or **L1**	0	n.d.
18^e^	3 equiv Mn instead of current and Et_3_N	20	76:24
19^e^	3 equiv Zn instead of current and Et_3_N	25	80:20

a Standard reaction conditions: graphite anode, nickel foam cathode, 2-phenyl-1-tosylaziridine (0.1 mmol, 1.0 equiv), β-bromostyrene (0.3 mmol, 3.0 equiv), ^*n*^Bu_4_NBF_4_ (0.1 mmol, 1.0 equiv), Et_3_N (0.5 mmol, 5.0 equiv), MgCl_2_ (0.025 mmol, 25 mol %), NiBr_2_·DME (0.01 mmol, 10 mol %), **L1** (0.012 mmol, 12 mol %), DMA (3.0 mL), constant current = 10 mA, undivided cell, N_2_, 2.5 h, 25 °C.

b Yields were determined by ^1^H NMR using 1,3,5-trimethoxybenzene as the internal standard; isolated yields after column chromatography are shown in brackets.

c The enantiomeric ratios (er) were determined by chiral high-performance liquid chromatography (HPLC).

d Reaction time = 5 h.

e Reaction time = 24 h.

f n.d. = not determined.

**Scheme 2:**
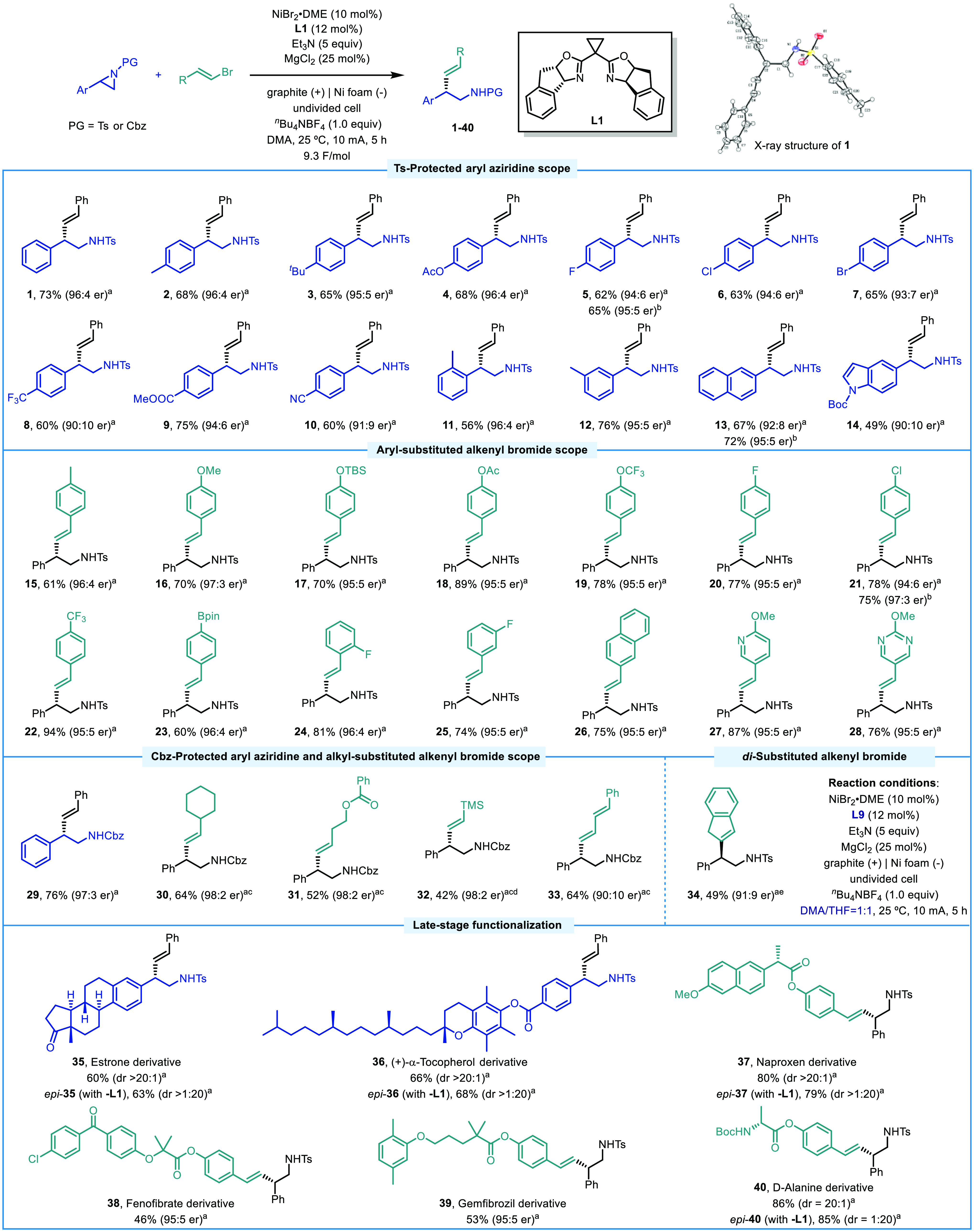
Substrate Scope Reaction conditions: See Table 1, entry 16. Isolated yields after column chromatography. Enantiomeric ratios (er) were determined by chiral HPLC. DIPEA instead of Et_3_N. 20 mol % NiBr_2_·DME and 24 mol % **L1**. Alkenyl bromide (5 equiv). **L9** was used as the ligand, and a 1:1 mixture of DMA/tetrahydrofuran (THF) was used as the solvent.

With the optimized conditions in hand, we sought to examine the generality of this transformation (Scheme 2). The absolute stereochemistry of compound **1** was unambiguously confirmed by X-ray diffraction analysis, and the configuration of all other products was assigned by analogy.^15^ A wide range of 2-aryl-substituted *N*-tosyl-protected aziridines bearing electron-donating groups (−Me, −^*t*^Bu, −OAc), halogens (−F, −Cl, −Br), and electron-withdrawing groups (−CF_3_, −COOMe, −CN) at the *para*-position of the phenyl ring readily underwent the cross-coupling with β-bromostyrene to form β-aryl *E*-configured homoallylic sulfonamides in moderate to good yields with high enantioselectivities (**2**–**10**). 2-(*o*-Tolyl)- and 2-(*m*-tolyl)-*N*-tosylaziridines were also well tolerated, demonstrating that increased steric hindrance has little effect on the reaction efficiency and enantioselectivity (**11** and **12**). Further, 2-naphthyl- and 5-indolyl-substituted aziridines also proved to be competent coupling partners (**13** and **14**). In some cases, the use of *N*,*N-*diisopropylethylamine (DIPEA) as the reductant improved both the yields and enantioselectivities as in the case of products **5** and **13** (see Table S12 in the Supporting Information for additional information).

Different alkenyl bromide partners were explored next. Styrenyl bromides bearing a variety of functional groups, such as methyl (**15**), methoxy (**16**), (*tert*-butyldimethylsilyl)oxy (**17**), acetoxy (**18**), trifluoromethoxy (**19**), fluoro (**20**), chloro (**21**), trifluoromethyl (**22**), and pinacol boronate (**23**), turned out to be compatible with the established protocol delivering the corresponding products in good yields and high enantiomeric ratios (60–94% yield, 95:5–97:3 er). *ortho*- and *meta*-Fluorophenyl-substituted alkenyl bromides could also participate in the reaction efficiently (**24** and **25**). Notably, the tolerance to halogen and pinacol boronate functional groups opens the possibility of subsequent derivatization of the β-aryl homoallylic amines. Alkenyl bromides bearing naphthalene (**26**), pyridine (**27**), and pyrimidine (**28**) rings were also successfully converted to the desired products with high enantioselectivity, thus highlighting the potential of this strategy in the synthesis of medicinal chemistry-relevant compounds.

We were pleased to find that benzyloxycarbonyl (Cbz)-protected aziridines also reacted smoothly with β-bromostyrene under the standard reaction conditions, furnishing product **29** in 76% yield and 97:3 er. This result further emphasizes the advantage of this electrochemical reduction protocol, as this type of compound was inaccessible with previous Ni-catalyzed aziridine asymmetric cross-electrophile couplings.^13c^ In addition to styrenyl bromides, β-alkyl substituted vinyl bromides (**30** and **31**), bromovinyl silane (**32**), and conjugated dienyl bromide (**33**) turned out to be suitable reaction partners delivering the corresponding Cbz-protected products with high to excellent enantioselectivities (up to 98:2 er). Remarkably, 2-bromo-1*H*-indene, a cyclic di-substituted alkenyl bromide, which is typically a challenging substrate in asymmetric alkenylations,^3b,3e,11c^ was a viable partner delivering **34** under modified reaction conditions using **L9** as the ligand in a DMA/THF binary solvent system.

The synthetic potential of this asymmetric electroreductive cross-coupling was further demonstrated through the late-stage functionalization of structurally diverse natural products and pharmaceutical agents. Specifically, aziridines derived from estrone (**35**) and (+)-α-tocopherol (**36**) could be readily incorporated into this protocol with excellent diastereocontrol. In addition, alkenyl bromides resembling derivatives of naproxen, fenofibrate, gemfibrozil, and d-alanine could all furnish chiral homoallylic amines **37**–**40** (*epi***-35**, *epi***-36**, *epi***-37**, *epi***-40** were obtained by using *ent*-ligand**-L1**) in moderate to good yields with high levels of diastereocontrol.

**Scheme 3:**
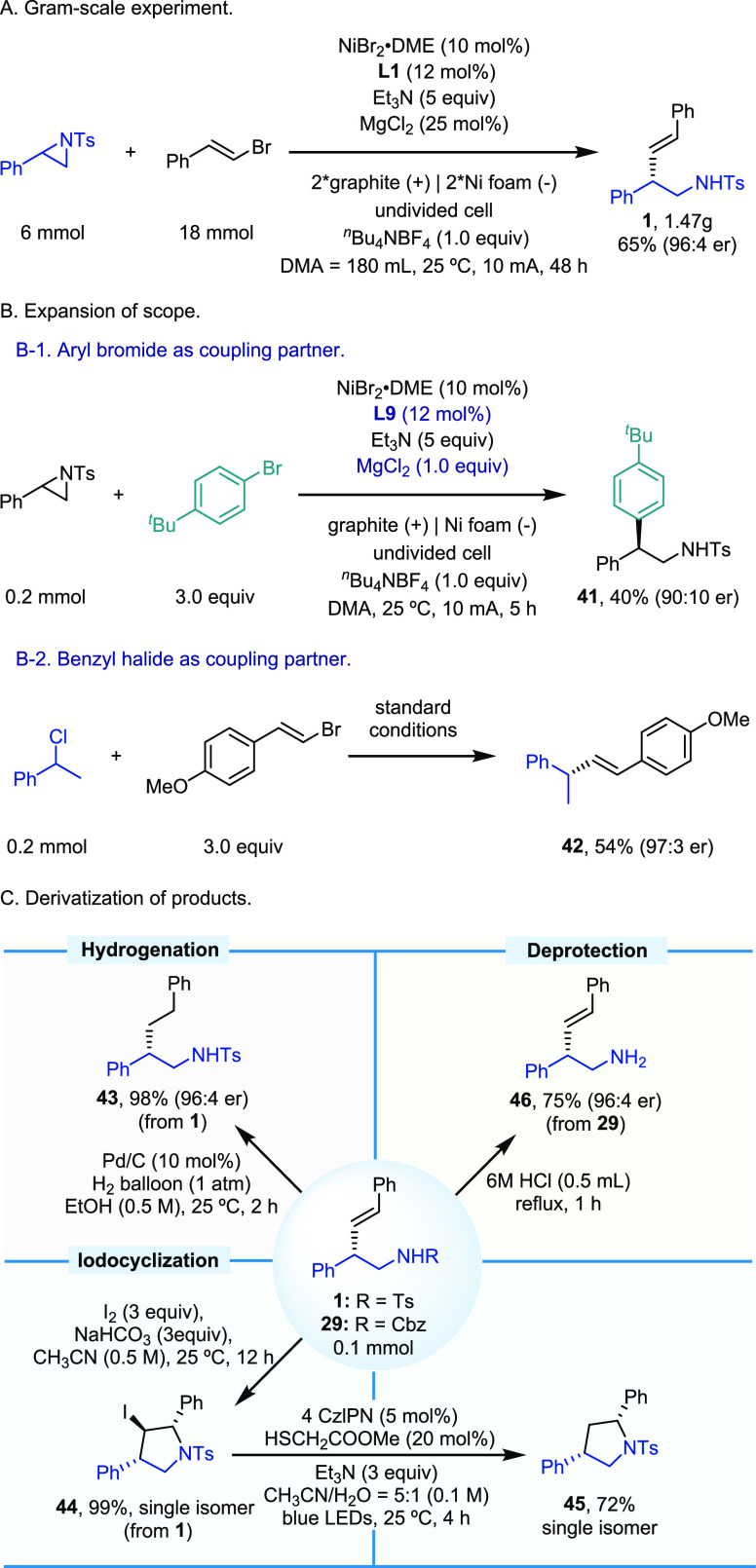
Synthetic Applications

The practicality of this methodology could be demonstrated in multigram-scale experiments (Scheme 3A). The constant current electrolysis of 6 mmol of 2-phenyl-1-tosylaziridine with β-bromostyrene produced the desired product **1** in 65% isolated yield and 96:4 er. Moreover, our protocol can be extended to other coupling partners (Scheme 3B). The reductive cross-coupling of 2-phenyl-1-tosylaziridine with 1-bromo-4-(*tert*-butyl)benzene was achieved under modified reaction conditions (**L9** as the ligand and 1 equiv of MgCl_2_ as the additive) furnishing β-aryl sulfonamide **41** in 40% yield and 90:10 er. Further, the coupling of (1-chloroethyl)benzene and (*E*)-1-(2-bromovinyl)-4-methoxybenzene under the standard reaction conditions delivered the desired product **42** in 54% yield and 97:3 er. Derivatization of the chiral homoallylic amine products could also be successfully accomplished (Scheme 3C). The palladium-catalyzed hydrogenation of **1** delivered the corresponding chiral β-branched alkylamine **43** with excellent stereofidelity. In the presence of I_2_ and NaHCO_3_, enantioenriched iodosubstituted pyrrolidine (**44**), containing three contiguous chiral centers, could be obtained in near-quantitative yield by diastereoselective iodocyclization of **1**. A photoredox-catalyzed dehalogenation of **44** furnished chiral 2,4-disubstituted pyrrolidine (**45**) by using Et_3_N as the halogen-atom transfer agent and methyl thioglycolate–H_2_O as the hydrogen atom donor. Finally, deprotection of the *N*-benzyloxycarbonyl group in **29** was successfully accomplished by treatment with 6 M HCl under reflux to deliver chiral homoallylic primary amine (**46**).

**Scheme 4:**
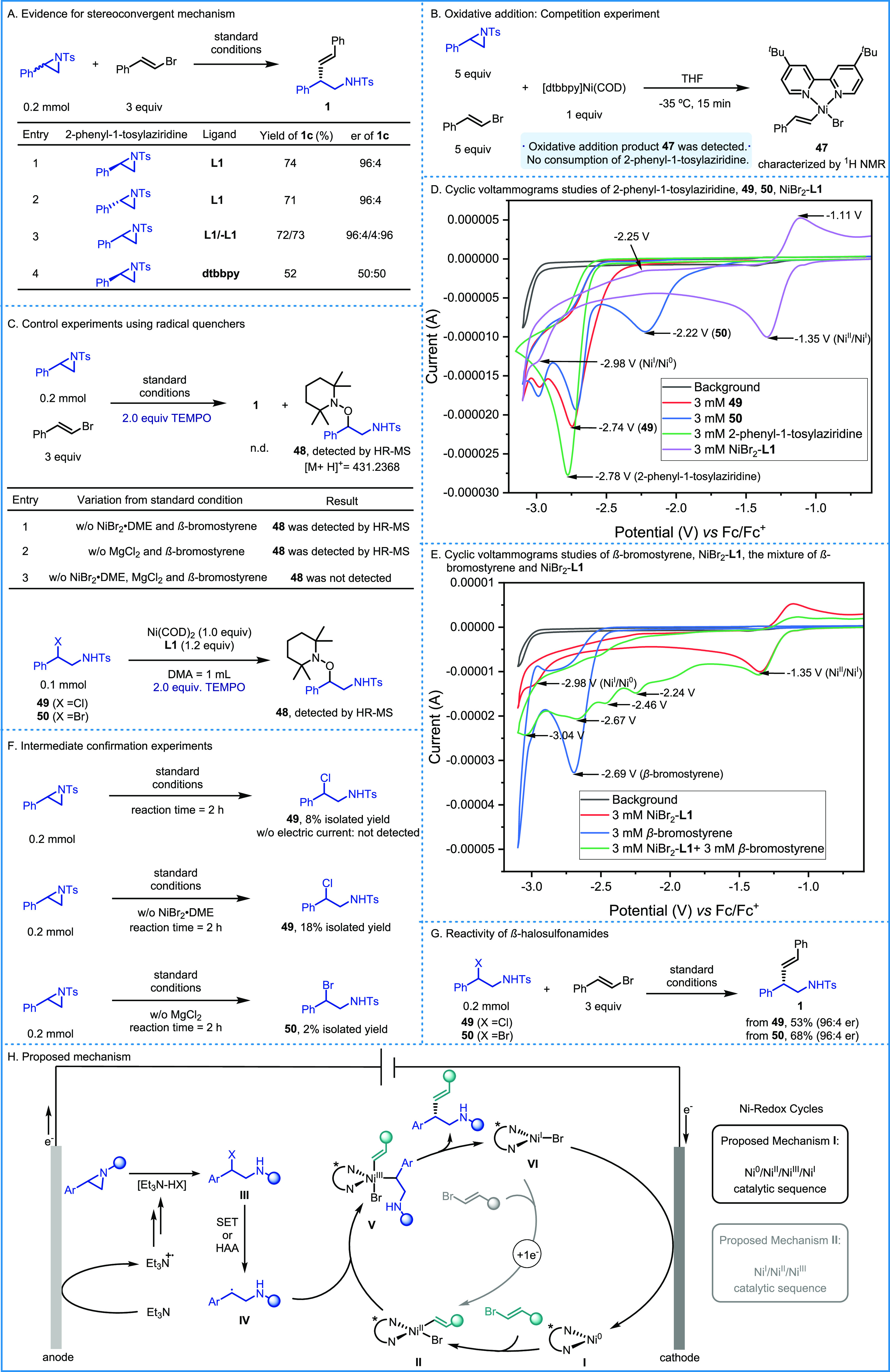
Mechanistic Studies and Proposed Mechanism

To acquire further insights into the mechanism of this transformation, several control experiments were designed.^15^ Both *R* and *S* enantiomers of 2-phenyl-1-tosylaziridine delivered the same enantioenriched product **1** under standard reaction conditions. The major enantiomer of the product was dictated by the stereochemistry of the ligand, demonstrating the stereoconvergent nature of this transformation (Scheme 4A, entries 1–3). The activation pathway for aziridine was investigated next.^13^ A competition experiment was designed featuring β-bromostyrene (5.0 equiv), 2-phenyl-1-tosylaziridine (5.0 equiv), and 1 equiv of [dtbbpy]Ni^0^(COD). The oxidative addition product of β-bromostyrene to Ni(0), complex **47**, was clearly detected by ^1^H NMR^15^ with no detectable consumption of 2-phenyl-1-tosylaziridine, which indicates that the activation of aziridines through oxidative addition to Ni(0) is not a favorable process under the reaction conditions (Scheme 4B). A direct single-electron reductive activation was also considered. As shown in Scheme 4A, entry 4, the reaction of (*R*)-*N*-*p*-tolylsulfonyl-2-phenylaziridine in the presence of 4,4′-di-*tert-*butyl-2,2′-bipyridine ligand furnished the corresponding product **1** in racemic form, thus hinting toward the intermediacy of a benzyl radical derived from aziridine under the applied conditions. Further investigations were carried out involving radical quenchers (Scheme 4C, top). The reaction was completely suppressed by adding 2,2,6,6-tetramethyl-1-piperidinyloxy (TEMPO, 2.0 equiv), and the TEMPO-benzyl adduct **48** could be detected by high-resolution mass spectrometry (HR-MS) in the mixture. Adduct **48** could still be detected when the reaction was performed in the absence of MgCl_2_ or NiBr_2_·DME. However, when neither MgCl_2_ nor NiBr_2_·DME was present, **48** could not be found in the reaction mixture. These results deem a direct single-electron reductive activation of aziridines unlikely. Still, the participation of benzyl radicals in the reaction could be justified by the formation and subsequent reduction of β-halo-sulfonamides under the utilized conditions. Reduction of the β-halo-sulfonamides could occur either directly at the cathode or by *in situ*-generated low-valent nickel species. The former possibility is supported by the fact that the TEMPO adduct **48** can be formed in the absence of the nickel catalyst. On the other hand, the reaction of β-halo-sulfonamides with stoichiometric Ni(COD)_2_/**L1** also delivered the TEMPO adduct **48**, thus indicating that Ni^0^**L1** species can also reduce the β-halo-sulfonamide to the benzyl radical (Scheme 4C, bottom). Cyclic voltammetry (CV) studies are also consistent with these results (Scheme 4D). The reductive potential of Ni^I^/Ni^0^ (*E*_1/2_ = −2.62 V *vs* Fc/Fc^+^ in DMA, reductive peak observed at −2.98 V) is more negative than those of β-chloro-sulfonamide **49** (*E*_1/2_ = −2.61 V *vs* Fc/Fc^+^ in DMA, reductive peak observed at −2.74 V) and β-bromo-sulfonamide **50** (*E*_1/2_ = −2.06 V *vs* Fc/Fc^+^ in DMA, reductive peak observed at −2.22 V), indicating that the putative β-halo-sulfonamide intermediates can indeed be reduced by Ni^0^**L1** species. These species are also more easily reduced than 2-phenyl-1-tosylaziridine (*E*_1/2_ = −2.70 V *vs* Fc/Fc^+^ in DMA, reductive peak observed at −2.78 V) so that, once formed, one would expect them to be preferentially reduced over the corresponding starting material. In contrast to previous reports,^12f,18^ the Ni^I^Br**L1** (*E*_1/2_ (Ni^II^/Ni^I^) = −1.23 V *vs* Fc/Fc^+^ in DMA, reductive peak observed at −1.35 V) is not competent for reducing the secondary halogens in the present reaction system. In order to unravel whether Ni(I) can undergo oxidative addition with alkenyl bromide, the cyclic voltammetry of NiBr_2_-**L1** was carried out in the presence of 1.0 equiv of β-bromostyrene (Scheme 4E). Some new reductive peaks appeared, suggesting that the oxidative addition of alkenyl bromide to Ni(I) is also a feasible process.

To gain additional insights into the participation of putative halogenated intermediates, the reaction was analyzed by MS (Scheme 4F). Interestingly, β-chloro-sulfonamide **49** can be isolated in 8% yield after 2 h of electrolysis in the absence of alkenyl bromide and in 18% yield when both NiBr_2_·DME and alkenyl bromide are removed from the reaction mixture. In sharp contrast, **49** was not detected in the absence of an electric current. These results hint toward a potential activation *via* nucleophilic halide ring-opening of the aziridine by *in situ*-formed R_3_N–HX (X = Cl or Br), although neither MgCl_2_ nor NiBr_2_·DME seem to be essential to this activation process. Since the reaction can also proceed in the absence of the MgCl_2_ additive, bromides are likely implicated in the nucleophilic ring opening process of the phenyl aziridine partners used in this transformation. As expected, we observed the formation of β-bromo-sulfonamide in the absence of MgCl_2_, but it is not detected under the standard conditions as a result of its facile reduction compared to the corresponding chloride under the utilized electrochemical conditions (Scheme 4F). Last, we aimed to demonstrate whether or not the proposed β-halo-sulfonamides can indeed behave as productive intermediates. When β-chloro-sulfonamide (**49**) and β-bromo-sulfonamide (**50**) were subjected to the standard reaction conditions, the cross-coupled product **1** was obtained in 53 and 68% yield, respectively. The enantiomeric ratio was identical to that obtained with the aziridine precursor (Scheme 4G). Further, experiments combining different catalytic amounts of **49** or **50** (0.1–0.3 equiv) with 4-(1-tosylaziridin-2-yl)phenyl acetate (0.9–0.7 equiv) under the reaction conditions generated products **1** and **4** in consistent high yields with respect to the corresponding precursors (see Section 7-6 in the Supporting Information).^15^ These results indeed support the idea of β-halo-sulfonamides as productive intermediates in the present transformation.

Based on the abovementioned investigations, two plausible mechanisms for this nickel-catalyzed electrochemical reductive cross-coupling can be proposed in Scheme 4H. The first one involves a Ni^0^/Ni^II^/Ni^III^/Ni^I^ catalytic sequence, wherein the oxidative addition of alkenyl bromide to Ni(0) **I** generates Ni(II) species **II**. In parallel, *in situ*-generated R_3_N–HX (X = Cl or Br) can mediate the nucleophilic halide ring-opening of the aryl aziridine delivering β-halo-sulfonamide intermediate **III**. Single-electron transfer (SET, through cathodic reduction or with Ni^0^**L1**) or halogen atom abstraction (HAA)^19^ can furnish the corresponding benzyl radical **IV**, which can then recombine with nickel-complex **II** to form Ni(III) species **V**. Reductive elimination produces the observed cross-coupled product, and the resulting Ni(I) species **VI** can be reduced to regenerate the Ni(0) at the cathode. This process is supported by the result that the operating potential of the cathode (−3.05 V *vs* Fc/Fc^+^) is more negative than that of Ni^I^/Ni^0^ (*E*_1/2_ (Ni^I^/Ni^0^) = −2.62 V *vs* Fc/Fc^+^) so that the cathode is competent to reduce Ni(I) species **VI** to Ni(0) **I** (see Section 7-8 in the Supporting Information).^15^ Concomitant anodic oxidation of Et_3_N to its radical cation is key to bypass the need for a sacrificial anode (Scheme 4H, black). The second pathway involves a Ni^I^/Ni^II^/Ni^III^ catalytic sequence.^11e^ As shown in Scheme 4E, the Ni(II)Br_2_ species can be reduced to Ni(I)Br **VI** at the cathode, and the subsequent oxidative addition of alkenyl bromide can directly deliver the key alkenyl-Ni(II) complex **II** under the reducing reaction conditions (Scheme 4H, gray).

## Conclusions

In summary, the first example of a nickel-catalyzed enantioselective electrochemical reductive cross-coupling between aryl aziridines and alkenyl bromides using triethylamine as the terminal reductant is presented here. Active metal electrodes are not required as sacrificial anodes, making this method more atom-economical and scalable for synthetic applications. The transformation exhibits a broad substrate scope and excellent functional group tolerance, allowing efficient access to chiral β-aryl homoallylic amines with high enantioselectivities and excellent *E*-stereoselectivity. The synthetic potential of this methodology has been demonstrated by its successful application to pharmacologically relevant substrates, scalability, and subsequent derivatization of the products. Mechanistic studies indicate that this transformation is consistent with a stereoconvergent mechanism in which β-halo-sulfonamides generated through nucleophilic halide ring-opening are likely intermediates along the reaction pathway. We believe that the lessons obtained here from combining electro-reduction with organic reductants will inspire the development of enantioselective electrochemical reductive cross-coupling reactions in the future.

## Supporting Information Available

Experimental procedures, characterization data, NMR spectra, HPLC traces, and crystallographic data (PDF)
