# Supplementary material for: Nickel-Catalyzed Enantioselective Electrochemical Reductive Cross-Coupling of Aryl Aziridines with Alkenyl Bromides
Source: J Am Chem Soc. 2023 Mar 7;145(11):6270–9. doi: 10.1021/jacs.2c12869 (PMC10037331; doi:10.1021/jacs.2c12869)
Supplement: Supplementary file 1 — ja2c12869_si_001.pdf [file ja2c12869_si_001.pdf]

Nickel-Catalyzed Enantioselective Electrochemical Reductive Cross-Coupling of Aryl Aziridines with Alkenyl Bromides

Xia Hu, Iván Cheng-Sánchez, Sergio Cuesta-Galisteo, Cristina Nevado*
Department of Chemistry, University of Zurich, Winterthurerstrasse 190, CH 8057
Zurich, Switzerland.

Table of content

1. General information S2

2. Optimization of the Reaction Conditions S3

3. Experimental Procedures and Characterization Data of Aziridines S8

4. Experimental Procedures and Characterization Data of Alkenyl Bromides S10

5. Experimental Procedures and Characterization Data of Products S14

6. Synthetic Transformations of Products S61

7. Mechanistic Investigations S66

   7-1. Evidence for stereoconvergent mechanism S66

   7-2. Oxidative addition: competition experiments S66

   7-3. Radical capture experiments and radical formation studies S70

   7-4. Cyclic voltammetry studies S72

   7-5. Intermediate confirmation experiment S76

   7-6. Reactivity of *β*-halo-sulfonamide S77

   7-7. Conversion of 2-phenyl-1-tosylaziridine to *β*-halo-sulfonamide with Et_3N-HX S78

   7-8. Measurement of cathodic operating potential S79

8. X-Ray Diffraction Analysis of Compound **1** S80

9. References S82

10. NMR Spectra S83

1. General information

Unless otherwise stated, reactions were carried out using dry solvents under nitrogen atmosphere. Starting materials were purchased from Aldrich, Fluka and TCI. Pentane, hexane and ethyl acetate were purchased with HPLC quality, degassed by purging thoroughly with nitrogen and dried over activated molecular sieves of appropriate size. Alternatively, tetrahydrofuran was dried using a solvent purification system (Pure Solv PS-MD-4EN, Innovative Technology Inc.) equipped with alumina drying columns under argon. Conversion was monitored by thin layer chromatography (TLC) using Merck TLC silica gel 60 F254 and visualized by UV light at 254 nm. Flash column chromatography was performed over silica gel (230-400 mesh). All electrolysis reactions were performed on ElectraSyn 2.0 with oven-dried vial (5 mL or 10 mL) unless otherwise noted. The nickel foam electrode was purchased from Xiamen Tmax Battery Equipments Limited and cut into 5.25 x 0.8 x 0.2 cm^3 plate. The graphite plate (5.25 x 0.8 x 0.2 cm^3) was purchased from IKA and could recyclable use. General procedure to recycle the graphite: after reaction, the graphite was sequentially washed with water and acetone. Then the graphite was immersed in acetone and ultrasonic cleaning for 10 minutes. After rinsing with acetone, the graphite was polished with sand paper and dried in the oven at 100 ºC before use. All NMR spectra were recorded on AV2-300, AV2-400 or AV2-500 MHz Bruker spectrometers. Chemical shifts are given in ppm and the spectra are calibrated using the residual chloroform signals (7.26 ppm for 1H NMR and 77.0 ppm for 13C NMR) and the residual dichloromethane signals (2.05 ppm for 1H NMR and 29.84 ppm, for 13C NMR). Multiplicities are abbreviated as follows: singlet (s), doublet (d), triplet (t), quartet (q), doublet-doublet (dd), quintet (quint), septet (sept), multiplet (m), and broad (b). Infrared spectra were recorded on a JASCO FT/IR - 4100 spectrometer. Absorptions are reported in wavenumber (cm^{-1}). High-resolution electrospray ionization and electronic impact mass spectrometry were performed on a Finnigan MAT 900 (Thermo Finnigan, San Jose, CA; USA) double focusing magnetic sector mass spectrometer. Ten spectra were acquired. A mass accuracy ≤ 2 ppm was obtained in the peak matching acquisition mode by using a solution containing 2 <l PEG200, 2 <l PPG450, and 1.5 mg NaOAc (all obtained from Sigma-Aldrich, CH-Buchs) dissolved in 100 mL MeOH (HPLC Supra grade, Scharlau, E-Barcelona) as internal standard. Melting points were measured on melting point operators: MPM-MHV from Müller + Krempel AG and are uncorrected. The enantiomeric ratios were determined by chiral HPLC analysis performed on JASCO HPLC system equipped with a PU-980 pump, a UV-970 detector, measured at 254 nm and a chiral column. Optical rotations were measured on a JASCO P-1010 polarimeter.

2. Optimization of the Reaction Conditions

Table S-1. Preliminary study[a]

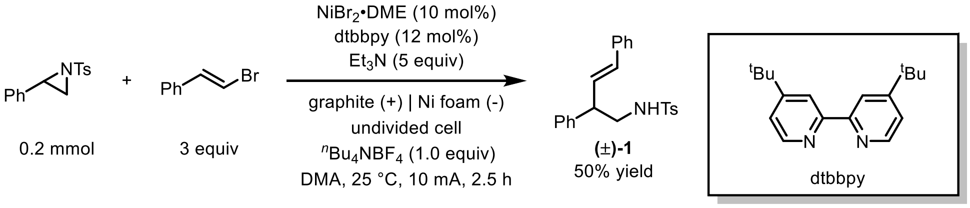

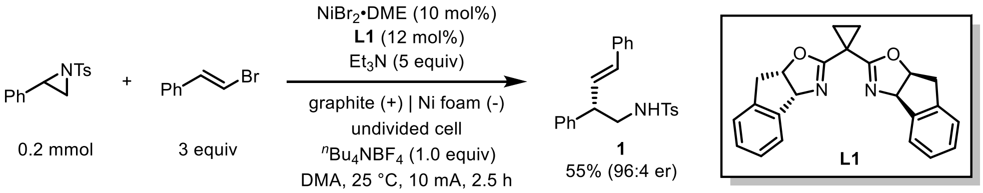

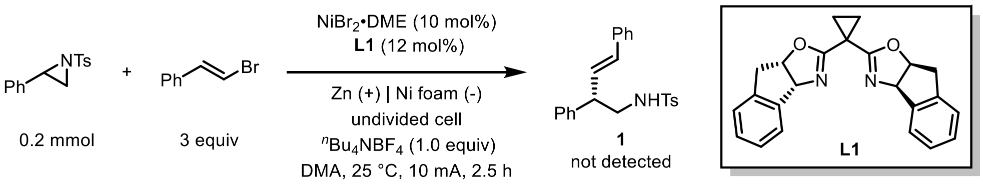

Table S-2. Effect of additives[a]

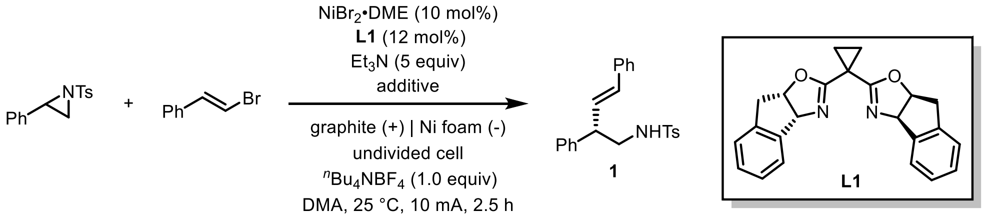

Entry	additive	yield (%)[b]	er[c]
1	without additive	56 (55)	96:4
2	$MgBr_2$ (25 mol%)	61	96:4
3	TBACl (25 mol%)	47	95:5
4	TMSCl (25 mol%)	40	95:5
5	$MgCl_2$ (25 mol%)	72 (70)	96:4
6	$MgCl_2$ (50 mol%)	69	96:4
7	TBABr (25 mol%)	44	95:5
8	TBAI (25 mol%)	42	95:5
9	$ZnCl_2$ (25 mol%)	17	96:4
10	$ZnBr_2$ (25 mol%)	41	96:4

[a]Reaction conditions: graphic anode, nickel foam cathode, 2-phenyl-1-tosylaziridin (0.1 mmol, 1.0 equiv), *β*-bromostyrene (0.3 mmol, 3.0 equiv), $^{n}Bu_4NBF_4$ (0.1 mmol, 1.0 equiv), Et_3N (0.5 mmol, 5.0 equiv), additive, $NiBr_2$•DME (0.01 mmol, 10 mol%), **L1** (0.012 mmol, 12 mol%), DMA (3.0 mL), constant current=10 mA, undivided cell, N_2, 2.5 h, 25 °C. [b]Yields were determined by ^{1}H NMR by using 1,3,5-trimethoxybenzene as internal standard, isolated yields were shown in parentheses. [c]The enantiomeric ratio (er) values were determined by chiral HPLC.

Table S-3. Effect of electrodes[a]

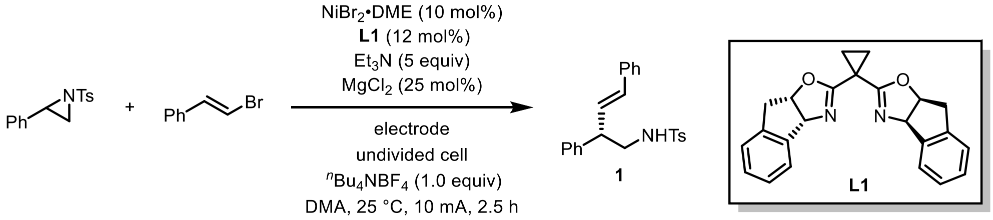

Entry	electrode	yield (%)[b]	er[c]
1	graphite (+) | Ni foam (-)	72 (70)	96:4
2	RVC (+) | Ni foam (-)	63	96:4
3	carbon felt (+) | Ni foam (-)	65	96:4
4	graphite (+) | Ni (-)	< 5%	n.d.
5	graphite (+) | Pt (-)	< 5%	n.d.

[a]Reaction conditions: corresponding electrode, 2-phenyl-1-tosylaziridine (0.1 mmol, 1.0 equiv), β-bromostyrene (0.3 mmol, 3.0 equiv), $^{n}Bu_4NBF_4$ (0.1 mmol, 1.0 equiv), Et_3N (0.5 mmol, 5.0 equiv), $MgCl_2$ (0.025 mmol, 25 mol%), [Ni] (0.01 mmol, 10 mol%), **L1** (0.012 mmol, 12 mol%), DMA (3.0 mL), constant current=10 mA, undivided cell, N_2, 2.5 h, 25 °C. [b]Yields were determined by ^{1}H NMR by using 1,3,5-trimethoxybenzene as internal standard, isolated yields were shown in parentheses. [c]The enantiomeric ratio (er) values were determined by chiral HPLC. n.d.= not determined.

Table S-4. Effect of ligands[a]

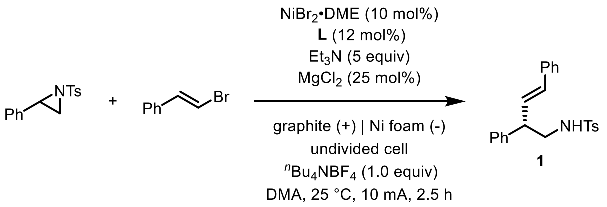

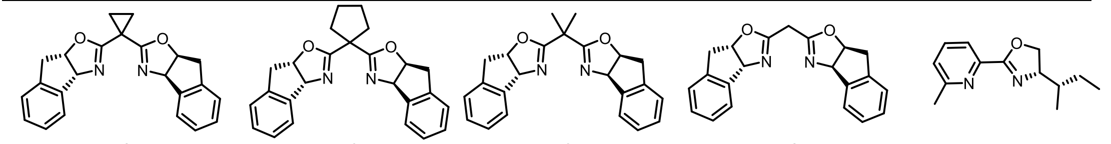

L1 72 (70%) yield[b] 96:4 er[c]; **L2** 50% yield[b] 89:11 er[c]; **L3** 45% yield[b] 73:27 er[c]; **L4** < 5% yield[b] er: n.d.; **L5** 47% yield[b] 56:44 er[c]

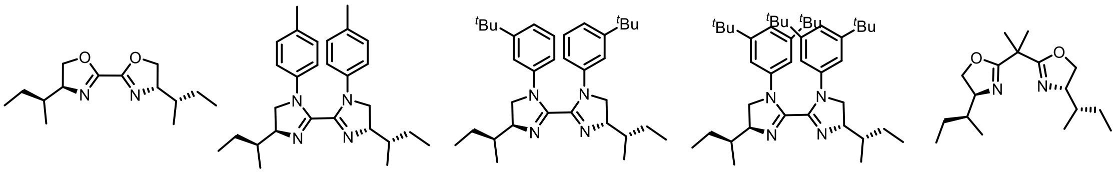

L6 37% yield[b] 16:84 er[c]; **L7** 59% yield[b] 15:85 er[c]; **L8** 71% yield[b] 10:90 er[c]; **L9** 62% yield[b] 7:93 er[c]; **L10** 50% yield[b] 15:85 er[c]

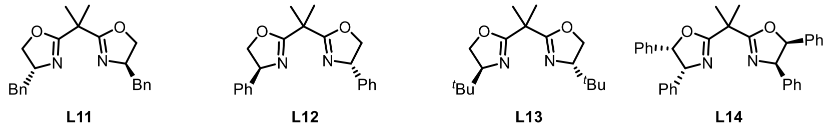

L11 52% yield[b] 84:16 er[c]; **L12** < 5% yield[b] er: n.d.; **L13** < 5% yield[b] er: n.d.; **L14** 19% yield[b] 77:23 er[c]

[a]Reaction conditions: graphic anode, nickel foam cathode, 2-phenyl-1-tosylaziridine (0.1 mmol, 1.0 equiv), β-bromostyrene (0.3 mmol, 3.0 equiv), $^{n}Bu_4NBF_4$ (0.1 mmol, 1.0 equiv), Et_3N (0.5 mmol, 5.0 equiv), $MgCl_2$ (0.025 mmol, 25 mol%), $NiBr_2$•DME (0.01 mmol, 10 mol%), **L** (0.012 mmol, 12 mol%), DMA (3.0 mL), constant current=10 mA, undivided cell, N_2, 2.5 h, 25 °C. [b]Yields were determined by ^{1}H NMR by using 1,3,5-trimethoxybenzene as internal standard, isolated yields were shown in parentheses. [c]The enantiomeric ratio (er) values were determined by chiral HPLC. n.d.= not determined.

Table S-5. Effect of nickel sources[a]

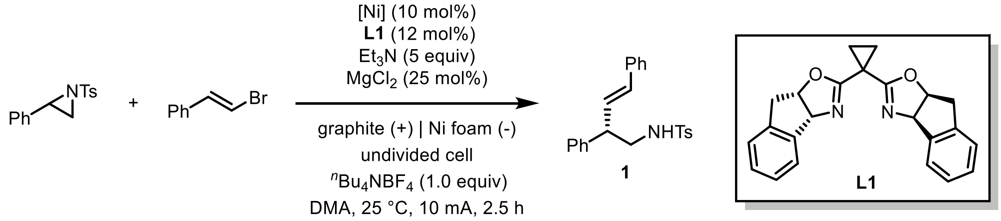

Entry	[Ni]	yield (%)[b]	er[c]
1	$NiBr_2$•DME	72 (70)	96:4
2	$NiCl_2$•DME	53	95:5
3	$NiBr_2$•diglyme	50	96:4
4	$NiBr_2$-**L1*** complex	65	95:5

[a]Reaction conditions: graphic anode, nickel foam cathode, 2-phenyl-1-tosylaziridine (0.1 mmol, 1.0 equiv), *β*-bromostyrene (0.3 mmol, 3.0 equiv), $^{n}Bu_4NBF_4$ (0.1 mmol, 1.0 equiv), Et_3N (0.5 mmol, 5.0 equiv), $MgCl_2$ (0.025 mmol, 25 mol%), [Ni] (0.01 mmol, 10 mol%), **L1** (0.012 mmol, 12 mol%), DMA (3.0 mL), constant current=10 mA, undivided cell, N_2, 2.5 h, 25 °C. [b]Yields were determined by ^1H NMR by using 1,3,5-trimethoxybenzene as internal standard, isolated yields were shown in parentheses. [c]The enantiomeric ratio (er) values were determined by chiral HPLC.

Table S-6 Effect of electrolytes[a]

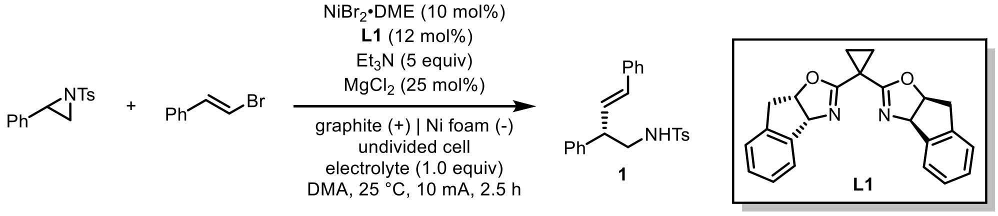

Entry	electrolyte	yield (%)[b]	er[c]
1	$^{n}Bu_4NBF_4$	72 (70)	96:4
2	$^{n}Bu_4NPF_6$	66	96:4
3	$NaBF_4$	70	95:5
4	$LiBF_4$	46	95:5
5	KPF_6	48	95:5

[a]Reaction conditions: graphic anode, nickel foam cathode, 2-phenyl-1-tosylaziridin (0.1 mmol, 1.0 equiv), *β*-bromostyrene (0.3 mmol, 3.0 equiv), electrolyte (0.1 mmol, 1.0 equiv), Et_3N (0.5 mmol, 5.0 equiv), $MgCl_2$ (0.025 mmol, 25 mol%), $NiBr_2$•DME (0.01 mmol, 10 mol%), **L1** (0.012 mmol, 12 mol%), DMA (3.0 mL), constant current=10 mA, undivided cell, N_2, 2.5 h, 25 °C. [b]Yields were determined by ^1H NMR by using 1,3,5-trimethoxybenzene as internal standard, isolated yields were shown in parentheses. [c]The enantiomeric ratio (er) values were determined by chiral HPLC.

Table S-7. Effect of nickel/ligand ratio and loading[a]

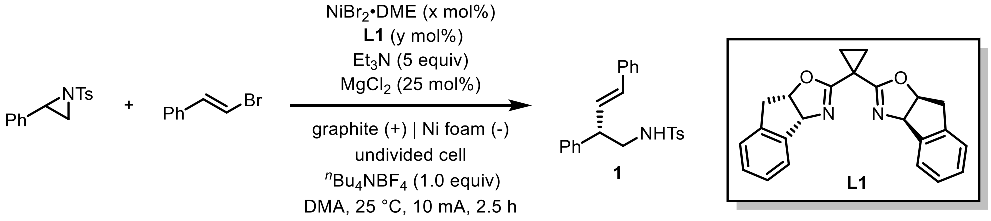

Entry	x:y	yield (%)[b]	er[c]
1	5: 6	26	96:4
2	10: 12	72 (70)	96:4
3	20: 24	72 (70)	96:4
4	10: 20	66	96:4

[a]Reaction conditions: graphic anode, nickel foam cathode, 2-phenyl-1-tosylaziridine (0.1 mmol, 1.0 equiv), *β*-bromostyrene (0.3 mmol, 3.0 equiv), $^{n}Bu_4NBF_4$ (0.1 mmol, 1.0 equiv), Et_3N (0.5 mmol, 5.0 equiv), $MgCl_2$ (0.025 mmol, 25 mol%), $NiBr_2$•DME (x mol%), **L1** (y mol%), DMA (3.0 mL), constant current=10 mA, undivided cell, N_2, 2.5 h, 25 °C. [b]Yields were determined by ^1H NMR by using 1,3,5-trimethoxybenzene as internal standard, isolated yields were shown in parentheses. [c]The enantiomeric ratio (er) values were determined by chiral HPLC.

Table S-8. Effect of current[a]

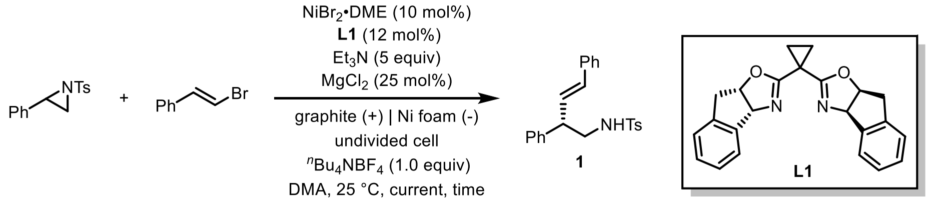

Entry	current, time	yield (%)[b]	er[c]
1	5 mA, 5 h	55	96:4
2	10 mA, 2.5 h	72 (70)	96:4
3	15 mA, 1 h 40 min	60	96:4

[a]Reaction conditions: graphic anode, nickel foam cathode, 2-phenyl-1-tosylaziridine (0.1 mmol, 1.0 equiv), *β*-bromostyrene (0.3 mmol, 3.0 equiv), ^{n}Bu$_4$NBF$_4$ (0.1 mmol, 1.0 equiv), Et$_3$N (0.5 mmol, 5.0 equiv), MgCl$_2$ (0.025 mmol, 25 mol%), NiBr$_2$•DME (0.01 mmol, 10 mol%), **L1** (0.012 mmol, 12 mol%), DMA (3.0 mL), corresponding constant current and reaction time, undivided cell, N$_2$, 25 °C. [b]Yields were determined by ^{1}H NMR by using 1,3,5-trimethoxybenzene as internal standard, isolated yields were shown in parentheses. [c]The enantiomeric ratio (er) values were determined by chiral HPLC.

Table S-9. Effect of the amount of alkenyl bromide[a]

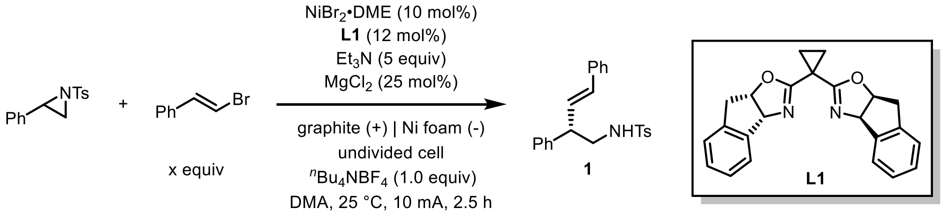

Entry	x equiv	yield (%)[b]	er[c]
1	2.0 equiv	59	96:4
2	3.0 equiv	72 (70)	96:4
3	4.0 equiv	74 (72)	96:4
4[d]	3.0 equiv	75 (73)	96:4
5[d]	4.0 equiv	75 (73)	96:4

[a]Reaction conditions: graphic anode, nickel foam cathode, 2-phenyl-1-tosylaziridine (0.1 mmol, 1.0 equiv), *β*-bromostyrene (x equiv), ^{n}Bu$_4$NBF$_4$ (0.1 mmol, 1.0 equiv), Et$_3$N (0.5 mmol, 5.0 equiv), MgCl$_2$ (0.025 mmol, 25 mol%), NiBr$_2$•DME (0.01 mmol, 10 mol%), **L1** (0.012 mmol, 12 mol%), DMA (3.0 mL), constant current=10 mA, undivided cell, N$_2$, 2.5 h, 25 °C. [b]Yields were determined by ^{1}H NMR by using 1,3,5-trimethoxybenzene as internal standard, isolated yields were shown in parentheses. [c]The enantiomeric ratio (er) values were determined by chiral HPLC. [d]reaction time=5 h.

Table S-10. Control experiments[a]

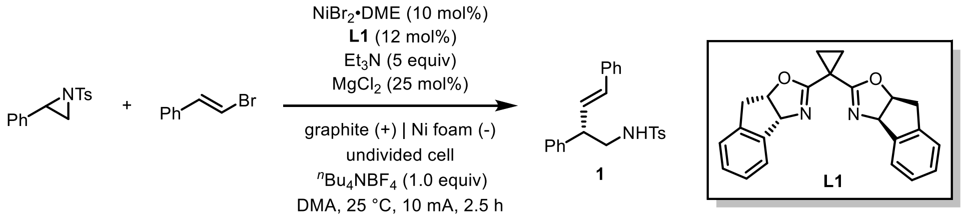

Entry	variation from above conditions	yield (%)[b]	er[c]
1	none	72 (70)	96:4
2	no electric current	0	n.d.
3	no NiBr$_2$•DME	0	n.d.
4	no ligand	0	n.d.
5[d]	3 equiv Mn instead of electric current and Et$_3$N	20	76:24
6[d]	3 equiv Zn instead of electric current and Et$_3$N	25	80:20

[a]Reaction conditions: graphic anode, nickel foam cathode, 2-phenyl-1-tosylaziridine (0.1 mmol, 1.0 equiv), *β*-bromostyrene (0.3 mmol, 3.0 equiv), ^{n}Bu$_4$NBF$_4$ (0.1 mmol, 1.0 equiv), Et$_3$N (0.5 mmol, 5.0 equiv), MgCl$_2$ (0.025 mmol, 25 mol%), NiBr$_2$•DME (0.01 mmol, 10 mol%), **L1** (0.012 mmol, 12 mol%), DMA (3.0 mL), constant current=10 mA, undivided cell, N$_2$, 2.5 h, 25 °C. [b]Yields were determined by ^{1}H NMR by using 1,3,5-trimethoxybenzene as internal standard, isolated yields were shown in parentheses. [c]The enantiomeric ratio (er) values were determined by chiral HPLC. [d]reaction time=24 h. n.d.= not determined.

Table S-11. Effect of the configuration of alkenyl bromide[a]

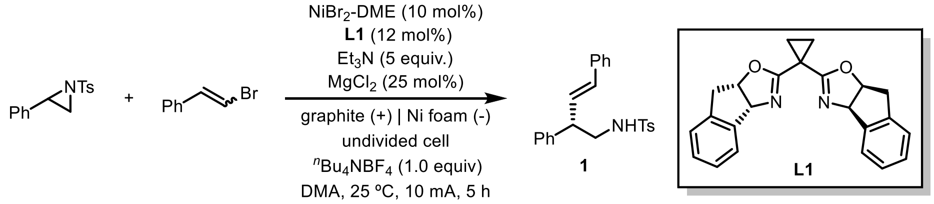

Entry	configuration of β-bromostyrene	yield (%)[b]	er[c]
1	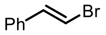	73	96:4
2	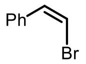	12	94:6
3	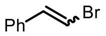 (*E*:*Z*=87:13)	71	96:4

[a]Reaction conditions: graphic anode, nickel foam cathode, 2-phenyl-1-tosylaziridine (0.2 mmol, 1.0 equiv), β-bromostyrene (0.6 mmol, 3.0 equiv), $^{n}Bu_4NBF_4$ (0.2 mmol, 1.0 equiv), Et_3N (1.0 mmol, 5.0 equiv), $MgCl_2$ (0.05 mmol, 25 mol%), $NiBr_2$•DME (0.02 mmol, 10 mol%), **L1** (0.024 mmol, 12 mol%), DMA (6.0 mL), constant current=10 mA, undivided cell, N_2, 5 h, 25 °C. [b] Isolated yields. [c]The enantiomeric ratio (er) values were determined by chiral HPLC.

3. Experimental Procedures and Characterization Data of Aziridines

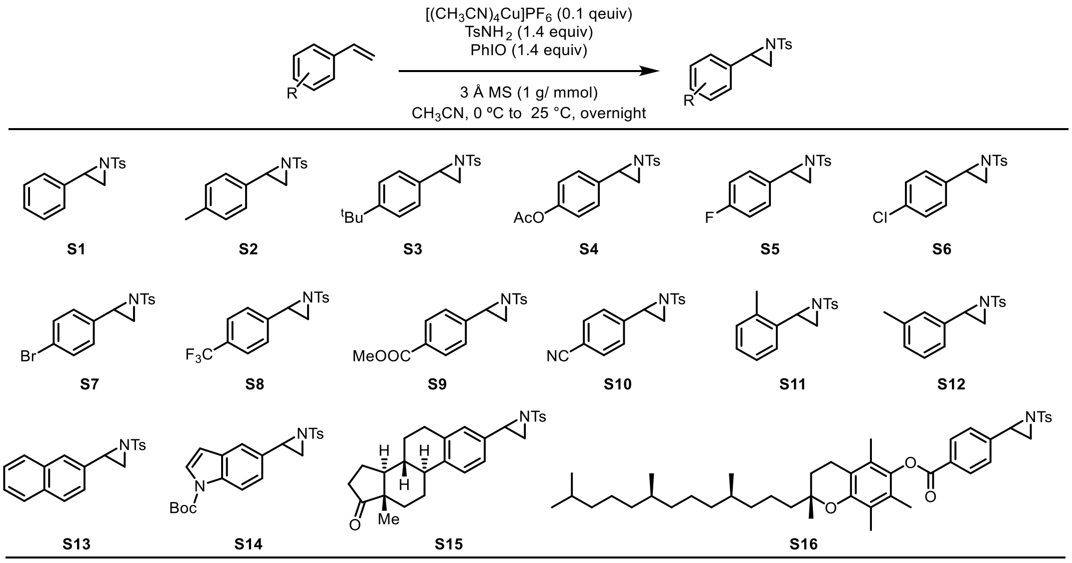

Scheme S-1. The synthesis of Ts-protected aziridines.

Procedure for the synthesis of Ts-protected aziridines: An oven-dried flask was charged with $TsNH_2$ (1.4 equiv), $Cu(MeCN)_4PF_6$ (0.1 equiv), alkene (1.0 equiv), activated 3 Å molecular sieves (need to activate in oven for 24 hours, 1.0 g/mmol alkene) and MeCN (2.5 mL/mmol alkene). The mixture was cooled in a 0 °C ice-water bath, and iodosylbenzene (1.4 equiv) was added in one portion. The mixture was allowed to warm to 25 °C and stirred for 18 hours. The resulting mixture was filtered through a pad of celite, and the filtrate was concentrated. The crude was purified by chromatography on silica gel to give the corresponding aziridines.

S1[1], **S2**[2], **S3**[2], **S4**[1], **S5**[1], **S6**[1], **S7**[2], **S8**[1], **S9**[1], **S10**[2], **S11**[1], **S12**[2], **S13**[1] and **S15**[1] were prepared according to the above procedure and analytical data are in agreement with previously reported values.

***tert*-Butyl 5-(1-tosylaziridin-2-yl)-1H-indole-1-carboxylate (S14)**

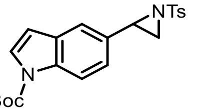

White solid. m.p.= 141-142 °C; ^{1}H NMR (400 MHz, CDCl3) δ 8.08 (d, *J* = 8.6 Hz, 1H), 7.94 – 7.86 (m, 2H), 7.60 (d, *J* = 3.7 Hz, 1H), 7.44 (d, *J* = 1.7 Hz, 1H), 7.34 (dd, *J* = 8.6, 0.8 Hz, 2H), 7.16 (dd, *J* = 8.6, 1.8 Hz, 1H), 6.52 (dd, *J* = 3.7, 0.8 Hz, 1H), 3.89 (dd, *J* = 7.1, 4.5 Hz, 1H), 3.05 (d, *J* = 7.2 Hz, 1H), 2.47 (d, *J* = 4.5 Hz, 1H), 2.45 (s, 3H), 1.68 (s, 9H); ^{13}C NMR (100 MHz, CDCl3) δ 149.5, 144.5, 135.2, 135.1, 130.6, 129.7, 129.3, 127.9, 126.7, 122.6, 119.0, 115.2, 107.0, 83.9, 41.5, 36.1, 28.2, 21.6; IR (film): ν (cm^{-1}) 2976, 1475, 1454, 1365, 1346, 1333, 1304, 1293, 1271, 1257, 1228, 1212, 1132, 1087, 1040, 1024, 945, 894, 853, 833, 814, 780, 767, 699, 670, 644, 623, 575, 557, 551, 533; HR-MS (ESI) m/z calcd for $C_{22}H_{25}N_2O_4S$ [M+H$^+$] 413.1530, found 413.1532.

(*R*)-2,5,7,8-Tetramethyl-2-((4R,8R)-4,8,12-trimethyltridecyl)chroman-6-yl 4-(1-tosylaziridin-2-yl)benzoate (S16)

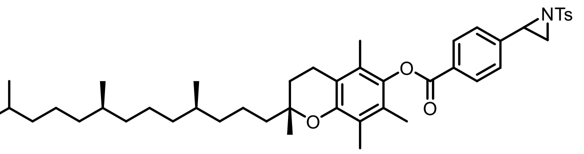

Colorless oil. ^{1}H NMR (400 MHz, $CDCl_3$) δ 8.20 (d, *J* = 8.0 Hz, 2H), 7.92 (d, *J* = 7.9 Hz, 2H), 7.45 – 7.34 (m, 4H), 3.87 (dd, *J* = 7.3, 4.3 Hz, 1H), 3.08 (d, *J* = 7.2 Hz, 1H), 2.63 (t, *J* = 6.8 Hz, 2H), 2.48 (s, 3H), 2.44 (d, *J* = 4.3 Hz, 1H), 2.14 (s, 3H), 2.05 (s, 3H), 2.01 (s,

3H), 1.83 – 1.00 (m, 31H), 0.96 – 0.82 (m, 12H); ^{13}C NMR (100 MHz, $CDCl_3$) δ 164.6, 149.5, 144.9, 140.8, 140.5, 134.8, 130.4, 129.9, 129.6, 128.0, 126.8, 126.8, 125.0, 123.2, 117.5, 75.1, 40.5, 39.6, 39.4, 37.4, 37.3, 36.2, 32.8, 31.2, 28.0, 24.8, 24.4, 23.7, 22.7, 22.6, 21.7, 21.0, 20.6, 19.8, 19.7, 13.0, 12.2, 11.8; IR (film): ν (cm^{-1}) 2949, 2925, 2866, 1736, 1727, 1461, 1414, 1379, 1330, 1271, 1236, 1177, 1163, 1111, 1017, 980, 910, 861, 821, 735, 721, 703, 693, 571; HR-MS (ESI) m/z calcd for $C_{45}H_{64}NO_5S$ [M+H$^+$] 730.4500, found 730.4502.

Synthesis of S17:

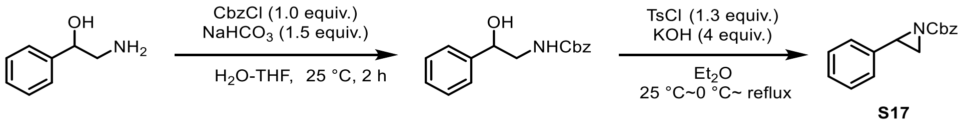

Scheme S-2. The synthesis of Cbz-protected aziridine **S17**.

Cbz-Protected aziridine **S17** was prepared according to the literature procedure[3] and analytical data are in agreement with previously reported values.

4. Experimental Procedures and Characterization Data of Alkenyl Bromides

General Procedure A:

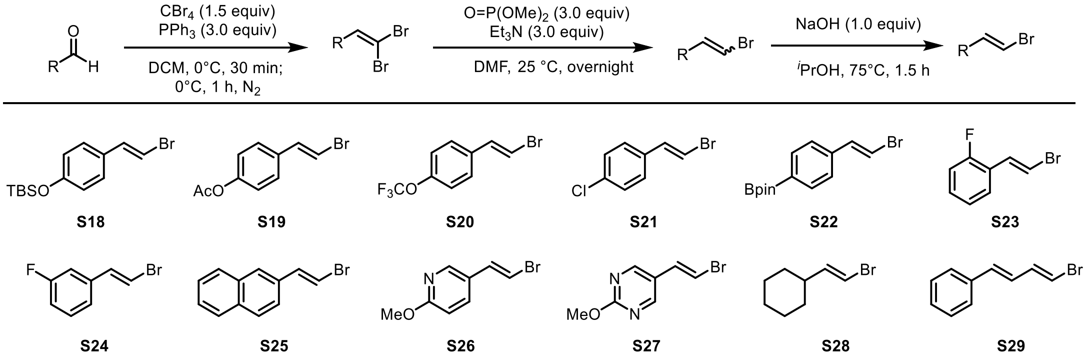

Scheme S-3. General procedure A for the synthesis of alkenyl bromides.

Step 1: To an oven-dried flask was added aldehyde (1.0 equiv), CBr_4 (1.5 equiv), and CH_2Cl_2 (0.25 M) under N_2. The flask was cooled to 0 °C, a solution of PPh_3 (3.0 equiv) in CH_2Cl_2 (1 M) was added to the reaction dropwise via addition funnel over 30 minutes. The solution was stirred at 0 °C under N_2 for 1 hour. The solution was concentrated to remove CH_2Cl_2 and $CHCl_3$ was added, the resulting mixture was filtered and washed with $CHCl_3$. The filtrate was concentrated and the crude material was purified by chromatography on silica gel to give the corresponding dibromoalkenes.

Step 2: To an oven-dried flask was added dibromoalkenes (1.0 equiv), and NEt_3 (3.0 equiv) in DMF (1 M) was added dimethylphosphonate (3.0 equiv) slowly. The solution was stirred at 25 °C for 18 hours. Water was added to the mixture, which was extracted with diethyl ether twice. The combined organic phases were washed with saturated brine and dried over $MgSO_4$, filtered, and concentrated. The crude material was purified by chromatography on silica gel to give the corresponding vinyl bromides as *E*/*Z* mixture.

Step 3: *E*/*Z* mixed vinyl bromides (1.0 equiv) was dissolved in iPrOH (0.5 M). Solid NaOH (1.0 equiv) was added and the mixture was heated to reflux for 1.5 hours. The reaction mixture was cooled to room temperature, diluted with Et_2O, and partitioned with distilled H_2O twice. The organic phase was collected, and washed with an aqueous solution of 1 M HCl, dried over $MgSO_4$, filtered, and concentrated. The crude material was purified by chromatography on silica gel to give the corresponding vinyl bromides with exclusively *E* configuration.

S18[4], **S19**[5], **S20**[4], **S21**[5], **S22**[4], **S23**[6], **S24**[6], **S25**[7], **S26**[8], **S27**[8], **S28**[4] and **S29**[4] were prepared according to general procedure A and analytical data are in agreement with previously reported values.

General Procedure B:

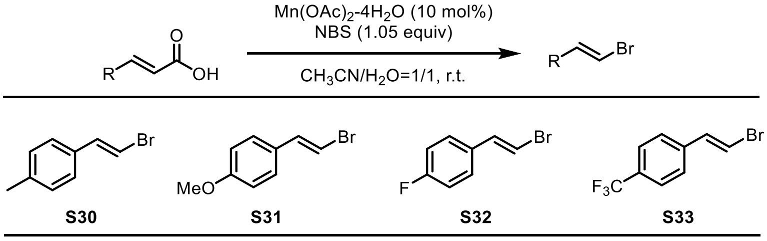

Scheme S-4. General procedure B for the synthesis of alkenyl bromides.

A round-bottomed flask was charged with cinnamyl acid derivatives (1.0 equiv), N-bromosuccinimide (NBS, 1.05 equiv), $Mn(OAc)_2·4H_2O$ (10 mol%), 1:1 H_2O/acetonitrile (1 M). The

reaction mixture was stirred at 25 °C and monitored by TLC analysis. After total conversion of substrates, acetonitrile was evaporated. The mixture was extracted by Et_2O twice. The combined organic layer was washed with saturated brine, dried over anhydrous $MgSO_4$, filtered, and concentrated. The residue was purified by chromatography on silica gel to give the corresponding vinyl bromides.

S30[4], **S31**[4], **S32**[5] and **S33**[4] were prepared according to general procedure B and analytical data are in agreement with previously reported values.

General Procedure C:

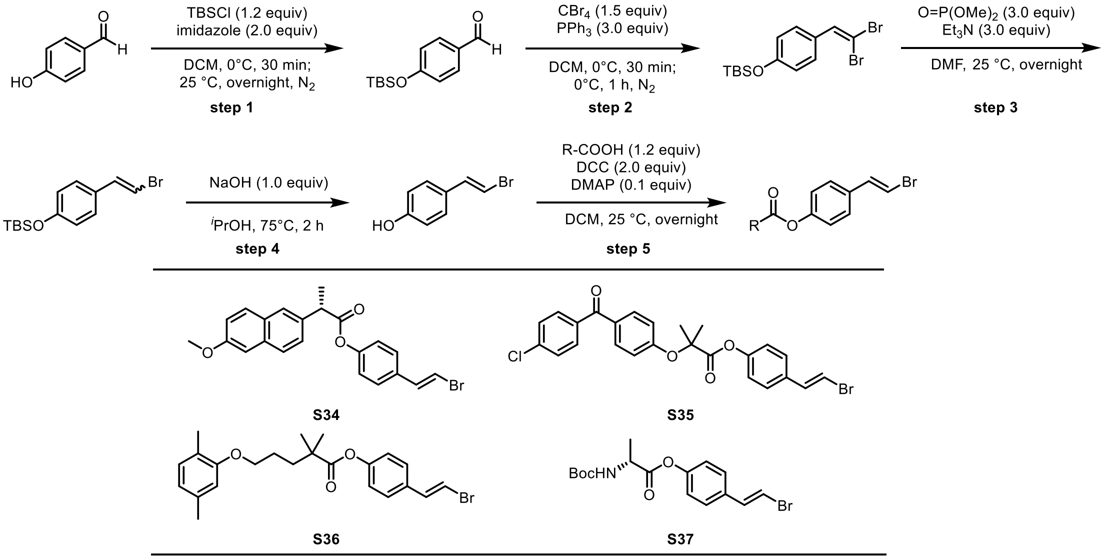

Scheme S-5. General procedure C for the synthesis of alkenyl bromides.

Step 1: To an oven-dried flask was added 4-hydroxybenzaldehyde (1.0 equiv), imidazole (2.0 equiv) and CH_2Cl_2 (0.5 M) under N_2 atmosphere. The flask was cooled to 0 °C in an ice-water bath, a solution of tert-butyldiphenylchlorosilane (TBSCl, 1.2 equiv) in CH_2Cl_2 (1 M) was added to the reaction dropwise via addition funnel. Stir the mixture at 25 °C for 18 hours, pour the reaction mixture into 150 mL of ice-water while stirring and extract the resulting mixture with DCM for three times. The combined organic layer was washed with saturated brine, dried over anhydrous $MgSO_4$, filtered, and concentrated. The residue was purified by chromatography on silica gel.

Step 2: To an oven-dried flask was added 4-((tert-butyldimethylsilyl)oxy)benzaldehyde (1.0 equiv), CBr_4 (1.5 equiv), and CH_2Cl_2 (0.25 M) under N_2. The flask was cooled to 0 °C, a solution of PPh_3 (3 equiv) in CH_2Cl_2 (1 M) was added to the reaction dropwise via addition funnel over 30 minutes. The solution was stirred at 0 °C under N_2 for 1 hour. The solution was concentrated to remove CH_2Cl_2 and $CHCl_3$ was added, the resulting mixture was filtered and washed with $CHCl_3$ twice. The filtrate was concentrated and the crude material was purified by chromatography on silica gel to give the corresponding dibromoalkenes.

Step 3: To an oven-dried flask was added tert-butyl(4-(2,2-dibromovinyl)phenoxy)dimethylsilane (1.0 equiv), and NEt_3 (3.0 equiv) in DMF (1 M) was added dimethylphosphonate (3.0 equiv) slowly. The solution was stirred at 25 °C for 18 hours. Water was added to the mixture, which was extracted with diethyl ether twice. The combined organic phases were washed with saturated brine and dried over $MgSO_4$, filtered, and concentrated. The crude material was purified by chromatography on silica gel.

Step 4: 4-(2-Bromovinyl)phenoxy (tert-butyl)dimethylsilane (1.0 equiv) was dissolved in iPrOH

(0.5 M). Solid NaOH (1.0 equiv) was added and the mixture was heated to reflux for 2 hours. The reaction mixture was cooled to room temperature, diluted with Et_2O, and partitioned with distilled H_2O twice. The organic phase was collected, and washed with an aqueous solution of 1 M HCl, dried over $MgSO_4$, filtered, and concentrated. The crude material was purified by chromatography on silica gel.

Step 5: To a solution of (*E*)-4-(2-bromovinyl)phenol (1.0 equiv), DCC (2.0 equiv) and DMAP (0.1 equiv) in DCM (0.1 M) was added acid (1.2 equiv). The reaction mixture was stirred at 25 °C for 12 hours. The resulting solution was then filtered and washed with DCM twice. The combined organic layer was concentrated, and the crude material was purified by chromatography on silica gel.

S34, **S35**, **S36** and **S37** were prepared according to general procedure **C**.

(*E*)-4-(2-Bromovinyl)phenyl (S)-2-(6-methoxynaphthalen-2-yl)propanoate (S34)

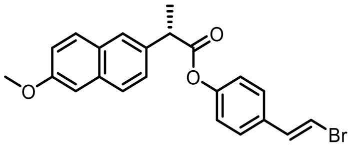

White solid. m.p.= 116-117 °C; ^{1}H NMR (400 MHz, $CDCl_3$) δ 7.71 – 7.63 (m, 3H), 7.41 (dd, *J* = 8.6, 1.7 Hz, 1H), 7.20 – 7.13 (m, 2H), 7.12 – 7.04 (m, 2H), 6.97 (d, *J* = 14.0 Hz, 1H), 6.90 – 6.84 (m, 2H), 6.62 (d, *J* = 14.0 Hz, 1H), 4.01 (q, *J* = 7.1 Hz, 1H), 3.85 (s, 3H), 1.61 (d, *J* = 7.1 Hz, 3H); ^{13}C NMR (100 MHz, $CDCl_3$) δ 173.0, 157.8, 150.7, 136.2, 135.0, 133.8, 133.6, 129.3, 129.0, 127.4, 127.0, 126.1, 126.0, 121.8, 119.1, 106.6, 105.6, 55.3, 45.6, 18.5; IR (film): ν (cm^{-1}) 3068, 2976, 2935, 1752, 1632, 1604, 1577, 1504, 1485, 1451, 1419, 1392, 1374, 1317, 1266, 1237, 1205, 1189, 1171, 1127, 1116, 1063, 1028, 1012, 954, 937, 929, 896, 852, 818, 778, 734, 682, 546, 516, 477, 473; HR-MS (ESI) m/z calcd for $C_{22}H_{20}BrO_3$ [M+H$^+$] 411.0590, found 411.0588.

(*E*)-4-(2-Bromovinyl)phenyl 2-(4-(4-chlorobenzoyl)phenoxy)-2-methylpropanoate (S35)

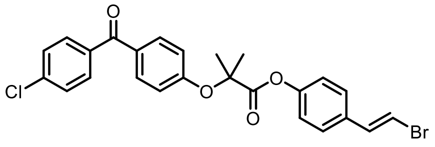

Colorless oil. ^{1}H NMR (400 MHz, $CDCl_3$) δ 7.74 – 7.68 (m, 2H), 7.67 – 7.61 (m, 2H), 7.40 – 7.35 (m, 2H), 7.24 – 7.18 (m, 2H), 7.00 (d, *J* = 14.0 Hz, 1H), 6.94 – 6.85 (m, 4H), 6.66 (d, *J* = 14.0 Hz, 1H), 1.75 (s, 6H); ^{13}C NMR (100 MHz, $CDCl_3$) δ 194.1, 172.3, 159.4, 150.1, 138.5, 136.2, 136.0, 134.1, 132.1, 131.2, 130.7, 128.6, 127.2, 121.6, 117.3, 107.1, 79.4, 25.4; IR (film): ν (cm^{-1}) 3073, 2993, 2941, 1754, 1653, 1596, 1503, 1385, 1314, 1304, 1282, 1273, 1247, 1206, 1184, 1149, 1108, 1090, 1013, 928, 851, 838, 761, 735, 522, 476; HR-MS (ESI) m/z calcd for $C_{25}H_{21}BrClO_4$ [M+H$^+$] 499.0306, found 499.0310.

(*E*)-4-(2-Bromovinyl)phenyl 5-(2,5-dimethylphenoxy)-2,2-dimethylpentanoate (S36)

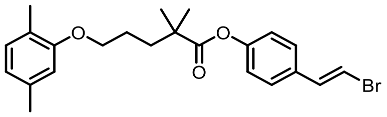

Colorless oil. ^{1}H NMR (400 MHz, $CDCl_3$) δ 7.25 – 7.17 (m, 2H), 7.01 (d, *J* = 14.0 Hz, 1H), 6.96 – 6.88 (m, 3H), 6.65 (d, *J* = 14.0 Hz, 1H), 6.59 (d, *J* = 7.5 Hz, 1H), 6.55 (s, 1H), 3.95 – 3.87 (m, 2H), 2.23 (s, 3H), 2.10 (s, 3H), 1.84 – 1.76 (m, 4H), 1.29 (s, 6H); ^{13}C NMR (100 MHz, $CDCl_3$) δ 176.2, 156.8, 150.9, 136.5, 136.2, 133.5, 130.4, 127.0, 123.6, 121.9, 120.8, 111.9, 106.5, 67.7, 42.5, 37.1, 25.2, 25.1, 21.4, 15.8; IR (film): ν (cm^{-1}) 2974, 2948, 2925, 2870, 1747, 1612, 1602, 1582, 1506, 1470, 1459, 1412, 1388, 1284, 1264, 1206, 1183, 1165, 1106, 1044, 1014, 948, 930, 892, 846, 803, 772, 735, 597, 587, 519; HR-MS (ESI) m/z calcd for $C_{23}H_{28}BrO_3$ [M+H$^+$] 431.1216, found 431.1218.

(*E*)-4-(2-Bromovinyl)phenyl (tert-butoxycarbonyl)-D-alaninate (S37)

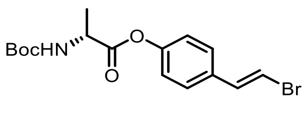

White solid. m.p.= 132-133 °C; ^{1}H NMR (400 MHz, $CDCl_3$) δ 7.28 – 7.19 (m, 2H), 7.04 – 6.97 (m, 3H), 6.67 (d, *J* = 14.0 Hz, 1H), 5.01 (d,

J = 4.6 Hz, 1H), 4.46 (t, J = 6.4 Hz, 1H), 1.47 (d, J = 7.2 Hz, 3H), 1.39 (s, 9H); ^{13}C NMR (100 MHz, $CDCl_3$) δ 171.9, 150.3, 136.1, 133.9, 130.2, 127.1, 121.7, 106.8, 80.1, 49.4, 28.3, 18.4; IR (film): ν (cm^{-1}) 3382, 3071, 2987, 2971, 2938, 1774, 1690, 1513, 1506, 1455, 1382, 1362, 1300, 1252, 1201, 1188, 1169, 1137, 1115, 1066, 1043, 1023, 1014, 960, 945, 892, 866, 835, 784, 756, 741, 586, 550, 531, 522, 507; HR-MS (ESI) m/z calcd for $C_{16}H_{21}BrNO_4$ [$M+H^+$] 370.0648, found 370.0651.

The synthesis of S38:

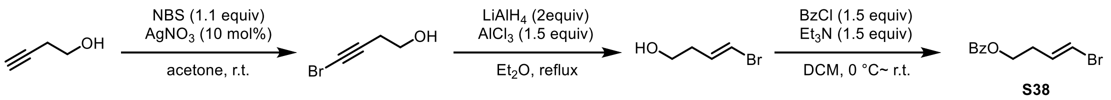

Scheme S-6. The synthesis of alkenyl bromide **S38**.

Alkenyl bromide **S38** was prepared according to the literature procedure[4] and analytical data are in agreement with previously reported values.

5. Experimental Procedures and Characterization Data of Products

General Procedure A (GP-A):

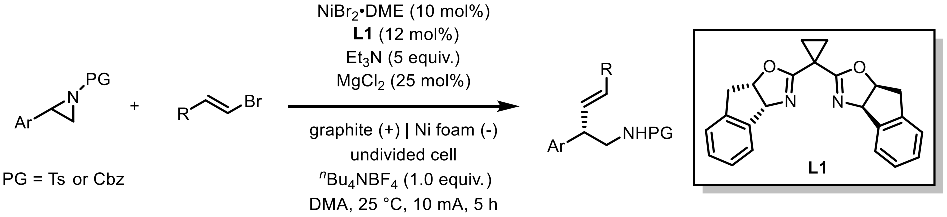

Scheme S-7. General procedure A for nickel-catalyzed enantioselective electrochemical reductive cross-coupling of aziridines with alkenyl bromides.

In a nitrogen-filled glove box, an oven-dried undivided ElectraSyn vial (10 mL) equipped with a stirring bar was charged with $NiBr_2$•DME (0.02 mmol, 10 mol%), **L1** (0.024 mmol, 12 mol%), $MgCl_2$ (0.05 mmol, 25 mol%), aziridines (0.2 mmol, 1 equiv), ${}^{n}Bu_4NBF_4$ (0.2 mmol, 1 equiv) and DMA (6 mL). The mixture was stirred at room temperature for 2 minutes. Et_3N (1 mmol, 5 equiv), alkenyl bromides (0.6 mmol, 3 equiv) were sequentially added. The vial was sealed with the ElectraSyn vial cap equipped with anode (graphite plate) and cathode (nickel foam plate), and then bring it out of glove box. After pre-stirring the resulting mixture for 2 minutes, a constant current of 10 mA was applied for 5 hours at 25 °C. After electrolysis, the reaction vial was disconnected from ElectraSyn 2.0, gently remove the cap with electrodes from the vial and transfer the reaction media to a separatory funnel. Both electrodes were rinsed with EtOAc. Then, water was added and the resulting mixture was extracted with EtOAc (3*10 mL). The organic layer was washed with brine (2*15 mL) and dried over with anhydrous magnesium sulfate. After concentration under reduced pressure, the crude mixture was purified by chromatography on silica gel with hexane: ethyl acetate mixtures as eluent to give the corresponding products.

General Procedure B (GP-B):

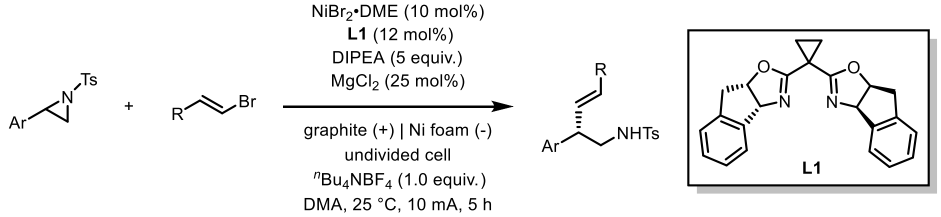

Scheme S-8. General procedure B for nickel-catalyzed enantioselective electrochemical reductive cross-coupling of aziridines with alkenyl bromides.

In a nitrogen-filled glove box, an oven-dried undivided ElectraSyn vial (10 mL) equipped with a stirring bar was charged with $NiBr_2$•DME (0.02 mmol, 10 mol%), **L1** (0.024 mmol, 12 mol%), $MgCl_2$ (0.05 mmol, 25 mol%), aziridines (0.2 mmol, 1 equiv), ${}^{n}Bu_4NBF_4$ (0.2 mmol, 1 equiv) and DMA (6 mL). The mixture was stirred at room temperature for 2 minutes. DIPEA (1 mmol, 5 equiv), alkenyl bromides (0.6 mmol, 3 equiv) were sequentially added. The vial was sealed with the ElectraSyn vial cap equipped with anode (graphite plate) and cathode (nickel foam plate), and then bring it out of glove box. After pre-stirring the resulting mixture for 2 minutes, a constant current of 10 mA was applied for 5 hours at 25 °C. After electrolysis, the reaction vial was disconnected from ElectraSyn 2.0, gently remove the cap with electrodes from the vial and transfer the reaction media to a separatory funnel. Both electrodes were rinsed with EtOAc. Then, water was added and the

resulting mixture was extracted with EtOAc (3*10 mL). The organic layer was washed with brine (2*15 mL) and dried over with anhydrous magnesium sulfate. After concentration under reduced pressure, the crude mixture was purified by chromatography on silica gel with hexane: ethyl acetate mixtures as eluent to give the corresponding products.

General Procedure C for 34 (GP-C):

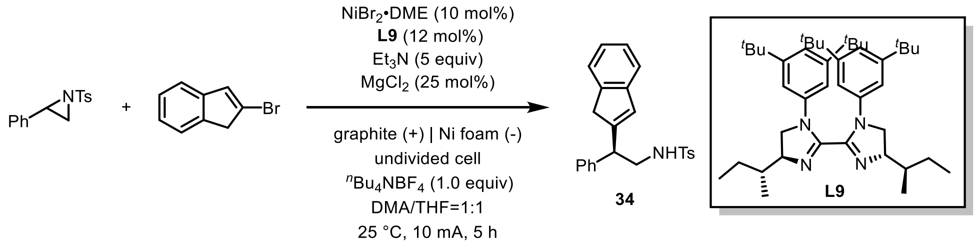

Scheme S-9. General procedure C for nickel-catalyzed enantioselective electrochemical reductive cross-coupling of aziridines with alkenyl bromides.

In a nitrogen-filled glove box, an oven-dried undivided ElectraSyn vial (10 mL) equipped with a stirring bar was charged with $NiBr_2$•DME (0.02 mmol, 10 mol%), **L1** (0.024 mmol, 12 mol%), $MgCl_2$ (0.05 mmol, 25 mol%), aziridines (0.2 mmol, 1 equiv), $^nBu_4NBF_4$ (0.2 mmol, 1 equiv), DMA (3 mL) and THF (3 mL). The mixture was stirred at room temperature for 2 minutes. Et_3N (1 mmol, 5 equiv), alkenyl bromides (0.6 mmol, 3 equiv) were sequentially added. The vial was sealed with the ElectraSyn vial cap equipped with anode (graphite plate) and cathode (nickel foam plate), and then bring it out of glove box. After pre-stirring the resulting mixture for 2 minutes, a constant current of 10 mA was applied for 5 hours at 25 ^{o}C. After electrolysis, the reaction vial was disconnected from ElectraSyn 2.0, gently remove the cap with electrodes from the vial and transfer the reaction media to a separatory funnel. Both electrodes were rinsed with EtOAc. Then, water was added and the resulting mixture was extracted with EtOAc (3*10 mL). The organic layer was washed with brine (2*15 mL) and dried over with anhydrous magnesium sulfate. After concentration under reduced pressure, the crude mixture was purified by chromatography on silica gel with hexane: ethyl acetate mixtures as eluent to give the corresponding product **34**.

Gram-scale synthesis:

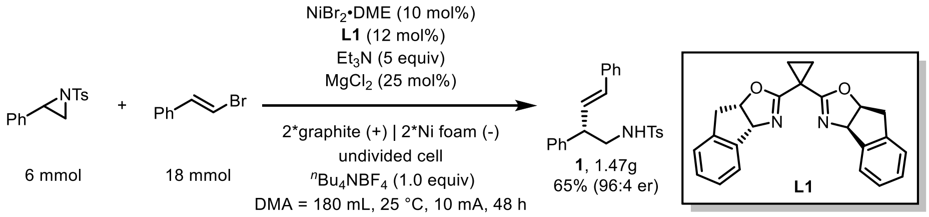

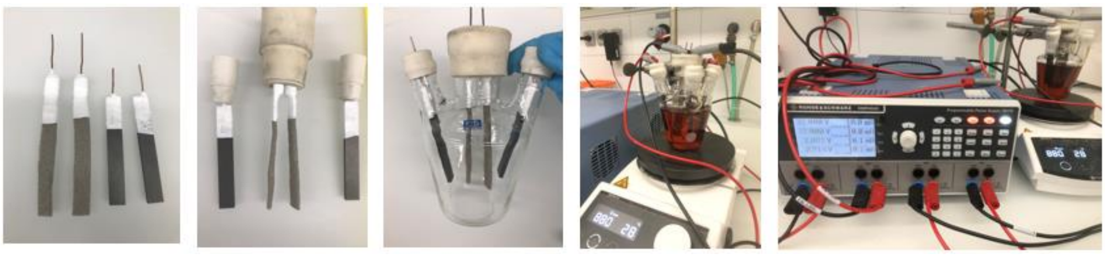

Scheme S-10. General procedure for gram-scale synthesis.

In a nitrogen-filled glove box, an oven-dried four-neck flask (200 mL) equipped with a stirring bar was charged with $NiBr_2$•DME (0.6 mmol, 10 mol%), **L1** (0.72 mmol, 12 mol%), $MgCl_2$ (1.5 mmol,

25 mol%), 2-phenyl-1-tosylaziridine (6 mmol, 1 equiv), $^{n}Bu_4NBF_4$ (6 mmol, 1 equiv) and DMA (180 mL). The mixture was stirred at room temperature for 2 minutes. Et_3N (30 mmol, 5 equiv), *β*-bromostyrene (18 mmol, 3 equiv) were sequentially added. The flask was equipped with anode (graphite plate*2) and cathode (nickel foam plate*2), and then bring it out of glove box. After pre-stirring the resulting mixture for 2 minutes, a constant current of 10 mA was applied for 48 h at 25 °C by using two channels of Rohde & Schwarz HMP4040. After electrolysis, the reaction flask was disconnected from Rohde & Schwarz HMP4040, gently remove the cap with electrodes from the vial and transfer the reaction media to a separatory funnel. Both electrodes were rinsed with EtOAc. Then, water was added and the resulting mixture was extracted with EtOAc (3*200 mL). The organic layer was washed with brine (2*300 mL) and dried over with anhydrous magnesium sulfate. After concentration under reduced pressure, the crude mixture was purified by chromatography on silica gel with hexane: ethyl acetate mixtures as eluent to give the corresponding product **1** in 65% yield and 96:4 er.

Table S-12. Effects of DIPEA and Et_3N on some substrates. [a]

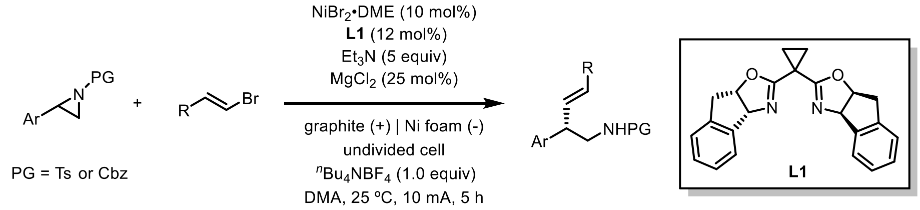

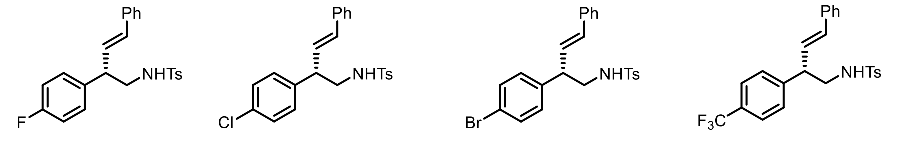

5, 62% (94:6 er)[a] 65% (95:5 er)[b]

6, 63% (94:6 er)[a] 63% (94:6 er)[b]

7, 65% (93:7 er)[a] 35% (91:9 er)[b]

8, 60% (90:10 er)[a] 45% (90:10 er)[b]

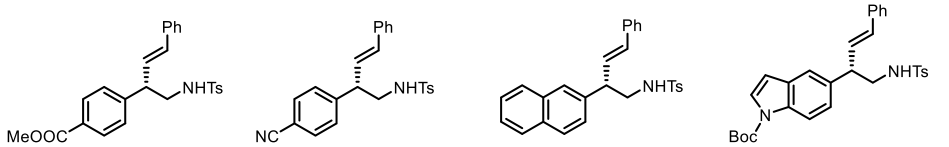

9, 75% (94:6 er)[a] 38% (95:5 er)[b]

10, 60% (91:9 er)[a] 30% (93:7 er)[b]

13, 67% (92:8 er)[a] 72% (95:5 er)[b]

14, 49% (90:10 er)[a] 44% (89:11 er)[b]

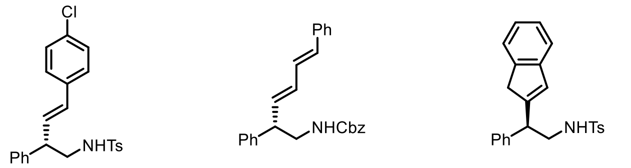

21, 78% (94:6 er)[a] 75% (97:3 er)[b]

33[c], 64% (90:10 er)[a] N.D.[d]

34[d], 49% (91:9 er)[a] N.D.[d]

[a]Reaction condition: graphite anode, nickel foam cathode, aziridines (0.2 mmol, 1.0 equiv), alkenyl bromides (0.6 mmol, 3.0 equiv), $^{n}Bu_4NBF_4$ (0.2 mmol, 1.0 equiv), Et3N (1.0 mmol, 5.0 equiv), $MgCl_2$ (0.05 mmol, 25 mol%), $NiBr_2$•DME (0.02 mmol, 10 mol%), **L1** (0.024 mmol, 12 mol%), DMA (6.0 mL), constant current=10 mA, undivided cell, N_2, 5 h, 25 °C, isolated yields were shown, enantiomer ratio (er) values were determined by chiral HPLC. [b] 5 equiv DIPEA inatead of 5 equiv Et_3N. [c]20 mol% NiBr2•DME and 24 mol% **L1**. [d]**L9** was used as ligand, DMA/THF = 3 mL/3 mL was used as solvent.

Table S-13. Nickel-catalyzed enantioselective electrochemical reductive cross-coupling of aryl aziridines with aryl bromides. [a]

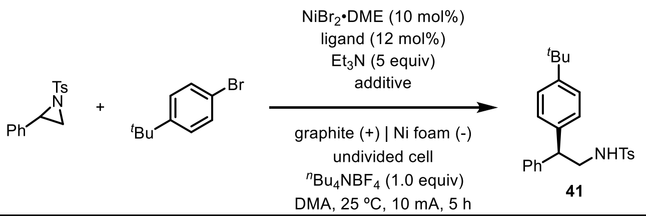

Entry	ligand	additive	yield (%)[b]	er[c]
1	**L1**	$MgCl_2$ (25 mol%)	N.D.	-
2	**L8**	$MgCl_2$ (25 mol%)	25	83:17
3	**L8**	$MgCl_2$ (1.0 equiv)	42	84:16
4	**L9**	$MgCl_2$ (1.0 equiv)	40	90:10

[a]Reaction conditions: graphic anode, nickel foam cathode, 2-phenyl-1-tosylaziridine (0.2 mmol, 1.0 equiv), 1-bromo-4-(*tert*-butyl)benzene (0.6 mmol, 3.0 equiv), $^{n}Bu_4NBF_4$ (0.2 mmol, 1.0 equiv), Et_3N (1.0 mmol, 5.0 equiv), additive, $NiBr_2$•DME (0.02 mmol, 10 mol%), ligand (0.024 mmol, 12 mol%), DMA (6.0 mL), constant current=10 mA, undivided cell, N_2, 5 h, 25 °C. [b]Isolated yields. [c]The enantiomeric ratio (er) values were determined by chiral HPLC.

Table S-14. Nickel-catalyzed enantioselective electrochemical reductive cross-coupling of benzyl halides with alkenyl bromides. [a]

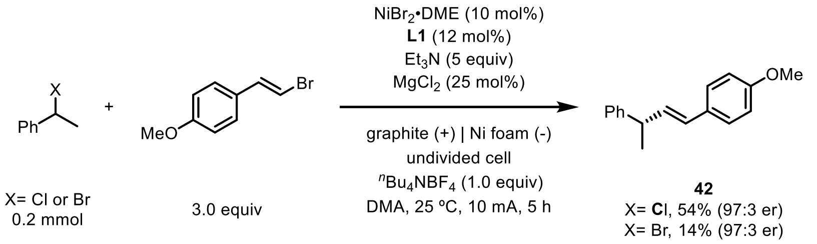

[a]Reaction conditions: graphic anode, nickel foam cathode,(1-chloroethyl)benzene or (1-bromoethyl)benzene (0.2 mmol, 1.0 equiv), (*E*)-1-(2-bromovinyl)-4-methoxybenzene (0.6 mmol, 3.0 equiv), $^{n}Bu_4NBF_4$ (0.2 mmol, 1.0 equiv), Et_3N (1.0 mmol, 5.0 equiv), $MgCl_2$ (0.05 mmol, 25 mol%), $NiBr_2$•DME (0.02 mmol, 10 mol%), ligand (0.024 mmol, 12 mol%), DMA (6.0 mL), constant current=10 mA, undivided cell, N_2, 5 h, 25 °C. [b]Isolated yields. [c]The enantiomeric ratio (er) values were determined by chiral HPLC.

Table S-15. Unsuccessful examples.

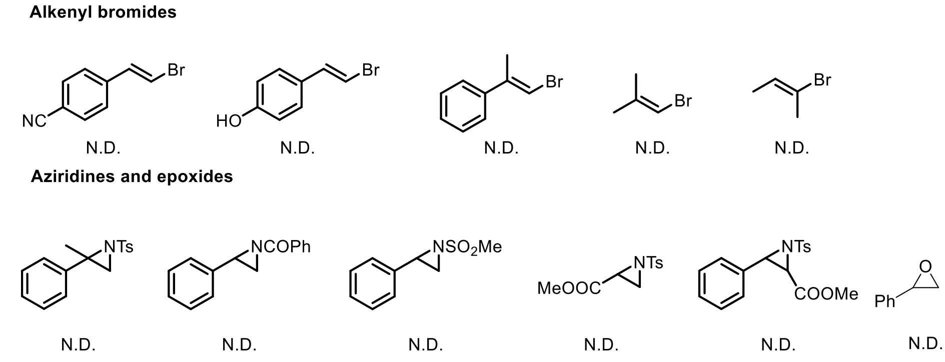

(*S*, *E*)-*N*-(2, 4-Diphenylbut-3-en-1-yl)-4-methylbenzenesulfonamide (1)

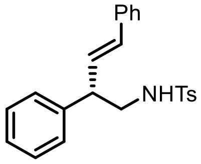

Prepared by **GP-A**. White solid, 55.1 mg, 73% yield. m.p. = 148-149 °C; ^{1}H NMR (400 MHz, $CDCl_3$) δ 7.68 – 7.54 (m, 2H), 7.26 – 7.10 (m, 10H), 7.08 – 7.01 (m, 2H), 6.29 (dd, *J* = 15.9, 0.8 Hz, 1H), 6.09 (dd, J = 15.9, 7.9 Hz, 1H), 4.44 (t, *J* = 6.2 Hz, 1H), 3.49 (q, *J* = 7.6 Hz, 1H), 3.32 – 3.14 (m, 2H), 2.34 (s, 3H); ^{13}C NMR (101 MHz, $CDCl_3$) δ 143.4, 140.2, 136.8, 136.5, 132.2, 129.7, 129.2, 128.9, 128.5, 127.6, 127.2, 127.1, 126.3, 48.6, 47.5, 21.5; IR (film): ν (cm^{-1}) 3295, 3275, 3024, 2917, 1596, 1495, 1420, 1318, 1289, 1150, 1090, 1067, 961, 862, 819, 748, 694, 665; HR-

MS (ESI) m/z calcd for $C_{23}H_{24}NO_2S$ [M+H$^+$] 378.1522, found 378.1521; $[\alpha]_D^{22.2}$ = -16.5 (c = 0.1, $CHCl_3$); HPLC conditions: AD-H column, hexane/2-propanol = 90/10, flow rate = 1.0 mL/min, λ = 254 nm, t_R = 25.0 min (minor), t_R = 27.5 min (major), 96:4 er.

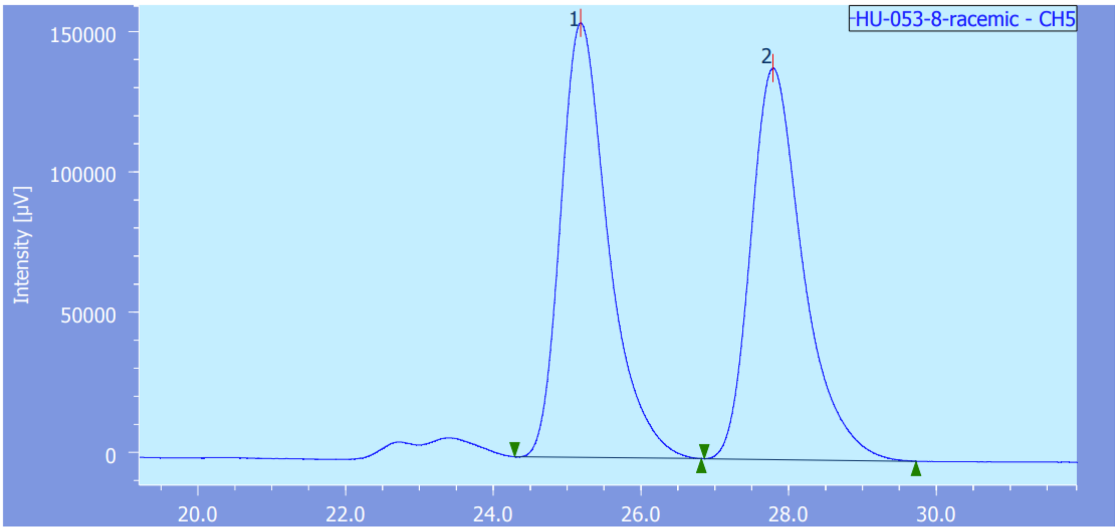

Decision

#	Peak Name	CH	tR [min]	Area [µV·sec]	Height [µV]	Area%	Height%	Quantity	NTP	Resolution	Symmetry Factor	Warning
1	Unknown	5	25.183	6951642	154757	50.264	52.599	N/A	7918	2.198	1.395	
2	Unknown	5	27.783	6878572	139463	49.736	47.401	N/A	8030	N/A	1.390	

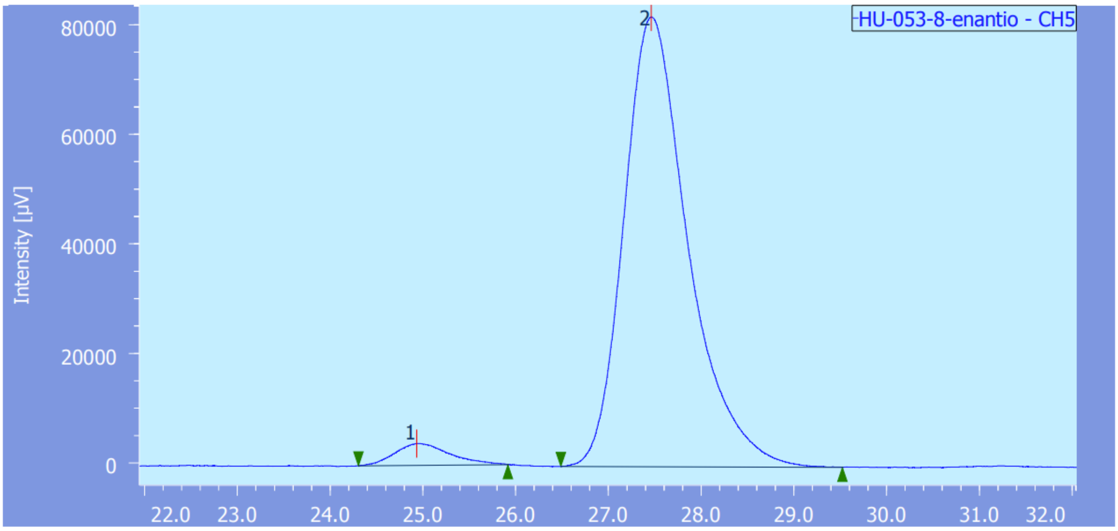

Decision

#	Peak Name	CH	tR [min]	Area [µV·sec]	Height [µV]	Area%	Height%	Quantity	NTP	Resolution	Symmetry Factor	Warning
1	Unknown	5	24.933	165715	3961	4.030	4.605	N/A	8401	2.208	1.329	
2	Unknown	5	27.460	3946464	82050	95.970	95.395	N/A	8289	N/A	1.367	

(*S*, *E*)-4-Methyl-*N*-(4-phenyl-2-(*p*-tolyl)but-3-en-1-yl)benzenesulfonamide (2)

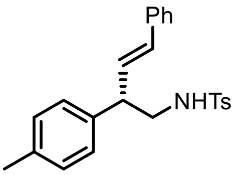

Prepared by **GP-A**. White solid, 53.3 mg, 68% yield. m.p. = 93-94 °C; ^{1}H NMR (400 MHz, $CDCl_3$) δ 7.65 – 7.57 (m, 2H), 7.24 – 7.09 (m, 7H), 7.05 – 6.98 (m, 2H), 6.97 – 6.90 (m, 2H), 6.27 (dd, *J* = 15.9, 0.9 Hz, 1H), 6.08 (dd, *J* = 15.9, 7.8 Hz, 1H), 4.42 (t, *J* = 5.8 Hz, 1H), 3.45 (q, *J* = 7.6 Hz, 1H), 3.27 – 3.12 (m, 2H), 2.34 (s, 3H), 2.23 (s, 3H); ^{13}C NMR (101 MHz, $CDCl_3$) δ 143.4, 137.1, 136.9, 136.8, 136.6, 132.0, 129.7, 129.6, 129.5, 128.5, 127.6, 127.5, 127.1, 126.2, 48.2, 47.5, 21.5, 21.0; IR (film): ν (cm^{-1}) 3266, 3024, 2922, 1597, 1514, 1495, 1425, 1318, 1291, 1150, 1093, 1075, 960, 815, 743, 692, 663, 551, 539; HR-MS (ESI) m/z calcd for $C_{24}H_{26}NO_2S$

[M+H$^+$] 392.1679, found 392.1677; $[\alpha]_D^{24.3}$ = -19.3 (c = 0.1, $CHCl_3$); HPLC conditions: AD-H column, hexane/2-propanol = 90/10, flow rate = 1.0 mL/min, λ = 254 nm, t_R = 24.3 min (major), t_R = 26.7 min (minor), 96:4 er.

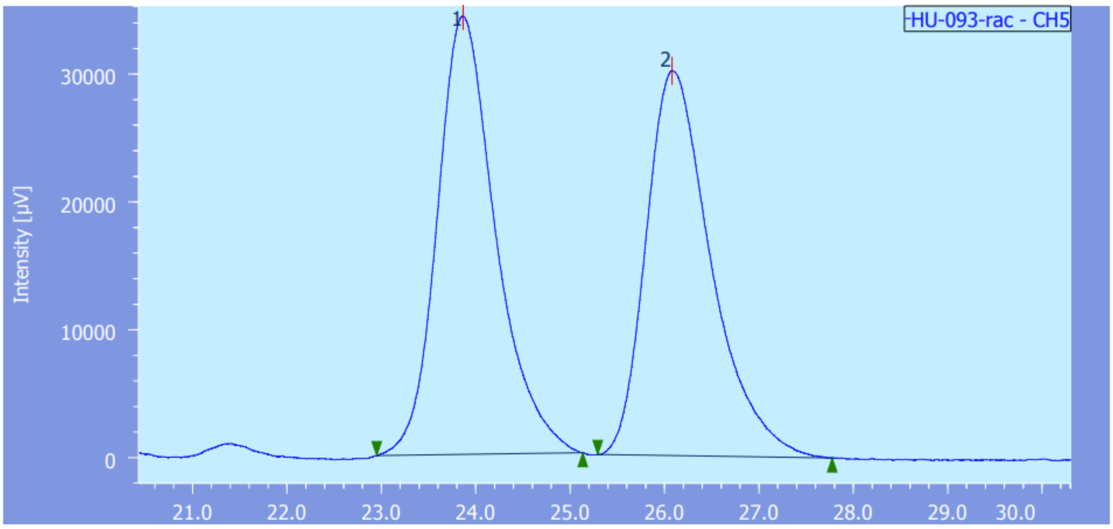

Decision

#	Peak Name	CH	tR [min]	Area [μV·sec]	Height [μV]	Area%	Height%	Quantity	NTP	Resolution	Symmetry Factor	Warning
1	Unknown	5	23.863	1475933	34266	50.629	53.267	N/A	7743	1.927	1.253	
2	Unknown	5	26.077	1439281	30064	49.371	46.733	N/A	7321	N/A	1.447	

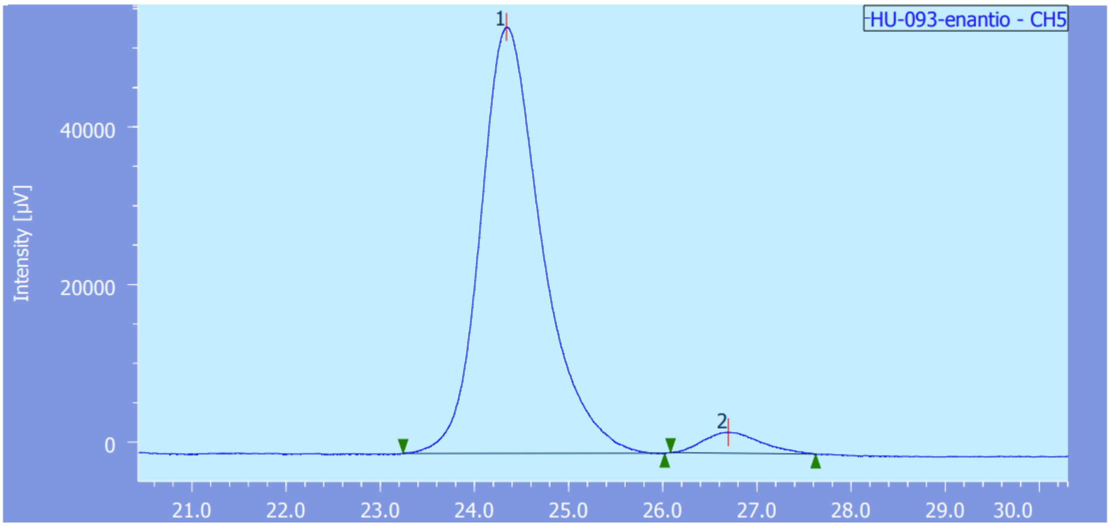

Decision

#	Peak Name	CH	tR [min]	Area [μV·sec]	Height [μV]	Area%	Height%	Quantity	NTP	Resolution	Symmetry Factor	Warning
1	Unknown	5	24.340	2450107	54048	95.564	95.329	N/A	7441	2.069	1.291	
2	Unknown	5	26.697	113730	2648	4.436	4.671	N/A	8544	N/A	1.268	

(*S*, 2*E*)-*N*-(2-(4-(*tert*-Butyl)phenyl)-4-phenylbut-3-en-1-yl)-4-methylbenzenesulfonamide (3)

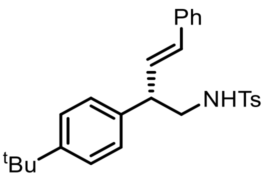

Prepared by **GP-A**. White solid, 56.3 mg, 65% yield. m.p. = 136-138 °C; ^{1}H NMR (400 MHz, $CDCl_3$) δ 7. 68 – 7.58 (m, 2H), 7.29 – 7.10 (m, 9H), 7.04 – 6.94 (m, 2H), 6.35 – 6.24 (m, 1H), 6.09 (dd, *J* = 15.9, 8.0 Hz, 1H), 4.34 (t, *J* = 6.1 Hz, 1H), 3.46 (q, *J* = 7.6 Hz, 1H), 3.31 – 3.15 (m, 2H), 2.36 (s, 3H), 1.23 (s, 9H); ^{13}C NMR (101 MHz, $CDCl_3$) δ 150.2, 143.4, 137.0, 136.9, 136.6, 132.0, 129.7, 129.4, 128.5, 127.6, 127.3, 127.1, 126.3, 125.9, 48.2, 47.4, 34.4, 31.3, 21.5; IR (film): ν (cm^{-1}) 3271, 3023, 2956, 1597, 1420, 1320, 1289, 1154, 1094, 1076, 960,

817, 751, 667, 556; HR-MS (ESI) m/z calcd for $C_{27}H_{32}NO_2S$ [M+H$^+$] 434.2148, found 434.2150; $[\alpha]_D^{24.3}$= -10.4 (c = 0.1, $CHCl_3$); HPLC conditions: OD-H column, hexane/2-propanol = 90/10, flow rate = 1.0 mL/min, λ = 254 nm, t_R = 11.8 min (minor), t_R = 15.4 min (major), 95:5 er.

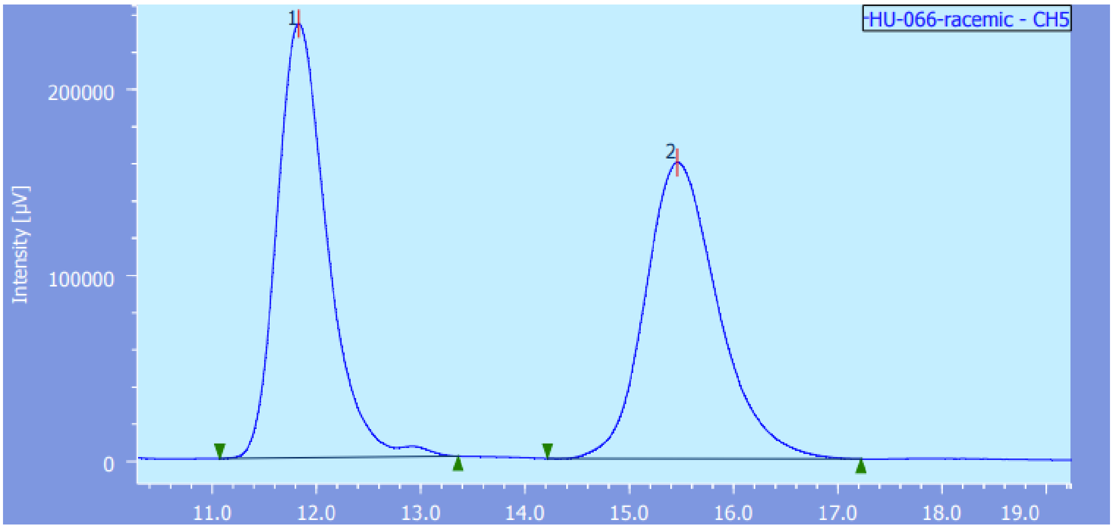

Decision

#	Peak Name	CH	tR [min]	Area [µV·sec]	Height [µV]	Area%	Height%	Quantity	NTP	Resolution	Symmetry Factor	Warning
1	Unknown	5	11.830	7825359	233330	50.198	59.439	N/A	3113	3.480	1.308	
2	Unknown	5	15.460	7763612	159223	49.802	40.561	N/A	2472	N/A	1.221	

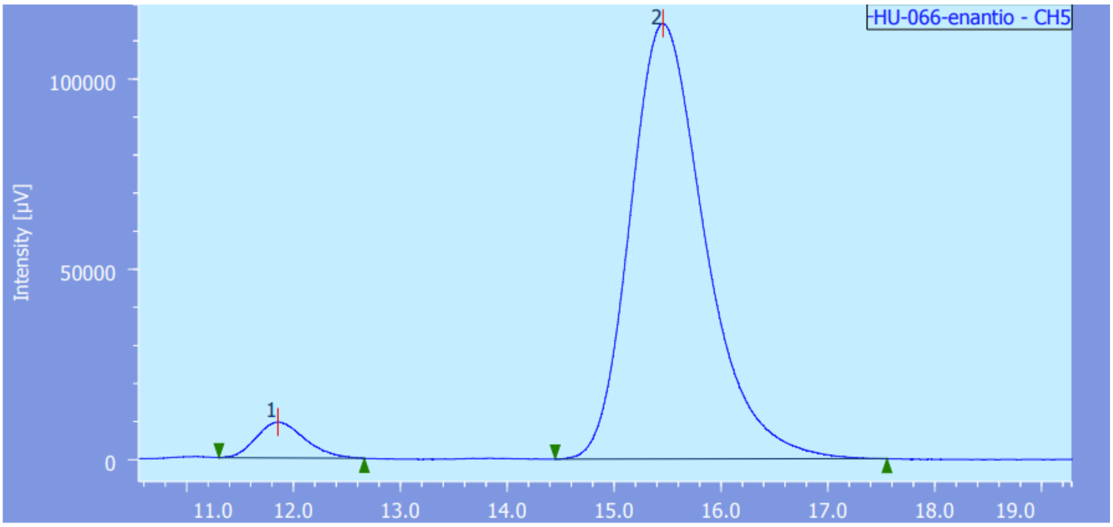

Decision

#	Peak Name	CH	tR [min]	Area [µV·sec]	Height [µV]	Area%	Height%	Quantity	NTP	Resolution	Symmetry Factor	Warning
1	Unknown	5	11.853	299495	9402	5.110	7.598	N/A	3215	3.475	1.230	
2	Unknown	5	15.457	5561537	114333	94.890	92.402	N/A	2473	N/A	1.315	

(*S*, *E*)-4-(1-((4-Methylphenyl)sulfonamido)-4-phenylbut-3-en-2-yl)phenyl acetate (4)

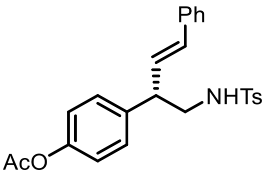

Prepared by **GP-A**. White solid, 59 mg, 68% yield. m.p. = 72-73 °C; ^{1}H NMR (400 MHz, $CDCl_3$) δ 7.69 – 7.58 (m, 2H), 7.26 – 7.12 (m, 7H), 7.11 – 7.03 (m, 2H), 6.98 – 6.91 (m, 2H), 6.31 (dd, *J* = 15.9, 0.9 Hz, 1H), 6.07 (dd, *J* = 15.9, 7.9 Hz, 1H), 4.43 (t, *J* = 6.3 Hz, 1H), 3.51 (q, *J* = 7.5 Hz, 1H), 3.28 – 3.14 (m, 2H), 2.35 (s, 3H), 2.21 (s, 3H); ^{13}C NMR (101 MHz, $CDCl_3$) δ 169.4, 149.7, 143.5, 137.8, 136.8, 136.4, 132.4, 129.8, 128.9, 128.6, 128.5, 127.8, 127.1, 126.3, 122.0, 48.1, 47.5, 21.5, 21.1; IR (film): ν (cm^{-1}) 3273, 2922, 2326, 2335, 1755, 1597, 1507, 1368, 1328, 1198, 1153, 1092, 1013, 972, 909, 845, 810, 744, 694, 664, 562, 552, 543, 535;

HR-MS (ESI) m/z calcd for $C_{25}H_{26}NO_4S$ $[M+H^+]$ 436.1577, found 436.1574; $[\alpha]_D^{24.2}$ = -19.2 (c = 0.1, $CHCl_3$); HPLC conditions: OD-H column, hexane/2-propanol = 90/10, flow rate = 1.0 mL/min, λ = 254 nm, t_R = 30.8 min (major), t_R = 35.8 min (minor), 96:4 er.

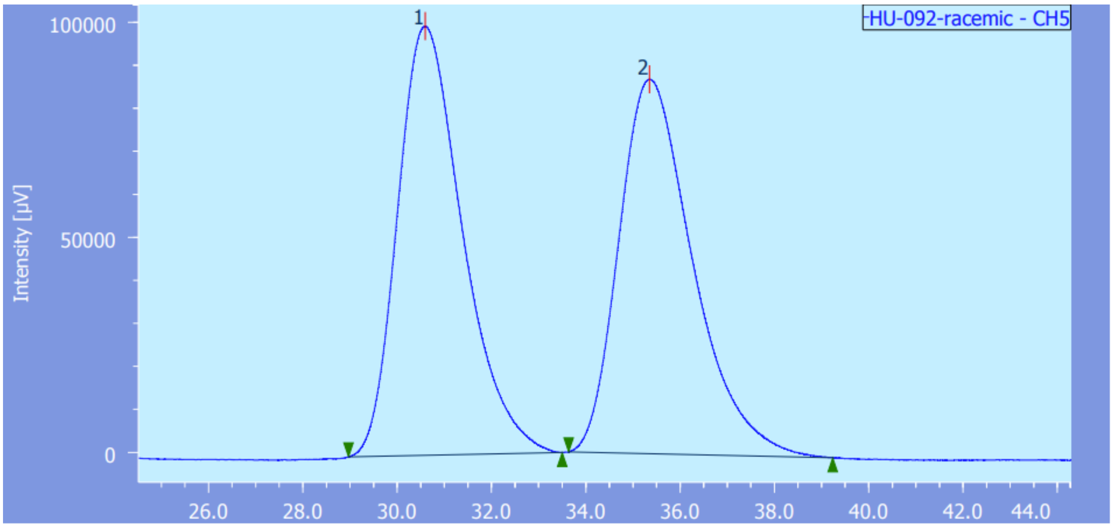

Decision

#	Peak Name	CH	tR [min]	Area [µV·sec]	Height [µV]	Area%	Height%	Quantity	NTP	Resolution	Symmetry Factor	Warning
1	Unknown	5	30.590	9547589	99701	50.191	53.401	N/A	2415	1.798	1.344	
2	Unknown	5	35.347	9475024	87001	49.809	46.599	N/A	2521	N/A	1.401	

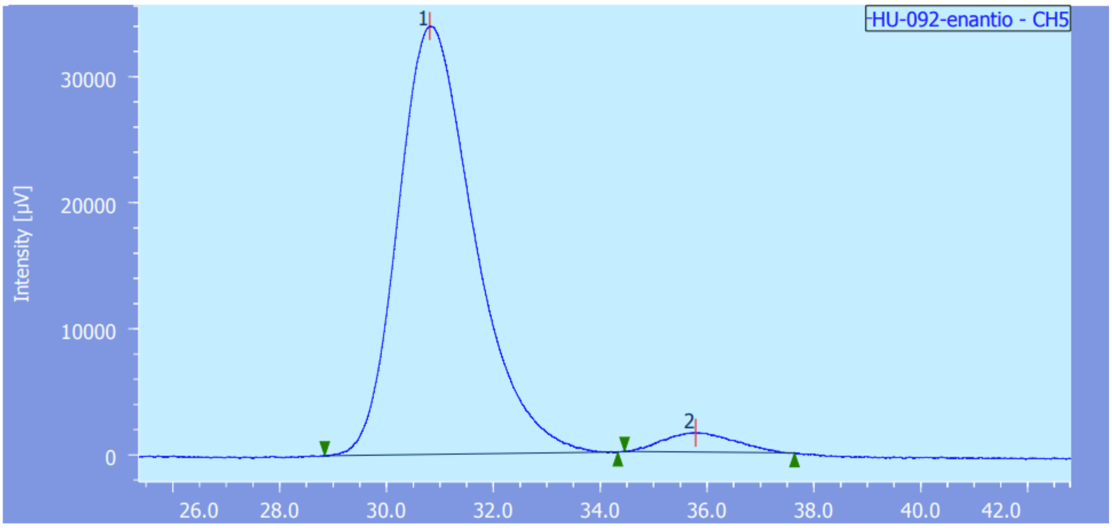

Decision

#	Peak Name	CH	tR [min]	Area [µV·sec]	Height [µV]	Area%	Height%	Quantity	NTP	Resolution	Symmetry Factor	Warning
1	Unknown	5	30.807	3366183	33964	95.926	95.671	N/A	2331	1.905	1.339	
2	Unknown	5	35.783	142973	1537	4.074	4.329	N/A	2836	N/A	1.164	

(*S*, *E*)-*N*-(2-(4-Fluorophenyl)-4-phenylbut-3-en-1-yl)-4-methylbenzenesulfonamide (5)

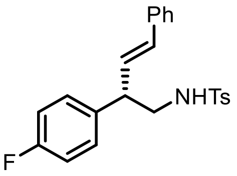

Prepared by **GP-B**. White solid, 51.5 mg, 65% yield. m.p. = 146-147 °C; ^{1}H NMR (400 MHz, $CDCl_3$) δ 7.68 – 7.56 (m, 2H), 7.26 – 7.11 (m, 7H), 7.06 – 6.97 (m, 2H), 6.94 – 6.84 (m, 2H), 6.28 (dd, J = 15.9, 0.7 Hz, 1H), 6.06 (dd, J = 15.9, 7.8 Hz, 1H), 4.48 (t, J = 6.2 Hz, 1H), 3.50 (q, J = 7.5 Hz, 1H), 3.32 – 3.11 (m, 2H), 2.35 (s, 3H); ^{13}C NMR (101 MHz, $CDCl_3$) δ 161.9 (d, J = 246.8), 143.5, 136.8, 136.4, 136.0 (d, J = 3.3), 132.4, 129.7, 129.2 (d, J = 8.0), 129.0, 128.5, 127.8, 127.1, 126.3, 115. 7 (d, J = 21.3), 47.9, 47.5, 21.5; ^{19}F NMR (377 MHz, $CDCl_3$) δ -115.18; IR (film): ν (cm^{-1}) 3290, 3266, 3029, 2929, 1598, 1509, 1496, 1422, 1321, 1308, 1222, 1152, 1094,

1081, 964, 847, 828, 819, 753, 692, 669, 550, 533; HR-MS (ESI) m/z calcd for $C_{23}H_{23}FNO_2S$ [M+H$^+$] 396.1428, found 396.1425; $[\alpha]_D^{24.2}$ = -23.2 (c = 0.1, $CHCl_3$); HPLC conditions: OD-H column, hexane/2-propanol = 95/5, flow rate = 0.5 mL/min, λ = 254 nm, t_R = 63.4 min (major), t_R = 78.4 min (minor), 95:5 er.

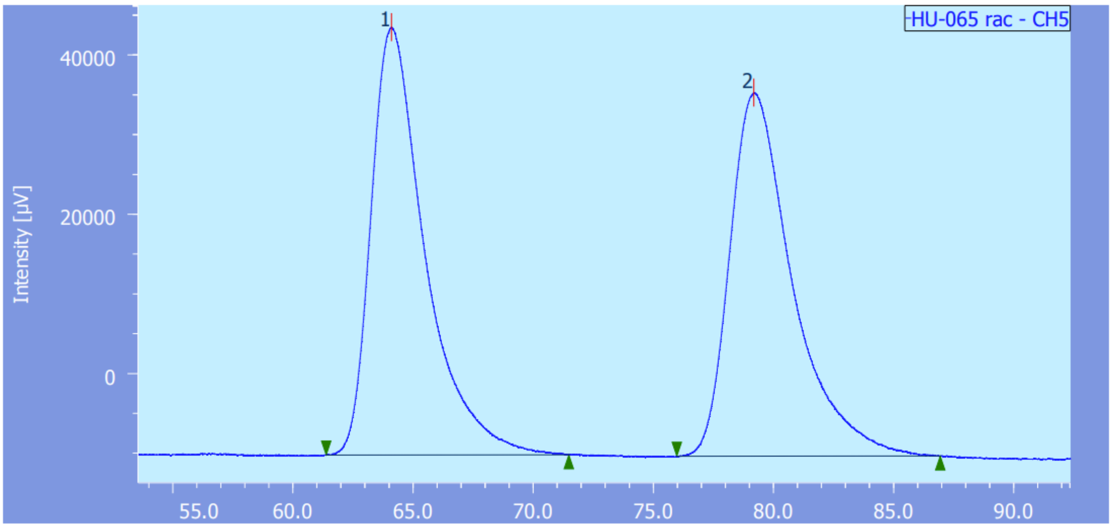

Decision

#	Peak Name	CH	tR [min]	Area [µV·sec]	Height [µV]	Area%	Height%	Quantity	NTP	Resolution	Symmetry Factor	Warning
1	Unknown	5	64.097	8369984	53672	50.342	54.061	N/A	4489	3.646	1.672	
2	Unknown	5	79.177	8256394	45608	49.658	45.939	N/A	5028	N/A	1.602	

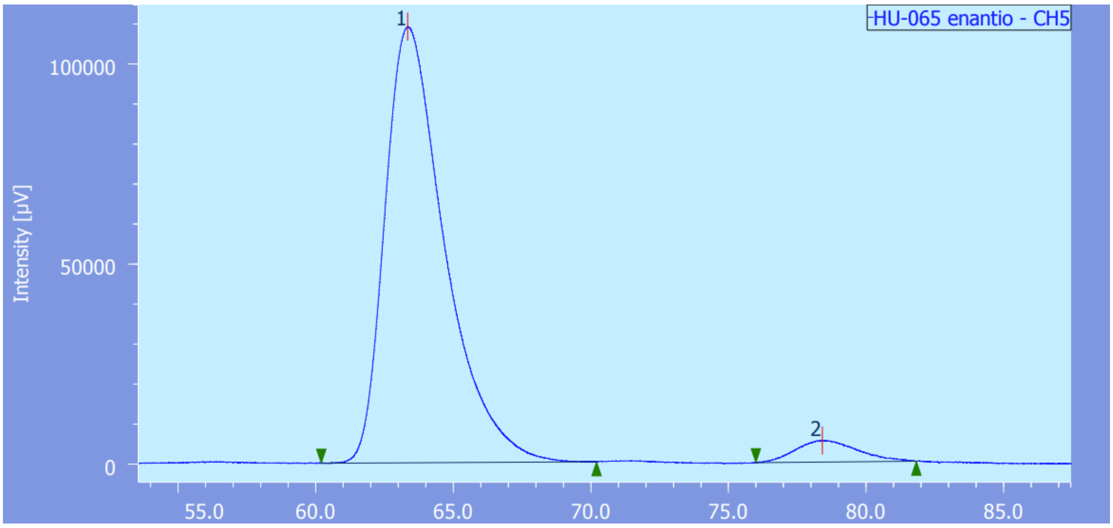

Decision

#	Peak Name	CH	tR [min]	Area [µV·sec]	Height [µV]	Area%	Height%	Quantity	NTP	Resolution	Symmetry Factor	Warning
1	Unknown	5	63.340	16464279	108917	95.109	95.275	N/A	4398	3.780	1.584	
2	Unknown	5	78.407	846595	5402	4.891	4.725	N/A	5647	N/A	1.255	

(*S*, *E*)-*N*-(2-(4-Chlorophenyl)-4-phenylbut-3-en-1-yl)-4-methylbenzenesulfonamide (6)

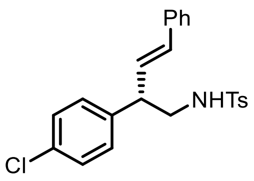

Prepared by **GP-A**. White solid, 51.9 mg, 63% yield. m.p. = 139-140 °C; ^{1}H NMR (400 MHz, $CDCl_3$) δ 7.64 – 7.55 (m, 2H), 7.25 – 7.09 (m, 9H), 7.02 – 6.94 (m, 2H), 6.28 (dd, *J* = 15.9, 1.0 Hz, 1H), 6.05 (dd, *J* = 15.9, 7.8 Hz, 1H), 4.56 (t, *J* = 6.2 Hz, 1H), 3.49 (q, *J* = 7.5 Hz, 1H), 3.27 – 3.12 (m, 2H), 2.34 (s, 3H); ^{13}C NMR (101 MHz, $CDCl_3$) δ 143.5, 138.8, 136.8, 136.3, 133.0, 132.5, 129.7, 129.0, 129.0, 128.6, 128.5, 127.8, 127.0, 126.3, 48.1, 47.4, 21.5; IR (film): ν (cm^{-1}) 3289, 3262, 3027, 2875, 1597, 1492, 1447, 1425, 1319, 1306, 1289, 1151, 1087,

1013, 964, 819, 746, 694, 670, 662, 557, 546, 532; HR-MS (ESI) m/z calcd for $C_{23}H_{23}ClNO_2S$ [M+H$^+$] 412.1132, found 412.1136; $[\alpha]_D$ $^{24.1}$ = -19.1 (c = 0.1, $CHCl_3$); HPLC conditions: OD-H column, hexane/2-propanol = 95/5, flow rate = 0.5 mL/min, λ = 254 nm, t_R = 69.1 min (major), t_R = 86.6 min (minor), 94:6 er.

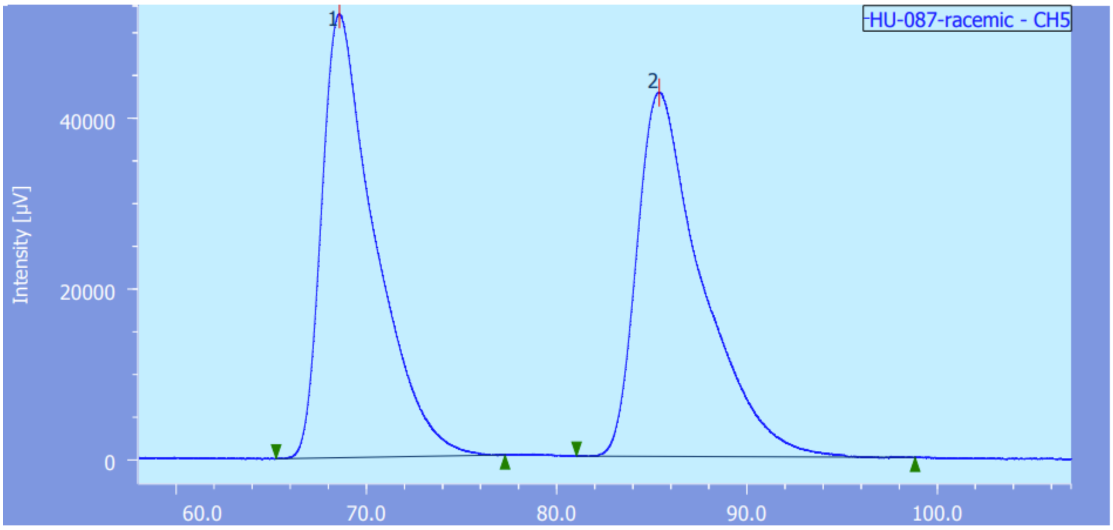

Decision

#	Peak Name	CH	tR [min]	Area [μV·sec]	Height [μV]	Area%	Height%	Quantity	NTP	Resolution	Symmetry Factor	Warning
1	Unknown	5	68.580	10082677	51844	49.878	54.907	N/A	3174	3.162	1.817	
2	Unknown	5	85.380	10132132	42577	50.122	45.093	N/A	3486	N/A	1.796	

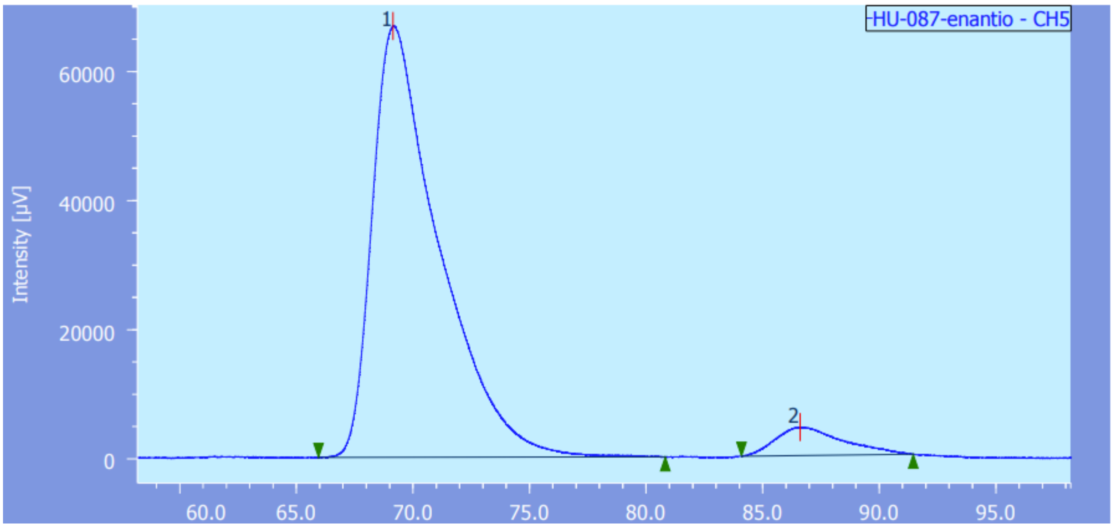

Decision

#	Peak Name	CH	tR [min]	Area [μV·sec]	Height [μV]	Area%	Height%	Quantity	NTP	Resolution	Symmetry Factor	Warning
1	Unknown	5	69.143	13302766	66773	93.808	93.821	N/A	3129	3.445	1.939	
2	Unknown	5	86.597	878007	4398	6.192	6.179	N/A	4409	N/A	1.488	

(*S*, *E*)-*N*-(2-(4-Bromophenyl)-4-phenylbut-3-en-1-yl)-4-methylbenzenesulfonamide (7)

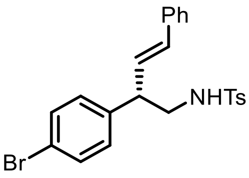

Prepared by **GP-A**. White solid, 59.3 mg, 65% yield. m.p. = 132-133 °C; ^{1}H NMR (400 MHz, $CDCl_3$) δ 7.66 – 7.54 (m, 2H), 7.36 – 7.28 (m, 2H), 7.22 – 7.10 (m, 7H), 6.98 – 6.88 (m, 2H), 6.28 (dd, *J* = 15.9, 1.0 Hz, 1H), 6.05 (dd, *J* = 15.9, 7.8 Hz, 1H), 4.52 (t, *J* = 6.2 Hz, 1H), 3.48 (q, *J* = 7.5 Hz, 1H), 3.29 – 3.12 (m, 2H), 2.35 (s, 3H); ^{13}C NMR (101 MHz, $CDCl_3$) δ 143.5, 139.3, 136.8, 136.3, 132.6, 132.0, 129.7, 129.4, 128.6, 127.8, 127.0, 126.3, 121.1, 48.2, 47.3, 21.5; IR (film): ν (cm^{-1}) 3285, 3025, 2922, 1595, 1488, 1446, 1425, 1406, 1319, 1306, 1152,

1090, 1072, 1010, 964, 817, 745, 691, 667, 553, 532, 491; HR-MS (ESI) m/z calcd for $C_{23}H_{23}BrNO_2S$ [M+H$^+$] 456.0627, found 456.0627; $[\alpha]_D^{24.3}$ = -20.6 (c = 0.1, $CHCl_3$); HPLC conditions: OD-H column, hexane/2-propanol = 92/8, flow rate = 0.5 mL/min, λ = 254 nm, t_R = 45.6 min (major), t_R = 54.7 min (minor), 93:7 er.

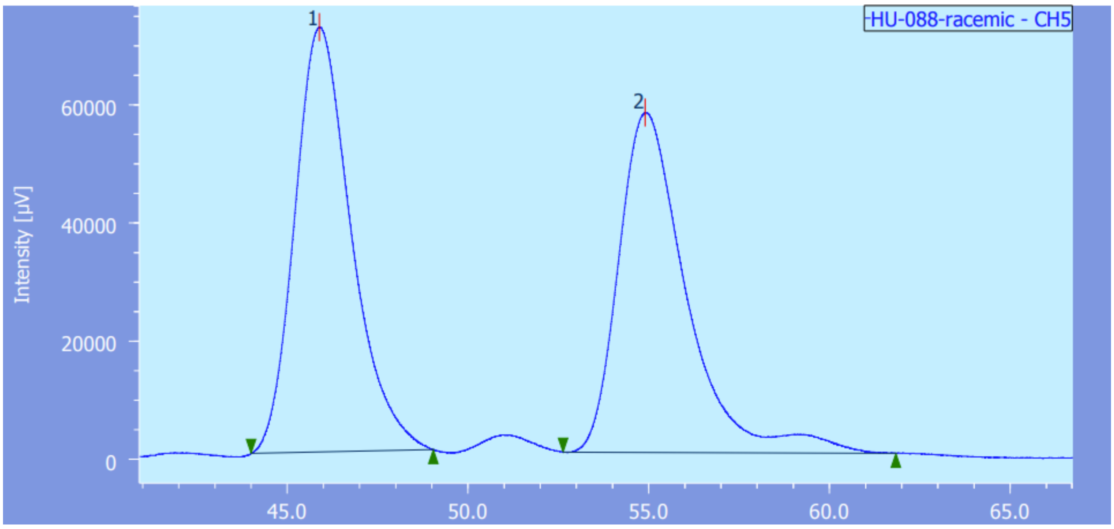

Decision

#	Peak Name	CH	tR [min]	Area [µV·sec]	Height [µV]	Area%	Height%	Quantity	NTP	Resolution	Symmetry Factor	Warning
1	Unknown	5	45.887	7822647	71895	50.883	55.551	N/A	4299	3.014	1.289	
2	Unknown	5	54.900	7551197	57526	49.117	44.449	N/A	4717	N/A	1.983	

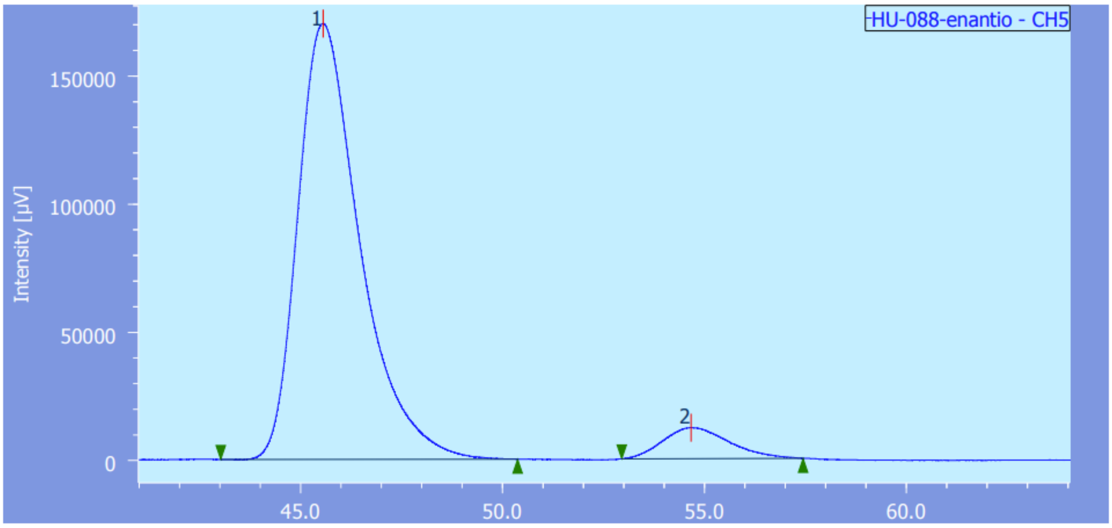

Decision

#	Peak Name	CH	tR [min]	Area [µV·sec]	Height [µV]	Area%	Height%	Quantity	NTP	Resolution	Symmetry Factor	Warning
1	Unknown	5	45.553	18157738	169861	92.915	93.358	N/A	4567	3.190	1.483	
2	Unknown	5	54.660	1384568	12085	7.085	6.642	N/A	5210	N/A	1.278	

(*S*, *E*)-4-Methyl-*N*-(4-phenyl-2-(4-(trifluoromethyl)phenyl)but-3-en-1-yl)benzenesulfonamide (8)

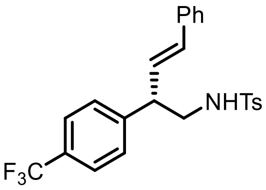

Prepared by **GP-A**. White solid, 53.4 mg, 60% yield. m.p. = 106-107 °C; ^{1}H NMR (400 MHz, $CDCl_3$) δ 7.66 – 7.56 (m, 2H), 7.50 – 7.42 (m, 2H), 7.24 – 7.12 (m, 9H), 6.32 (dd, *J* = 15.9, 1.0 Hz, 1H), 6.07 (dd, *J* = 15.9, 8.0 Hz, 1H), 4.58 (t, *J* = 6.3 Hz, 1H), 3.59 (q, *J* = 7.6 Hz, 1H), 3.36 – 3.14 (m, 2H), 2.34 (s, 3H); ^{13}C NMR (101 MHz, $CDCl_3$) δ 144.5, 143.6, 136.7, 136.2, 133.0, 129.8, 129.5 (q, *J* = 32.6 Hz), 128.6, 128.2, 128.1, 127.9, 127.0, 126.3, 125.8 (q, *J* =

3.7 Hz), 124.0 (d, J = 273.0 Hz), 48.6, 47.3, 21.5; ^{19}F NMR (377 MHz, $CDCl_3$) δ -62.51; IR (film): ν (cm^{-1}) 3266, 3026, 2927, 1617, 1598, 1495, 1447, 1420, 1321, 1157, 1117, 1066, 1017, 964, 848, 836, 814, 751, 738, 691, 665, 597, 554, 531; HR-MS (ESI) m/z calcd for $C_{24}H_{23}F_3NO_2S$ [M+H$^+$] 446.1396, found 446.1399; $[\alpha]_D{}^{24.3}$ = -20.8 (c = 0.1, $CHCl_3$); HPLC conditions: OD-H column, hexane/2-propanol = 90/10, flow rate = 1.0 mL/min, λ = 254 nm, t_R = 16.6 min (major), t_R = 20.7 min (minor), 90:10 er.

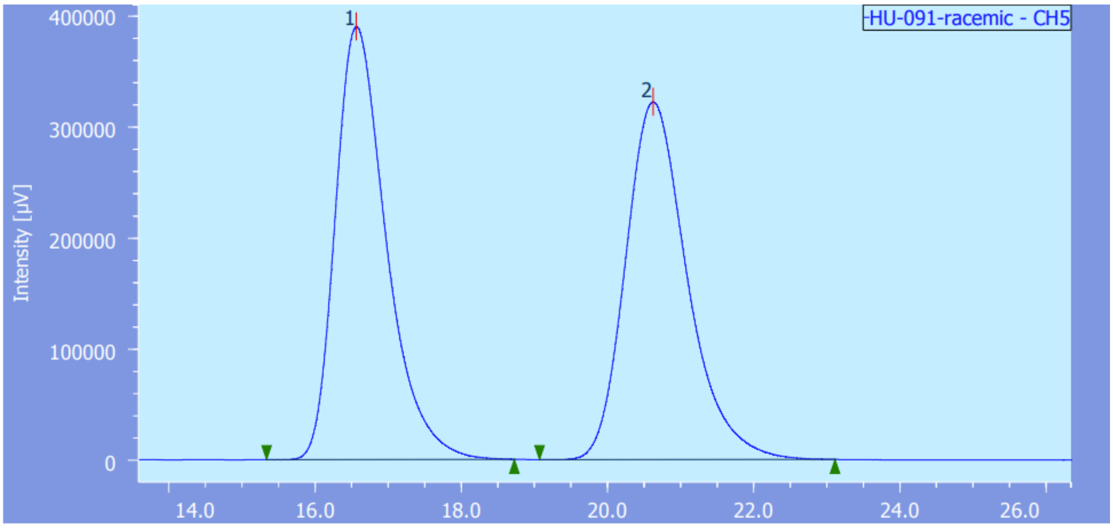

Decision

#	Peak Name	CH	tR [min]	Area [µV·sec]	Height [µV]	Area%	Height%	Quantity	NTP	Resolution	Symmetry Factor	Warning
1	Unknown	5	16.563	18865664	389923	49.958	54.753	N/A	2870	2.973	1.412	
2	Unknown	5	20.623	18897190	322224	50.042	45.247	N/A	3017	N/A	1.277	

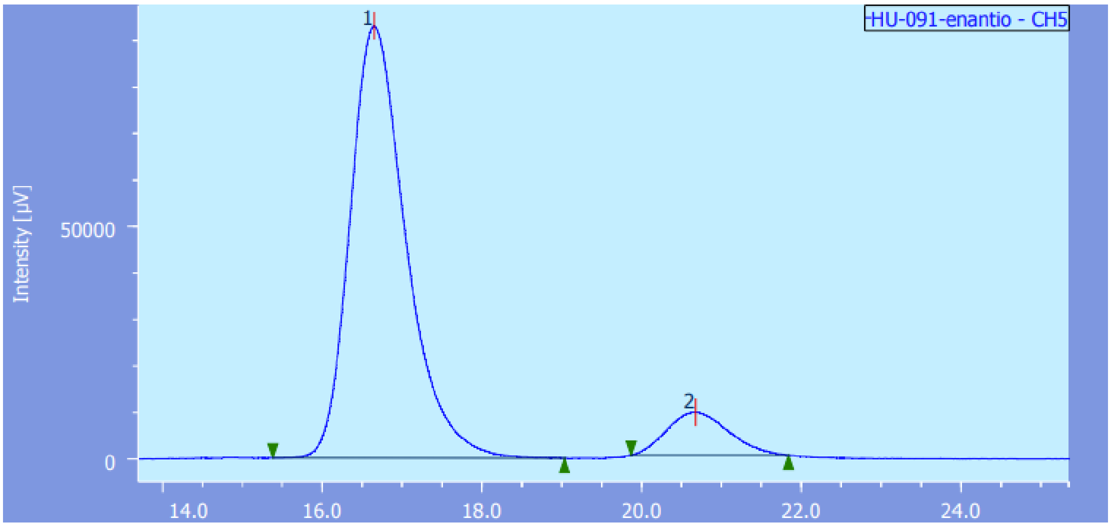

Decision

#	Peak Name	CH	tR [min]	Area [µV·sec]	Height [µV]	Area%	Height%	Quantity	NTP	Resolution	Symmetry Factor	Warning
1	Unknown	5	16.650	4528608	92930	90.042	90.916	N/A	2896	2.997	1.337	
2	Unknown	5	20.673	500843	9285	9.958	9.084	N/A	3231	N/A	1.180	

Methyl (*S*, *E*)-4-(1-((4-methylphenyl)sulfonamido)-4-phenylbut-3-en-2-yl)benzoate (9)

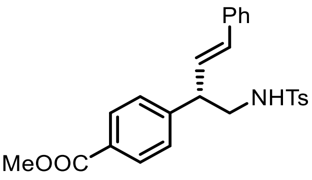

Prepared by **GP-A**. White solid, 65.6 mg, 75% yield. m.p. = 133-134 °C; ^{1}H NMR (400 MHz, $CDCl_3$) δ 7.93 – 7.83 (m, 2H), 7.66 – 7.56 (m, 2H), 7.26 – 7.09 (m, 9H), 6.31 (dd, J = 15.9, 0.8 Hz, 1H), 6.08 (dd, J = 15.9, 7.9 Hz, 1H), 4.55 (t, J = 6.3 Hz, 1H), 3.83 (s, 3H), 3.58 (q, J = 7.6 Hz, 1H), 3.32 – 3.18 (m, 2H), 2.34 (s, 3H); ^{13}C NMR (101

MHz, $CDCl_3$) δ 166.7, 145.6, 143.6, 136.8, 136.3, 132.9, 130.2, 129.7, 129.1, 128.6, 128.3, 127.8, 127.7, 127.0, 126.3, 52.1, 48.8, 47.3, 21.5; IR (film): ν (cm^{-1}) 3232, 2952, 2846, 1718, 1608, 1598, 1495, 1447, 1433, 1322, 1275, 1184, 1154, 1108, 1090, 1074, 1018, 977, 964, 910, 815, 776, 748, 706, 691, 672, 557, 544, 534, 491; HR-MS (ESI) m/z calcd for $C_{25}H_{26}NO_4S$ [M+H$^+$] 436.1577, found 436.1579; $[\alpha]_D^{23.9}$ = -19.6 (c = 0.1, $CHCl_3$); HPLC conditions: OD-H column, hexane/2-propanol = 90/10, flow rate = 1.0 mL/min, λ = 254 nm, t_R = 41.1 min (major), t_R = 49.2 min (minor), 94:6 er.

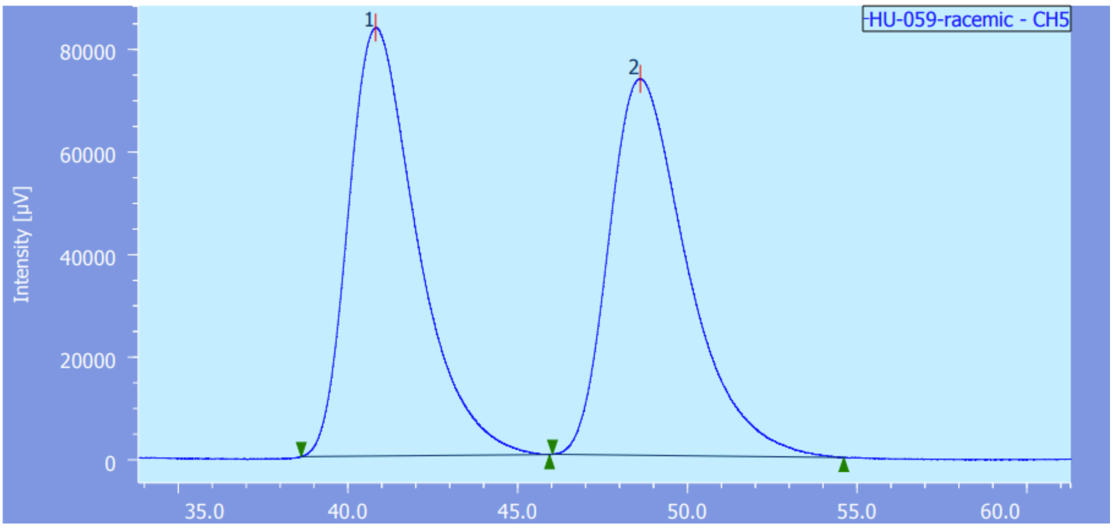

Decision

#	Peak Name	CH	tR [min]	Area [µV·sec]	Height [µV]	Area%	Height%	Quantity	NTP	Resolution	Symmetry Factor	Warning
1	Unknown	5	40.813	11752247	83481	49.934	53.223	N/A	2054	2.027	1.499	
2	Unknown	5	48.607	11783540	73370	50.066	46.777	N/A	2240	N/A	1.484	

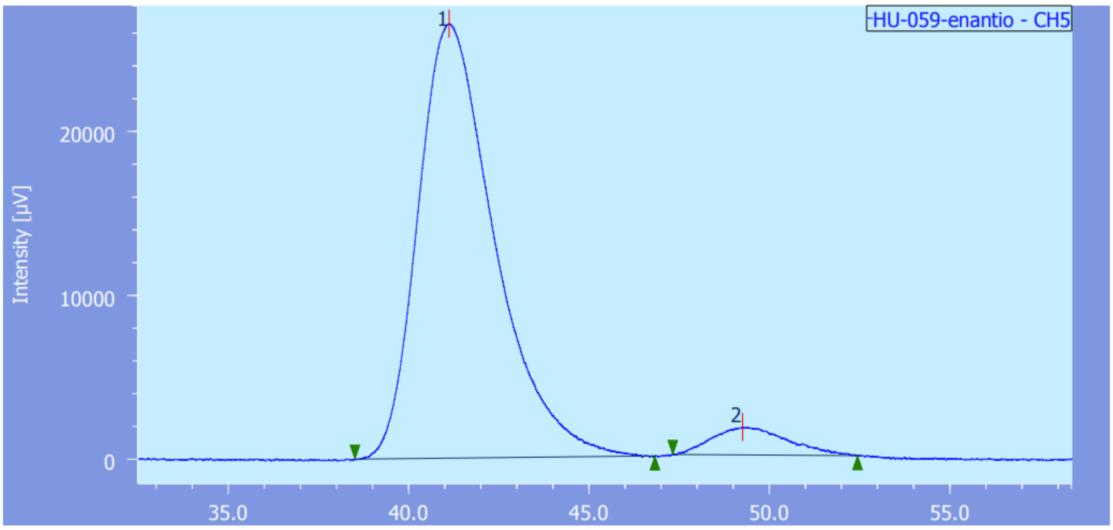

Decision

#	Peak Name	CH	tR [min]	Area [µV·sec]	Height [µV]	Area%	Height%	Quantity	NTP	Resolution	Symmetry Factor	Warning
1	Unknown	5	41.117	3907568	26494	94.070	93.968	N/A	1923	2.116	1.452	
2	Unknown	5	49.250	246317	1701	5.930	6.032	N/A	2479	N/A	1.335	

(*S*, *E*)-*N*-(2-(4-Cyanophenyl)-4-phenylbut-3-en-1-yl)-4-methylbenzenesulfonamide (10)

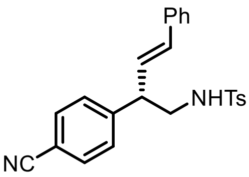

Prepared by **GP-A**. White solid, 48.3 mg, 60% yield. m.p. = 90-91 °C; ^{1}H NMR (400 MHz, $CDCl_3$) δ 7.65 – 7.58 (m, 2H), 7.52 – 7.45 (m, 2H), 7.25 – 7.14 (m, 9H), 6.32 (dd, *J* = 15.9, 0.7 Hz, 1H), 6.05 (dd, *J* = 15.9, 8.0 Hz, 1H), 4.63 (t, *J* = 6.4 Hz, 1H), 3.60 (q, *J* = 7.6 Hz, 1H), 3.30 – 3.17 (m, 2H),

2.36 (s, 3H); ^{13}C NMR (101 MHz, $CDCl_3$) δ 146.0, 143.7, 136.7, 136.0, 133.4, 132.6, 129.8, 128.62, 128.57, 128.1, 127.6, 127.0, 126.3, 118.6, 111.1, 48.9, 47.2, 21.5; IR (film): ν (cm^{-1}) 3276, 3028, 2924, 2227, 1606, 1598, 1504, 1494, 1448, 1416, 1325, 1306, 1291, 1155, 1092, 966, 831, 814, 753, 693, 662, 550; HR-MS (ESI) m/z calcd for $C_{24}H_{23}N_2O_2S$ [M+H$^+$] 403.1475, found 403.1472; $[\alpha]_D^{24.0}$ = -18.4 (c = 0.1, $CHCl_3$); HPLC conditions: OD-H column, hexane/2-propanol = 90/10, flow rate = 1.0 mL/min, λ = 254 nm, t_R = 48.2 min (major), t_R = 72.0 min (minor), 91:9 er.

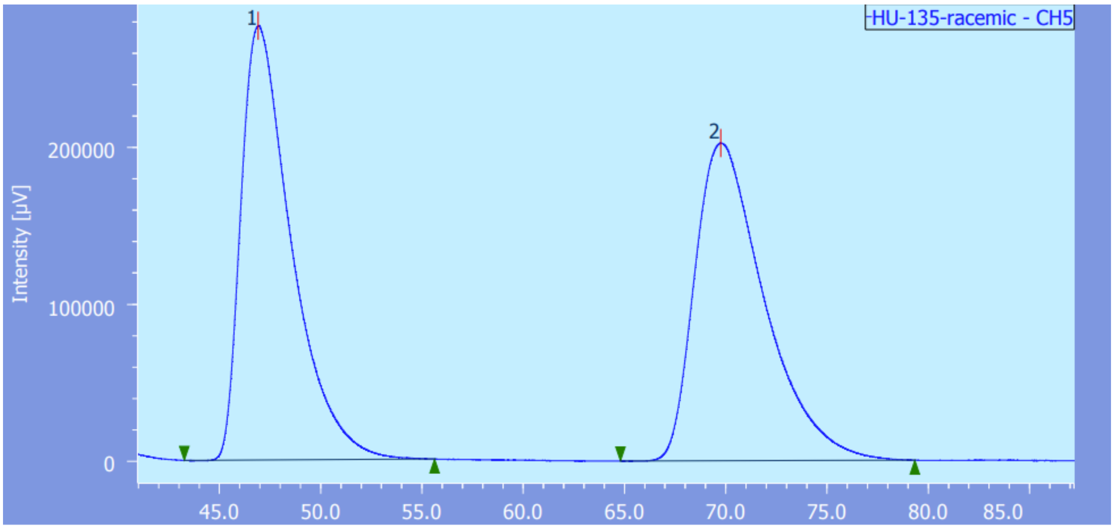

Decision

#	Peak Name	CH	tR [min]	Area [µV·sec]	Height [µV]	Area%	Height%	Quantity	NTP	Resolution	Symmetry Factor	Warning
1	Unknown	5	46.913	47911330	276454	49.766	57.742	N/A	1826	4.344	1.843	
2	Unknown	5	69.740	48362852	202321	50.234	42.258	N/A	2059	N/A	1.624	

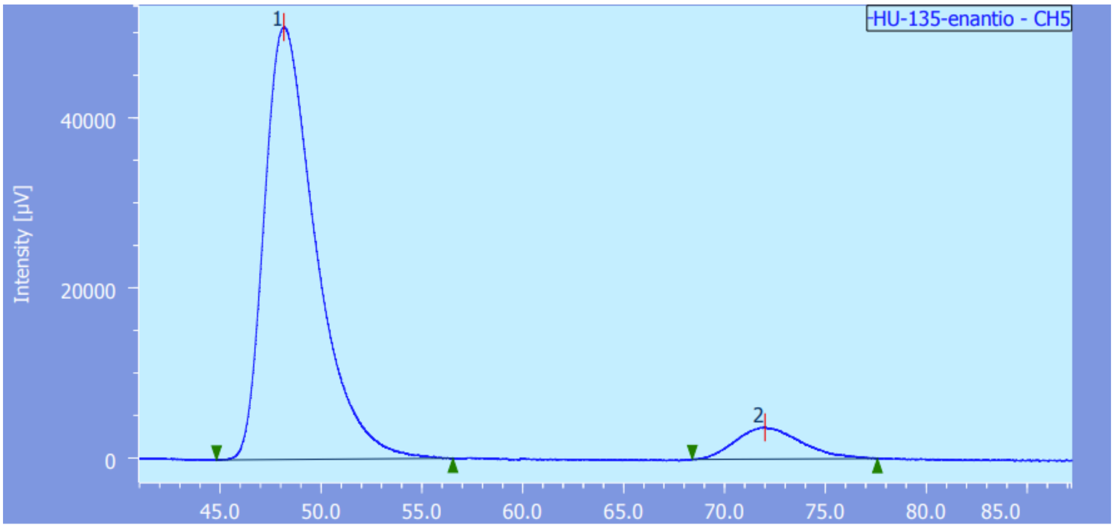

Decision

#	Peak Name	CH	tR [min]	Area [µV·sec]	Height [µV]	Area%	Height%	Quantity	NTP	Resolution	Symmetry Factor	Warning
1	Unknown	5	48.157	9017963	50801	91.478	93.170	N/A	1837	4.553	1.593	
2	Unknown	5	71.997	840133	3724	8.522	6.830	N/A	2300	N/A	1.289	

(*S, E*)-4-Methyl-*N*-(4-phenyl-2-(*o*-tolyl)but-3-en-1-yl)benzenesulfonamide (11)

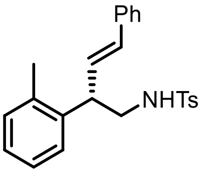

Prepared by **GP-A**. White solid, 43.9 mg, 56% yield. m.p. = 93-94 ºC; ^{1}H NMR (400 MHz, $CDCl_3$) δ 7.67 – 7.58 (m, 2H), 7.23 – 7.11 (m, 7H), 7.10 – 7.02 (m, 3H), 7.00 – 6.93 (m, 1H), 6.25 (dd, J = 15.9, 0.9 Hz, 1H), 6.04 (dd, J = 15.9, 7.7 Hz, 1H), 4.46 (t, J = 6.1 Hz, 1H), 3.77 (q, J = 7.4 Hz, 1H), 3.30 – 3.14 (m, 2H), 2.35 (s, 3H), 2.20 (s, 3H); ^{13}C NMR (101 MHz, $CDCl_3$) δ 143.4, 138.1,

136.9, 136.5, 132.1, 131.0, 129.7, 129.1, 128.5, 127.6, 127.1, 127.0, 126.5, 126.2, 125.9, 46.7, 44.2, 21.5, 19.4; IR (film): ν (cm^{-1}) 3297, 3026, 2920, 1646, 1598, 1494, 1447, 1422, 1318, 1289, 1150, 1092, 1076, 962, 817, 748, 691, 664, 551, 537; HR-MS (ESI) m/z calcd for $C_{24}H_{26}NO_2S$ [M+H$^+$] 392.1679, found 392.1682; $[\alpha]_D^{24.3}$ = -18.2 (c = 0.1, $CHCl_3$); HPLC conditions: AD-H column, hexane/2-propanol = 90/10, flow rate = 1.0 mL/min, λ = 254 nm, t_R = 19.1 min (minor), t_R = 26.0 min (major), 96:4 er.

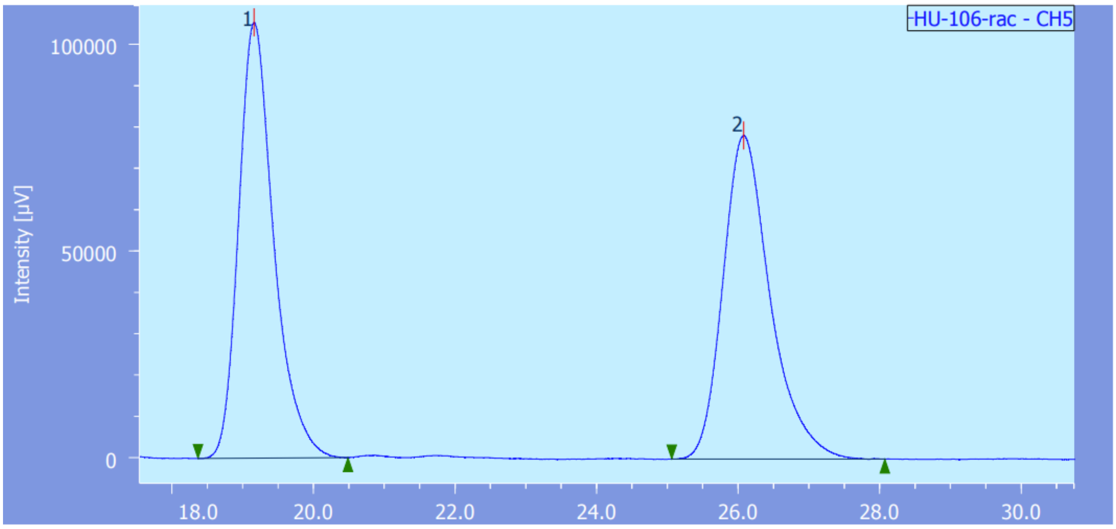

Decision

#	Peak Name	CH	tR [min]	Area [µV·sec]	Height [µV]	Area%	Height%	Quantity	NTP	Resolution	Symmetry Factor	Warning
1	Unknown	5	19.160	3636251	105340	49.954	57.366	N/A	7746	6.796	1.320	
2	Unknown	5	26.077	3642970	78288	50.046	42.634	N/A	7945	N/A	1.319	

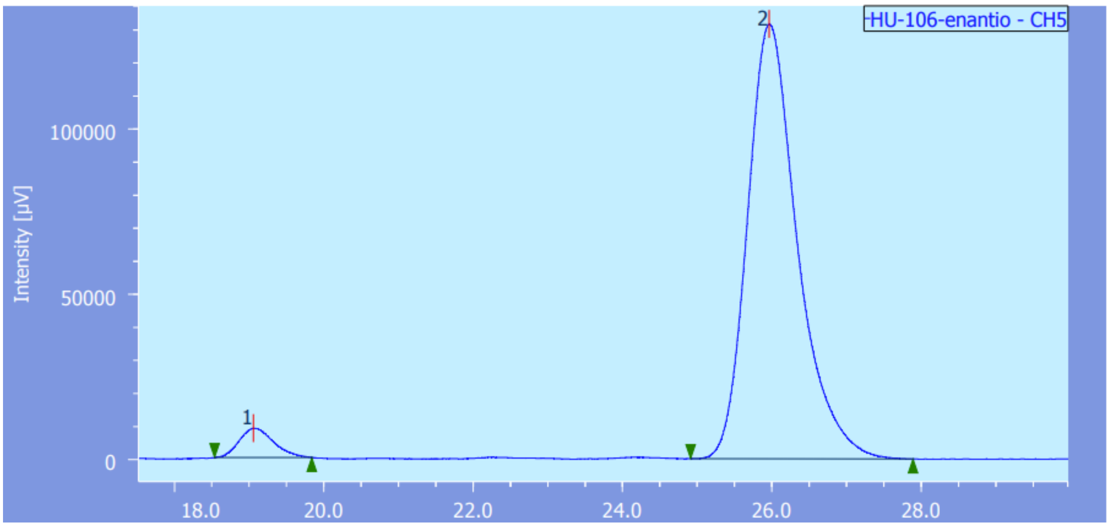

Decision

#	Peak Name	CH	tR [min]	Area [µV·sec]	Height [µV]	Area%	Height%	Quantity	NTP	Resolution	Symmetry Factor	Warning
1	Unknown	5	19.057	284800	8870	4.456	6.320	N/A	8180	6.886	1.272	
2	Unknown	5	25.963	6106308	131473	95.544	93.680	N/A	7897	N/A	1.319	

(*S*, *E*)-4-Methyl-*N*-(4-phenyl-2-(*m*-tolyl)but-3-en-1-yl)benzenesulfonamide (12)

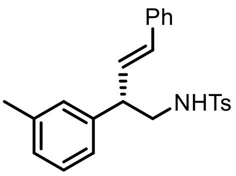

Prepared by **GP-A**. White solid, 59.6 mg, 76% yield. m.p. = 94-95 °C; ^{1}H NMR (400 MHz, $CDCl_3$) δ 7.67 – 7.56 (m, 2H), 7.24 – 7.06 (m, 8H), 7.01 – 6.92 (m, 1H), 6.88 – 6.79 (m, 2H), 6.29 (dd, *J* = 15.9, 0.7 Hz, 1H), 6.08 (dd, *J* = 15.9, 7.9 Hz, 1H), 4.39 (t, *J* = 6.1 Hz, 1H), 3.45 (q, *J* = 7.6 Hz, 1H), 3.29 – 3.15 (m, 2H), 2.34 (s, 3H), 2.23 (s, 3H); ^{13}C NMR (101 MHz, $CDCl_3$)

δ 143.4, 140.1, 138.6, 136.9, 136.6, 132.0, 129.7, 129.4, 128.8, 128.5, 128.4, 128.0, 127.6, 127.1, 126.3, 124.6, 48.6, 47.4, 21.5, 21.4; IR (film): ν (cm^{-1}) 3271, 3026, 2922, 1598, 1494, 1419, 1320, 1290, 1150, 1092, 1074, 961, 819, 789, 751, 701, 690, 671, 551; HR-MS (ESI) m/z calcd for $C_{24}H_{26}NO_2S$ [M+H$^+$] 392.1679, found 392.1679; $[\alpha]_D^{24.3}$ = -20.0 (c = 0.1, $CHCl_3$); HPLC conditions: AD-H column, hexane/2-propanol = 90/10, flow rate = 1.0 mL/min, λ = 254 nm, t_R = 20.2 min (minor), t_R = 23.6 min (major), 95:5 er.

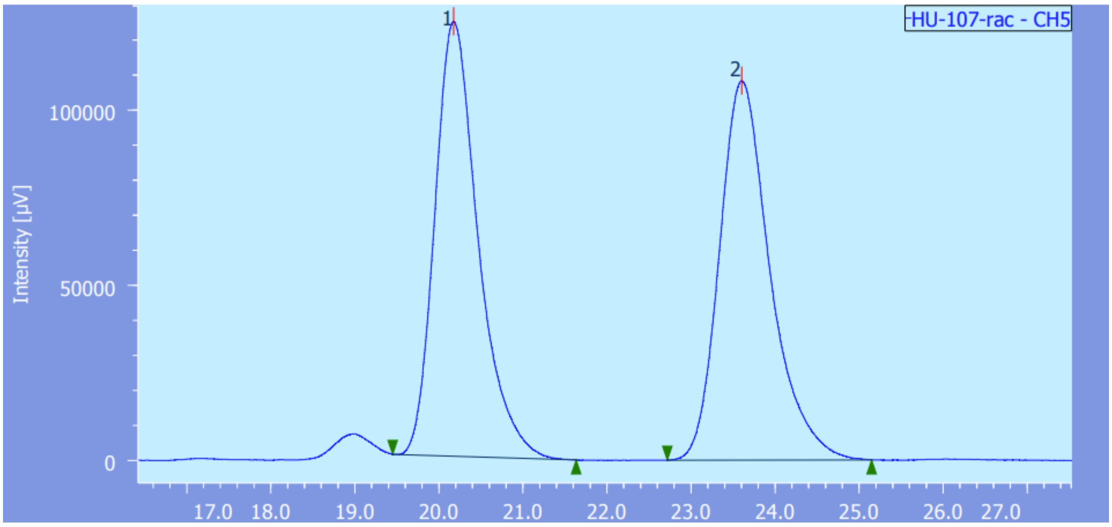

Decision

#	Peak Name	CH	tR [min]	Area [µV·sec]	Height [µV]	Area%	Height%	Quantity	NTP	Resolution	Symmetry Factor	Warning
1	Unknown	5	20.173	4444775	123927	49.542	53.401	N/A	7881	3.499	1.340	
2	Unknown	5	23.600	4527014	108142	50.458	46.599	N/A	8009	N/A	1.310	

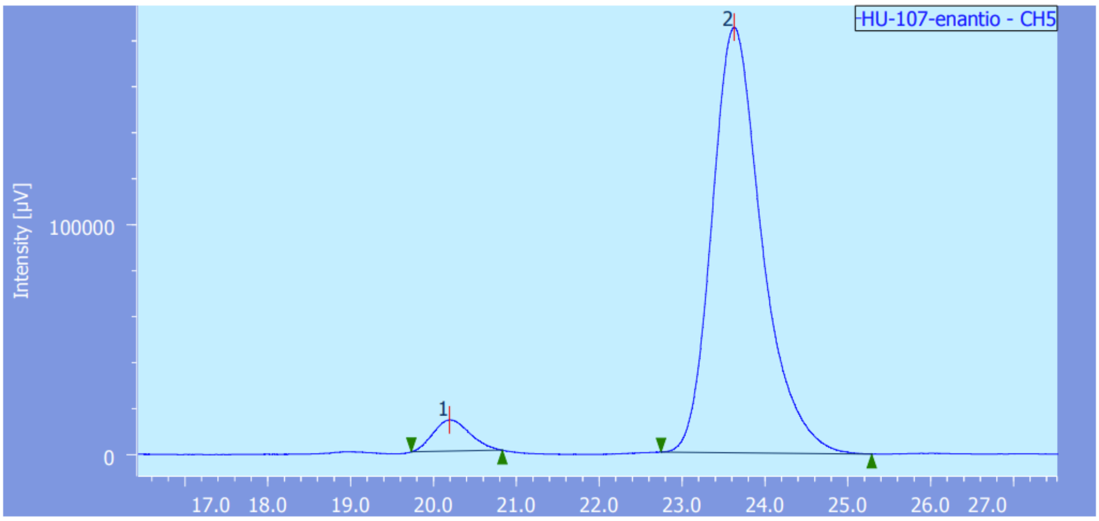

Decision

#	Peak Name	CH	tR [min]	Area [µV·sec]	Height [µV]	Area%	Height%	Quantity	NTP	Resolution	Symmetry Factor	Warning
1	Unknown	5	20.193	421009	13576	5.148	6.836	N/A	9314	3.633	1.177	
2	Unknown	5	23.627	7757563	185013	94.852	93.164	N/A	7974	N/A	1.296	

(*S*, *E*)-4-Methyl-*N*-(2-(naphthalen-2-yl)-4-phenylbut-3-en-1-yl)benzenesulfonamide (13)

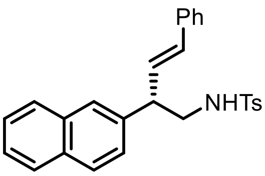

Prepared by **GP-B**. White solid, 61.6 mg, 72% yield. m.p. = 96-97 °C; ^{1}H NMR (400 MHz, $CDCl_3$) δ7.75 – 7.63 (m, 3H), 7.62 – 7.55 (m, 2H), 7.47 (s, 1H), 7.42 – 7.34 (m, 2H), 7.25 – 7.11 (m, 8H), 6.40 – 6.27 (m, 1H), 6.18 (dd, *J* = 15.9, 7.6 Hz, 1H), 4.46 (t, *J* = 6.2 Hz, 1H), 3.66 (q, *J* = 7.5 Hz, 1H), 3.43 – 3.24 (m, 2H), 2.32 (s, 3H); ^{13}C NMR (101 MHz, $CDCl_3$)

δ 143.4, 137.6, 136.8, 136.5, 133.4, 132.5, 132.3, 129.7, 129.2, 128.7, 128.5, 127.6, 127.6, 127.0, 126.4, 126.3, 125.9, 125.6, 48.7, 47.4, 21.5; IR (film): ν (cm^{-1}) 3274, 3055, 3025, 1597, 1495, 1425, 1324, 1291, 1152, 1092, 1073, 1064, 963, 906, 857, 815, 742, 730, 691, 669, 662, 552, 475; HR-MS (ESI) m/z calcd for $C_{27}H_{26}NO_2S$ [M+H$^+$] 428.1679, found 428.1680; $[\alpha]_D^{23.6}$ = -5.6 (c = 0.1, $CHCl_3$); HPLC conditions: OD-H column, hexane/2-propanol = 90/10, flow rate = 1.0 mL/min, λ = 254 nm, t_R = 25.2 min (major), t_R = 29.7 min (minor), 95:5 er.

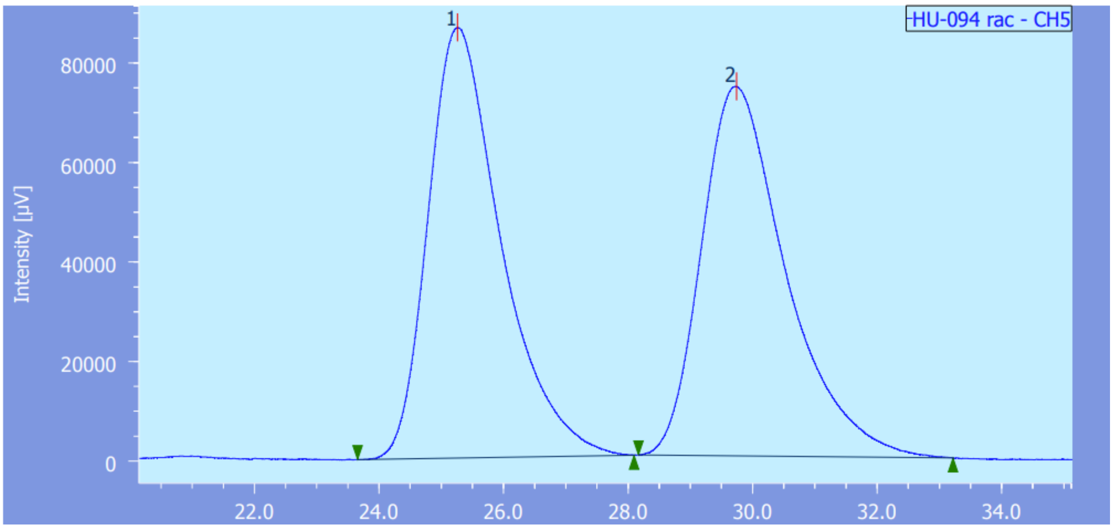

Decision

#	Peak Name	CH	tR [min]	Area [µV·sec]	Height [µV]	Area%	Height%	Quantity	NTP	Resolution	Symmetry Factor	Warning
1	Unknown	5	25.257	7044743	86481	50.115	53.807	N/A	2392	2.019	1.455	
2	Unknown	5	29.740	7012412	74245	49.885	46.193	N/A	2482	N/A	1.440	

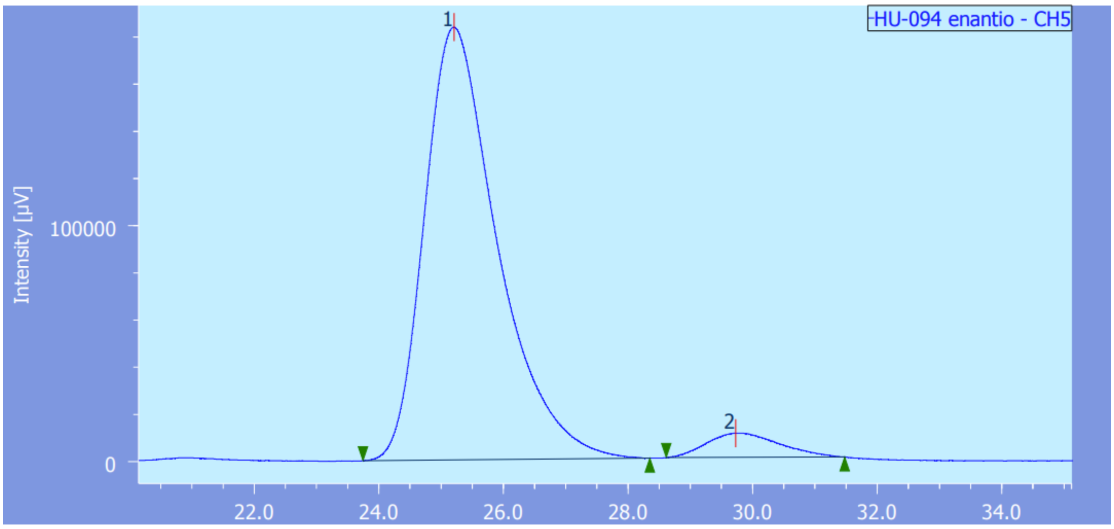

Decision

#	Peak Name	CH	tR [min]	Area [µV·sec]	Height [µV]	Area%	Height%	Quantity	NTP	Resolution	Symmetry Factor	Warning
1	Unknown	5	25.207	14927277	183348	94.701	94.701	N/A	2388	2.123	1.457	
2	Unknown	5	29.723	835203	10259	5.299	5.299	N/A	2914	N/A	1.285	

***tert*-Butyl (*S*, *E*)-5-(1-((4-methylphenyl)sulfonamido)-4-phenylbut-3-en-2-yl)-1H-indole-1-carboxylate (14)**

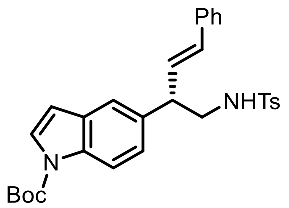

Prepared by **GP-A**. White solid, 50.8 mg, 49% yield. m.p. = 69-70 °C; ^{1}H NMR (400 MHz, $CDCl_3$) δ 7.98 (d, J = 8.4 Hz, 1H), 7.64 – 7.56 (m, 2H), 7.52 (d, J = 3.6 Hz, 1H), 7.26 – 7.10 (m, 8H), 6.98 (dd, J = 8.6, 1.7 Hz, 1H), 6.44 – 6.39 (m, 1H), 6.30 (dd, J = 16.0, 0.5 Hz, 1H), 6.22 – 6.11 (m, 1H), 4.34 (t, J = 6.1 Hz, 1H), 3.59 (q, J = 7.5 Hz, 1H), 3.39 – 3.18 (m, 2H), 2.35 (s, 3H), 1.59 (s, 9H); ^{13}C NMR (101 MHz, $CDCl_3$) δ 149.6, 143.4, 136.8, 136.6, 134.4, 131.9, 131.0, 129.7, 129.7, 128.5, 127.6, 127.1, 126.5, 126.3, 123.8, 119.9, 115.6, 107.1, 83.8, 48.4, 47.7, 28.2, 21.5; IR (film): ν (cm^{-1}) 3281, 2978, 2924, 2853, 2366, 2358, 1729, 1598, 1495, 1468, 1445, 1371, 1352, 1327, 1254, 1157, 1131, 1083, 1022, 965, 838, 813, 749, 730, 693, 663, 551; HR-MS (ESI) m/z calcd for $C_{30}H_{33}N_2O_4S$ [M+H$^+$] 517.2156, found 517.2155; $[\alpha]_D^{24.0}$ = -12.2 (c = 0.1, $CHCl_3$); HPLC conditions: OD-H column, hexane/2-propanol = 90/10, flow rate = 1.0 mL/min, λ = 254 nm, t_R = 19.9 min (major), t_R = 30.2 min (minor), 90:10 er.

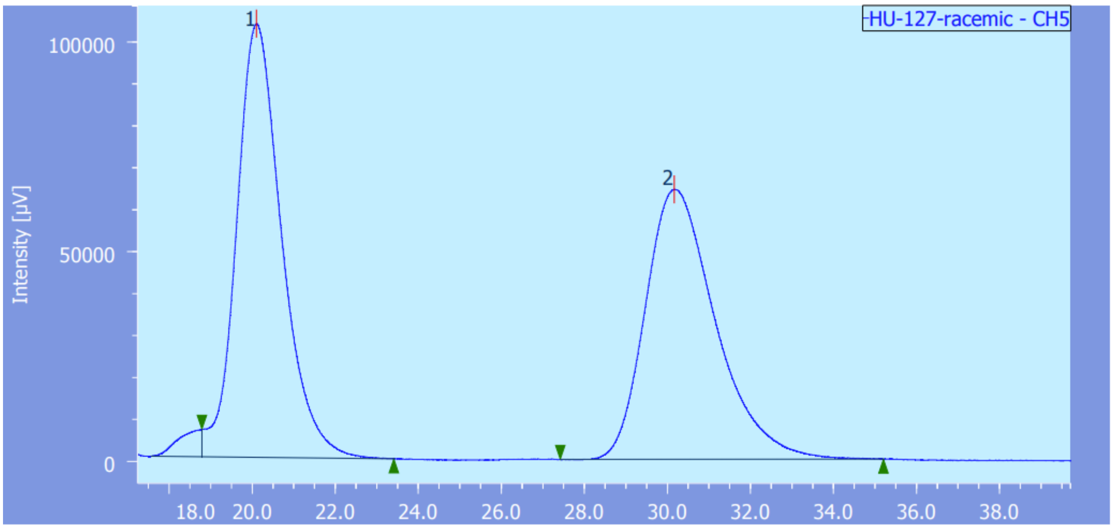

Decision

#	Peak Name	CH	tR [min]	Area [µV·sec]	Height [µV]	Area%	Height%	Quantity	NTP	Resolution	Symmetry Factor	Warning
1	Unknown	5	20.100	7819997	103312	50.871	61.620	N/A	1777	4.104	N/A	
2	Unknown	5	30.160	7552360	64348	49.129	38.380	N/A	1608	N/A	1.383	

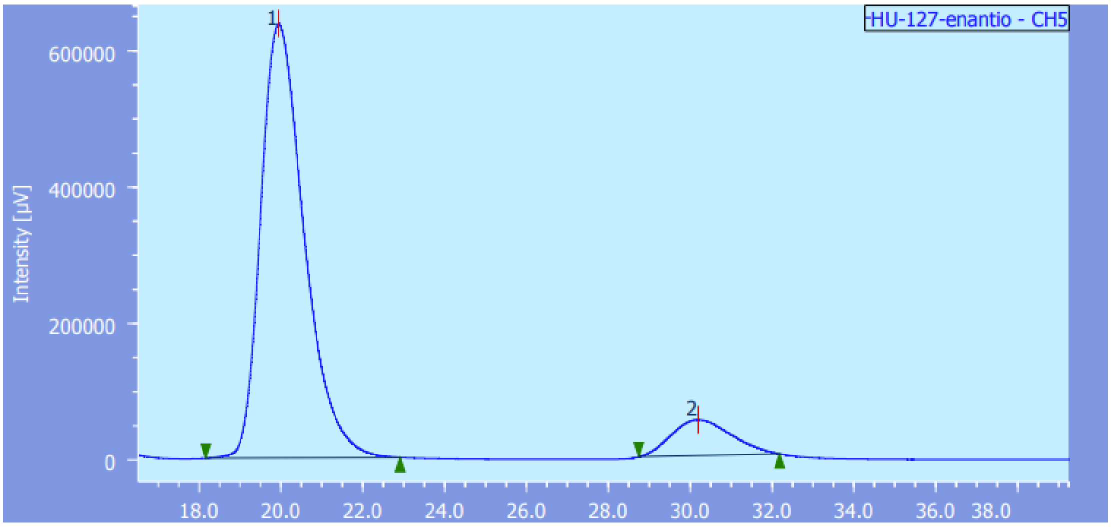

Decision

#	Peak Name	CH	tR [min]	Area [µV·sec]	Height [µV]	Area%	Height%	Quantity	NTP	Resolution	Symmetry Factor	Warning
1	Unknown	5	19.937	46942580	636591	89.788	92.392	N/A	1767	4.385	1.409	
2	Unknown	5	30.187	5338758	52420	10.212	7.608	N/A	1872	N/A	1.191	

(*S*, *E*)-4-Methyl-N-(2-phenyl-4-(p-tolyl)but-3-en-1-yl)benzenesulfonamide (15)

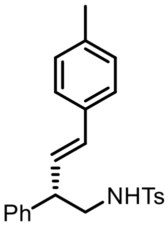

Prepared by **GP-A**. White solid, 48.0 mg, 61% yield. m.p. = 156-157 °C; ^{1}H NMR (400 MHz, $CDCl_3$) δ 7.69 – 7.55 (m, 2H), 7.26 – 7.13 (m, 5H), 7.13 – 7.08 (m, 2H), 7.08 – 6.98 (m, 4H), 6.27 (d, *J* = 15.9 Hz, 1H), 6.03 (dd, *J* = 15.9, 7.9 Hz, 1H), 4.33 (t, *J* = 6.1 Hz, 1H), 3.47 (q, *J* = 7.6 Hz, 1H), 3.34 – 3.12 (m, 2H), 2.36 (s, 3H), 2.24 (s, 3H); ^{13}C NMR (101 MHz, $CDCl_3$) δ 143.5, 140.3, 137.6, 136.9, 133.7, 132.1, 129.7, 129.2, 128.9, 128.1, 127.6, 127.2, 127.1, 126.2, 48.6, 47.5, 21.5, 21.2; IR (film): ν (cm^{-1}) 3294, 3027, 2919, 1596, 1512, 1495, 1453, 1442, 1423, 1317, 1291, 1148, 1090, 1068, 1026, 966, 864, 840, 816, 800, 695, 666, 552, 530, 495; HR-MS (ESI) m/z calcd for $C_{24}H_{26}NO_2S$ [M+H$^+$] 392.1679, found 392.1682; $[\alpha]_D^{24.2}$ = -24.5 (c = 0.1, $CHCl_3$); HPLC conditions: OD-H column, hexane/2-propanol = 95/5, flow rate = 0.5 mL/min, λ = 254 nm, t_R = 41.9 min (minor), t_R = 71.4 min (major), 96:4 er.

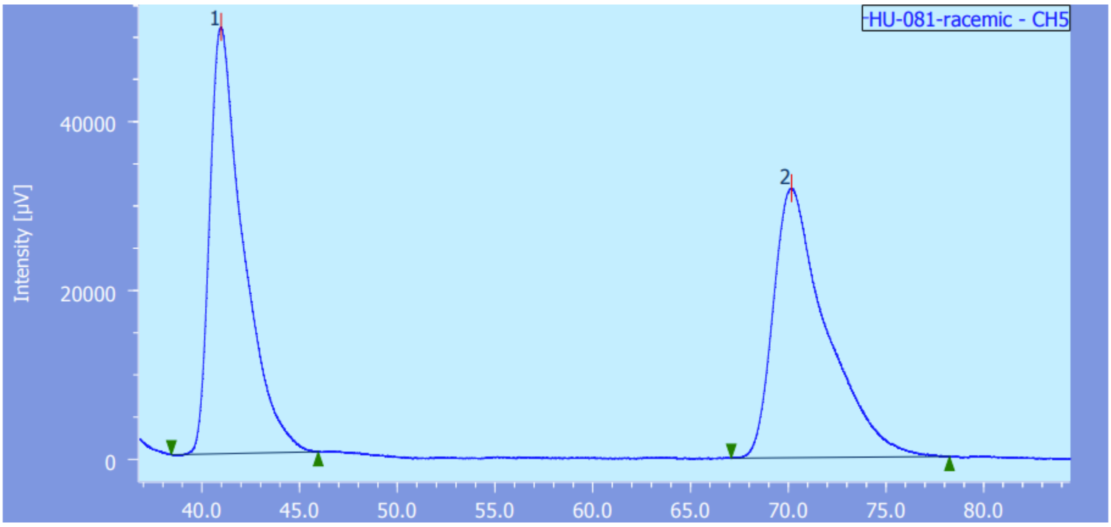

Decision

#	Peak Name	CH	tR [min]	Area [µV·sec]	Height [µV]	Area%	Height%	Quantity	NTP	Resolution	Symmetry Factor	Warning
1	Unknown	5	40.980	6044541	50532	50.097	61.288	N/A	3069	7.805	1.871	
2	Unknown	5	70.153	6021112	31918	49.903	38.712	N/A	3826	N/A	1.784	

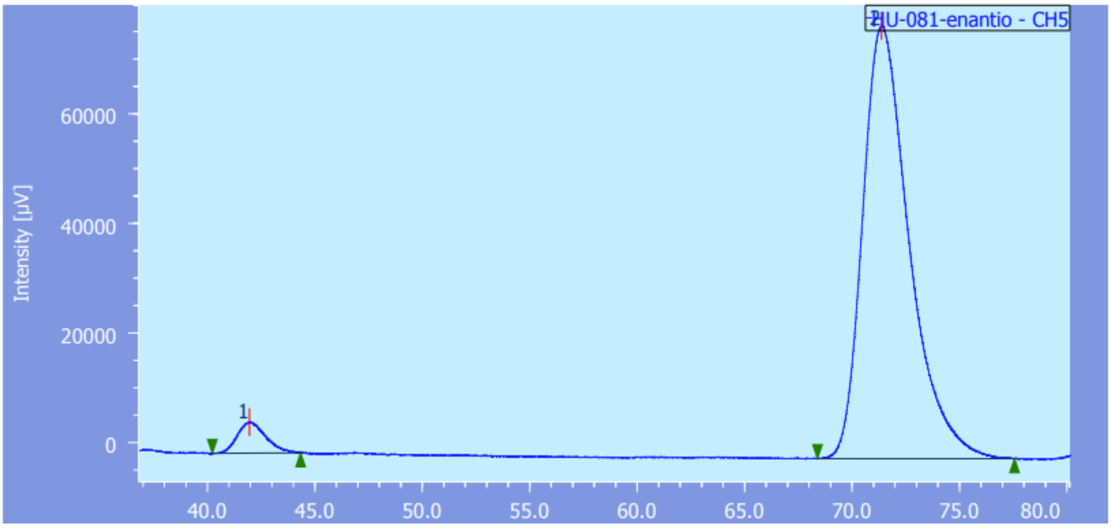

Decision

#	Peak Name	CH	tR [min]	Area [µV·sec]	Height [µV]	Area%	Height%	Quantity	NTP	Resolution	Symmetry Factor	Warning
1	Unknown	5	41.947	517168	5621	4.177	6.646	N/A	4889	9.507	1.261	
2	Unknown	5	71.357	11863333	78957	95.823	93.354	N/A	5631	N/A	1.443	

(*S*, *E*)-*N*-(4-(4-Methoxyphenyl)-2-phenylbut-3-en-1-yl)-4-methylbenzenesulfonamide (16)

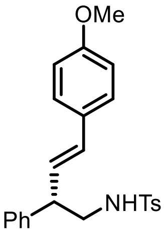

Prepared by **GP-A**. White solid, 57.1 mg, 70% yield. m.p. = 134-135 ºC; ^{1}H NMR (400 MHz, $CDCl_3$) δ 7.66 – 7.58 (m, 2H), 7.24 – 7.11 (m, 7H), 7.08 – 7.01 (m, 2H), 6.77 – 6.69 (m, 2H), 6.24 (d, *J* = 15.9 Hz, 1H) 5.94 (dd, *J* = 15.9, 8.0 Hz, 1H), 4.41 (t, *J* = 6.2 Hz, 1H), 3.71 (s, 3H), 3.45 (q, *J* = 7.6 Hz, 1H), 3.27 – 3.17 (m, 2H), 2.35 (s, 3H); ^{13}C NMR (101 MHz, $CDCl_3$) δ 159.2, 143.4, 140.5, 136.9, 131.7, 129.7, 129.3, 128.9, 127.6, 127.4, 127.2, 127.1, 126.9, 113.9, 55.2, 48.6, 47.5, 21.5; IR (film): ν (cm^{-1}) 3290, 2932, 2833, 1607, 1509, 1493, 1452, 1443, 1422, 1320, 1311, 1293, 1250, 1175, 1149, 1092, 1068, 1030, 962, 865, 842, 819, 811, 698, 669, 552, 534, 514; HR-MS (ESI) m/z calcd for $C_{24}H_{26}NO_3S$ [M+H^{+}] 408.1628, found 408.1628; $[\alpha]_D^{24.2}$ = -27.1 (c = 0.1, $CHCl_3$); HPLC conditions: OD-H column, hexane/2-propanol = 90/10, flow rate = 1.0 mL/min, λ = 254 nm, t_R = 19.5 min (minor), t_R = 32.2 min (major), 97:3 er.

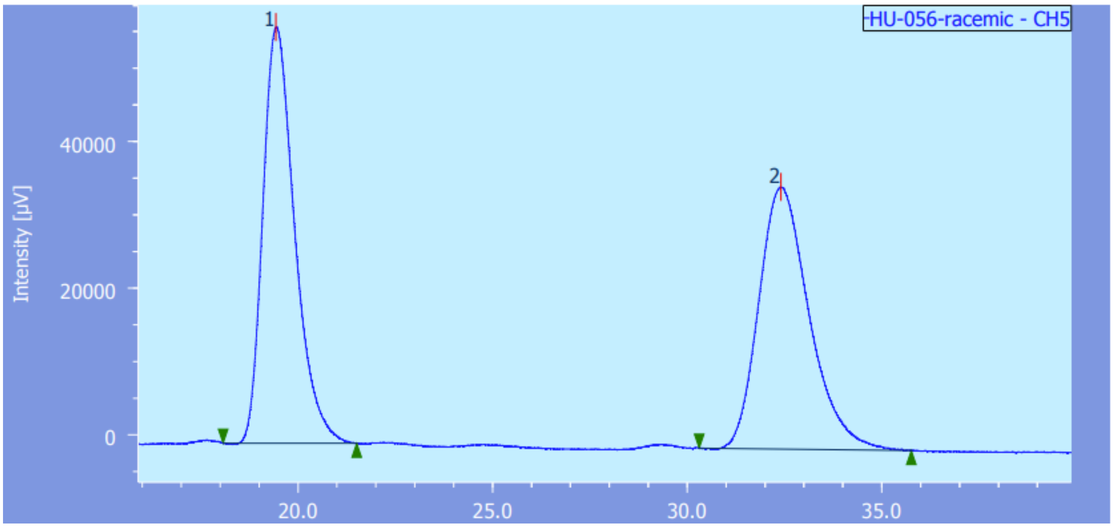

Decision

#	Peak Name	CH	tR [min]	Area [µV·sec]	Height [µV]	Area%	Height%	Quantity	NTP	Resolution	Symmetry Factor	Warning
1	Unknown	5	19.433	3223209	56698	50.126	61.364	N/A	2817	6.880	1.388	
2	Unknown	5	32.403	3207059	35698	49.874	38.636	N/A	3132	N/A	1.290	

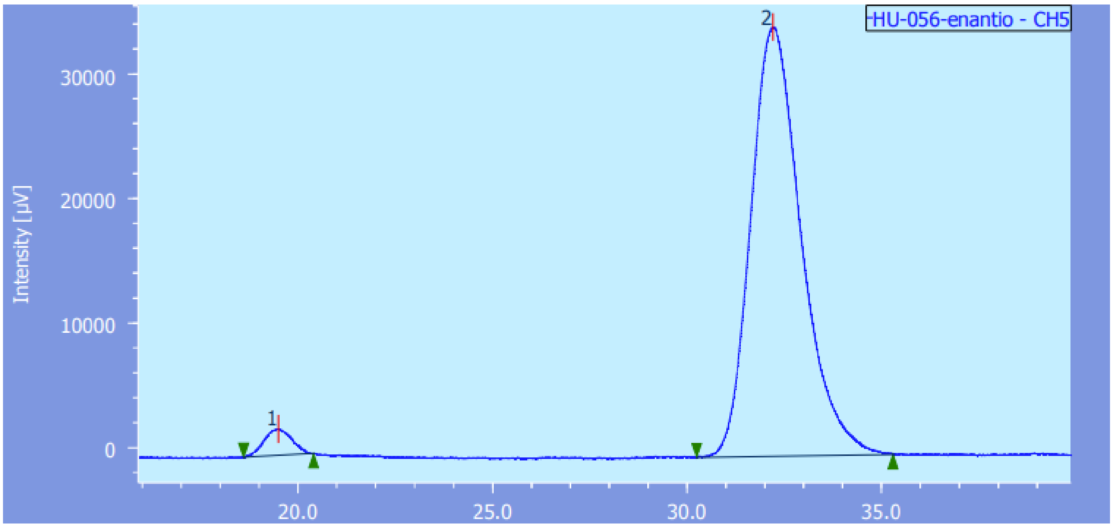

Decision

#	Peak Name	CH	tR [min]	Area [µV·sec]	Height [µV]	Area%	Height%	Quantity	NTP	Resolution	Symmetry Factor	Warning
1	Unknown	5	19.500	107167	2139	3.331	5.846	N/A	3323	6.942	0.949	
2	Unknown	5	32.203	3110279	34447	96.669	94.154	N/A	3092	N/A	1.296	

(*S*, *E*)-*N*-(4-(4-((*tert*-Butyldimethylsilyl)oxy)phenyl)-2-phenylbut-3-en-1-yl)-4-methylbenzenesulfonamide (17)

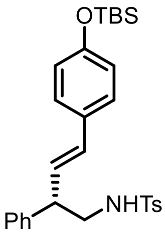

Prepared by **GP-A**. White solid, 71 mg, 70% yield. m.p. = 86-87 °C; ^{1}H NMR (400 MHz, $CDCl_3$) δ 7.56 – 7.45 (m, 2H), 7.15 – 7.01 (m, 5H), 7.00 – 6.90 (m, 4H), 6.60 – 6.54 (m, 2H), 6.12 (d, *J* = 15.8 Hz, 1H), 5.83 (dd, *J* = 15.9, 8.0 Hz, 1H), 4.33 (t, *J* = 6.2 Hz, 1H), 3.34 (q, *J* = 7.6 Hz, 1H), 3.17 – 3.04 (m, 2H), 2.24 (s, 3H), 0.79 (s, 9H), -0.00 (s, 6H); ^{13}C NMR (101 MHz, $CDCl_3$) δ 155.4, 143.4, 140.5, 136.9, 131.7, 129.9, 129.7, 128.9, 127.6, 127.4, 127.2, 127.1, 120.2, 115.7, 48.6, 47.5, 25.6, 21.5, 18.2, -4.5; IR (film): ν (cm^{-1}) 3254, 3029, 2953, 2929, 2856, 1604, 1509, 1471, 1453, 1429, 1416, 1329, 1261, 1152, 1094, 1070, 974, 914, 843, 780, 757, 699, 665, 552, 528; HR-MS (ESI) m/z calcd for $C_{29}H_{38}NO_3SSi$ [M+H$^+$] 508.2336, found 508.2340; $[\alpha]_D^{24.0}$ = -22.4 (c = 0.1, $CHCl_3$); HPLC conditions: OD-H column, hexane/2-propanol = 90/10, flow rate = 1.0 mL/min, λ = 254 nm, t_R = 8.5 min (minor), t_R = 11.7 min (major), 95:5 er.

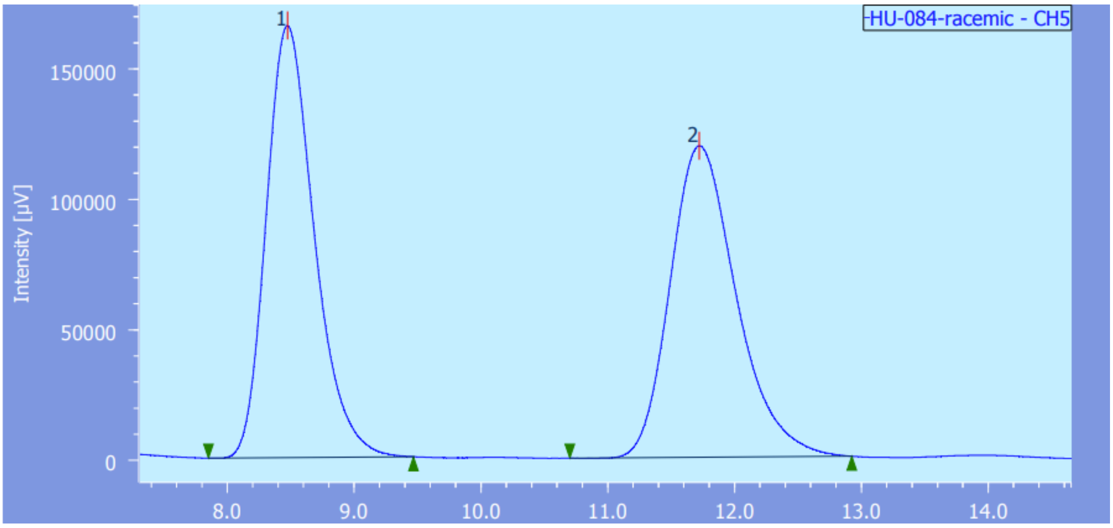

Decision

#	Peak Name	CH	tR [min]	Area [µV·sec]	Height [µV]	Area%	Height%	Quantity	NTP	Resolution	Symmetry Factor	Warning
1	Unknown	5	8.473	4357598	165513	50.125	58.091	N/A	2479	4.023	1.282	
2	Unknown	5	11.720	4335878	119407	49.875	41.909	N/A	2499	N/A	1.285	

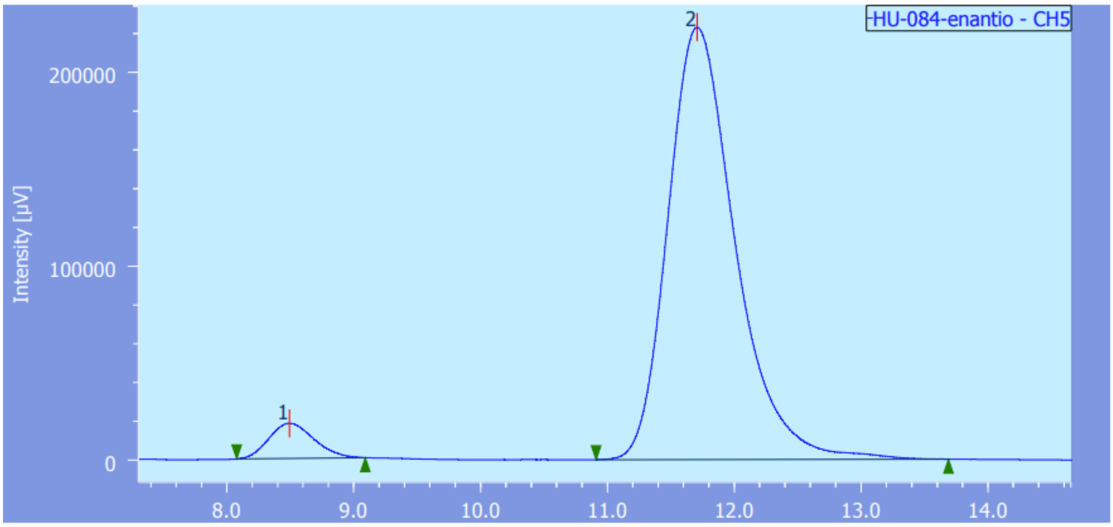

Decision

#	Peak Name	CH	tR [min]	Area [µV·sec]	Height [µV]	Area%	Height%	Quantity	NTP	Resolution	Symmetry Factor	Warning
1	Unknown	5	8.493	450791	18128	5.199	7.524	N/A	2631	4.033	1.179	
2	Unknown	5	11.707	8220637	222802	94.801	92.476	N/A	2506	N/A	1.322	

(*S*, *E*)-4-(4-((4-Methylphenyl)sulfonamido)-3-phenylbut-1-en-1-yl)phenyl acetate (18)

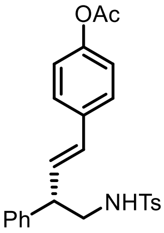

Prepared by **GP-A**. White solid, 77.4 mg, 89% yield. m.p. = 159-160 °C; ^{1}H NMR (400 MHz, $CDCl_3$) δ 7.67 – 7.56 (m, 2H), 7.26 – 7.13 (m, 7H), 7.07 – 7.01 (m, 2H), 6.96 – 6.88 (m, 2H), 6.27 (d, *J* = 15.9 Hz, 1H), 6.05 (dd, *J* = 15.9, 7.9 Hz, 1H), 4.43 (t, *J* = 6.2 Hz, 1H), 3.49 (q, *J* = 7.6 Hz, 1H), 3.33 – 3.14 (m, 2H), 2.34 (s, 3H), 2.20 (s, 3H); ^{13}C NMR (101 MHz, $CDCl_3$) δ 169.4, 150.0, 143.5, 140.1, 136.8, 134.3, 131.2, 129.7, 129.6, 129.0, 127.6, 127.3, 127.2, 127.1, 121.6, 48.6, 47.4, 21.5, 21.1; IR (film): ν (cm^{-1}) 3290, 3031, 2923, 1755, 1599, 1505, 1444, 1425, 1374, 1321, 1198, 1166, 1150, 1092, 1071, 1016, 962, 911, 870, 852, 818, 778, 696, 666, 552, 534, 511; HR-MS (ESI) m/z calcd for $C_{25}H_{26}NO_4S$ [M+H$^+$] 436.1577, found 436.1579; $[\alpha]_D^{24.0}$ = -30.6 (c = 0.1, $CHCl_3$); HPLC conditions: OD-H column, hexane/2-propanol = 90/10, flow rate = 1.0 mL/min, λ = 254 nm, t_R = 33.8 min (minor), t_R = 48.9 min (major), 95:5 er.

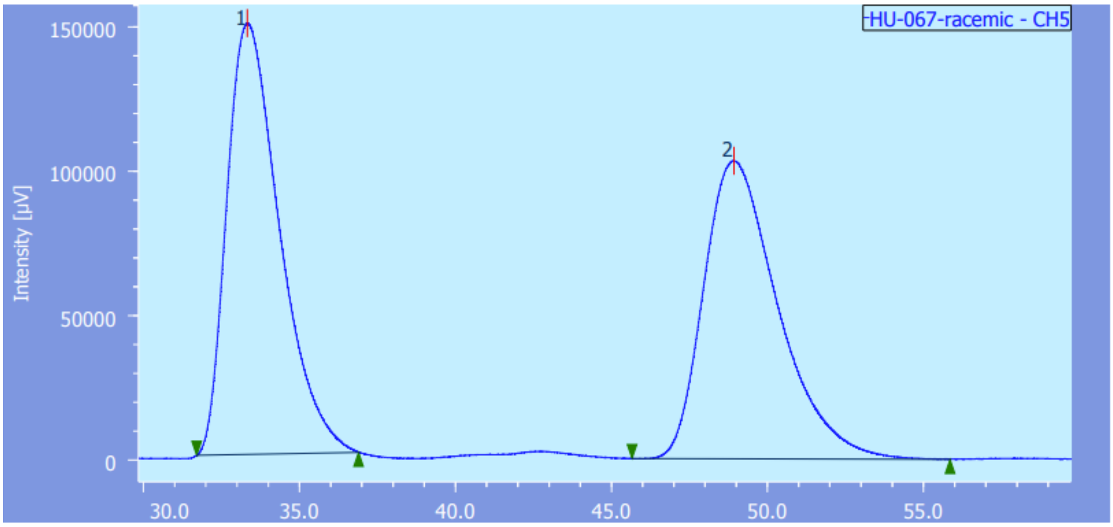

Decision

#	Peak Name	CH	tR [min]	Area [µV·sec]	Height [µV]	Area%	Height%	Quantity	NTP	Resolution	Symmetry Factor	Warning
1	Unknown	5	33.323	17549210	149198	50.681	59.122	N/A	1874	4.252	1.520	
2	Unknown	5	48.917	17077764	103157	49.319	40.878	N/A	2095	N/A	1.442	

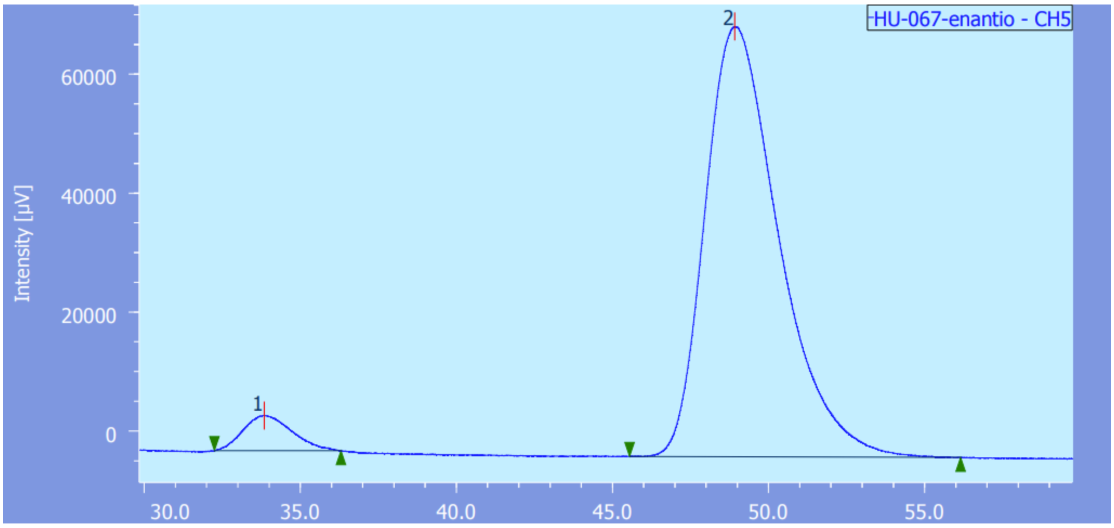

Decision

#	Peak Name	CH	tR [min]	Area [µV·sec]	Height [µV]	Area%	Height%	Quantity	NTP	Resolution	Symmetry Factor	Warning
1	Unknown	5	33.843	639540	5880	5.113	7.522	N/A	2141	4.225	1.257	
2	Unknown	5	48.910	11867981	72293	94.887	92.478	N/A	2143	N/A	1.445	

(*S*, *E*)-4-Methyl-*N*-(2-phenyl-4-(4-(trifluoromethoxy)phenyl)but-3-en-1-yl)benzenesulfonamide (19)

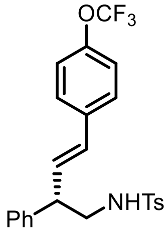

Prepared by **GP-A**. White solid, 71.8 mg, 78% yield. m.p. = 119-120 °C; ^{1}H NMR (400 MHz, $CDCl_3$) δ 7.65 – 7.58 (m, 2H), 7.25 – 7.14 (m, 7H), 7.07 – 7.00 (m, 4H), 6.28 (d, *J* = 15.9 Hz, 1H), 6.09 (dd, *J* = 15.9, 7.8 Hz, 1H), 4.52 (t, *J* = 6.2 Hz, 1H), 3.51 (q, *J* = 7.5 Hz, 1H), 3.31 – 3.16 (m, 2H), 2.34 (s, 3H); ^{13}C NMR (101 MHz, $CDCl_3$) δ 148.5, 143.5, 140.0, 136.8, 135.4, 130.7, 130.5, 129.7, 129.0, 127.6, 127.5, 127.4, 127.1, 121.0, 120.4 (q, *J* = 258.0 Hz), 48.7, 47.4, 21.5; ^{19}F NMR (377 MHz, $CDCl_3$) δ -57.85; IR (film): ν (cm^{-1}) 3291, 3029, 2924, 2364, 1598, 1506, 1496, 1453, 1446, 1425, 1320, 1312, 1259, 1215, 1149, 1093, 1070, 1033, 1019, 972, 963, 860, 817, 696, 666, 552, 537, 520; HR-MS (ESI) m/z calcd for $C_{24}H_{23}F_3NO_3S$ [M+H$^+$] 462.1345, found 462.1346; $[\alpha]_D^{22.6}$ = -32.7 (c = 0.1, $CHCl_3$); HPLC conditions: OD-H column, hexane/2-propanol = 90/10, flow rate = 1.0 mL/min, λ = 254 nm, t_R = 10.8 min (minor), t_R = 18.9 min (major), 95:5 er.

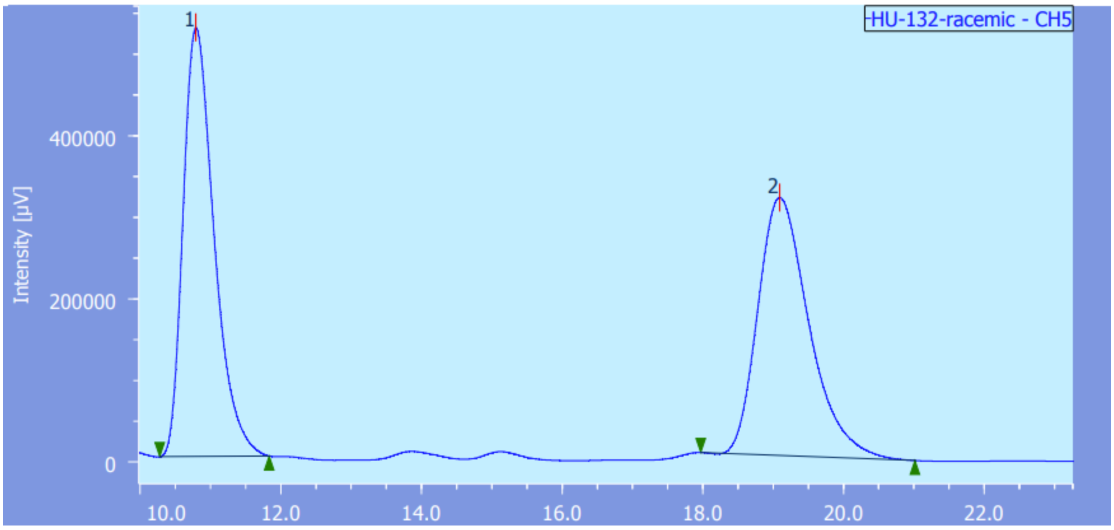

Decision

#	Peak Name	CH	tR [min]	Area [μV·sec]	Height [μV]	Area%	Height%	Quantity	NTP	Resolution	Symmetry Factor	Warning
1	Unknown	5	10.793	16083447	525705	50.179	62.449	N/A	2985	7.949	1.425	
2	Unknown	5	19.090	15968851	316107	49.821	37.551	N/A	3435	N/A	1.367	

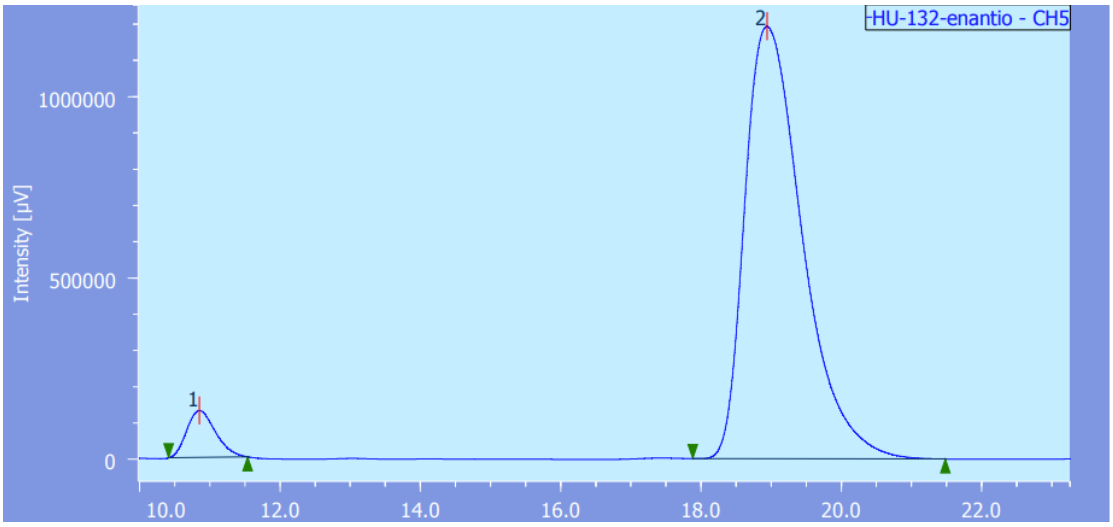

Decision

#	Peak Name	CH	tR [min]	Area [μV·sec]	Height [μV]	Area%	Height%	Quantity	NTP	Resolution	Symmetry Factor	Warning
1	Unknown	5	10.850	3691692	129254	5.150	9.782	N/A	3269	7.263	1.259	
2	Unknown	5	18.940	67996045	1192153	94.850	90.218	N/A	2639	N/A	1.560	

(*S*, *E*)-*N*-(4-(4-Fluorophenyl)-2-phenylbut-3-en-1-yl)-4-methylbenzenesulfonamide (20)

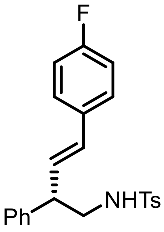

Prepared by **GP-A**. White solid, 61.0 mg, 77% yield. m.p. = 123-124 °C; ^{1}H NMR (400 MHz, $CDCl_3$) δ 7.67 – 7.55 (m, 2H), 7.25 – 7.12 (m, 7H), 7.08 – 7.00 (m, 2H), 6.93 – 6.82 (m, 2H), 6.25 (d, J = 15.9 Hz, 1H), 6.02 (dd, J = 15.9, 7.9 Hz, 1H), 4.48 (t, J = 6.2 Hz, 1H), 3.48 (q, J = 7.6 Hz, 1H), 3.30 – 3.13 (m, 2H), 2.34 (s, 3H); ^{13}C NMR (101 MHz, $CDCl_3$) δ 162.3 (d, J = 247.8 Hz), 143.5, 140.2, 136.8, 132.7 (d, J = 3.3 Hz), 131.0, 129.7, 129.05 (d, J = 2.2 Hz), 128.96, 127.8 (d, J = 8.1 Hz), 127.6, 127.3, 127.1, 115.4 (d, J = 21.7 Hz), 48.7, 47.5, 21.5; ^{19}F NMR (377 MHz, $CDCl_3$) δ -114.26; IR (film): ν (cm^{-1}) 3289, 3029, 2922, 2873, 1598, 1507, 1453, 1445, 1424, 1321, 1294, 1224, 1149, 1092, 1073, 1029, 1021, 964, 871, 858, 819, 793, 761, 697, 665, 552, 532, 506, 488; HR-MS (ESI) m/z calcd for $C_{23}H_{23}FNO_2S$ [M+H^{+}] 396.1428, found 396.1429; $[\alpha]_D^{24.3}$ = -19.4 (c = 0.1, $CHCl_3$); HPLC conditions: OD-H column, hexane/2-propanol = 95/5, flow rate = 0.5 mL/min, λ = 254 nm, t_R = 47.3 min (minor), t_R = 82.7 min (major), 95:5 er.

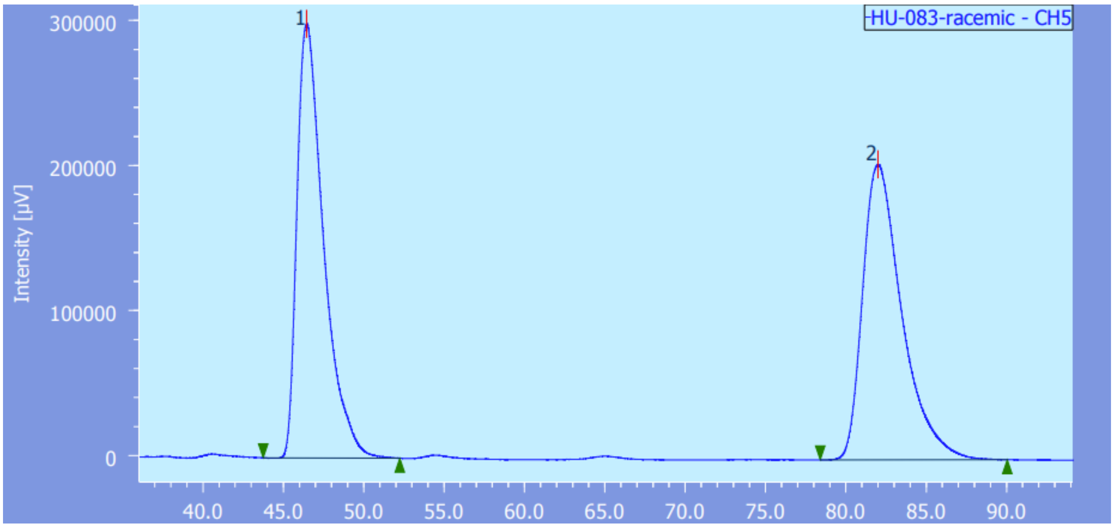

Decision

#	Peak Name	CH	tR [min]	Area [µV·sec]	Height [µV]	Area%	Height%	Quantity	NTP	Resolution	Symmetry Factor	Warning
1	Unknown	5	46.433	34520860	299116	49.997	59.490	N/A	4041	9.902	1.759	
2	Unknown	5	81.980	34524851	203684	50.003	40.510	N/A	5878	N/A	1.565	

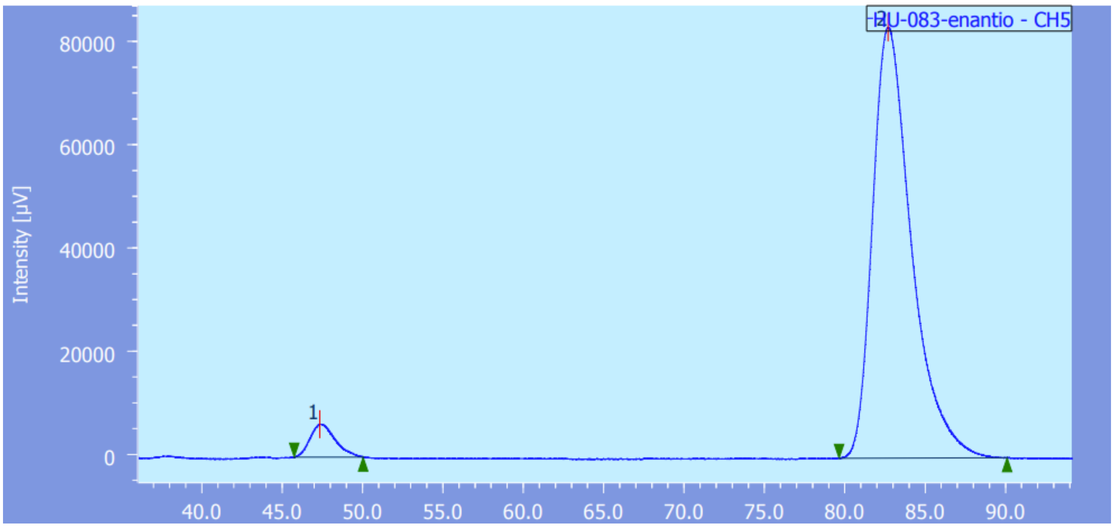

Decision

#	Peak Name	CH	tR [min]	Area [µV·sec]	Height [µV]	Area%	Height%	Quantity	NTP	Resolution	Symmetry Factor	Warning
1	Unknown	5	47.343	689827	6390	4.677	7.118	N/A	4480	10.030	1.333	
2	Unknown	5	82.680	14060827	83392	95.323	92.882	N/A	6096	N/A	1.505	

(*S*, *E*)-*N*-(4-(4-Chlorophenyl)-2-phenylbut-3-en-1-yl)-4-methylbenzenesulfonamide (21)

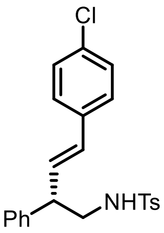

Prepared by **GP-B**. White solid, 62.1 mg, 75% yield. m.p. = 133-134 °C; ^{1}H NMR (400 MHz, $CDCl_3$) δ 7.67 – 7.55 (m, 2H), 7.25 – 7.07 (m, 9H), 7.06 – 7.00 (m, 2H), 6.23 (d, J = 15.9 Hz, 1H), 6.08 (dd, J = 15.9, 7.8 Hz, 1H), 4.57 (t, J = 6.2 Hz, 1H), 3.49 (q, J = 7.6 Hz, 1H), 3.33 – 3.10 (m, 2H), 2.33 (s, 3H); ^{13}C NMR (101 MHz, $CDCl_3$) δ 143.4, 140.0, 136.8, 135.1, 133.2, 130.9, 130.0, 129.7, 129.0, 128.6, 127.6, 127.5, 127.3, 127.0, 48.7, 47.4, 21.5; IR (film): ν (cm^{-1}) 3260, 3030, 2924, 1649, 1599, 1488, 1454, 1430, 1404, 1325, 1306, 1289, 1224, 1149, 1092, 1077, 1010, 970, 840, 813, 788, 753, 695, 659, 552, 534, 494; HR-MS (ESI) m/z calcd for $C_{23}H_{23}ClNO_2S$ [M+H$^+$] 412.1132, found 412.1131; $[\alpha]_D^{24.4}$ = -23.3 (c = 0.1, $CHCl_3$); HPLC conditions: OD-H column, hexane/2-propanol = 90/10, flow rate = 1.0 mL/min, λ = 254 nm, t_R = 14.1 min (minor), t_R = 25.4 min (major), 97:3 er.

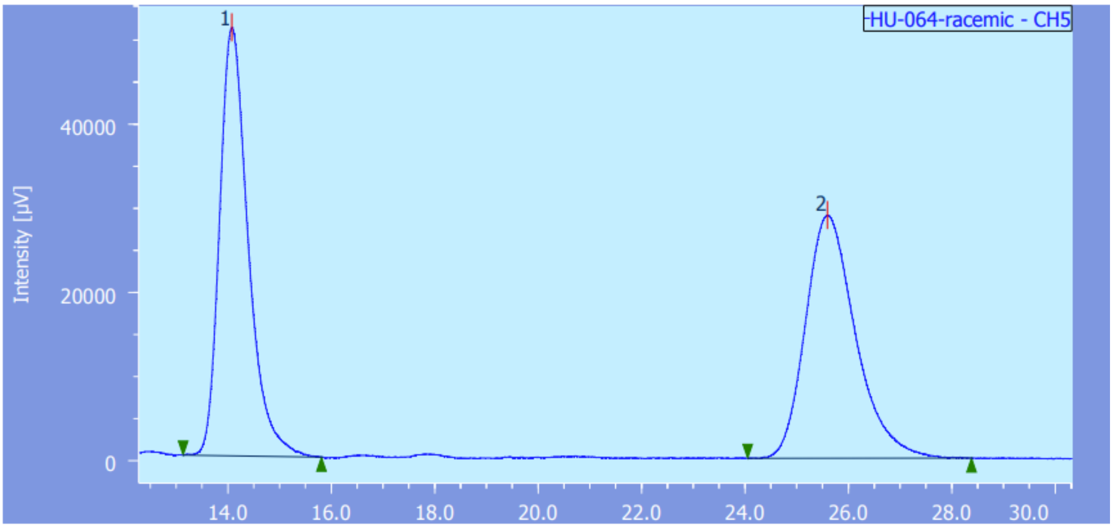

Decision

#	Peak Name	CH	tR [min]	Area [µV·sec]	Height [µV]	Area%	Height%	Quantity	NTP	Resolution	Symmetry Factor	Warning
1	Unknown	5	14.077	1957753	50919	50.171	63.808	N/A	3351	8.570	1.368	
2	Unknown	5	25.590	1944385	28881	49.829	36.192	N/A	3536	N/A	1.300	

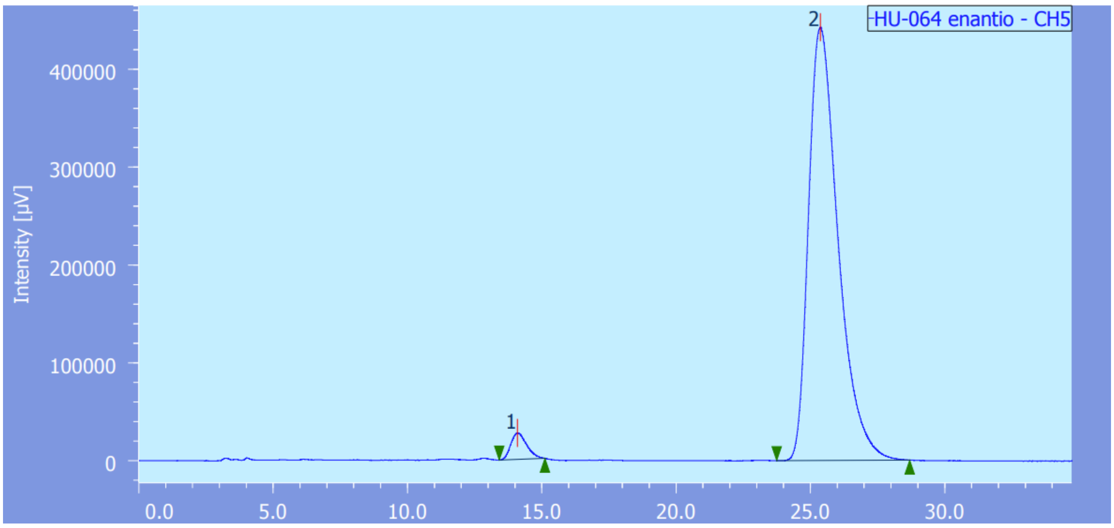

Decision

#	Peak Name	CH	tR [min]	Area [µV·sec]	Height [µV]	Area%	Height%	Quantity	NTP	Resolution	Symmetry Factor	Warning
1	Unknown	5	14.083	1114490	27054	3.188	5.765	N/A	2667	7.411	1.320	
2	Unknown	5	25.357	33842215	442204	96.812	94.235	N/A	2679	N/A	1.507	

(*S*, *E*)-4-Methyl-*N*-(2-phenyl-4-(4-(trifluoromethyl)phenyl)but-3-en-1-yl)benzenesulfonamide (22)

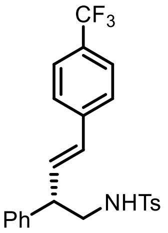

Prepared by **GP-A**. White solid, 83.5 mg, 94% yield. m.p. = 126-127 °C; ^{1}H NMR (400 MHz, CDCl$_3$) δ 7.65 – 7.55 (m, 2H), 7.46 – 7.37 (m, 2H), 7.30 – 7.12 (m, 7H), 7.09 – 7.00 (m, 2H), 6.32 (d, *J* = 16.0 Hz, 1H), 6.22 (dd, *J* = 15.9, 7.5 Hz, 1H), 4.66 (t, *J* = 6.2 Hz, 1H), 3.53 (q, *J* = 7.5 Hz, 1H), 3.35 – 3.12 (m, 2H), 2.32 (s, 3H); ^{13}C NMR (101 MHz, CDCl$_3$) δ 143.5, 140.1 (d, *J* = 1.2 Hz), 139.8, 136.8, 132.2, 130.8, 129.7, 129.3 (q, *J* = 32.5 Hz), 129.0, 127.7, 127.4, 127.0, 126.4, 125.4(q, *J* = 3.8 Hz), 124.1 (d, *J* = 272.5 Hz), 48.8, 47.4, 21.4; ^{19}F NMR (377 MHz, CDCl$_3$) δ -62.45; IR (film): ν (cm^{-1}) 3276, 3031, 2928, 1651, 1612, 1600, 1494, 1454, 1430, 1414, 1325, 1306, 1288, 1226, 1185, 1151, 1120, 1093, 1065, 1013, 971, 953, 850, 814, 754, 696, 656, 598, 579, 553, 535, 497; HR-MS (ESI) m/z calcd for $C_{23}H_{23}ClNO_2S$ [M+H$^+$] 446.1396, found 446.1398; [α]$_D$ $^{24.3}$

= -25.8 (c = 0.1, $CHCl_3$); HPLC conditions: OD-H column, hexane/2-propanol = 90/10, flow rate = 1.0 mL/min, λ = 254 nm, t_R = 12.4 min (minor), t_R = 24.4 min (major), 95:5 er.

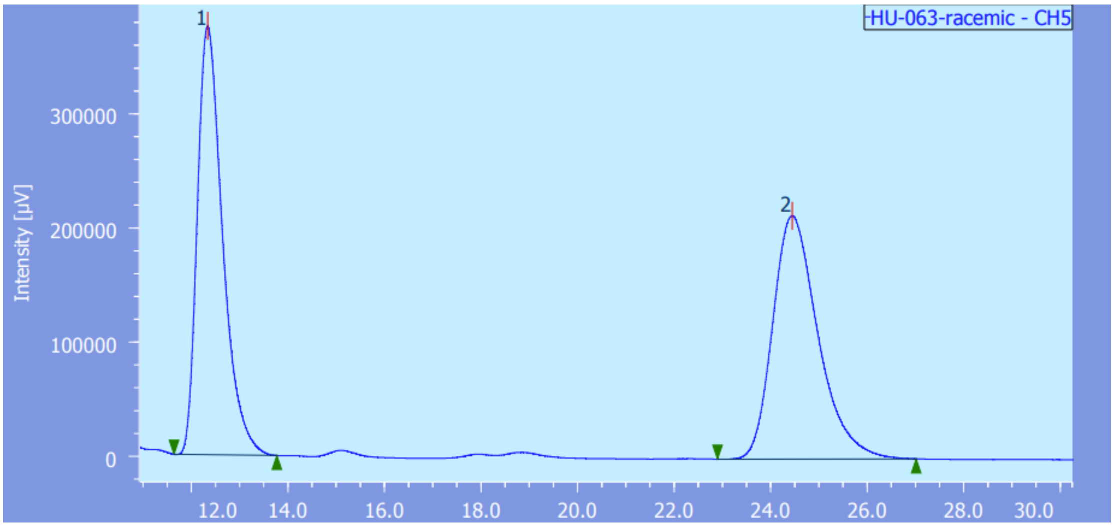

Decision

#	Peak Name	CH	tR [min]	Area [μV·sec]	Height [μV]	Area%	Height%	Quantity	NTP	Resolution	Symmetry Factor	Warning
1	Unknown	5	12.337	13675244	375390	49.623	63.814	N/A	2794	9.323	1.463	
2	Unknown	5	24.443	13882757	212871	50.377	36.186	N/A	3426	N/A	1.342	

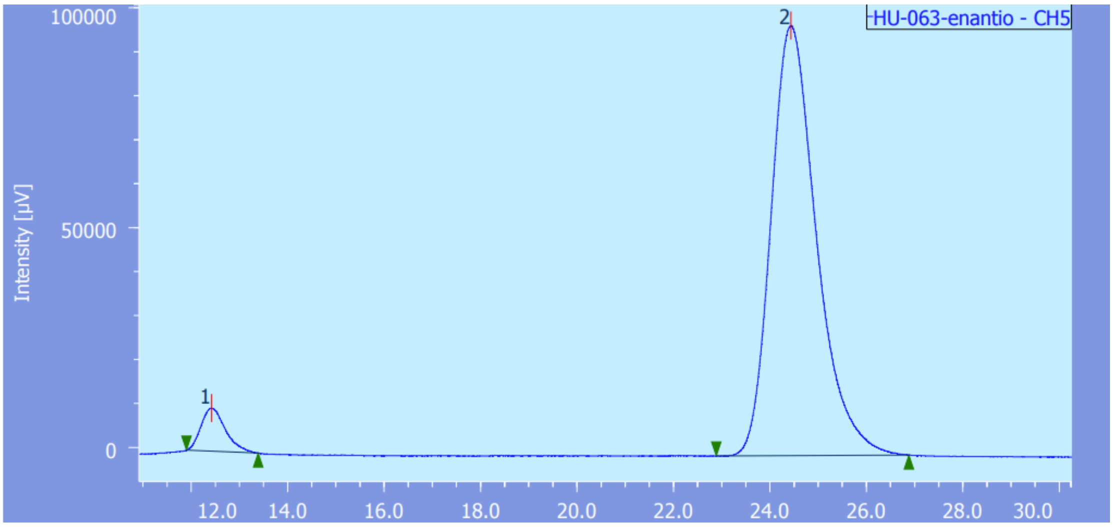

Decision

#	Peak Name	CH	tR [min]	Area [μV·sec]	Height [μV]	Area%	Height%	Quantity	NTP	Resolution	Symmetry Factor	Warning
1	Unknown	5	12.423	344438	9781	5.108	9.093	N/A	2944	9.311	1.331	
2	Unknown	5	24.433	6398499	97784	94.892	90.907	N/A	3422	N/A	1.313	

(*S*, *E*)-4-Methyl-*N*-(2-phenyl-4-(4-(4,4,5,5-tetramethyl-1,3,2-dioxaborolan-2-yl)phenyl)but-3-en-1-yl)benzenesulfonamide (23)

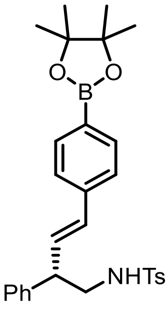

Prepared by **GP-A**. White solid, 60.5 mg, 60% yield. m.p. = 166-167 °C; ^{1}H NMR (400 MHz, $CDCl_3$) δ 7.69 – 7.57 (m, 4H), 7.27 – 7.14 (m, 7H), 7.08 – 7.02 (m, 2H), 6.29 (d, *J* = 15.9 Hz, 1H), 6.16 (dd, *J* = 15.9, 7.7 Hz, 1H), 4.33 (t, *J* = 6.2 Hz, 1H), 3.50 (q, *J* = 7.6 Hz, 1H), 3.31 – 3.17 (m, 2H), 2.36 (s, 3H), 1.26 (s, 12H); ^{13}C NMR (101 MHz, $CDCl_3$) δ 143.5, 140.0, 139.2, 136.8, 135.0, 132.2, 130.3, 129.7, 129.0, 127.7, 127.4, 127.1, 125.6, 83.8, 48.7, 47.4, 24.8, 21.5; IR (film): ν (cm^{-1}) 3249, 2976, 2924, 1606, 1517, 1494, 1448, 1434, 1399, 1360, 1320, 1268, 1144, 1090, 1074, 1024, 1018, 966, 957, 857, 834, 808, 788, 756, 737, 698, 657,

552, 540; HR-MS (ESI) m/z calcd for $C_{29}H_{35}BNO_4S$ [M+H$^+$] 504.2374, found 504.2376; $[\alpha]_D^{24.5}$ = -29.1 (c = 0.1, $CHCl_3$); HPLC conditions: OD-H column, hexane/2-propanol = 90/10, flow rate = 1.0 mL/min, λ = 254 nm, t_R = 12.4 min (minor), t_R = 18.4 min (major), 96:4 er.

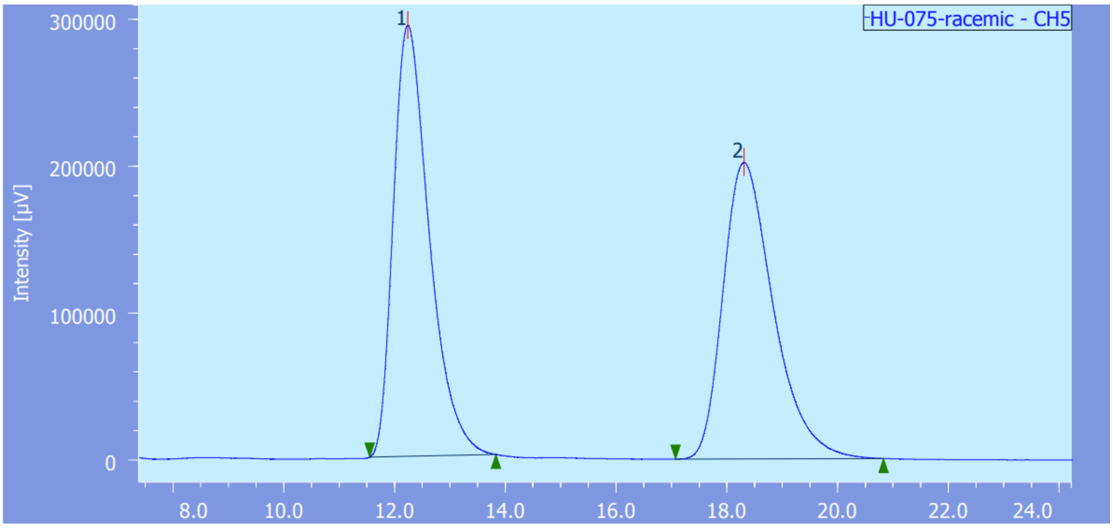

Decision

#	Peak Name	CH	tR [min]	Area [µV·sec]	Height [µV]	Area%	Height%	Quantity	NTP	Resolution	Symmetry Factor	Warning
1	Unknown	5	12.240	13197636	293274	50.840	59.231	N/A	1772	4.365	1.470	
2	Unknown	5	18.307	12761674	201862	49.160	40.769	N/A	2032	N/A	1.400	

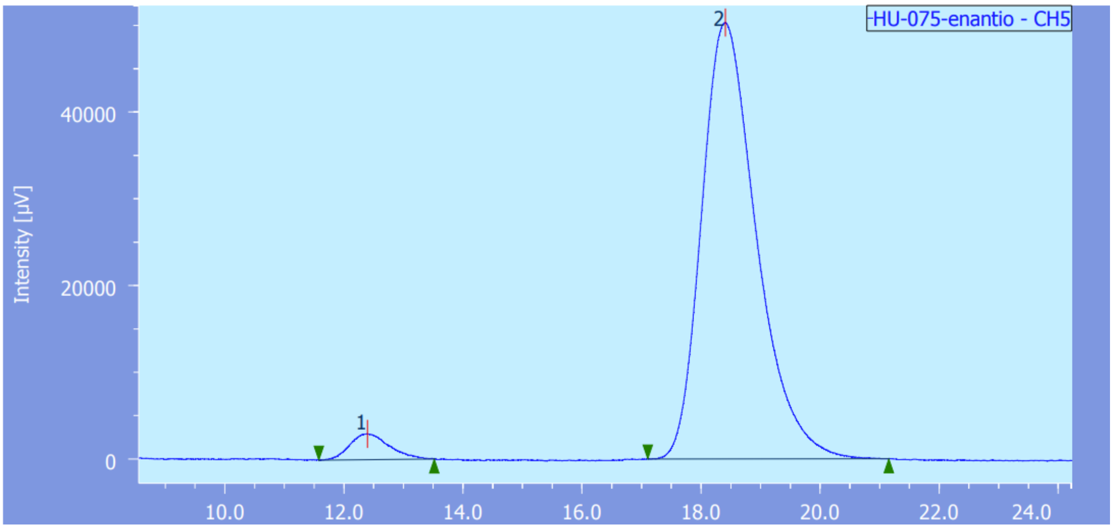

Decision

#	Peak Name	CH	tR [min]	Area [µV·sec]	Height [µV]	Area%	Height%	Quantity	NTP	Resolution	Symmetry Factor	Warning
1	Unknown	5	12.393	137383	2973	4.092	5.584	N/A	1646	4.204	1.267	
2	Unknown	5	18.403	3219970	50273	95.908	94.416	N/A	2003	N/A	1.340	

(*S*, *E*)-*N*-(4-(2-Fluorophenyl)-2-phenylbut-3-en-1-yl)-4-methylbenzenesulfonamide (24)

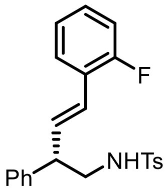

Prepared by **GP-A**. White solid, 64.1 mg, 81% yield. m.p. = 131-132 ºC; ^{1}H NMR (400 MHz, $CDCl_3$) δ 7.66 – 7.59 (m, 2H), 7.29 – 7.04 (m, 9H), 7.00 – 6.88 (m, 2H), 6.43 (d, *J* = 16.1 Hz, 1H), 6.20 (dd, *J* = 16.1, 8.1 Hz, 1H), 4.43 (t, *J* = 6.2 Hz, 1H), 3.49 (q, *J* = 7.7 Hz, 1H), 3.32 – 3.17 (m, 2H), 2.34 (s, 3H); ^{13}C NMR (101 MHz, $CDCl_3$) δ 160.1 (d, *J* = 250.2 Hz), 143.5, 140.0, 136.9, 132.0 (d, *J* = 5.0 Hz), 129.7, 129.0, 128.9 (d, *J* = 8.5 Hz), 127.6, 127.4 (d, *J* = 3.7 Hz), 127.3, 127.1, 124.8 (d, *J* = 3.3 Hz), 124.3 (d, *J* = 12.2 Hz), 124.0 (d, *J* = 3.5 Hz), 115.7 (d, *J* = 22.2 Hz), 49.1, 47.4, 21.5; ^{19}F NMR (377 MHz, $CDCl_3$) δ -117.87; IR (film): ν (cm^{-1}) 3297, 3265, 3029, 2920, 1597, 1576, 1488, 1454, 1423,

1319, 1312, 1293, 1230, 1151, 1090, 1078, 1032, 963, 906, 849, 820, 771, 752, 696, 672, 551, 522; HR-MS (ESI) m/z calcd for $C_{23}H_{23}FNO_2S$ [M+H$^+$] 396.1428, found 396.1432; $[\alpha]_D^{24.3}$ = -12.8 (c = 0.1, $CHCl_3$); HPLC conditions: OD-H column, hexane/2-propanol = 90/10, flow rate = 1.0 mL/min, λ = 254 nm, t_R = 12.6 min (minor), t_R = 17.3 min (major), 96:4 er.

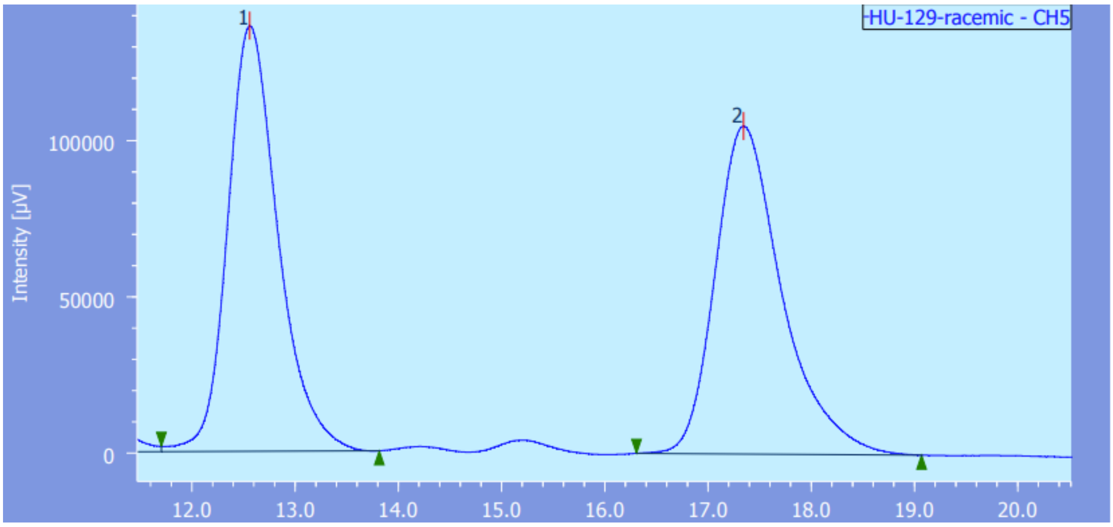

Decision

#	Peak Name	CH	tR [min]	Area [µV·sec]	Height [µV]	Area%	Height%	Quantity	NTP	Resolution	Symmetry Factor	Warning
1	Unknown	5	12.560	4633549	136073	49.505	56.482	N/A	3429	4.835	1.263	
2	Unknown	5	17.343	4726176	104839	50.495	43.518	N/A	3797	N/A	1.386	

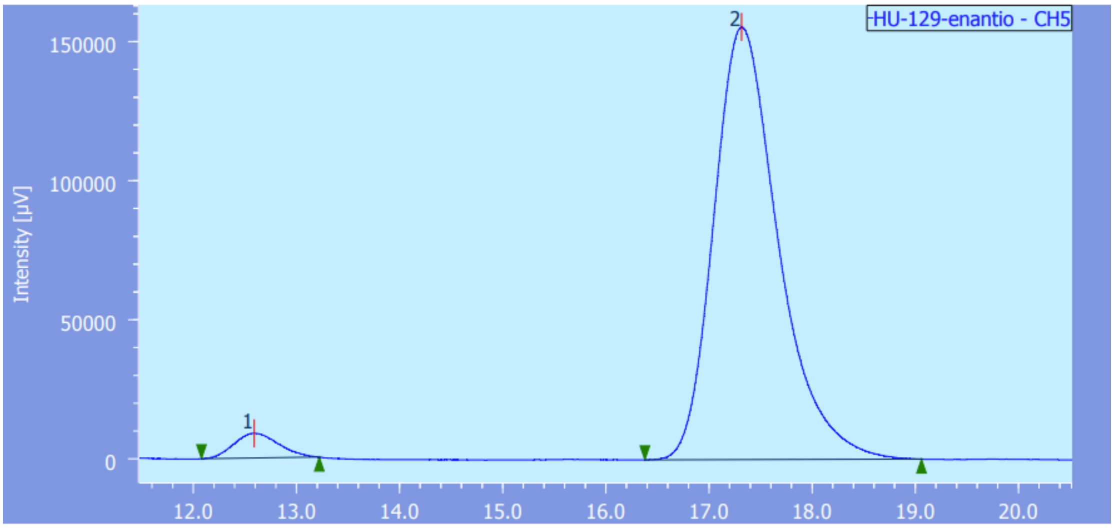

Decision

#	Peak Name	CH	tR [min]	Area [µV·sec]	Height [µV]	Area%	Height%	Quantity	NTP	Resolution	Symmetry Factor	Warning
1	Unknown	5	12.590	268739	8884	3.824	5.407	N/A	3790	4.924	1.143	
2	Unknown	5	17.313	6758143	155422	96.176	94.593	N/A	3924	N/A	1.346	

(*S*, *E*)-*N*-(4-(3-Fluorophenyl)-2-phenylbut-3-en-1-yl)-4-methylbenzenesulfonamide (25)

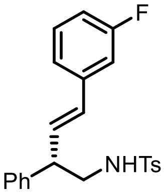

Prepared by **GP-A**. White solid, 58.4 mg, 74% yield. m.p. = 132-134 °C; ^{1}H NMR (400 MHz, $CDCl_3$) δ 7.65 – 7.59 (m, 2H), 7.27 – 7.11 (m, 6H), 7.07 – 7.02 (m, 2H), 6.96 (d, J = 7.7 Hz, 1H), 6.91 – 6.86 (m, 1H), 6.82 (tdd, J = 8.5, 2.6, 0.8 Hz, 1H), 6.25 (d, J = 15.9 Hz, 1H), 6.11 (dd, J = 15.9, 7.8 Hz, 1H), 4.47 (t, J = 6.2 Hz, 1H), 3.50 (q, J = 7.5 Hz, 1H), 3.30 – 3.16 (m, 2H), 2.34 (s, 3H); ^{13}C NMR (101 MHz, $CDCl_3$) δ 163.0 (d, J = 246.3 Hz), 143.5, 139.9, 138.9 (d, J = 7.7 Hz), 136.8, 131.1 (d, J = 2.5 Hz), 130.8, 129.9 (d, J = 8.4 Hz), 129.7, 129.0, 127.6, 127.4, 127.1, 122.2(d, J = 2.7 Hz), 114.4 (d,

J = 21.4 Hz),112.7 (d, J = 21.9 Hz), 48.6, 47.4, 21.5; ^{19}F NMR (377 MHz, $CDCl_3$) δ -113.42; IR (film): ν (cm^{-1}) 3295, 3265, 3031, 2924, 1607, 1598, 1579, 1485, 1453, 1444, 1421, 1319, 1310, 1250, 1150, 1093, 1074, 957, 906, 869, 853, 819, 777, 761, 696, 668, 552, 521; HR-MS (ESI) m/z calcd for $C_{23}H_{23}FNO_2S$ [$M+H^+$] 396.1428, found 396.1431; $[\alpha]_D^{24.3}$ = -10.6 (c = 0.1, $CHCl_3$); HPLC conditions: OD-H column, hexane/2-propanol = 90/10, flow rate = 1.0 mL/min, λ = 254 nm, t_R = 14.4 min (minor), t_R = 19.7 min (major), 95:5 er.

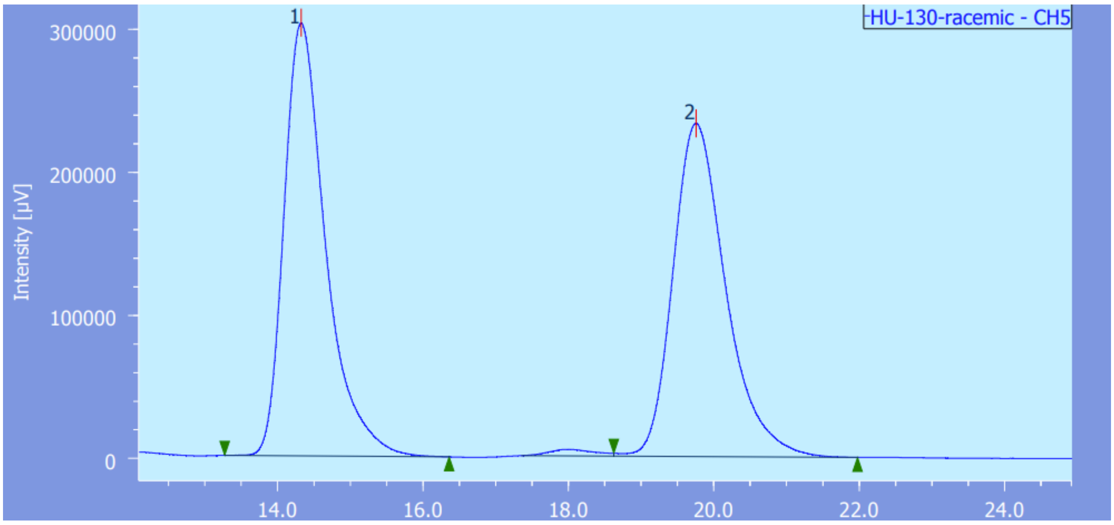

Decision

#	Peak Name	CH	tR [min]	Area [µV·sec]	Height [µV]	Area%	Height%	Quantity	NTP	Resolution	Symmetry Factor	Warning
1	Unknown	5	14.323	12062925	302763	50.568	56.522	N/A	3330	4.780	1.496	
2	Unknown	5	19.753	11792105	232892	49.432	43.478	N/A	3779	N/A	1.330	

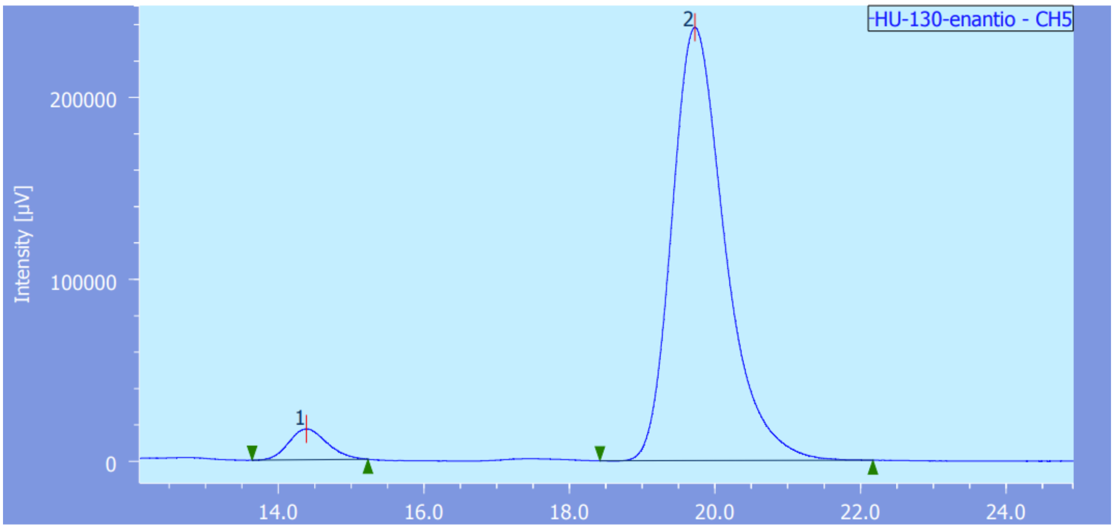

Decision

#	Peak Name	CH	tR [min]	Area [µV·sec]	Height [µV]	Area%	Height%	Quantity	NTP	Resolution	Symmetry Factor	Warning
1	Unknown	5	14.383	625284	16972	4.931	6.658	N/A	3510	4.743	1.167	
2	Unknown	5	19.723	12055035	237952	95.069	93.342	N/A	3760	N/A	1.344	

(*S*, *E*)-4-Methyl-*N*-(4-(naphthalen-2-yl)-2-phenylbut-3-en-1-yl)benzenesulfonamide (26)

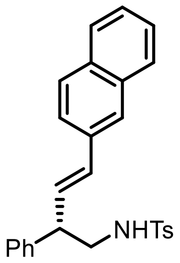

Prepared by **GP-A**. White solid, 64.5 mg, 75% yield. m.p. = 166-167 °C; ^{1}H NMR (400 MHz, $CDCl_3$) δ 7.85 – 7.71 (m, 5H), 7.67 (s, 1H), 7.56 – 7.43 (m, 3H), 7.39 – 7.26 (m, 5H), 7.23 – 7.16 (m, 2H), 6.57 (d, *J* = 15.9 Hz, 1H), 6.33 (dd, *J* = 15.9, 7.9 Hz, 1H), 4.52 (t, *J* = 6.2 Hz, 1H), 3.66 (q, *J* = 7.6 Hz, 1H), 3.47 – 3.31 (m, 2H), 2.44 (s, 3H); ^{13}C NMR (101 MHz, $CDCl_3$) δ 143.5, 140.2, 136.9, 134.0, 133.5, 133.0, 132.3, 129.7, 129.6, 129.0, 128.2, 127.9, 127.7, 127.6, 127.3, 127.1, 126.3, 125.9, 123.4, 48.8, 47.5, 21.5; IR (film): ν (cm^{-1}) 3262, 3027, 2919, 2358, 2338, 1596, 1507, 1494, 1452, 1428, 1327, 1306, 1288, 1149, 1090, 1019, 961, 860, 838, 824, 811, 755, 742, 695, 659, 552, 541; HR-MS (ESI) m/z calcd for $C_{27}H_{26}NO_2S$ [M+H$^+$] 428.1679, found 428.1683; $[\alpha]_D^{24.5}$ = -32.6 (c = 0.1, $CHCl_3$); HPLC conditions: OD-H column, hexane/2-propanol = 90/10, flow rate = 1.0 mL/min, λ = 254 nm, t_R = 28.4 min (minor), t_R = 50.4 min (major), 95:5 er.

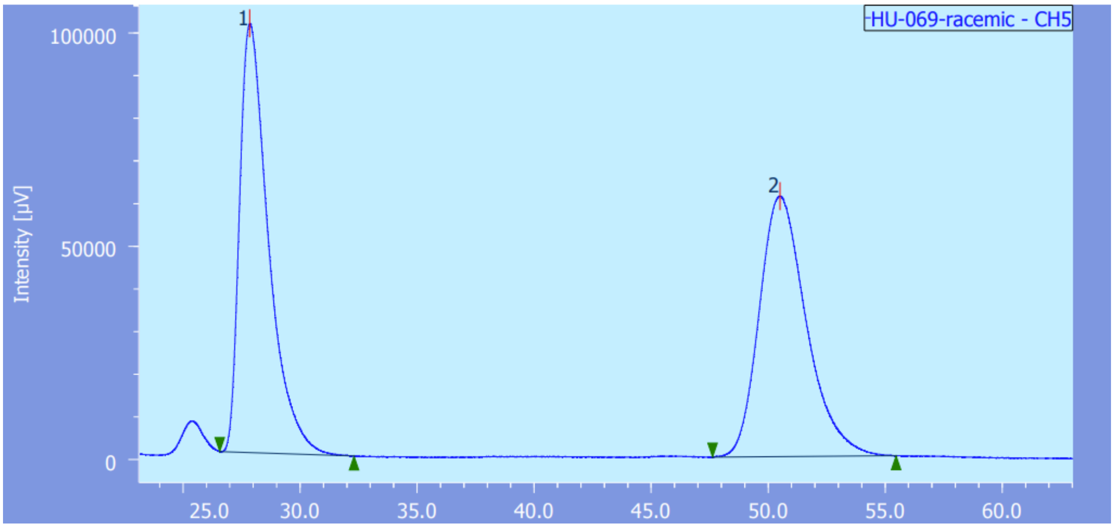

Decision

#	Peak Name	CH	tR [min]	Area [µV·sec]	Height [µV]	Area%	Height%	Quantity	NTP	Resolution	Symmetry Factor	Warning
1	Unknown	5	27.850	8619167	100536	50.230	62.226	N/A	2654	7.887	1.786	
2	Unknown	5	50.500	8540203	61030	49.770	37.774	N/A	3155	N/A	1.293	

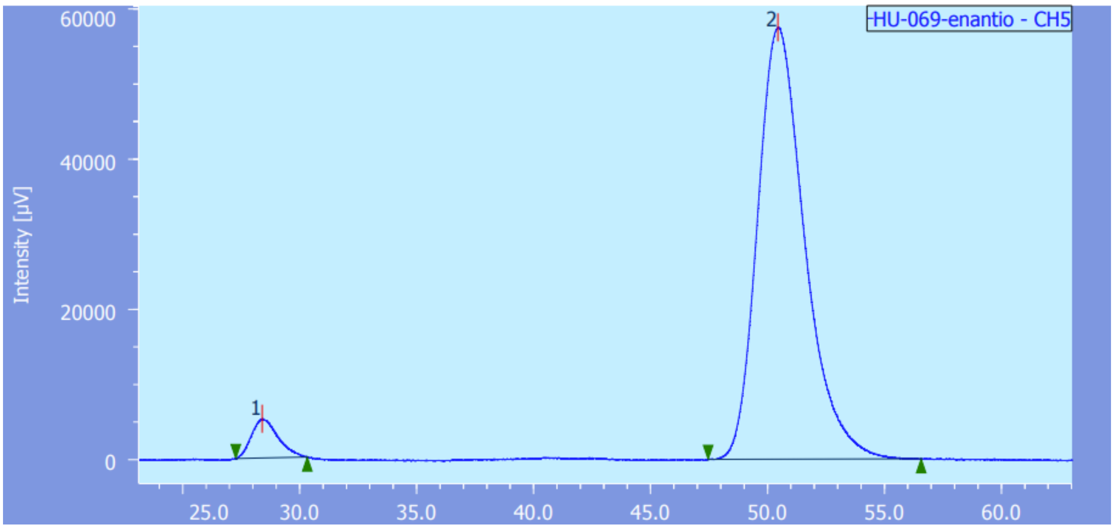

Decision

#	Peak Name	CH	tR [min]	Area [µV·sec]	Height [µV]	Area%	Height%	Quantity	NTP	Resolution	Symmetry Factor	Warning
1	Unknown	5	28.397	409137	5214	4.841	8.322	N/A	3008	7.823	1.352	
2	Unknown	5	50.433	8041807	57435	95.159	91.678	N/A	3179	N/A	1.322	

(*S*, *E*)-*N*-(4-(6-Methoxypyridin-3-yl)-2-phenylbut-3-en-1-yl)-4-methylbenzenesulfonamide

(27)

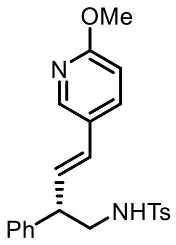

Prepared by **GP-A**. White solid, 71.3 mg, 87% yield. m.p. = 97-98 °C; ^{1}H NMR (400 MHz, $CDCl_3$) δ 7.91 (d, *J* = 2.4 Hz, 1H), 7.67 – 7.58 (m, 2H), 7.48 (dd, *J* = 8.7, 2.5 Hz, 1H), 7.26 – 7.12 (m, 5H), 7.08 – 7.00 (m, 2H), 6.58 (d, *J* = 8.7 Hz, 1H), 6.21 (d, *J* = 15.9 Hz, 1H), 5.99 (dd, *J* = 15.9, 7.9 Hz, 1H), 4.58 (t, *J* = 6.2 Hz, 1H), 3.83 (s, 3H), 3.49 (q, *J* = 7.6 Hz, 1H), 3.30 – 3.15 (m, 2H), 2.34 (s, 3H); ^{13}C NMR (101 MHz, $CDCl_3$) δ 163.6, 145.4, 143.5, 140.2, 136.9, 135.4, 129.7, 129.0, 128.6, 128.3, 127.6, 127.3, 127.1, 125.8, 110.8, 53.5, 48.8, 47.5, 21.5; IR (film): ν (cm^{-1}) 3271, 3027, 2950, 2852, 1601, 1564, 1492, 1451, 1435, 1383, 1325, 1297, 1285, 1257, 1248, 1154, 1130, 1090, 1078, 1064, 1021, 965, 863, 839, 807, 766, 755, 701, 664, 554, 526; HR-MS (ESI) m/z calcd for $C_{23}H_{25}N_2O_3S$ [M+H$^+$] 409.1580, found 409.1582; $[\alpha]_D^{24.5}$ = -15.8 (c = 0.1, $CHCl_3$); HPLC conditions: OD-H column, hexane/2-propanol = 90/10, flow rate = 1.0 mL/min, λ = 254 nm, t_R = 24.1 min (minor), t_R = 34.9 min (major), 95:5 er.

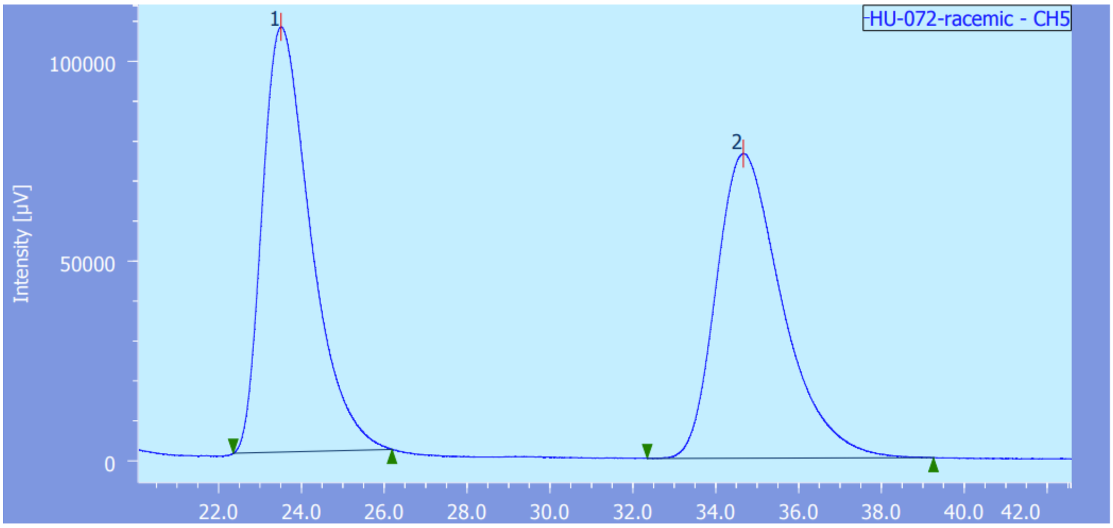

Decision

#	Peak Name	CH	tR [min]	Area [µV·sec]	Height [µV]	Area%	Height%	Quantity	NTP	Resolution	Symmetry Factor	Warning
1	Unknown	5	23.507	8641236	106326	50.561	58.254	N/A	2006	4.526	1.548	
2	Unknown	5	34.663	8449614	76196	49.439	41.746	N/A	2378	N/A	1.422	

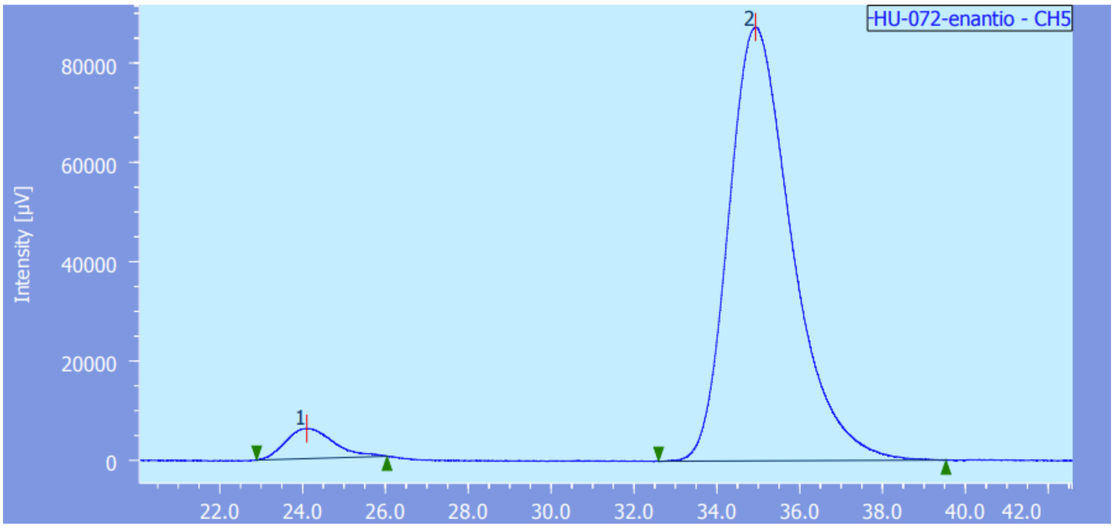

Decision

#	Peak Name	CH	tR [min]	Area [µV·sec]	Height [µV]	Area%	Height%	Quantity	NTP	Resolution	Symmetry Factor	Warning
1	Unknown	5	24.097	488933	6075	5.024	6.511	N/A	2083	4.507	1.346	
2	Unknown	5	34.930	9242169	87223	94.976	93.489	N/A	2662	N/A	1.366	

(*S*, *E*)-*N*-(4-(2-Methoxypyrimidin-5-yl)-2-phenylbut-3-en-1-yl)-4-methylbenzenesulfonamide (28)

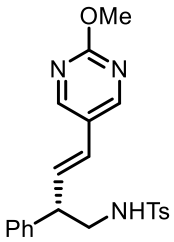

Prepared by **GP-A**. White solid, 62.3 mg, 76% yield. m.p. = 72-73 °C; ^{1}H NMR (400 MHz, $CDCl_3$) δ 8.33 (s, 2H), 7.67 – 7.59 (m, 2H), 7.27 – 7.15 (m, 5H), 7.09 – 7.02 (m, 2H), 6.20 – 6.07 (m, 2H), 4.66 (t, *J* = 6.3 Hz, 1H), 3.92 (s, 3H), 3.54 (q, *J* = 7.2, 1H), 3.33 – 3.17 (m, 2H), 2.35 (s, 3H); ^{13}C NMR (101 MHz, $CDCl_3$) δ 164.6, 156.6, 143.5, 139.7, 136.9, 131.2, 129.7, 129.1, 127.6, 127.5, 127.0, 124.8, 124.3, 55.0, 49.0, 47.4, 21.5; IR (film): ν (cm^{-1}) 3282, 3027, 2924, 2858, 1661, 1594, 1555, 1494, 1471, 1409, 1323, 1289, 1154, 1091, 1047, 1029, 965, 813, 796, 760, 701, 659, 550; HR-MS (ESI) m/z calcd for $C_{22}H_{24}N_3O_3S$ [M+H$^+$] 410.1533, found 410.1536; $[\alpha]_D^{24.4}$ = -19.0 (c = 0.1, $CHCl_3$); HPLC conditions: OD-H column, hexane/2-propanol = 85/15, flow rate = 1.0 mL/min, λ = 254 nm, t_R = 43.0 min (major), t_R = 66.2 min (minor), 95:5 er.

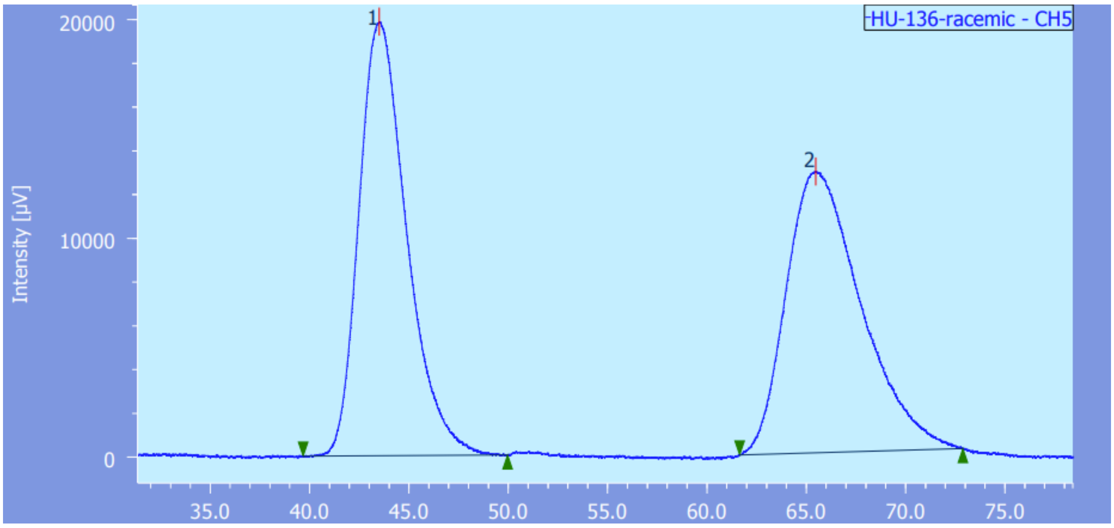

Decision

#	Peak Name	CH	tR [min]	Area [µV·sec]	Height [µV]	Area%	Height%	Quantity	NTP	Resolution	Symmetry Factor	Warning
1	Unknown	5	43.507	3316164	19832	49.634	60.680	N/A	1627	3.944	1.349	
2	Unknown	5	65.460	3365102	12851	50.366	39.320	N/A	1462	N/A	1.449	

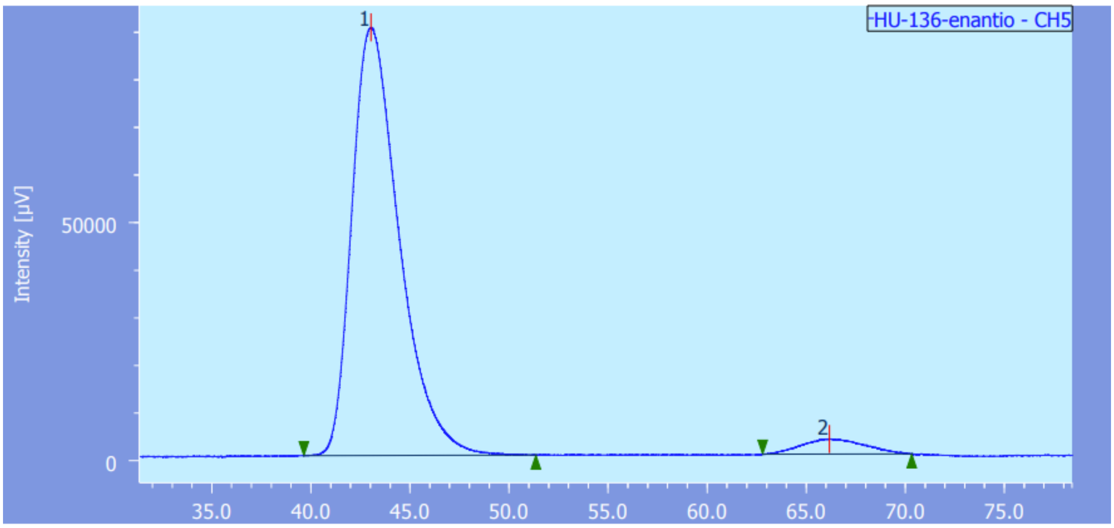

Decision

#	Peak Name	CH	tR [min]	Area [μV·sec]	Height [μV]	Area%	Height%	Quantity	NTP	Resolution	Symmetry Factor	Warning
1	Unknown	5	43.023	14953413	89885	95.461	96.572	N/A	1623	4.417	1.468	
2	Unknown	5	66.163	710943	3191	4.539	3.428	N/A	1803	N/A	1.146	

Benzyl (*S*, *E*)-(2, 4-diphenylbut-3-en-1-yl)carbamate (29)

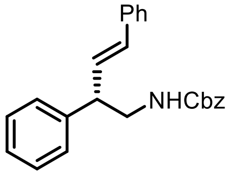

Prepared by **GP-A**. White solid, 54.2 mg, 76% yield. m.p. = 63-64 °C; ^{1}H NMR (400 MHz, $CDCl_3$) δ 7.30 – 7.10 (m, 15H), 6.38 (d, *J* = 15.9 Hz, 1H), 6.23 (dd, *J* = 15.9, 7.3 Hz, 1H), 4.99 (s, 2H), 4.80 – 4.62 (m, 1H), 3.68 – 3.40 (m, 3H); ^{13}C NMR (101 MHz, $CDCl_3$) δ 156.2, 141.1, 136.9, 136.5, 131.6, 130.2, 128.8, 128.5, 128.5, 128.1, 127.8, 127.5, 127.0, 126.2, 66.6, 49.2, 45.7; IR (film): ν (cm^{-1}) 3348, 3058, 3029, 2932, 2359, 2342, 1689, 1601, 1533, 1494, 1451, 1434, 1362, 1268, 1238, 1145, 1040, 1027, 1008, 962, 910, 767, 756, 734, 693, 640, 614, 545; HR-MS (ESI) m/z calcd for $C_{24}H_{24}NO_2$ [M+H$^+$] 358.1802, found 358.1805; $[\alpha]_D^{24.5}$ = -4.6 (c = 0.1, $CHCl_3$); HPLC conditions: OD-H column, hexane/2-propanol = 90/10, flow rate = 1.0 mL/min, λ = 254 nm, t_R = 19.8 min (minor), t_R = 25.9 min (major), 97:3 er.

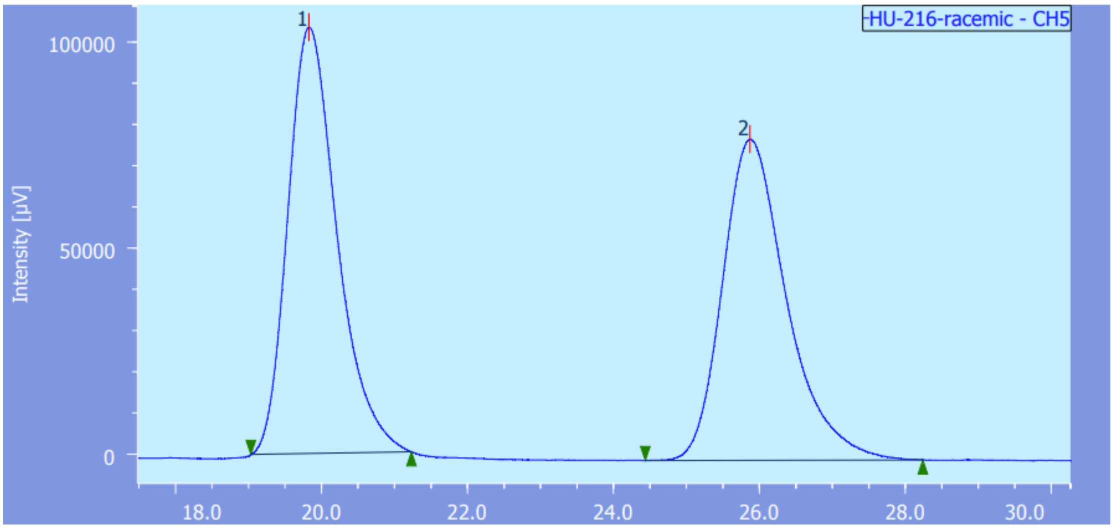

Decision

#	Peak Name	CH	tR [min]	Area [μV·sec]	Height [μV]	Area%	Height%	Quantity	NTP	Resolution	Symmetry Factor	Warning
1	Unknown	5	19.827	4847322	103340	50.490	57.036	N/A	4316	4.401	1.333	
2	Unknown	5	25.867	4753316	77844	49.510	42.964	N/A	4484	N/A	1.354	

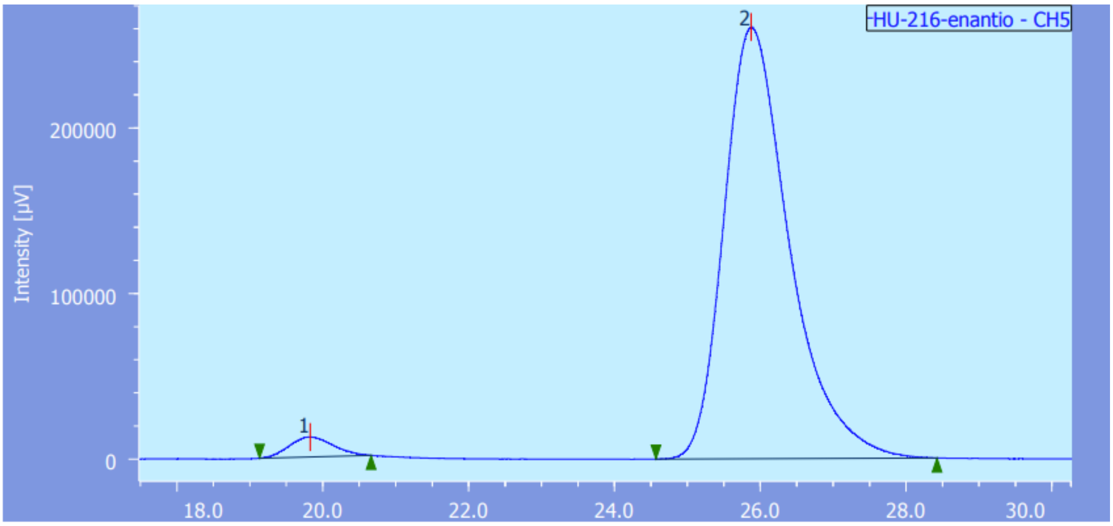

Decision

#	Peak Name	CH	tR [min]	Area [µV·sec]	Height [µV]	Area%	Height%	Quantity	NTP	Resolution	Symmetry Factor	Warning
1	Unknown	5	19.827	504879	12023	3.013	4.411	N/A	4917	4.484	1.116	
2	Unknown	5	25.873	16251656	260566	96.987	95.589	N/A	4328	N/A	1.421	

Benzyl (*S*, *E*)-(4-cyclohexyl-2-phenylbut-3-en-1-yl)carbamate (30)

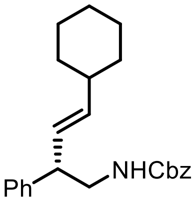

Prepared by **GP-A**. White solid, 46.7 mg, 64% yield. m.p. = 70-71 °C; ^{1}H NMR (400 MHz, $CDCl_3$) δ 7.35 – 7.01 (m, 10H), 5.50 – 5.32 (m, 2H), 5.00 (s, 2H), 4.74 – 4.50 (m, 1H), 3.55 – 3.13 (m, 3H), 1.94 – 1.77 (m, 1H), 1.68 – 1.51 (m, 5H), 1.25 – 0.88 (m, 5H); ^{13}C NMR (101 MHz, $CDCl_3$) δ 156.2, 141.9, 138.8, 136.6, 128.7, 128.5, 128.1, 127.6, 127.5, 126.7, 66.6, 48.8, 45.8, 40.6, 33.0, 32.9, 26.1, 25.98, 25.97; IR (film): ν (cm^{-1}) 3356, 3026, 2918, 2844, 1695, 1538, 1492, 1448, 1441, 1305, 1286, 1269, 1241, 1146, 1039, 1028, 1010, 968, 762, 749, 730, 694, 647, 547; HR-MS (ESI) m/z calcd for $C_{24}H_{30}NO_2$ [M+H$^+$] 364.2271, found 364.2275; $[\alpha]_D^{24.6}$ = -4.7 (c = 0.1, $CHCl_3$); HPLC conditions: OD-H column, hexane/2-propanol =97/3, flow rate = 1.0 mL/min, λ = 210 nm, t_R = 26.3 min (minor), t_R = 42.8 min (major), 98:2 er.

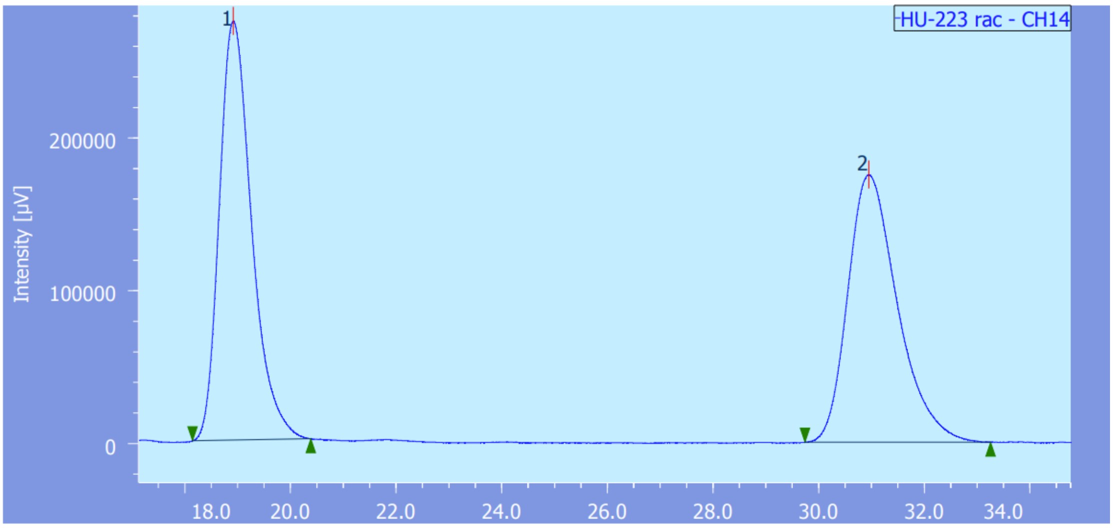

Decision

#	Peak Name	CH	tR [min]	Area [µV·sec]	Height [µV]	Area%	Height%	Quantity	NTP	Resolution	Symmetry Factor	Warning
1	Unknown	14	18.917	11647939	274245	50.500	61.038	N/A	4808	8.709	1.346	
2	Unknown	14	30.943	11417351	175060	49.500	38.962	N/A	5440	N/A	1.349	

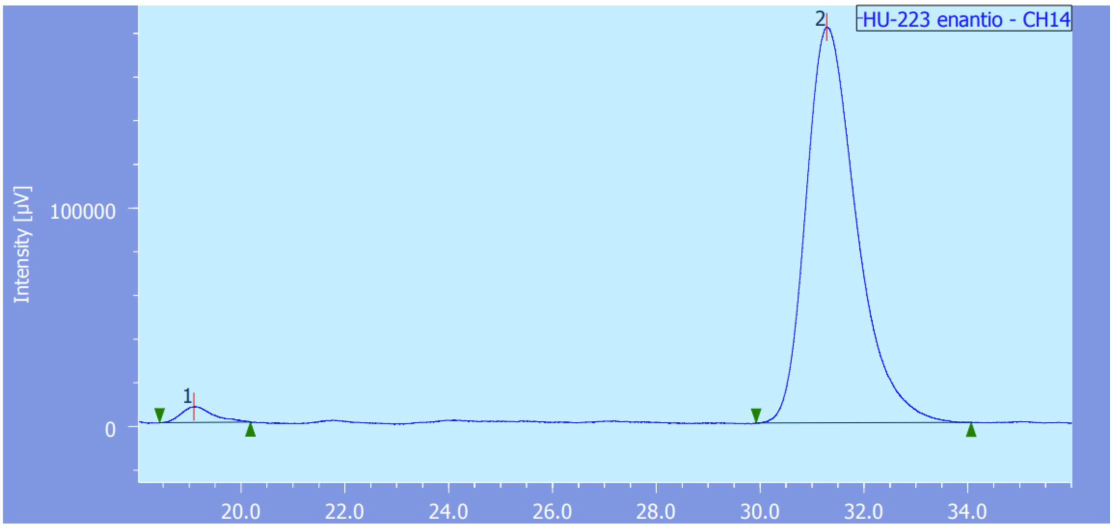

Decision

#	Peak Name	CH	tR [min]	Area [µV·sec]	Height [µV]	Area%	Height%	Quantity	NTP	Resolution	Symmetry Factor	Warning
1	Unknown	14	19.093	310219	7215	2.467	3.831	N/A	5073	8.725	1.456	
2	Unknown	14	31.280	12265375	181134	97.533	96.169	N/A	5239	N/A	1.394	

(*S*, *E*)-6-(((Benzyloxy)carbonyl)amino)-5-phenylhex-3-en-1-yl benzoate (31)

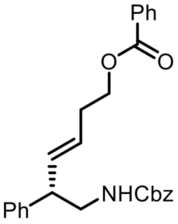

Prepared by **GP-A**. Colorless oil, 45.1 mg, 52% yield. ^{1}H NMR (400 MHz, $CDCl_3$) δ 7.94 – 7.86 (m, 2H), 7.50 – 7.40 (m, 1H), 7.36 – 7.03 (m, 12H), 5.64 (dd, J = 15.3, 5.8 Hz, 1H), 5.57 – 5.46 (m, 1H), 4.99 (s, 2H), 4.78– 4.55 (m, 1H), 4.26 (t, J = 6.5 Hz, 2H), 3.50 – 3.25 (m, 3H), 2.41 (q, J = 6.3 Hz, 2H); ^{13}C NMR (101 MHz, $CDCl_3$) δ 166.5, 156.2, 141.3, 136.5, 133.4, 132.8, 130.2, 129.5, 128.7, 128.5, 128.3, 128.1, 127.8, 127.6, 126.8, 66.6, 64.0, 48.8, 45.5, 32.2; IR (film): ν (cm^{-1}) 3427, 3357, 3061, 3031, 2955, 1713, 1601, 1515, 1495, 1452, 1314, 1270, 1252, 1176, 1111, 1070, 1026, 968, 773, 752, 712, 698, 675; HR-MS (ESI) m/z calcd for $C_{27}H_{28}NO_4$ $[M+H^+]$ 430.2013, found 430.2016; $[\alpha]_D^{24.4}$ = -4.9 (c = 0.1, $CHCl_3$); HPLC conditions: OD-H column, hexane/2-propanol =95/5, flow rate = 0.5 mL/min, λ = 220 nm, t_R = 120.9 min (major), t_R = 138.5 min (minor), 98:2 er.

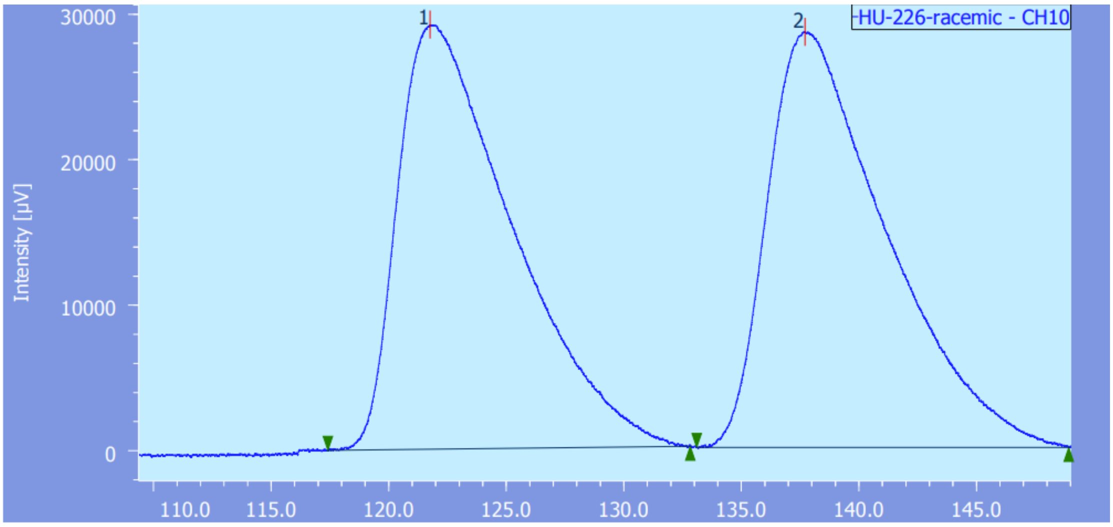

Decision

#	Peak Name	CH	tR [min]	Area [µV·sec]	Height [µV]	Area%	Height%	Quantity	NTP	Resolution	Symmetry Factor	Warning
1	Unknown	10	121.757	9863410	29153	49.539	50.528	N/A	2996	1.770	2.064	
2	Unknown	10	137.693	10046947	28543	50.461	49.472	N/A	3616	N/A	1.854	

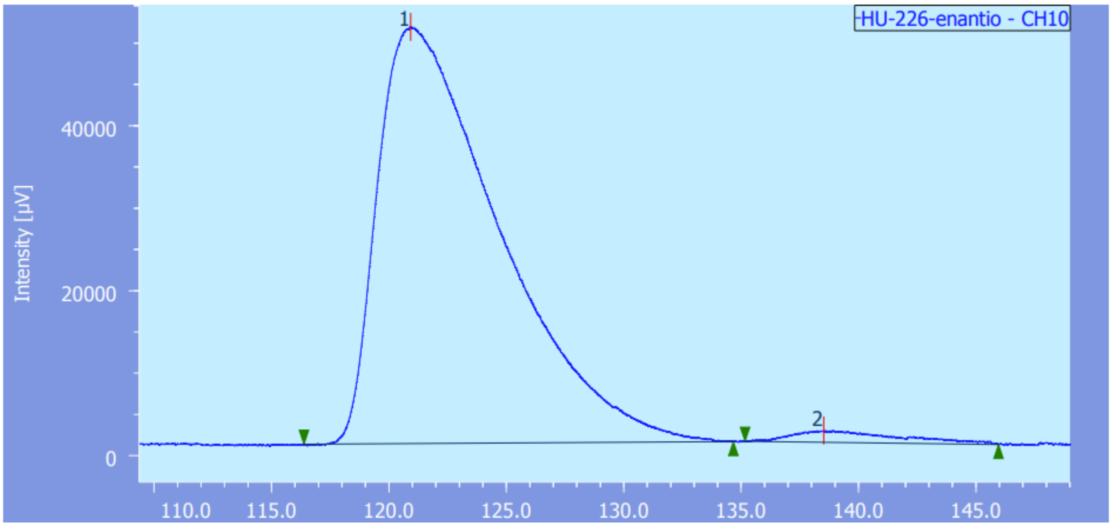

Decision

#	Peak Name	CH	tR [min]	Area [μV·sec]	Height [μV]	Area%	Height%	Quantity	NTP	Resolution	Symmetry Factor	Warning
1	Unknown	10	120.927	18136668	50445	97.599	97.261	N/A	2698	1.867	2.317	
2	Unknown	10	138.513	446196	1420	2.401	2.739	N/A	3348	N/A	1.695	

Benzyl (*S*, *E*)-(2-phenyl-4-(trimethylsilyl)but-3-en-1-yl)carbamate (32)

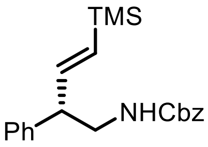

Prepared by **GP-A**. Colorless oil, 30.0 mg, 42% yield. ^{1}H NMR (400 MHz, $CDCl_3$) δ 7.35 – 7.23 (m, 7H), 7.22 – 7.16 (m, 1H), 7.16 – 7.06 (m, 2H), 6.06 (dd, J = 18.7, 6.6 Hz, 1H), 5.71 (dd, J = 18.7, 1.1 Hz, 1H), 5.03 (s, 2H), 4.73 – 7.52 (m, 1H), 3.63 – 3.32 (m, 3H), -0.00 (s, 9H); ^{13}C NMR (101 MHz, $CDCl_3$) δ 156.2, 145.9, 141.0, 136.5, 132.2, 128.8, 128.5, 128.1, 127.9, 126.9, 66.6, 52.1, 45.3, -1.3; IR (film): ν (cm^{-1}) 3389, 2953, 2933, 1715, 1698, 1613, 1600, 1517, 1492, 1453, 1434, 1273, 1249, 1224, 1194, 1134, 1000, 985, 871, 838, 762, 732, 701, 693; HR-MS (ESI) m/z calcd for $C_{21}H_{28}NO_2Si$ [M+H$^+$] 354.1884, found 354.1885; $[\alpha]_D^{24.5}$ = -5.9 (c = 0.1, $CHCl_3$); HPLC conditions: OD-H column, hexane/2-propanol =95/5, flow rate = 0.5 mL/min, λ = 220 nm, t_R = 19.8 min (minor), t_R = 28.2 min (major), 98:2 er.

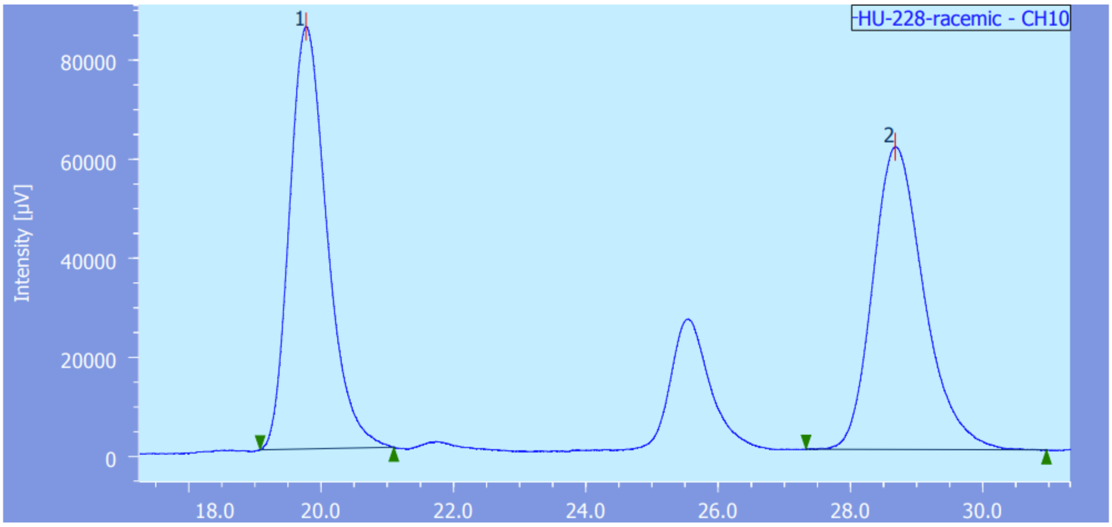

Decision

#	Peak Name	CH	tR [min]	Area [μV·sec]	Height [μV]	Area%	Height%	Quantity	NTP	Resolution	Symmetry Factor	Warning
1	Unknown	10	19.773	3355194	85180	50.443	58.237	N/A	6017	7.432	1.284	
2	Unknown	10	28.673	3296283	61083	49.557	41.763	N/A	6890	N/A	1.279	

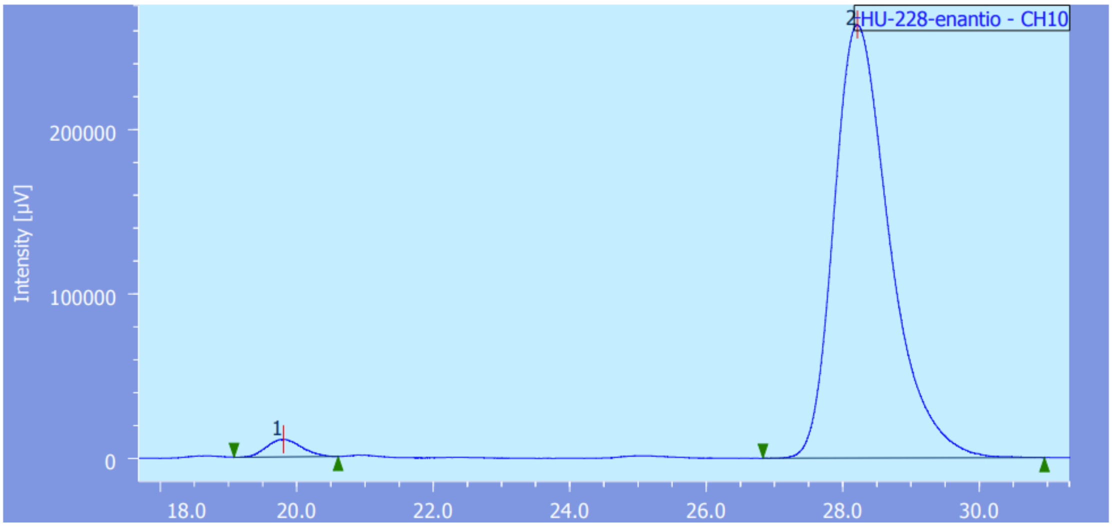

Decision

#	Peak Name	CH	tR [min]	Area [μV·sec]	Height [μV]	Area%	Height%	Quantity	NTP	Resolution	Symmetry Factor	Warning
1	Unknown	10	19.807	381583	10604	2.491	3.870	N/A	6651	6.973	1.121	
2	Unknown	10	28.210	14937866	263386	97.509	96.130	N/A	6095	N/A	1.396	

Benzyl ((S, 3*E*, 5*E*)-2,6-diphenylhexa-3,5-dien-1-yl)carbamate (33)

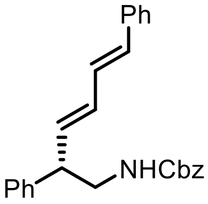

Prepared by **GP-A**. Colorless oil, 49.1 mg, 64% yield. ^{1}H NMR (400 MHz, $CDCl_3$) δ 7.30 – 7.10 (m, 15H), 6.66 (dd, J = 15.6, 10.4 Hz, 1H), 6.40 (d, J = 15.7 Hz, 1H), 6.18 (dd, J = 15.2, 10.4 Hz, 1H), 5.84 (dd, J = 15.2, 6.9 Hz, 1H), 5.00 (s, 2H), 4.76 – 4.60 (m, 1H), 3.57 – 3.30 (m, 3H); ^{13}C NMR (101 MHz, $CDCl_3$) δ 156.2, 141.1, 137.2, 136.5, 134.4, 132.2, 132.1, 128.8, 128.6, 128.5, 128.1, 128.1, 127.8, 127.5, 127.0, 126.3, 66.7, 49.0, 45.6; IR (film): ν (cm^{-1}) 3422, 3351, 3060, 3029, 1697, 1600, 1516, 1494, 1453, 1410, 1353, 1330, 1245, 1135, 1027, 972, 910, 749, 736, 694; HR-MS (ESI) m/z calcd for $C_{26}H_{26}NO_2$ [M+H^{+}] 384.1958, found 384.1955; $[\alpha]_D^{24.4}$ = -6.4 (c = 0.1, $CHCl_3$); HPLC conditions: OD-H column, hexane/2-propanol =90/10, flow rate = 1.0 mL/min, λ = 300 nm, t_R = 25.2 min (minor), t_R = 31.7 min (major), 90:10 er.

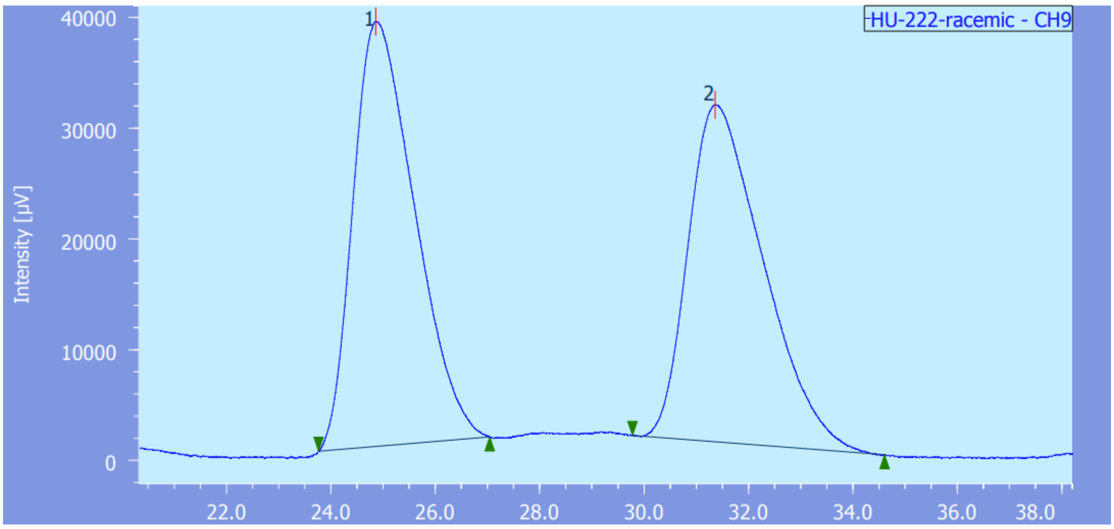

Decision

#	Peak Name	CH	tR [min]	Area [μV·sec]	Height [μV]	Area%	Height%	Quantity	NTP	Resolution	Symmetry Factor	Warning
1	Unknown	9	24.860	3143254	38321	50.704	55.759	N/A	2021	2.657	1.452	
2	Unknown	9	31.360	3056006	30405	49.296	44.241	N/A	2168	N/A	1.522	

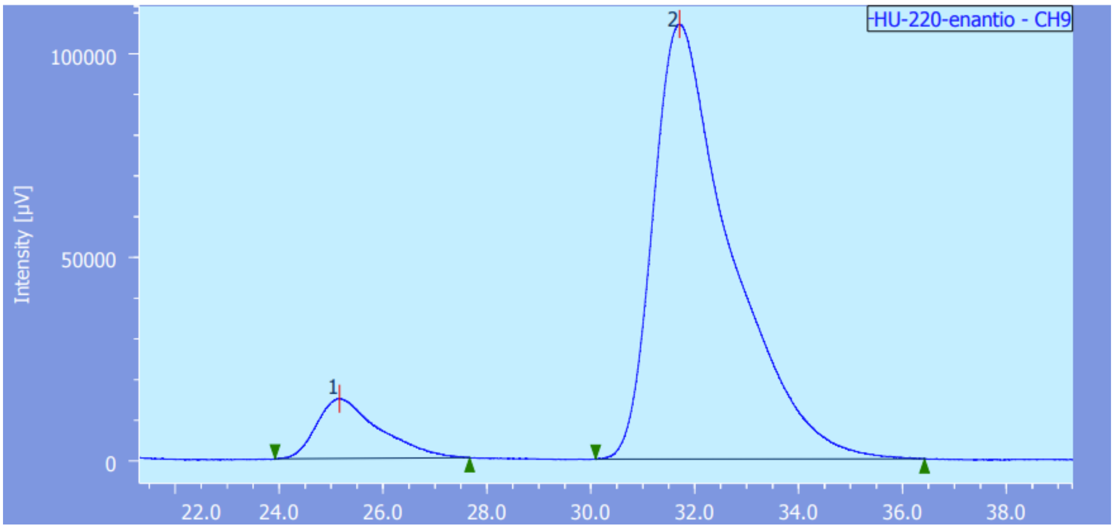

Decision

#	Peak Name	CH	tR [min]	Area [µV·sec]	Height [µV]	Area%	Height%	Quantity	NTP	Resolution	Symmetry Factor	Warning
1	Unknown	9	25.163	1223106	14642	9.896	12.063	N/A	2302	2.835	1.583	
2	Unknown	9	31.703	11136903	106731	90.104	87.937	N/A	2515	N/A	1.800	

(*R*)-*N*-(2-(1H-Inden-2-yl)-2-phenylethyl)-4-methylbenzenesulfonamide (34)

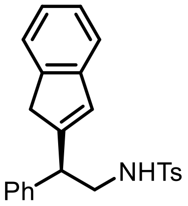

Prepared by **GP-B**. White solid, 38 mg, 49% yield. m.p. = 162-163 ºC; ^{1}H NMR (400 MHz, $CDCl_3$) δ 7.64 – 7.57 (m, 2H), 7.22 – 7.11 (m, 8H), 7.07 – 6.98 (m, 3H), 6.48 (d, J = 0.6 Hz, 1H), 4.45 (t, J = 6.2 Hz, 1H), 3.79 (t, J = 7.6 Hz, 1H), 3.50 (dt, J = 12.5, 7.1 Hz, 1H), 3.30 (ddd, J = 12.5, 8.0, 5.5 Hz, 1H), 3.11 – 2.96 (m, 2H), 2.34 (s, 3H); ^{13}C NMR (101 MHz, $CDCl_3$) δ 148.6, 144.3, 143.5, 142.8, 140.1, 136.7, 129.7, 128.9, 127.9, 127.6, 127.3, 127.1, 126.4, 124.5, 123.5, 120.6, 47.3, 46.69, 40.2, 21.5; IR (film): ν (cm^{-1}) 3260, 3027, 2872, 1599, 1493, 1454, 1426, 1391, 1331, 1306, 1288, 1245, 1228, 1213, 1156, 1093, 1075, 1023, 1016, 914, 880, 840, 813, 752, 719, 700, 675, 662, 553, 532; HR-MS (ESI) m/z calcd for $C_{24}H_{24}NO_2S$ [M+H$^+$] 390.1522, found 390.1524; $[\alpha]_D^{24.6}$ = 14.2 (c = 0.1, $CHCl_3$); HPLC conditions: OD-H column, hexane/2-propanol = 90/10, flow rate = 1.0 mL/min, λ = 254 nm, t_R = 20.1 min (major), t_R = 25.0 min (minor), 91:9 er.

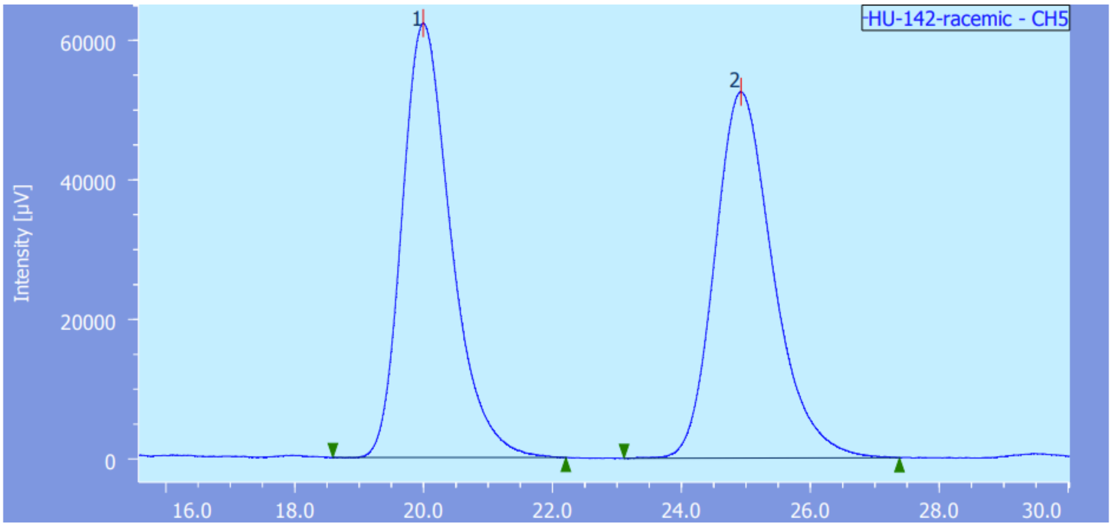

Decision

#	Peak Name	CH	tR [min]	Area [µV·sec]	Height [µV]	Area%	Height%	Quantity	NTP	Resolution	Symmetry Factor	Warning
1	Unknown	5	19.990	3287864	62172	49.871	54.239	N/A	3492	3.334	1.327	
2	Unknown	5	24.920	3304931	52454	50.129	45.761	N/A	3823	N/A	1.253	

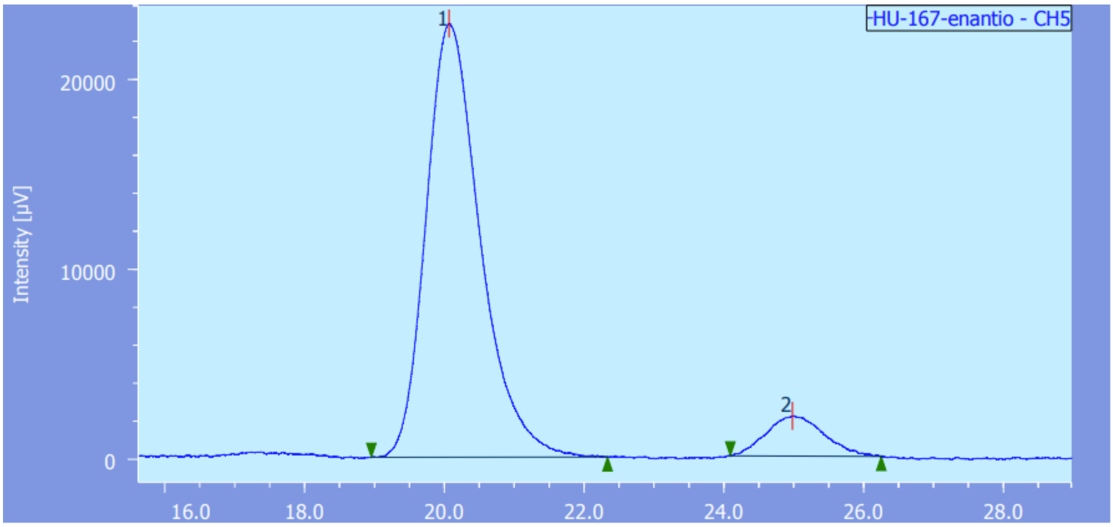

Decision

#	Peak Name	CH	tR [min]	Area [μV·sec]	Height [μV]	Area%	Height%	Quantity	NTP	Resolution	Symmetry Factor	Warning
1	Unknown	5	20.067	1227720	22812	90.972	91.537	N/A	3432	3.414	1.334	
2	Unknown	5	24.980	121842	2109	9.028	8.463	N/A	4346	N/A	1.170	

4-Methyl-*N*-((*S*, E)-2-((8*R*, 9*S*, 13*S*, 14*S*)-13-methyl-17-oxo-7, 8, 9, 11, 12, 13, 14, 15, 16, 17-decahydro-6H-cyclopenta[a]phenanthren-3-yl)-4-phenylbut-3-en-1-yl)benzenesulfonamide (35)

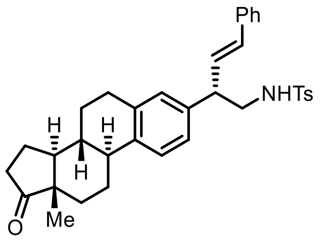

Prepared by **GP-A**. White solid, 66.6 mg, 60% yield. m.p. = 97-98 ^{o}C; ^{1}H NMR (400 MHz, $CDCl_3$) δ 7.66 – 7.60 (m, 2H), 7.23 – 7.10 (m, 8H), 6.83 (dd, *J* = 8.1, 1.6 Hz, 1H), 6.78 (s, 1H), 6.30 (d, *J* = 15.9 Hz, 1H), 6.08 (dd, *J* = 15.9, 8.0 Hz, 1H), 4.45 (t, *J* = 6.1 Hz, 1H), 3.43 (q, *J* = 7.7 Hz, 1H), 3.22 (t, *J* = 6.6 Hz, 2H), 2.84 – 2.72 (m, 2H), 2.47 – 2.28 (m, 5H), 2.23 – 2.14 (m, 1H), 2.10 – 1.84 (m, 4H), 1.61 – 1.29 (m, 6H), 0.83 (s, 3H); ^{13}C NMR (101 MHz, $CDCl_3$) δ 220.8, 143.4, 138.8, 137.6, 137.1, 136.9, 136.6, 131.9, 129.7, 129.4, 128.4, 128.3, 127.6, 127.1, 126.2, 125.9, 124.8, 50.4, 48.2, 47.9, 47.4, 44.2, 38.0, 35.8, 31.5, 29.4, 26.4, 25.6, 21.5, 13.8; IR (film): ν (cm^{-1}) 3281, 2925, 2855, 2358, 2342, 1734, 1599, 1496, 1451, 1435, 1405, 1326, 1306, 1289, 1258, 1157, 1090, 1056, 964, 909, 842, 812, 760, 751, 735, 694, 661, 578, 563, 551; HR-MS (ESI) m/z calcd for $C_{35}H_{40}NO_3S$ [M+H^{+}] 554.2723, found 554.2726; $[\alpha]_D^{24.4}$ = 60.7 (c = 0.1, $CHCl_3$).

4-Methyl-*N*-((*R*, E)-2-((8*R*, 9*S*, 13*S*, 14*S*)-13-methyl-17-oxo-7, 8, 9, 11, 12, 13, 14, 15, 16, 17-decahydro-6H-cyclopenta[a]phenanthren-3-yl)-4-phenylbut-3-en-1-yl)benzenesulfonamide (*epi*-35)

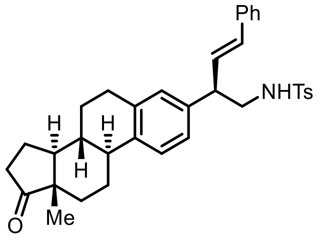

Prepared by **GP-A** by using **-L1** as ligand. Colorless oil, 70.0 mg, 63% yield. ^{1}H NMR (400 MHz, $CDCl_3$) δ7.67 – 7.60 (m, 2H), 7.26 – 7.20 (m, 6H), 7.18 – 7.12 (m, 2H), 6.84 (dd, *J* = 8.1, 1.6 Hz, 1H), 6.79 (s, 1H), 6.31 (d, *J* = 15.9 Hz, 1H), 6.08 (dd, *J* = 15.9, 8.0 Hz, 1H), 4.30 (t, *J* = 6.0 Hz, 1H), 3.43 (q, *J* = 7.6 Hz, 1H), 3.23 (t, *J* = 6.6 Hz, 2H), 2.86 – 2.74 (m, 2H), 2.48 – 2.30 (m, 5H), 2.26 – 2.16 (m, 1H), 2.12 – 1.86 (m, 4H), 1.61 – 1.32 (m, 6H), 0.84 (s, 3H); ^{13}C NMR (101 MHz, $CDCl_3$) δ 220.8, 143.5, 138.9, 137.6, 137.2, 136.9, 136.6, 132.0, 129.7, 129.4, 128.5, 128.3, 127.7, 127.2, 126.3, 126.0, 124.9, 50.4, 48.2, 48.0, 47.4, 44.3, 38.1, 35.8, 31.6, 29.4, 26.4, 25.6, 21.6, 13.8; $[\alpha]_D^{23.9}$ = -60.4 (c = 0.1, $CHCl_3$).

(*R*)-2, 5, 7, 8-Tetramethyl-2-((4R, 8R)-4, 8, 12-trimethyltridecyl)chroman-6-yl 4-((*S*, *E*)-1-((4-methylphenyl)sulfonamido)-4-phenylbut-3-en-2-yl)benzoate (36)

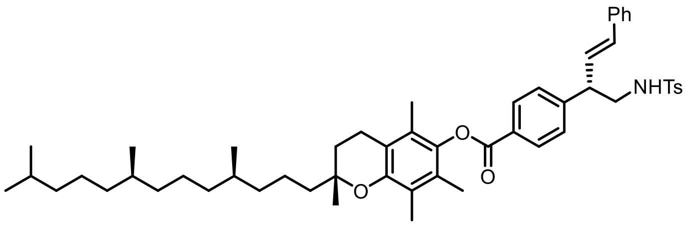

Prepared by **GP-A**. Colorless oil, 110.2 mg, 66% yield. ^{1}H NMR (400 MHz, $CDCl_3$) δ 8.20 – 7.99 (m, 2H), 7.72 – 7.53 (m, 2H), 7.29 – 7.10 (m, 9H), 6.35 (dd, *J* = 15.9, 0.8 Hz, 1H), 6.11 (dd, *J* = 15.9, 8.0 Hz, 1H), 4.47 (t, *J* = 6.2 Hz, 1H), 3.63 (q, *J* = 7.6 Hz, 1H), 3.29 (t, *J* = 6.8 Hz, 2H), 2.54 (t, *J* = 6.7 Hz, 2H), 2.36 (s, 3H), 2.04 (s, 3H), 1.97 (s, 3H), 1.93 (s, 3H), 1.82 – 1.62 (m, 2H), 1.52 – 1.13 (m, 18H), 1.09 – 0.93 (m, 6H), 0.83 – 0.74 (m, 12H); ^{13}C NMR (101 MHz, $CDCl_3$) δ 164.7, 149.5, 146.2, 143.6, 140.5, 136.8, 136.3, 133.0, 130.8, 129.8, 128.65, 128.58, 128.2, 128.0, 127.9, 127.1, 126.8, 126.3, 125.1, 123.1, 117.5, 75.1, 48.9, 47.3, 40.4, 39.6, 39.3, 37.4, 37.3, 32.8, 31.2, 31.0, 28.0, 24.8,

24.4, 24.2, 23.6, 22.7, 22.6, 21.5, 21.0, 20.6, 19.7, 19.6, 13.0, 12.2, 11.8; IR (film): ν (cm^{-1}) 3286, 2949, 2924, 2866, 2361, 1732, 1610, 1599, 1576, 1495, 1459, 1414, 1378, 1330, 1273, 1235, 1181, 1157, 1090, 1016, 964, 913, 856, 813, 768, 746, 706, 693, 660, 551; HR-MS (ESI) m/z calcd for $C_{53}H_{72}NO_5S$ [M+H$^+$] 834.5126 found 834.5130; $[\alpha]_D^{24.5}$ = -5.6 (c = 0.1, $CHCl_3$).

(*R*)-2, 5, 7, 8-Tetramethyl-2-((4R, 8R)-4, 8, 12-trimethyltridecyl)chroman-6-yl 4-((*R*, *E*)-1-((4-methylphenyl)sulfonamido)-4-phenylbut-3-en-2-yl)benzoate (*epi*-36)

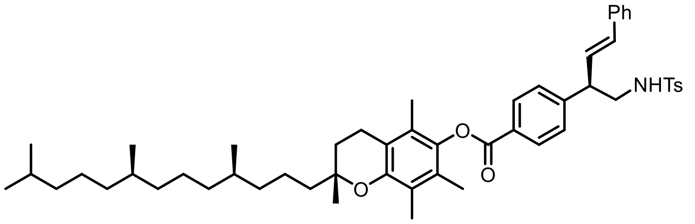

Prepared by **GP-A** by using **-L1** as ligand. Colorless oil, 113.6 mg, 68% yield. ^{1}H NMR (400 MHz, $CDCl_3$) δ 8.15 – 8.05 (m, 2H), 7.68 – 7.58 (m, 2H), 7.27 – 7.13 (m, 9H), 6.35 (d, *J* = 15.9 Hz, 1H), 6.11 (dd, *J* = 15.9, 7.9 Hz, 1H), 4.48 (t, *J* = 6.2 Hz, 1H), 3.63 (q, *J* = 7.5 Hz, 1H), 3.29 (t, *J* = 6.8 Hz, 2H), 2.54 (t, *J* = 6.6 Hz, 2H), 2.35 (s, 3H), 2.04 (s, 3H), 1.97 (s, 3H), 1.93 (s, 3H), 1.82 – 1.64 (m, 2H), 1.55 – 1.13 (m, 18H), 1.10 – 0.92 (m, 6H), 0.84 – 0.73 (m, 12H); ^{13}C NMR (101 MHz, $CDCl_3$) δ 164.7, 149.5, 146.2, 143.6, 140.5, 136.8, 136.3, 133.0, 130.8, 129.8, 128.7, 128.6, 128.2, 128.0, 127.9, 127.1, 126.8, 126.3, 125.1, 123.1, 117.5, 75.1, 48.9, 47.3, 40.4, 39.6, 39.3, 37.4, 37.3, 32.8, 31.2, 31.0, 28.0, 24.8, 24.4, 24.2, 23.6, 22.7, 22.6, 21.5, 21.0, 20.6, 19.7, 19.6, 13.0, 12.2, 11.8. $[\alpha]_D^{24.7}$ = 5.3 (c = 0.1, $CHCl_3$).**4-((*S*, *E*)-4-((4-Methylphenyl)sulfonamido)-3-phenylbut-1-en-1-yl)phenyl (S)-2-(6-methoxynaphthalen-2-yl)propanoate (37)**

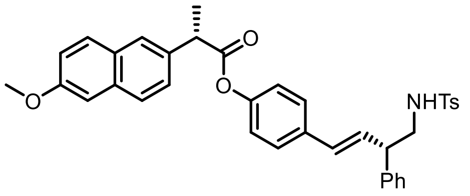

Prepared by **GP-A**. White solid, 96.8 mg, 80% yield. m.p. = 55-56 °C; ^{1}H NMR (400 MHz, $CDCl_3$) δ 7.71 – 7.57 (m, 5H), 7.41 (dd, *J* = 8.6, 1.7 Hz, 1H), 7.25 – 7.13 (m, 7H), 7.10 – 7.00 (m, 4H), 6.86 – 6.80 (m, 2H), 6.24 (d, *J* = 15.9 Hz, 1H), 6.01 (dd, *J* = 15.9, 7.8 Hz, 1H), 4.30 (t, *J* = 6.2 Hz, 1H), 4.04 – 3.95 (m, 1H), 3.84 (s, 3H), 3.47 (q, *J* = 7.5 Hz, 1H), 3.22 (t, *J* = 6.9 Hz, 2H), 2.34 (s, 3H), 1.60 (d, *J* = 7.1 Hz, 3H); ^{13}C NMR (101 MHz, $CDCl_3$) δ 173.0, 157.7, 150.2, 143.5, 140.1, 136.8, 135.0, 134.2, 133.8, 131.2, 129.7, 129.4, 129.3, 129.0, 127.6, 127.4, 127.3, 127.10, 127.07, 126.10, 126.06, 121.5, 119.1, 105.6, 55.3, 48.6, 47.4, 45.5, 21.5, 18.5; IR (film): ν (cm^{-1}) 3284, 3028, 2932, 1749, 1631, 1604,, 1505, 1486, 1453, 1391, 1326, 1263, 1230, 1213, 1198, 1156, 1092, 1069, 1030, 1016, 967, 927, 909, 893, 853, 813, 731, 699, 661, 551, 541,510, 473; HR-MS (ESI) m/z calcd for $C_{37}H_{36}NO_5S$ [M+H$^+$] 606.2309, found 606.2305; $[\alpha]_D^{24.6}$ = 44.3 (c = 0.1, $CHCl_3$).

4-((*S*, *E*)-4-((4-Methylphenyl)sulfonamido)-3-phenylbut-1-en-1-yl)phenyl (S)-2-(6-methoxynaphthalen-2-yl)propanoate (*epi*-37)

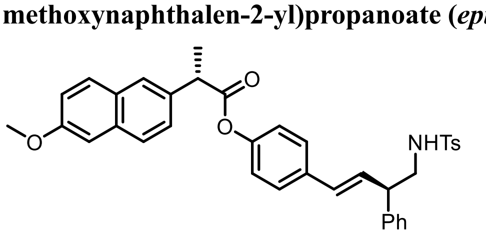

Prepared by **GP-A** by using **-L1** as ligand. Colorless oil, 95.5 mg, 79% yield. ^{1}H NMR (400 MHz, $CDCl_3$) δ 7.71 – 7.56 (m, 5H), 7.40 (dd, *J* = 8.5, 1.7 Hz, 1H), 7.23 – 7.11 (m, 7H), 7.10 – 6.98 (m, 4H), 6.88 – 6.78 (m, 2H), 6.22 (d, *J* = 15.9 Hz, 1H), 6.00 (dd, *J* = 15.9, 7.8 Hz, 1H), 4.37 (t, *J* = 6.2 Hz, 1H), 4.06 – 3.93 (m, 1H), 3.83 (s, 3H), 3.46 (q, *J* = 7.5 Hz, 1H), 3.20 (t, *J* = 6.9 Hz, 2H), 2.32 (s, 3H), 1.60 (d, *J* = 7.1 Hz, 3H); ^{13}C NMR (101 MHz, $CDCl_3$) δ 173.0, 157.7, 150.2, 143.4, 140.1, 136.8, 135.0, 134.2, 133.8, 131.2, 129.7, 129.4, 129.3, 129.0, 127.6, 127.4, 127.3, 127.10, 127.07, 126.10, 126.06, 121.5, 119.1, 105.6, 55.3, 48.6, 47.4, 45.5,

21.5, 18.5; $[\alpha]_D^{24.8}$ = -43.7 (c = 0.1, $CHCl_3$).**(*S*, *E*)-4-(4-((4-Methylphenyl)sulfonamido)-3-phenylbut-1-en-1-yl)phenyl 2-(4-(4-chlorobenzoyl)phenoxy)-2-methylpropanoate (38)**

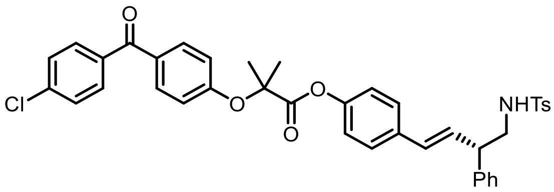

Prepared by **GP-A**. Colorless oil, 63.9 mg, 46% yield. ^1H NMR (400 MHz, $CDCl_3$) δ 7.73 – 7.68 (m, 2H), 7.66 – 7.58 (m, 4H), 7.40 – 7.35 (m, 2H), 7.25 – 7.13 (m, 7H), 7.06 – 7.02 (m, 2H), 6.93 – 6.88 (m, 2H), 6.85 – 6.80 (m, 2H), 6.27 (d, *J* = 15.9 Hz, 1H), 6.06 (dd, *J* = 15.9, 7.8 Hz, 1H), 4.39 (t, *J* = 6.3 Hz, 1H), 3.49 (q, *J* = 7.5 Hz, 1H), 3.29 – 3.17 (m, 2H), 2.34 (s, 3H), 1.74 (s, 6H); ^{13}C NMR (101 MHz, $CDCl_3$) δ 194.2, 172.3, 159.5, 149.7, 143.5, 140.0, 138.4, 136.8, 136.2, 134.8, 132.1, 131.2, 131.0, 130.7, 120.0, 129.7, 129.0, 128.6, 127.6, 127.4, 127.3, 127.1, 121.2, 117.3, 79.4, 48.6, 47.4, 25.4, 21.5; IR (film): ν (cm^{-1}) 3286, 3032, 2991, 2921, 1753, 1651, 1596, 1504, 1452, 1415, 1397, 1386, 1328, 1319, 1305, 1285, 1277, 1247, 1211, 1195, 1157, 1114, 1090, 1014, 970, 928, 910, 851, 837, 813, 730, 701, 660, 552; HR-MS (ESI) m/z calcd for $C_{40}H_{37}ClNO_6S$ $[M+H^+]$ 694.2025, found 694.2023; $[\alpha]_D^{24.4}$ = -22.6 (c = 0.1, $CHCl_3$). HPLC conditions: AD-H column, hexane/2-propanol = 60/40, flow rate = 1.0 mL/min, λ = 254 nm, t_R = 51.3 min (major), t_R = 59.7 min (minor), 95:5 er.

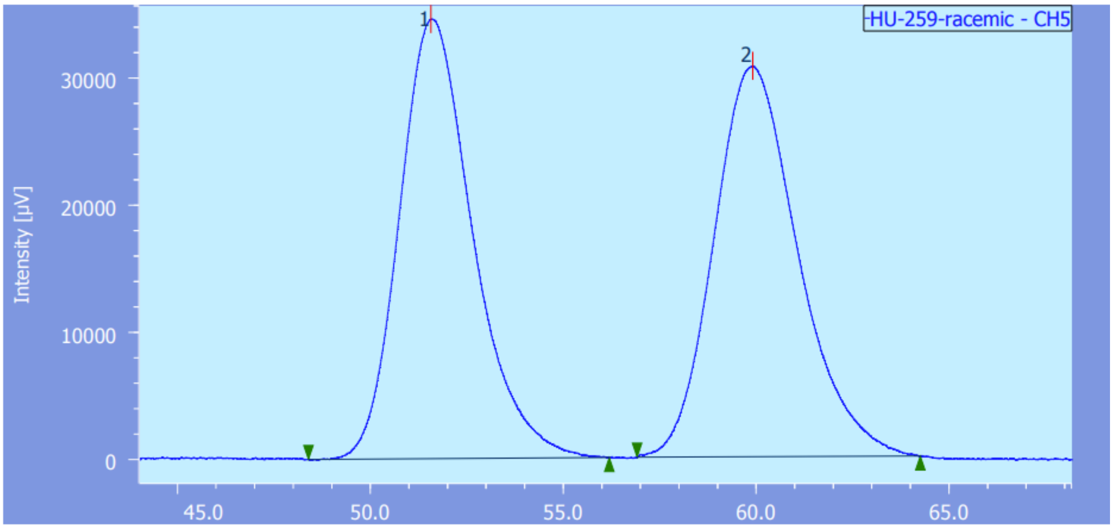

Decision

#	Peak Name	CH	tR [min]	Area [µV·sec]	Height [µV]	Area%	Height%	Quantity	NTP	Resolution	Symmetry Factor	Warning
1	Unknown	5	51.567	4562733	34544	49.499	52.935	N/A	3710	2.288	1.287	
2	Unknown	5	59.910	4655024	30713	50.501	47.065	N/A	3722	N/A	1.207	

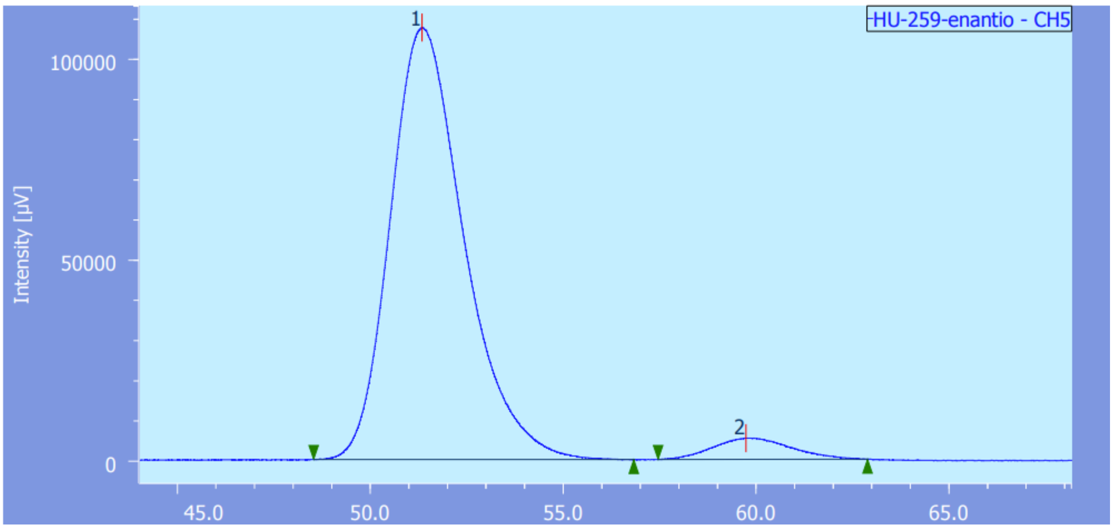

Decision

#	Peak Name	CH	tR [min]	Area [µV·sec]	Height [µV]	Area%	Height%	Quantity	NTP	Resolution	Symmetry Factor	Warning
1	Unknown	5	51.337	14266908	107448	95.029	95.301	N/A	3634	2.343	1.306	
2	Unknown	5	59.737	746277	5298	4.971	4.699	N/A	3987	N/A	1.217	

(*S*, *E*)-4-(4-((4-Methylphenyl)sulfonamido)-3-phenylbut-1-en-1-yl)phenyl 5-(2, 5-dimethylphenoxy)-2,2-dimethylpentanoate (39)

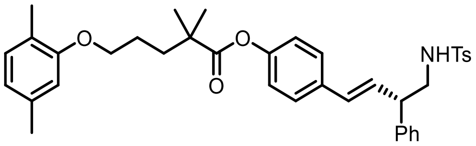

Prepared by **GP-A**. Colorless oil, 64.8 mg, 53% yield. ^{1}H NMR (400 MHz, $CDCl_3$) δ 7.70 – 7.51 (m, 2H), 7.26 – 7.12 (m, 7H), 7.07 – 7.00 (m, 2H), 6.92 (d, *J* = 7.5 Hz, 1H), 6.90 – 6.83 (m, 2H), 6.58 (d, *J* = 7.5 Hz, 1H), 6.54 (s, 1H), 6.26 (d, *J* = 15.9 Hz, 1H), 6.04 (dd, *J* = 15.9, 7.8 Hz, 1H), 4.43 (t, *J* = 6.2 Hz, 1H), 3.98 – 3.80 (m, 2H), 3.48 (q, *J* = 7.5 Hz, 1H), 3.22 (t, *J* = 6.8 Hz, 2H), 2.34 (s, 3H), 2.22 (s, 3H), 2.09 (s, 3H), 1.86 – 1.70 (m, 4H), 1.28 (s, 6H); ^{13}C NMR (101 MHz, $CDCl_3$) δ 176.2, 156.8, 150.4, 143.4, 140.1, 136.8, 136.4, 134.2, 131.2, 130.3, 129.7, 129.4, 129.0, 127.6, 127.3, 127.2, 127.1, 123.5, 121.6, 120.7, 111.9, 67.7, 48.6, 47.4, 42.4, 37.1, 25.2, 25.1, 21.5, 21.4, 15.8; IR (film): ν (cm^{-1}) 3292, 2922, 2870, 1746, 1614, 1600, 1585, 1506, 1472, 1452, 1413, 1390, 1325, 1286, 1262, 1209, 1198, 1186, 1156, 1111, 1045, 967, 810, 758, 700, 661, 551, 543, 513; HR-MS (ESI) m/z calcd for $C_{38}H_{44}NO_5S$ [M+H$^+$] 626.2935, found 626.2930; $[\alpha]_D^{24.5}$ = -27.1 (c = 0.1, $CHCl_3$). HPLC conditions: OD-H column, hexane/2-propanol = 90/10, flow rate = 1.0 mL/min, λ = 254 nm, t_R = 27.6 min (minor), t_R = 33.2 min (major), 95:5 er.

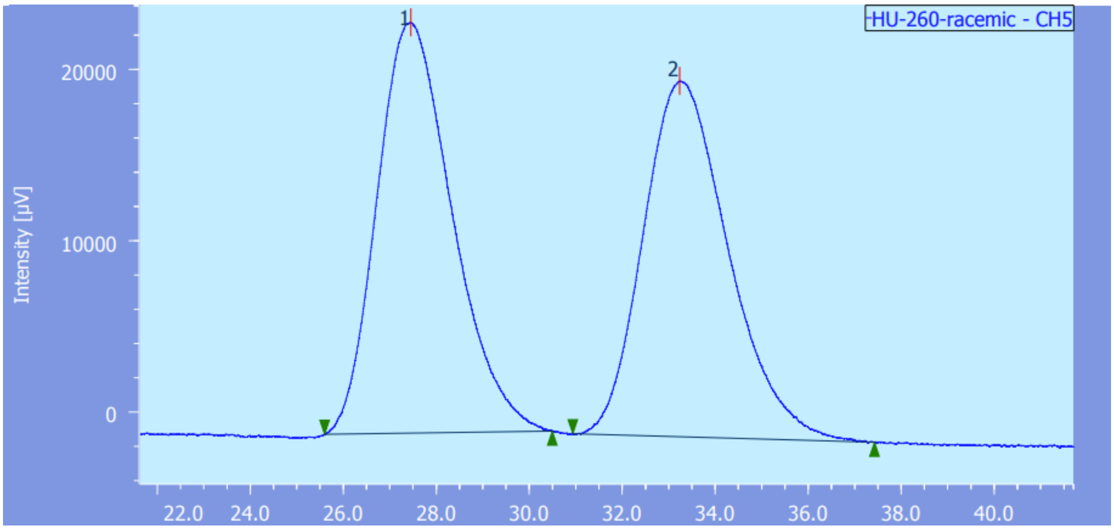

Decision

#	Peak Name	CH	tR [min]	Area [µV·sec]	Height [µV]	Area%	Height%	Quantity	NTP	Resolution	Symmetry Factor	Warning
1	Unknown	5	27.453	2699003	23955	50.514	53.573	N/A	1364	1.837	1.254	
2	Unknown	5	33.233	2644044	20760	49.486	46.427	N/A	1589	N/A	1.276	

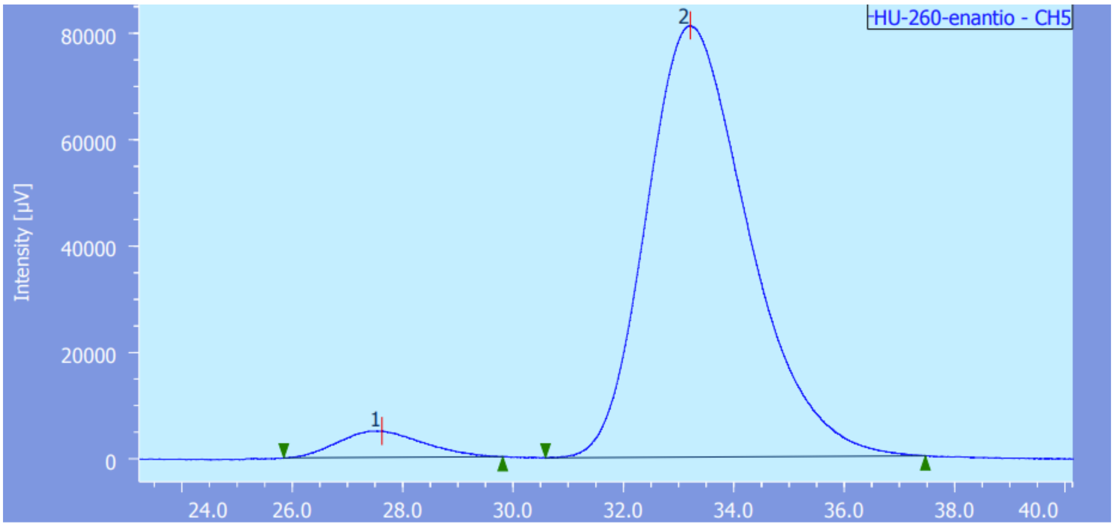

Decision

#	Peak Name	CH	tR [min]	Area [µV·sec]	Height [µV]	Area%	Height%	Quantity	NTP	Resolution	Symmetry Factor	Warning
1	Unknown	5	27.623	535140	4959	4.884	5.762	N/A	1427	1.785	1.107	
2	Unknown	5	33.207	10422539	81103	95.116	94.238	N/A	1574	N/A	1.305	

4-((*S*, *E*)-4-((4-Methylphenyl)sulfonamido)-3-phenylbut-1-en-1-yl)phenyl (*tert*-butoxycarbonyl)-D-alaninate (40)

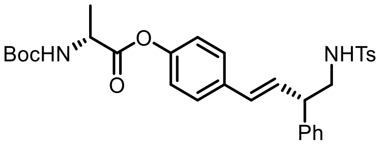

Prepared by **GP-A**. White solid, 97.1 mg, 86% yield. m.p. = 149-150 °C; ^{1}H NMR (400 MHz, $CDCl_3$) δ 7.66 – 7.58 (m, 2H), 7.26 – 7.14 (m, 7H), 7.08 – 7.01 (m, 2H), 6.97 – 6.90 (m, 2H), 6.27 (d, *J* = 15.9 Hz, 1H), 6.06 (dd, *J* = 15.9, 7.8 Hz, 1H), 5.03 (d, *J* = 6.3 Hz, 1H), 4.47 (t, *J* = 6.2 Hz, 2H), 3.49 (q, *J* = 7.5 Hz, 1H), 3.31 – 3.14 (m, 2H), 2.35 (s, 3H), 1.46 (d, *J* = 7.2 Hz, 3H), 1.38 (s, 9H); ^{13}C NMR (101 MHz, $CDCl_3$) δ 172.0, 155.1, 149.8, 143.5, 140.1, 136.8, 134.6, 131.1, 129.8, 129.7, 129.0, 127.6, 127.3, 127.2, 127.1, 121.4, 80.1, 49.4, 48.6, 47.4, 28.3, 21.5, 18.4; IR (film): ν (cm^{-1}) 3380, 3295, 2977, 2931, 1764, 1688, 1601, 1504, 1454, 1426, 1366, 1322, 1307, 1251, 1211, 1192, 1163, 1145, 1093, 1064, 1014, 964, 893, 865, 842, 817, 761, 731, 696, 662, 551, 536, 510, 494; HR-MS (ESI) m/z calcd for $C_{31}H_{37}N_2O_6S$ [M+H$^+$]

565.2367, found 565.2369; $[\alpha]_D^{24.6}$ = -10.7 (c = 0.1, $CHCl_3$).

4-((*R*, *E*)-4-((4-Methylphenyl)sulfonamido)-3-phenylbut-1-en-1-yl)phenyl (*tert*-butoxycarbonyl)-D-alaninate (*epi*-40)

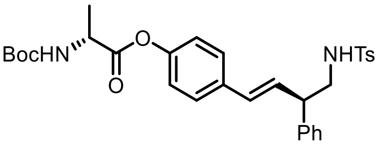

Prepared by **GP-A** by using **-L1** as ligand. Colorless oil, 97.5 mg, 86% yield. ^{1}H NMR (400 MHz, $CDCl_3$) δ 7.66 – 7.60 (m, 2H), 7.26 – 7.15 (m, 7H), 7.08 – 7.02 (m, 2H), 6.97 – 6.91 (m, 2H), 6.28 (d, *J* = 15.9 Hz, 1H), 6.06 (dd, *J* = 15.9, 7.8 Hz, 1H), 5.02 (d, *J* = 6.2 Hz, 1H), 4.46 (t, *J* = 6.8 Hz, 1H), 4.40 (t, *J* = 6.2 Hz, 1H), 3.50 (q, *J* = 7.5 Hz, 1H), 3.28 – 3.20 (m, 2H), 2.35 (s, 3H), 1.47 (d, *J* = 7.2 Hz, 3H), 1.39 (s, 9H); ^{13}C NMR (101 MHz, $CDCl_3$) δ 172.0, 155.1, 149.9, 143.5, 140.1, 136.9, 134.6, 131.1, 129.7, 129.0, 127.6, 127.33, 127.26, 127.1, 121.4, 80.1, 49.4, 48.6, 47.4, 28.3, 21.5, 18.4; $[\alpha]_D^{24.8}$ = 11.1 (c = 0.1, $CHCl_3$).

(*S*)-*N*-(2-(4-(*tert*-Butyl)phenyl)-2-phenylethyl)-4-methylbenzenesulfonamide (41)

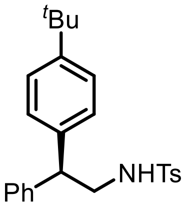

Prepared by **GP-A** by using **L9** as ligand and 1.0 equiv $MgCl_2$ as additive. White solid, 32.6 mg, 40% yield. m.p. = 158-159 °C; ^{1}H NMR (400 MHz, $CDCl_3$) δ 7.61 (d, *J* = 8.2 Hz, 2H), 7.26 – 7.09 (m, 7H), 7.05 – 6.98 (m, 2H), 6.94 (d, *J* = 8.3 Hz, 2H), 4.25 (t, *J* = 6.1 Hz, 1H), 3.94 (t, *J* = 8.0 Hz, 1H), 3.51 – 3.41 (m, 2H), 2.37 (s, 3H), 1.21 (s, 9H); ^{13}C NMR (101 MHz, $CDCl_3$) δ 150.0, 143.5, 140.8, 137.5, 136.8, 129.7, 128.8, 127.9, 127.5, 127.2, 127.0, 125.8, 50.1, 47.3, 34.4, 31.3, 21.5; IR (film): ν (cm^{-1}) 3281, 2960, 2907, 2861, 1597, 1495, 1454, 1426, 1413, 1321, 1150, 1092, 1072, 874, 856, 832, 818, 751, 702, 683, 667, 599, 548, 521, 491; HR-MS (ESI) m/z calcd for $C_{25}H_{30}NO_2S$ [M+H$^+$] 408.1992, found 408.1993; $[\alpha]_D^{23.1}$ = 3.3 (c = 0.1, $CHCl_3$); HPLC conditions: OD-H column, hexane/2-propanol = 90/10, flow rate = 1.0 mL/min, λ = 254 nm, t_R = 10.6 min (minor), t_R =14.2 min (major), 90:10 er.

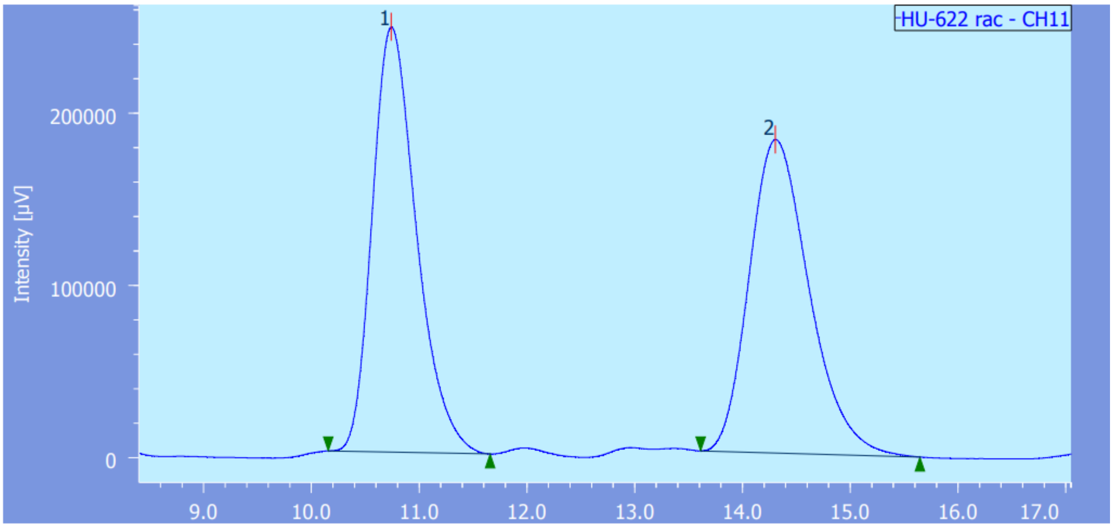

Decision

#	Peak Name	CH	tR [min]	Area [µV·sec]	Height [µV]	Area%	Height%	Quantity	NTP	Resolution	Symmetry Factor	Warning
1	Unknown	11	10.740	7040761	246720	50.012	57.564	N/A	3376	4.087	1.319	
2	Unknown	11	14.303	7037521	181880	49.988	42.436	N/A	3216	N/A	1.282	

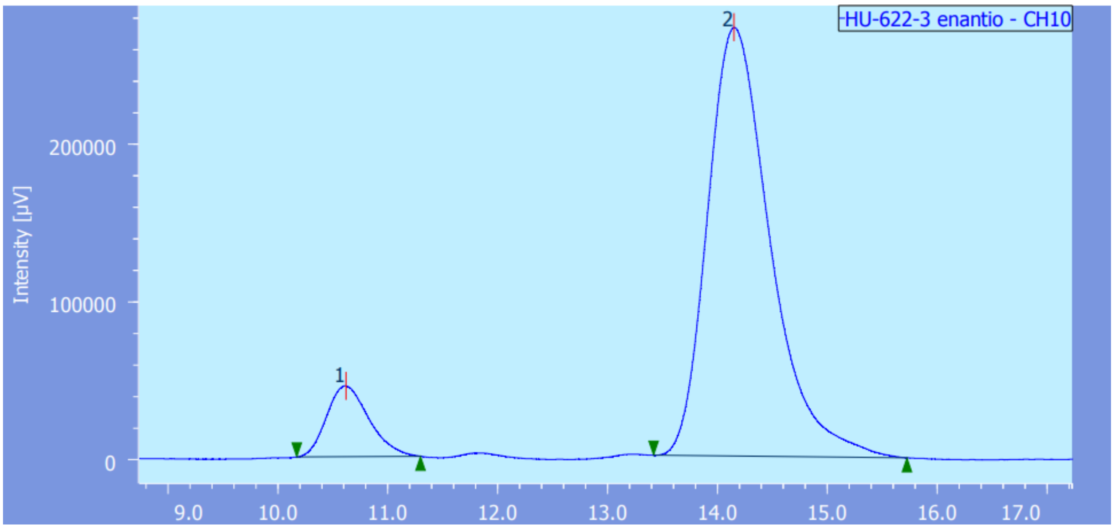

Decision

#	Peak Name	CH	tR [min]	Area [µV·sec]	Height [µV]	Area%	Height%	Quantity	NTP	Resolution	Symmetry Factor	Warning
1	Unknown	10	10.620	1206321	44907	10.078	14.184	N/A	3625	4.132	1.233	
2	Unknown	10	14.150	10764113	271686	89.922	85.816	N/A	3154	N/A	1.415	

(*S*, *E*)-1-Methoxy-4-(3-phenylbut-1-en-1-yl)benzene (42)

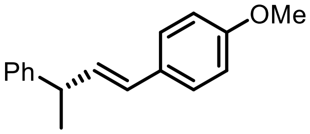

Prepared by **GP-A**. Colorless oil, 25.8 mg, 54% yield. ^{1}H NMR (400 MHz, $CDCl_3$) δ 7.36 – 7.26 (m, 6H), 7.25 – 7.19 (m, 1H), 6.89 – 6.80 (m, 2H), 6.38 (d, *J* = 16.0 Hz, 1H), 6.26 (dd, *J* = 15.9, 6.7 Hz, 1H), 3.81 (s, 3H), 3.70 – 3.56 (m, 1H), 1.47 (d, *J* = 7.0 Hz, 3H); ^{13}C NMR (101 MHz, $CDCl_3$) δ 158.8, 145.9, 133.1, 130.4, 128.4, 127.8, 127.3, 127.2, 126.1, 113.9, 55.3, 42.5, 21.3; IR (film): ν (cm^{-1}) 3020, 2959, 2932, 2838, 1731, 1606, 1511, 1453, 1248, 1215, 1173, 1035, 968, 819, 749, 701, 666; HR-MS (ESI) m/z calcd for $C_{19}H_{19}O$ [M+H$^+$] 239.1430, found 239.1429; $[\alpha]_D^{23.4}$ = -48.6 (c = 0.1, $CHCl_3$); HPLC conditions: OJ-H column, hexane/2-propanol = 90/10, flow rate = 1.0 mL/min, λ = 254 nm, t_R = 11.1 min (minor), t_R =13.7 min (major), 97:4 er.

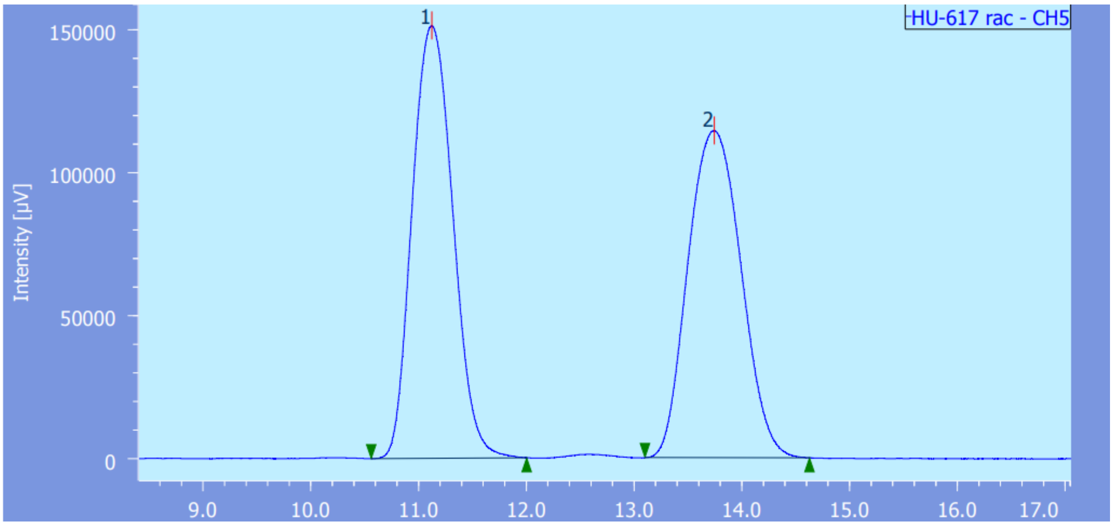

Decision

#	Peak Name	CH	tR [min]	Area [µV·sec]	Height [µV]	Area%	Height%	Quantity	NTP	Resolution	Symmetry Factor	Warning
1	Unknown	5	11.123	3970649	151319	50.281	56.945	N/A	3908	3.172	1.127	
2	Unknown	5	13.743	3926276	114410	49.719	43.055	N/A	3385	N/A	1.088	

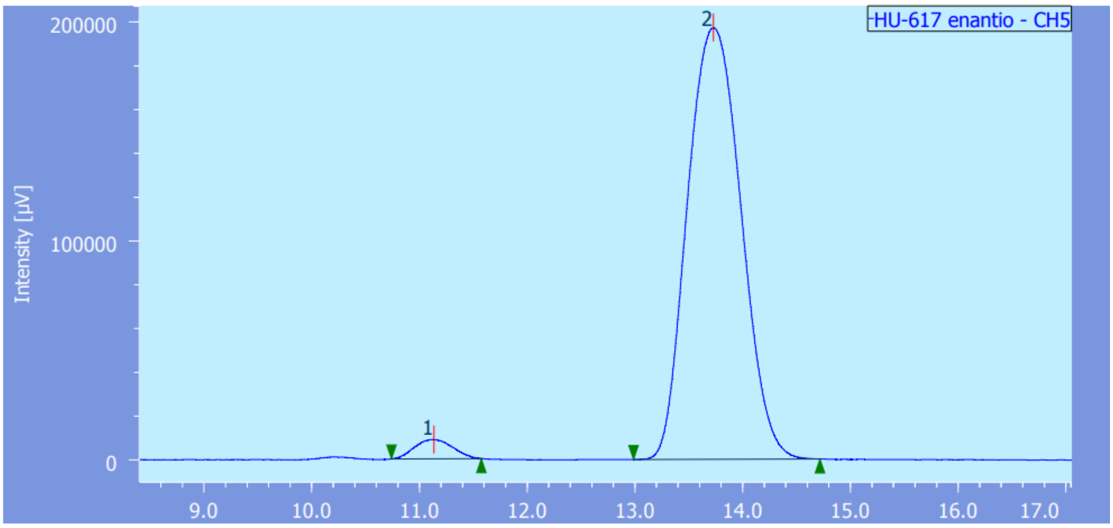

Decision

#	Peak Name	CH	tR [min]	Area [µV·sec]	Height [µV]	Area%	Height%	Quantity	NTP	Resolution	Symmetry Factor	Warning
1	Unknown	5	11.133	218011	8762	3.096	4.256	N/A	4082	3.154	1.049	
2	Unknown	5	13.727	6824070	197087	96.904	95.744	N/A	3326	N/A	1.110	

6. Synthetic Transformations of Products

(*S*)-*N*-(2, 4-Diphenylbutyl)-4-methylbenzenesulfonamide (43)

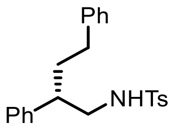

A reaction tube was charged (*S*, *E*)-*N*-(2, 4-diphenylbut-3-en-1-yl)-4-methylbenzenesulfonamide (**1**) (37.7 mg, 0.1 mmol), ethanol (2 mL) and Pd/C (10.6 mg, 0.01 mmol, 10 wt%). The tube was evacuated and backfilled with H_2 (1 atm) and this process was repeated 3 times. The reaction was stirred at 25 °C with a hydrogen balloon until TLC shows complete consumption of starting material. The crude mixture was filtered through a short pad of celite and the solvent evaporated under reduced pressure. After purification by chromatography on silica gel (hexanes : EtOAc = 5:1), the title compound was obtained as a white solid in 98% yield (37.4 mg). m.p. = 134-135 °C; ^{1}H NMR (400 MHz, $CDCl_3$) δ 7.57 – 7.50 (m, 2H), 7.26 – 7.12 (m, 7H), 7.12 – 7.05 (m, 1H), 7.01 – 6.91 (m, 4H), 4.11 (dd, *J* = 8.3, 4.1 Hz, 1H), 3.29 – 3.12 (m, 1H), 3.00 – 2.86 (m, 1H), 2.66 – 2.54 (m, 1H), 2.44 – 2.25 (m, 5H), 1.95 – 1.82 (m, 1H), 1.81 – 1.67 (m, 1H); ^{13}C NMR (101 MHz, $CDCl_3$) δ 143.3, 141.5, 141.0, 136.8, 129.6, 129.0, 128.32, 128.27, 127.8, 127.2, 127.0, 125.9, 48.5, 44.9, 35.0, 33.1, 21.5; IR (film): ν (cm^{-1}) 33293, 3029, 2933, 2918, 1601, 1494, 1451, 1423, 1320, 1310, 1290, 1149, 1094, 1069, 1060, 847, 819, 758, 748, 697, 671, 551, 528, 498, 489; HR-MS (ESI) m/z calcd for $C_{23}H_{26}NO_2S$ [M+H$^+$] 380.1679, found 380.1680; $[\alpha]_D^{24.6}$ = -12.7 (c = 0.1, $CHCl_3$); HPLC conditions: OJ-H column, hexane/2-propanol = 95/5, flow rate = 1.0 mL/min, λ = 220 nm, t_R = 67.0 min (minor), t_R = 83.2 min (major), 96:4 er.

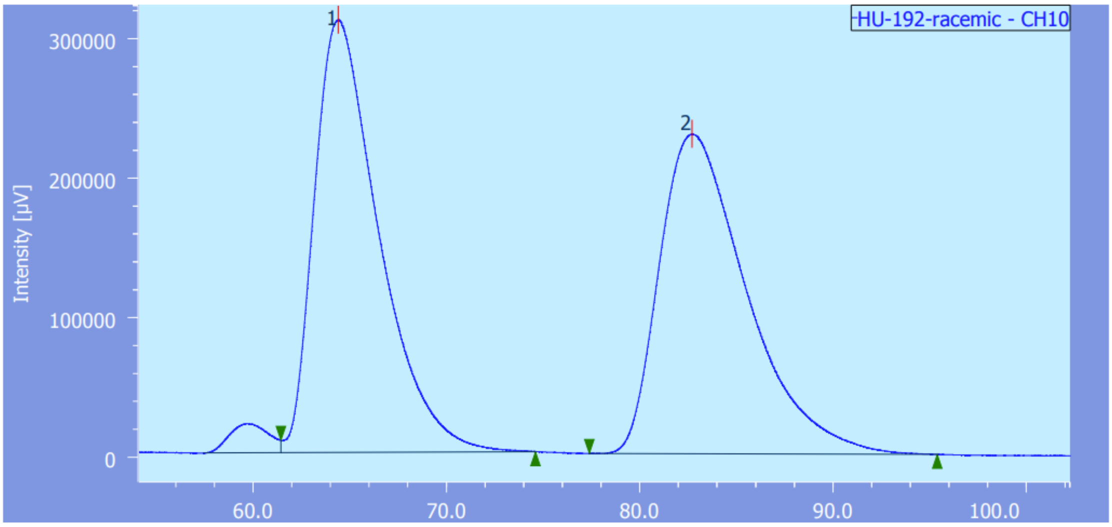

Decision

#	Peak Name	CH	tR [min]	Area [µV·sec]	Height [µV]	Area%	Height%	Quantity	NTP	Resolution	Symmetry Factor	Warning
1	Unknown	10	64.407	72032525	309975	50.049	57.495	N/A	1857	2.604	1.632	
2	Unknown	10	82.697	71891427	229162	49.951	42.505	N/A	1665	N/A	1.642	

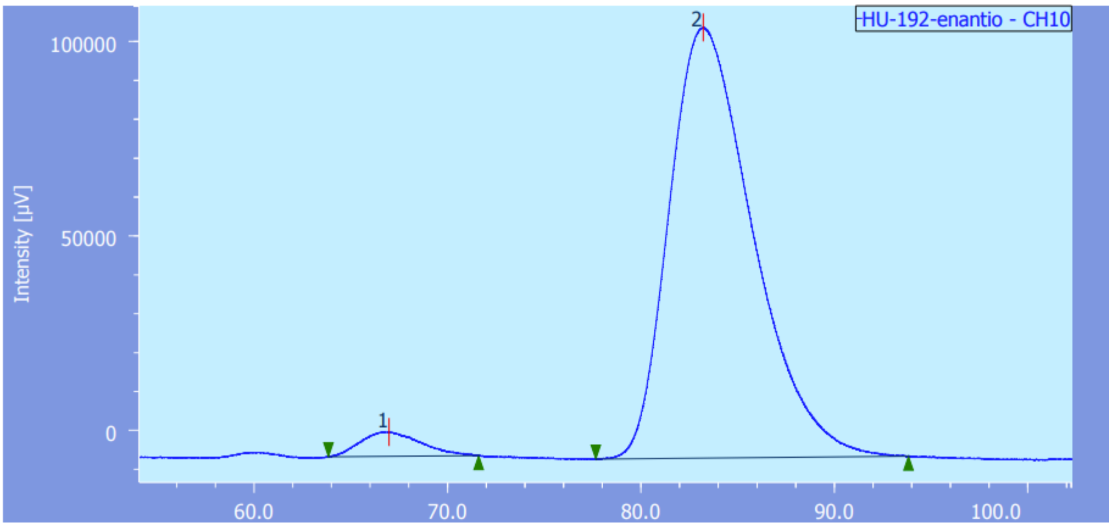

Decision

#	Peak Name	CH	tR [min]	Area [μV·sec]	Height [μV]	Area%	Height%	Quantity	NTP	Resolution	Symmetry Factor	Warning
1	Unknown	10	66.980	1347041	6250	3.896	5.347	N/A	2057	2.362	1.204	
2	Unknown	10	83.210	33230711	110639	96.104	94.653	N/A	1787	N/A	1.408	

(2*S*, 3*R*, 4*S*)-3-iodo-2, 4-diphenyl-1-tosylpyrrolidine (44)

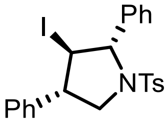

To an oven-dried vial was added $NaHCO_3$ (0.3 mmol, 3 equiv), (*S*, *E*)-*N*-(2, 4-diphenylbut-3-en-1-yl)-4-methylbenzenesulfonamide (**1**) (37.7 mg, 0.1 mmol) and MeCN (1.5 ml), I_2 (0.3 mmol, 3 equiv) was then added to the reaction in one portion, the reaction was allowed to stir for 12 hours. After the reaction was complete as monitored by TLC, the reaction was quenched by 2 mL $Na_2S_2O_3$. The product was extracted with EtOAc, dried over Na_2SO_4, concentrated, and purified by chromatography on silica gel (hexanes : EtOAc = 5:1), the title compound was obtained as a white solid in 99% yield (50.0 mg). m.p. = 123-124 °C; ^{1}H NMR (400 MHz, $CDCl_3$) δ 7.53 – 7.46 (d, *J* = 8.3 Hz, 2H), 7.38 – 7.30 (m, 2H), 7.29 – 7.16 (m, 8H), 7.11 – 7.05 (dt, *J* = 4.1, 2.3 Hz, 2H), 4.91 (d, *J* = 8.9 Hz, 1H), 4.01 (dd, *J* = 11.5, 8.1 Hz, 1H), 3.93 (dd, *J* = 11.5, 8.9 Hz, 1H), 3.48 (t, *J* = 11.3 Hz, 1H), 3.11 (td, *J* = 11.2, 8.1 Hz, 1H), 2.36 (s, 3H); ^{13}C NMR (101 MHz, $CDCl_3$) δ 143.7, 138.7, 135.9, 135.1, 129.6, 128.9, 128.5, 128.2, 128.1, 127.4, 127.3, 127.2, 73.4, 54.8, 54.7, 35.7, 21.5; IR (film): ν (cm^{-1}) 2933, 1596, 1495, 1461, 1457, 1346, 1313, 1231, 1217, 1161, 1149, 1091, 1072, 1032, 1016, 976, 912, 856, 842, 818, 806, 795, 766, 754, 698, 688, 659, 616, 576, 545, 525, 495; HR-MS (ESI) m/z calcd for $C_{23}H_{23}INO_2S$ [M+H$^+$] 504.0489, found 504.0492; $[\alpha]_D^{24.6}$ = 43.9 (c = 0.1, $CHCl_3$).

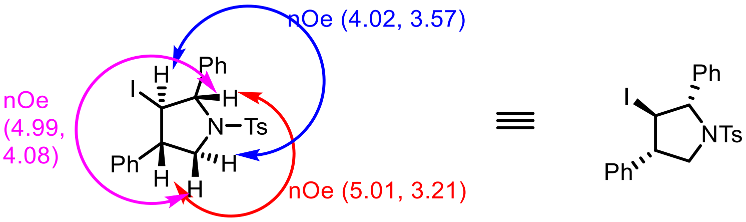

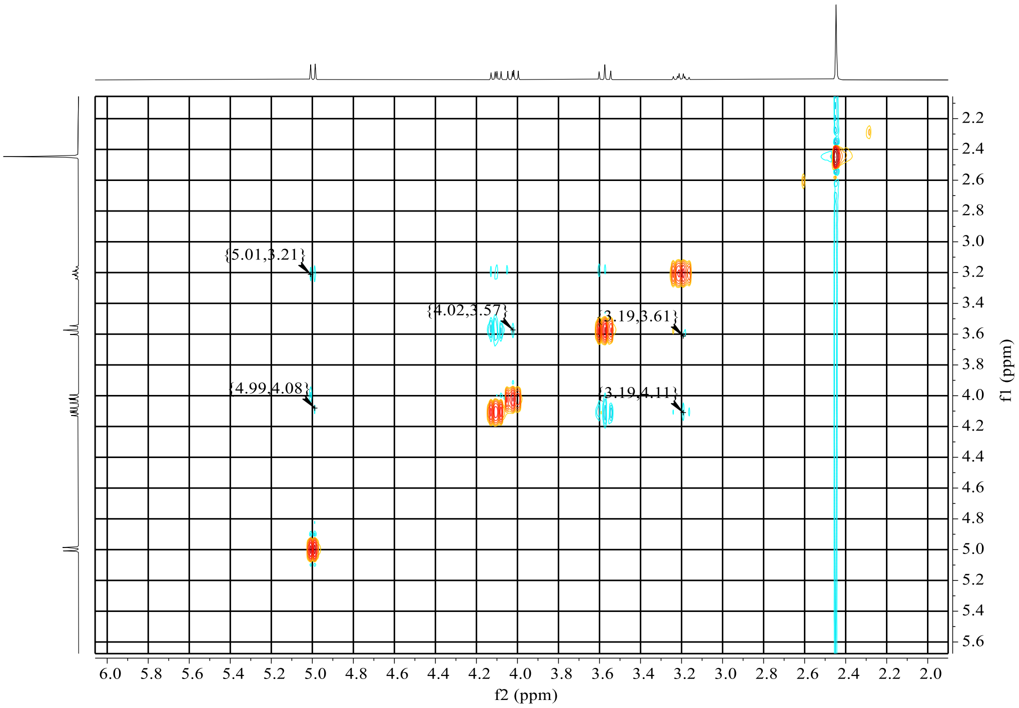

Figure S-1. NOESY of **44**.

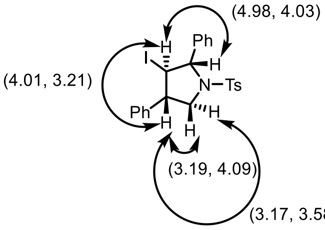

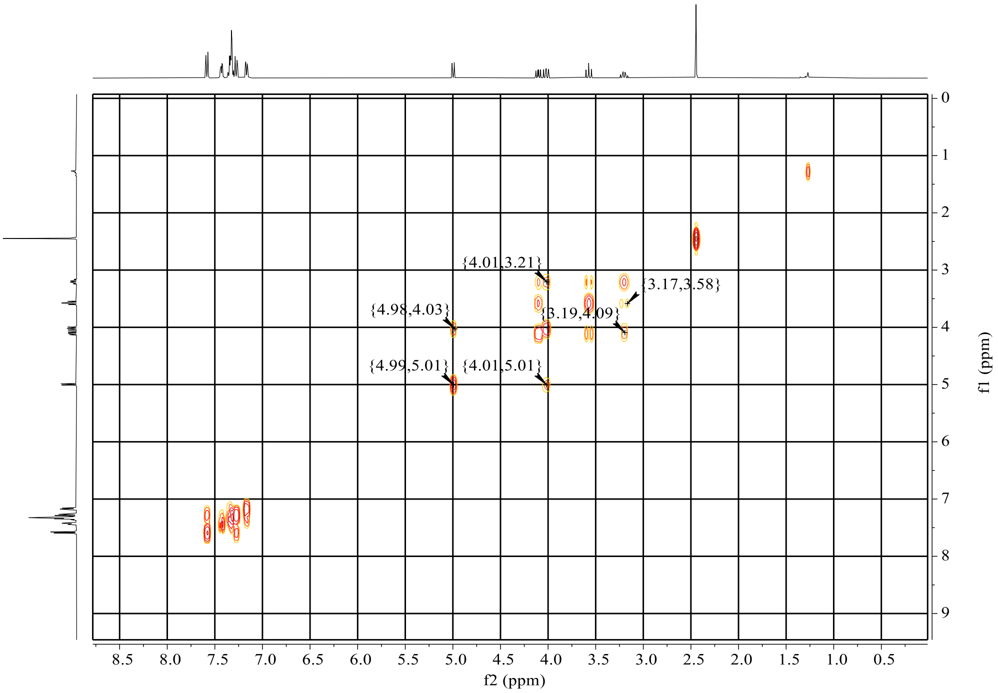

Figure S-2. COSY of **44**.

(2*R*, 4*S*)-2, 4-diphenyl-1-tosylpyrrolidine (45)

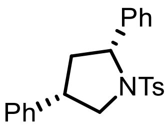

A oven-dried vial equipped with a stirring bar was charged with (2*S*, 3*R*, 4*S*)-3-iodo-2, 4-diphenyl-1-tosylpyrrolidine (50.3 mg, 0.1 mmol) and 4CzIPN (1,2,3,5-Tetrakis(carbazol-9-yl)-4,6-dicyanobenzene, 4 mg, 5 mol%), the tube was evacuated and refilled with N_2 for three times. Degassed $CH_3CN–H_2O$ (5:1) (0.1 M), methyl thioglycolate (1.8 uL, 0.02 mmol, 20 mol%) and NEt_3 (42 uL, 0.3 mmol, 3.0 equiv) were sequentially added and the mixture was stirred in front of blue LEDs for 4 hours at 25 °C. Brine (3 mL) and EtOAc (3 mL) were added and the mixture was shaken vigorously. 1, 3-dinitrobenzene (17 mg, 0.1 mmol, 1.0 equiv) was added and the layers were separated. The aqueous layer was extracted with EtOAc, the combined organic layers were dried ($MgSO_4$), filtered, evaporated, and purified by chromatography on silica gel (hexanes : EtOAc = 10:1), the title compound was obtained as a colorless oil in 72% yield (27.4 mg). ^1H NMR (400 MHz, $CDCl_3$) δ 7.60 – 7.52 (m, 2H), 7.30 – 7.11 (m, 10H), 7.07 – 7.01 (m, 2H), 4.74 (dd, *J* = 9.9, 7.0 Hz, 1H), 4.09 (ddd, *J* = 11.3, 7.5, 1.3 Hz, 1H), 3.43 (t, *J* = 11.3 Hz, 1H), 2.97 – 2.80 (m, 1H), 2.68 – 2.53 (m, 1H), 2.35 (s, 3H), 2.03 – 1.91 (m, 1H); ^{13}C NMR (101 MHz, $CDCl_3$) δ 143.3, 142.5, 139.0, 135.6, 129.6, 128.6, 128.4, 127.4, 127.3, 127.1, 127.0, 126.4, 64.4, 55.9, 44.3, 43.6, 21.5; IR (film): ν (cm^{-1}) 3059, 3029, 2924, 2880, 1599, 1495, 1451, 1345, 1302, 1287, 1183, 1160, 1090, 1076, 1027, 959, 816, 755, 740, 697, 660, 589, 566, 545, 509; HR-MS (ESI) m/z calcd for $C_{23}H_{24}NO_2S$ [$M+H^+$] 378.1522, found 378.1523; $[\alpha]_D^{24.6}$ = 32.1 (c = 0.1, $CHCl_3$).

(*S*, *E*)-2, 4-Diphenylbut-3-en-1-amine (46)

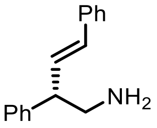

In a round bottom flask equipped with a magnetic stir bar and reflux condenser, heat benzyl (*S*, *E*)-(2,4-diphenylbut-3-en-1-yl)carbamate (**29**) (0.1 mmol, 35.7 mg) to reflux in 6 M HCl (0.5 mL) with stirring until TLC shows complete consumption of starting material. After reaction, basify the reaction system with an aqueous solution of NaOH (2 M) to pH 10-12. Extract the reaction with DCM (3 x 10 mL). The combined organic layers were dried ($MgSO_4$), filtered, evaporated, and purified by chromatography on silica gel (DCM : MeOH = 10:1), the title compound was obtained as a colorless oil in 75% yield (16.8 mg). ^1H NMR (400 MHz, $CDCl_3$) δ 7.36 – 7.30 (m, 2H), 7.29 – 7.23 (m, 2H), 7.21 – 7.06 (m, 6H), 6.47 (d, *J* = 15.8 Hz, 1H), 6.19 (dd, *J* = 15.8, 8.4 Hz, 1H), 4.81 (s, 2H), 3.56 (q, *J* = 7.8 Hz, 1H), 2.90 (d, *J* = 7.7 Hz, 2H); ^{13}C NMR (101 MHz, $CDCl_3$) δ 140.6, 136.8, 132.6, 129.4, 128.9, 128.5, 127.7, 127.6, 127.2, 126.5, 50.0, 45.2; IR (film): ν (cm^{-1}) 3024, 2920, 2852, 1599, 1494, 1453, 1379, 1311, 1029, 963, 847, 745, 692, 537; HR-MS (ESI) m/z calcd for $C_{16}H_{18}N$ [$M+H^+$] 224.1434, found 224.1435; $[\alpha]_D^{24.6}$ = -4.7 (c = 0.1, $CHCl_3$); HPLC conditions: AD-H column, hexane/2-propanol = 90/10, flow rate = 1.0 mL/min, λ = 254 nm, t_R = 8.6 min (major), t_R = 9.8 min (minor), 96:4 er.

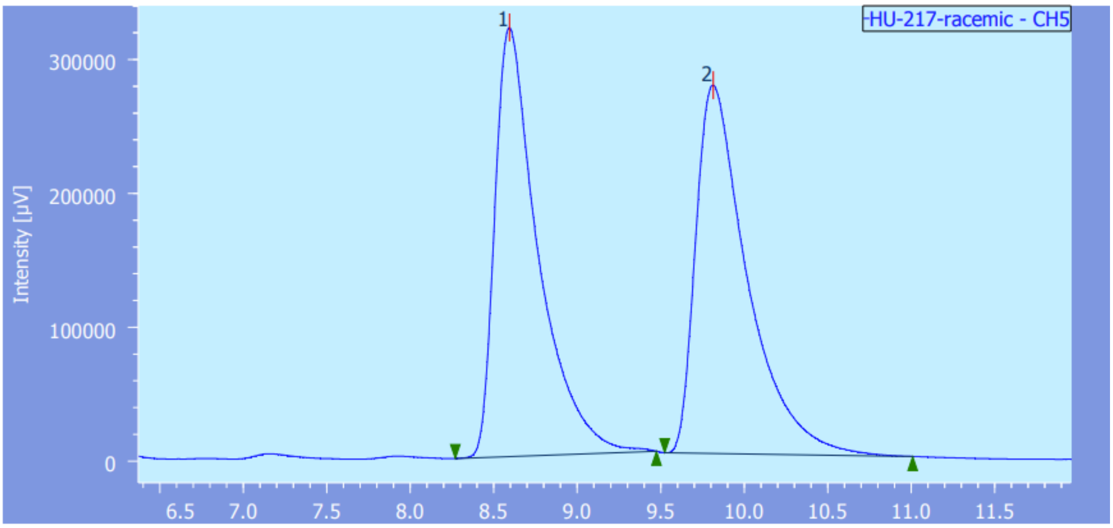

Decision

#	Peak Name	CH	tR [min]	Area [µV·sec]	Height [µV]	Area%	Height%	Quantity	NTP	Resolution	Symmetry Factor	Warning
1	Unknown	5	8.593	5790204	320332	49.431	53.800	N/A	6161	2.530	1.933	
2	Unknown	5	9.813	5923518	275079	50.569	46.200	N/A	5504	N/A	1.949	

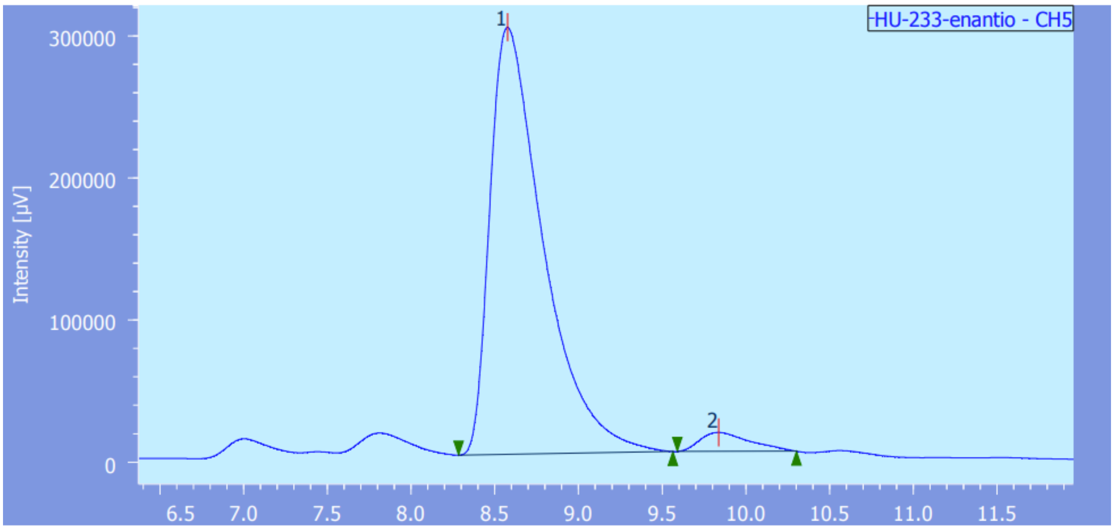

Decision

#	Peak Name	CH	tR [min]	Area [µV·sec]	Height [µV]	Area%	Height%	Quantity	NTP	Resolution	Symmetry Factor	Warning
1	Unknown	5	8.577	6660141	300458	96.012	95.756	N/A	3801	2.276	1.945	
2	Unknown	5	9.837	276658	13315	3.988	4.244	N/A	5051	N/A	1.504	

7. Mechanistic Investigations

7-1. Evidence for stereoconvergent mechanism

All reactions were performed following the standard procedure **GP-A**. (*S*)-*N*-(*p*-Tolylsulfonyl)-2-phenylaziridine and (*R*)-*N*-(*p*-tolylsulfonyl)-2-phenylaziridine were prepared according to the literature procedure and analytical data are in agreement with previously reported values.[2,9] As shown in Scheme S-11, the racemic mixture of phenyl aziridine delivered the same product as the corresponding enantiomers (entries 1-3), thus suggesting that the major enantiomer of the product was dictated by the stereochemistry of the ligand **L1**. Using the opposite enantiomer of the ligand, **-L1**, the opposite enantiomer of the product was observed (entry 4). Enantioenriched phenyl aziridines furnished the corresponding racemic products when 4,4'-di-*tert*-butyl-2,2'-bipyridine was used as ligand (entries 5 and 6). These results demonstrate the stereoconvergent nature of this transformation.

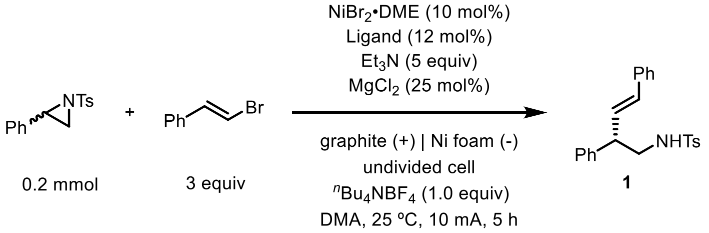

Entry	2-phenyl-1-tosylaziridine	Ligand	Yield of **1** (%)	er of **1**
1	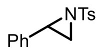	**L1**	73	96:4
2	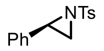	**L1**	74	96:4
3	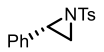	**L1**	71	96:4
4	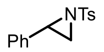	**-L1**	73	4:96
5	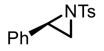	dtbbpy	52	50:50
6	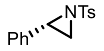	dtbbpy	50	50:50

Scheme S-11. Evidence for stereoconvergent mechanism.

7-2. Oxidative addition: competition experiments

7-2-1. Preparation and characterization of [4,4′-di(tert-buty)-1,1′-bipyridine][2-phenylethen-1-yl]nickel(II) bromide 47

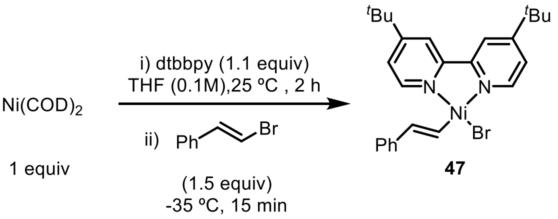

Scheme S-12. Preparation of **47**.

A flame dried 10 mL Schlenk flask equipped with a stirring bar was charged with $Ni(COD)_2$ (0.20 mmol, 1 equiv) in a N_2-filled glovebox. 4,4′-di(*tert*-Buty)-1,1′-bipyridine (0.22 mmol, 1.1 equiv)

was added under a flow of N_2, and the solids were dissolved in 2 mL of SPS-quality THF. The resulting solution was stirred for 2 hours at 25 °C. After this time, the mixture was cooled down at -35 °C in an acetonitrile/dry ice bath and *β*-bromostyrene (0.30 mmol, 1.5 equiv) was added. The resulting mixture was stirred additional 15 minutes, followed by precipitation with 8 mL of SPS-quality pentane. The suspension was filtered under N_2 and triturated with pentane (2 x 8 mL). The orange solid was dried for 18 hours under high vacuum to give the organonickel complex (57 mg, 56 % yield). The solid was dissolved at -35 °C under N_2 in CD_2Cl_2 (degassed and dried over P_2O_5). The ^{1}H-NMR spectrum was recorded immediately after preparing the sample.

1. *Some water that condensed in the walls of the tube is observed in the spectrum as a broad singlet at 1.3 ppm.*
2. *^{1}H-NMR spectrum shows the diamagnetic character of the Ni-complex, that in combination with the desymmetrization of the signals of the bipyridine endorse a square-planar geometry.*

^{1}H NMR (500 MHz, CD_2Cl_2) δ 9.25 (d, J = 6.0 Hz, 1H), 8.96 (d, J = 7.7 Hz, 2H), 8.43 (d, J = 6.1 Hz, 1H), 7.86 (s, 1H), 7.81 (s, 1H), 7.52 (d, J = 5.7 Hz, 1H), 7.47 (d, J = 7.7 Hz, 1H), 7.27 (t, J = 7.3 Hz, 2H), 7.07 (t, J = 7.4 Hz, 1H), 6.83 (d, J = 9.2 Hz, 1H), 6.03 (d, J = 9.3 Hz, 1H), 1.42 (s, 9H), 1.34 (s, 9H). IR (film): ν (cm-1) 2952, 2923, 2876 (t-Bu C-H υ), 1611 (C=C olefin υ), 1546, 1483, 1405 (aromatic C-C υ), 1251, 1022 (aromatic C-H δ), 848, 835 (*t*-Bu C-H δ).

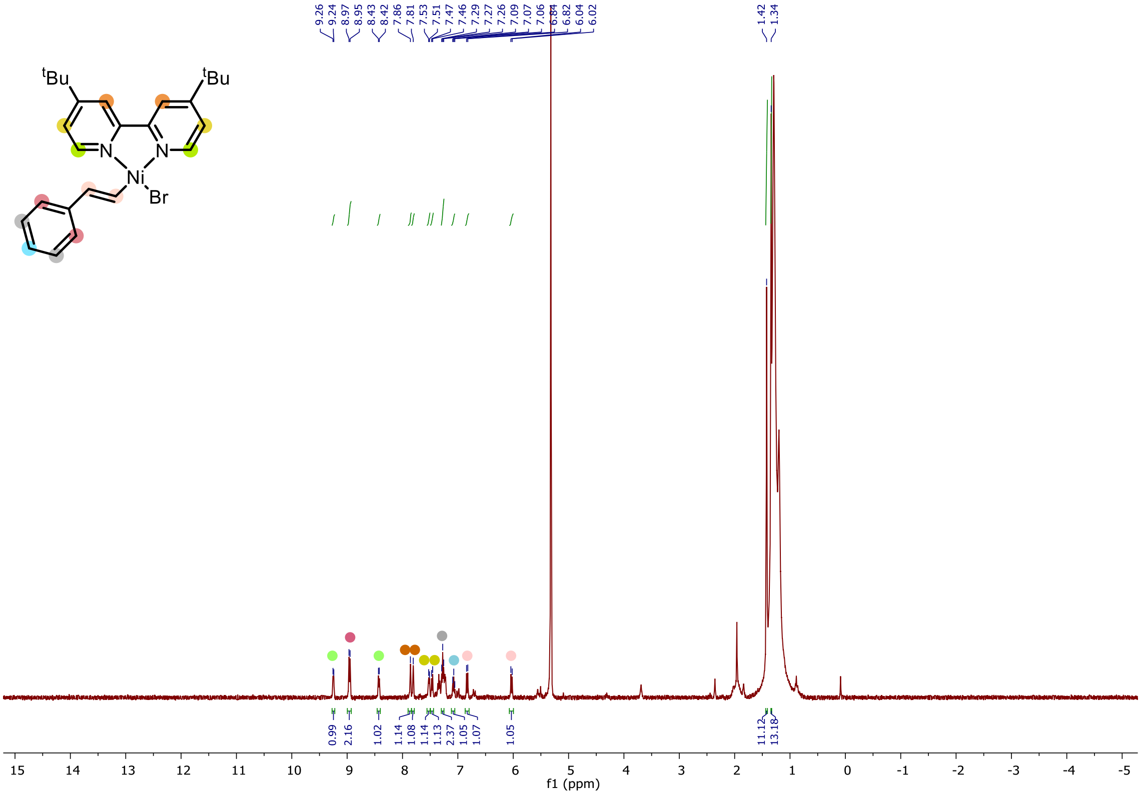

Figure S-3. ^{1}H NMR of [4,4'-di(*tert*-buty)-1,1'-bipyridine][2-phenylethen-1-yl]nickel(II) bromide.

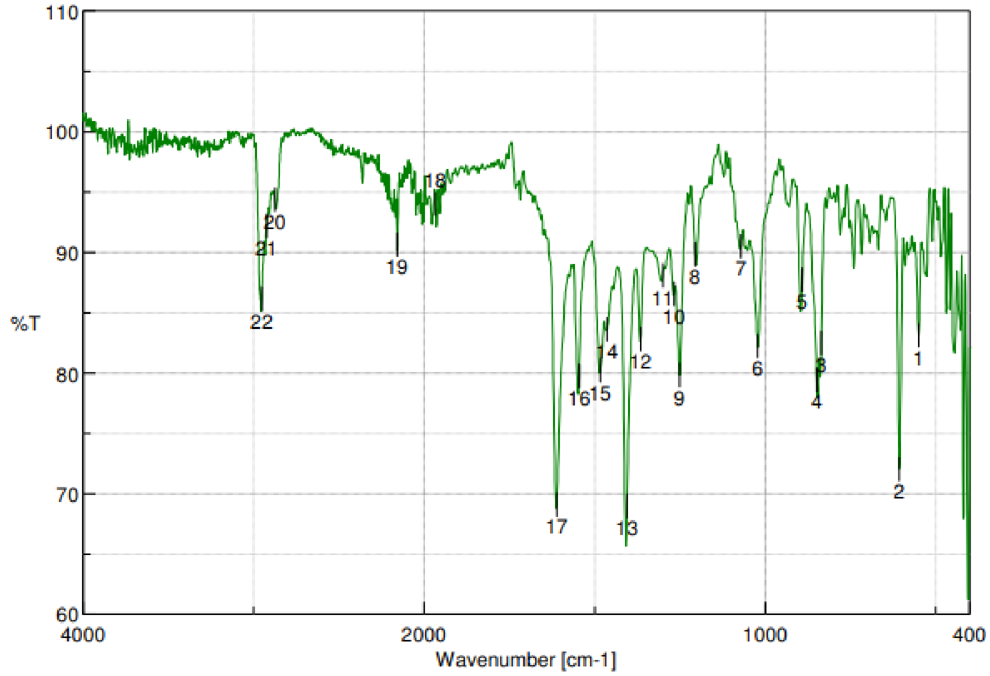

Figure S-4. IR of [4,4'-di(*tert*-buty)-1,1'-bipyridine][2-phenylethen-1-yl]nickel(II) bromide.

7-2-2. Oxidative addition competition experiment

In order to study the competition between alkenyl bromides and aziridines for oxidative addition onto Ni(0), an experiment employing excess of both substrates in the presence of 1 equiv of Ni(0) was carried out. The reaction mixture was analyzed by ^{1}H-NMR spectroscopy clearly revealing the formation of alkenyl-Ni(II) complex **47** as per comparison with the independently prepared sample depicted above. The remaining aziridine was quantified by comparison with 1,3,5-trimethoxybenzene as internal standard. No appreciable consumption of 2-phenyl-1-tosylaziridinecould be detected.

Oxidative addition competition experiment with (dtbbpy)Ni(0):

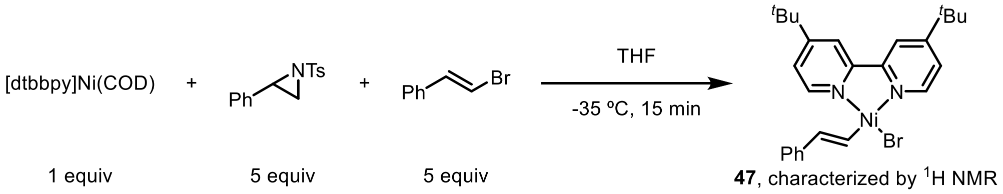

Scheme S-13. Oxidative addition competition experiment with (dtbbpy)Ni(0), 2-phenyl-1-tosylaziridine, and *β*-bromostyrene.

A flame dried 10 mL Schlenk flask equipped with a stir bar was charged with $Ni(COD)_2$ (0.10 mmol, 1 equiv) in a N_2-filled glovebox. 4,4'-di(*tert*-buty)-1,1'-bipyridine (0.10 mmol, 1 equiv) was added under a flow of N_2, and the solids were dissolved in 2 mL of SPS-quality THF. The resulting solution was stirred for 2 hours at 25 °C. After this time, the mixture was cold down at -35 ºC in an acetonitrile:dry ice bath. Then, a solution of *β*-bromostyrene (0.5 mmol, 5 equiv), 2-phenyl-1-tosylaziridine (0.5 mmol, 5 equiv) and 1,3,5-trimethoxybenzene (0.1 mmol, 1 equiv) in 1 mL of SPS-quality THF was added. The resulting mixture was stirred additional 15 minutes and concentrated under high vacuum. The residue was dissolved at -35 ºC under N_2 in CD_2Cl_2 (degassed and dried over P_2O_5). The ^{1}H-NMR spectrum was recorded immediately after preparing the sample.

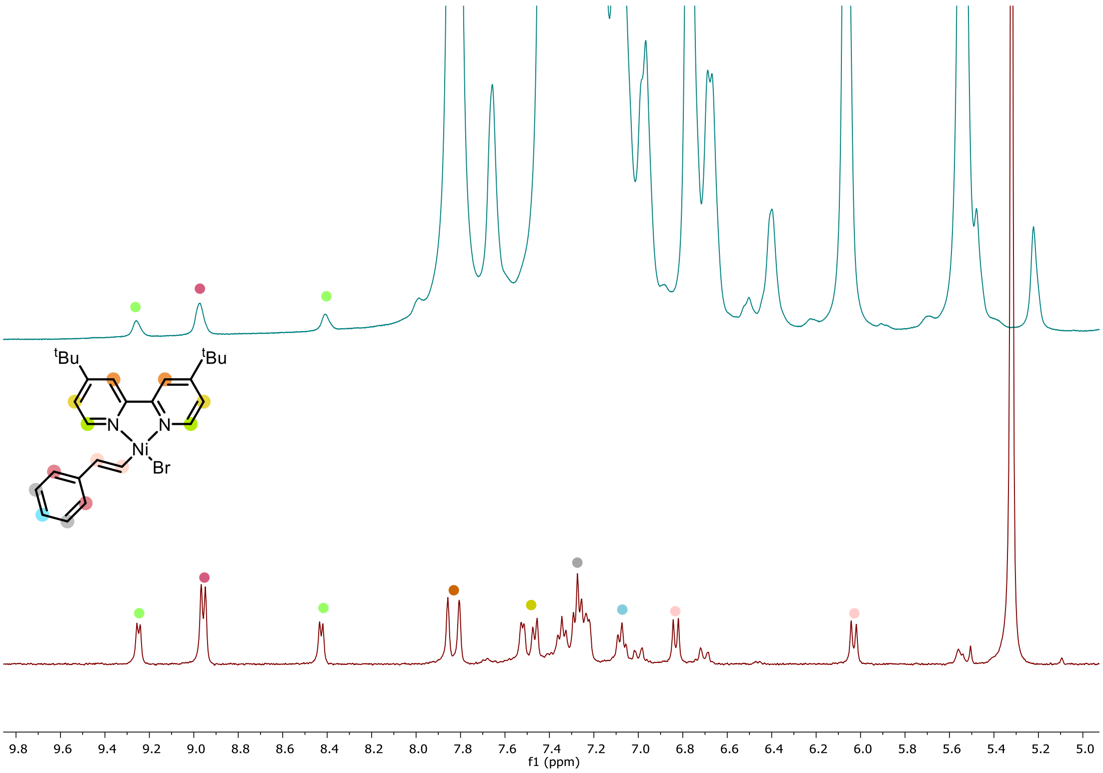

Figure S-5. Evidence (^{1}H NMR) for the oxidative addition of *β*-bromostyrene to (dtbbpy)Ni(0).

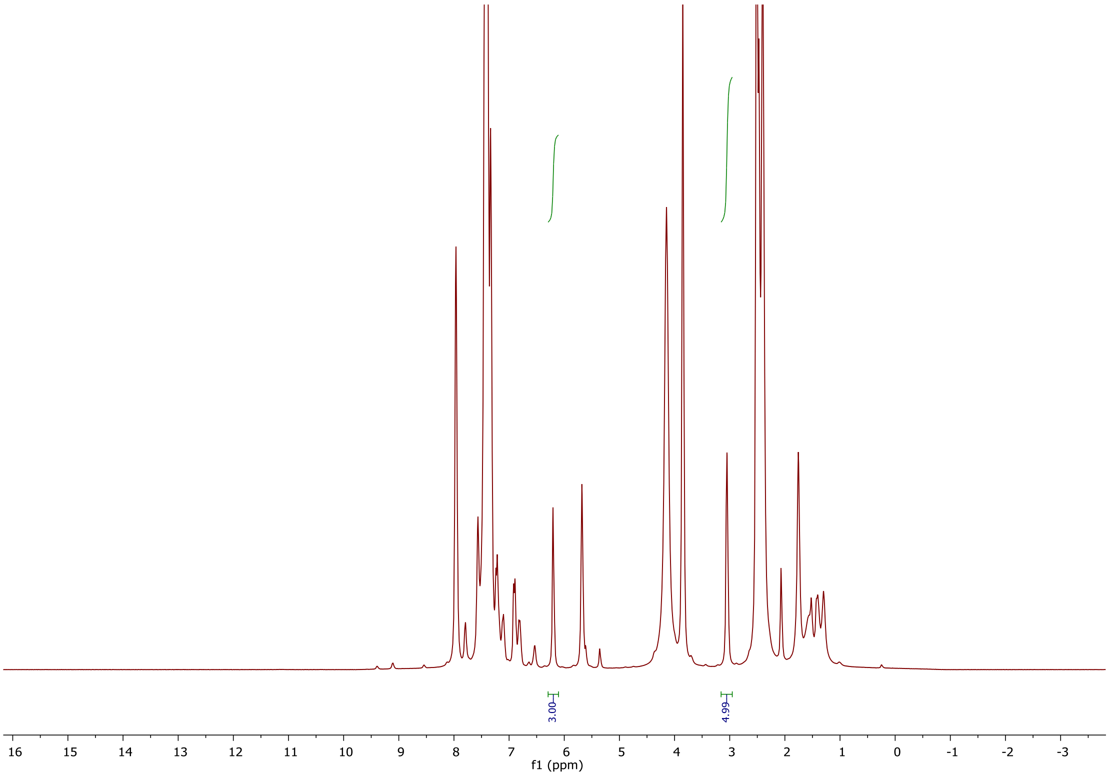

Figure S-6. Evidence (^{1}H NMR) for no consumption of 2-phenyl-1-tosylaziridine in oxidative addition competition experiment with (dtbbpy)Ni(0).

7-3. Radical capture experiments and radical formation studies

7-3-1. Radical capture experiment

In order to investigate whether this stereoconvergent cross-coupling reaction proceeds via a radical intermediate, we studied the reaction in the presence of 2.0 equivalents of radical quencher 2,2,6,6-tetramethyl-1-piperidinyloxy (TEMPO) (Scheme S-14). The reaction was performed following the standard procedure **GP-A**, and the reaction mixture was analyzed by HR-MS after reaction. It was found that the reaction was completely suppressed by adding TEMPO (2.0 equiv) and the TEMPO-benzyl adduct **48** could be detected by HR-MS (Figure S-7), which suggests that a benzyl radical derived from the aziridine is generated under the applied conditions.

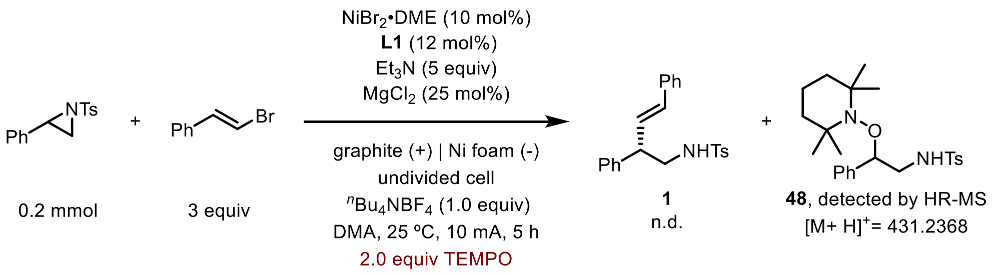

Scheme S-14. Radical capture experiment with TEMPO under standard reaction conditions.

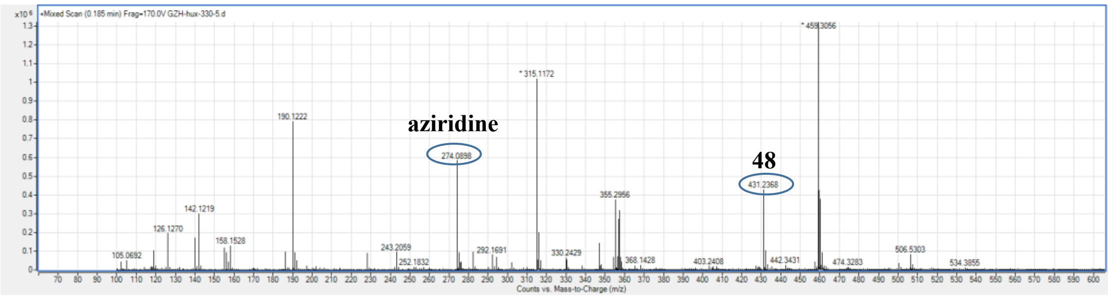

Figure S-7. HR-MS for radical capture experiment under standard reaction conditions.

7-3-2. Radical formation studies under electrolytic conditions

In order to study the possible pathway for the generation of benzyl radical, we carried out the experiments shown in Scheme S-15. All reactions were performed following the standard procedure **GP-A** except for the absence of alkenyl bromide. The reaction mixtures were analyzed by HR-MS after the indicated time. It was found that TEMPO-benzyl adduct **48** could still be detected when the reaction was performed in the absence of $NiBr_2$•DME or $MgCl_2$ (Figure S-8 and S-9). However, when neither $MgCl_2$ nor $NiBr_2$•DME were present, **48** could not be found in the reaction mixture (Figure S-10). These results indicated that the direct single electron reductive activation of aziridine was unlikely, and the benzyl radical could be generated through the formation and subsequent reduction of β-halo-sulfonamide intermediates. A single electron reduction of the β-halo-sulfonamides could occur directly at the cathode since the TEMPO adduct **48** can be formed in the absence of the $NiBr_2$•DME (Scheme S-15, entry 1).

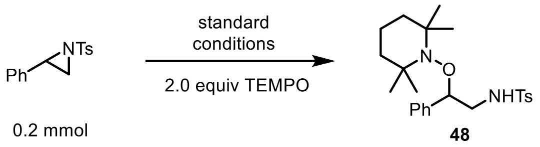

Entry	Variation from standard condition	Result
1	w/o $NiBr_2$•DME	**48** was detected by HR-MS
2	w/o $MgCl_2$	**48** was detected by HR-MS
3	w/o $NiBr_2$•DME, $MgCl_2$	**48** was not detected

Scheme S-15. Radical formation studies under electrolytic conditions.

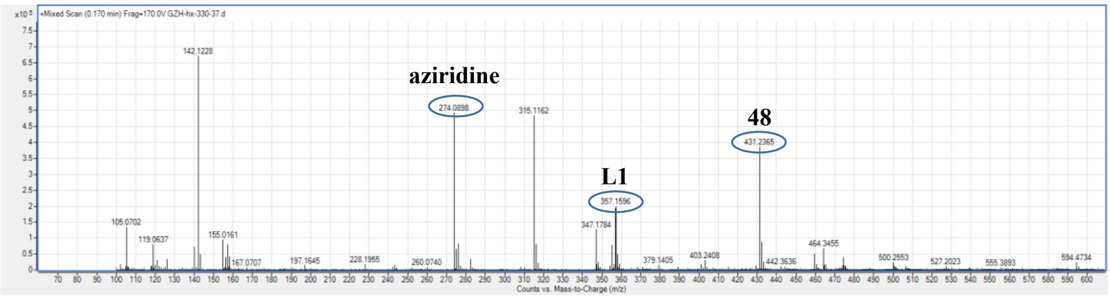

Figure S-8. HR-MS for radical formation studies without $NiBr_2$•DME (Scheme S-15, entry 1).

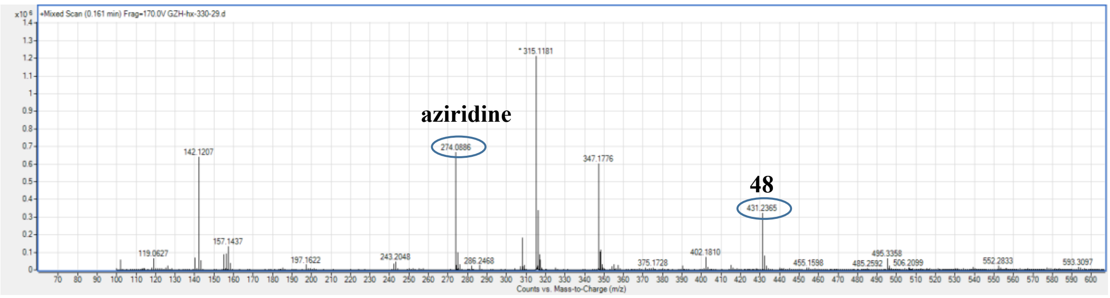

Figure S-9. HR-MS for radical formation studies without $MgCl_2$ (Scheme S-15, entry 2).

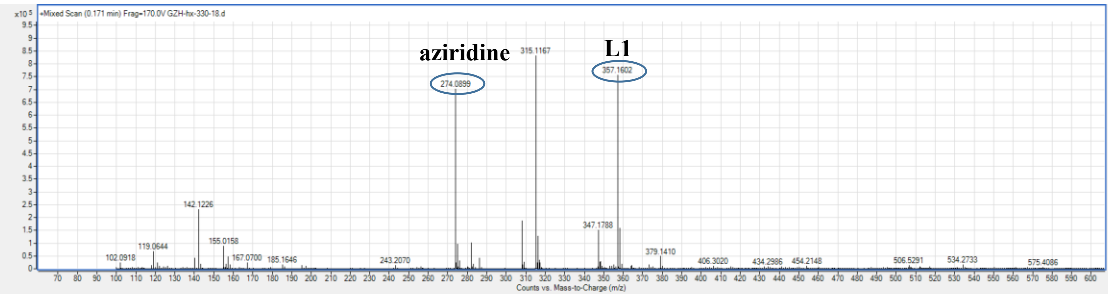

Figure S-10. HR-MS for radical formation studies without $NiBr_2$•DME and $MgCl_2$ (Scheme S-15, entry 3).

7-3-3. Radical formation studies using *β*-halo-sulfonamides with stoichiometric Ni(COD)$_2$/L1

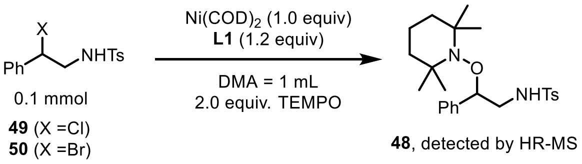

Scheme S-16. Radical formation studies using **49** or **50** with stoichiometric Ni(COD)$_2$/**L1**.

N-(2-Chloro-2-phenylethyl)-4-methylbenzenesulfonamide (**49**) and *N*-(2-bromo-2-phenylethyl)-4-methylbenzenesulfonamide (**50**) were prepared according to the literature procedure and analytical data are in agreement with previously reported values.[10] In a N_2-filled glovebox, a flame dried 5 mL Schlenk flask equipped with a stirring bar was charged with Ni(COD)$_2$ (0.10 mmol, 1equiv), **49**

or **50** (0.10 mmol, 1equiv), **L1** (0.12 mmol, 1.2 equiv), TEMPO (0.20 mmol, 2 equiv) and DMA (1 mL). The resulting mixture was stirred for 24 hours at 25 ºC, and the reaction mixture was analyzed by HR-MS after the indicated time. We found that the reaction of *β*-halo-sulfonamides **49** or **50** with stoichiometric Ni(COD)$_2$/**L1** also delivered the TEMPO adduct **48** (Figure S-11 and S-12), indicating that Ni^0**L1** species can also reduce the *β*-halo-sulfonamide to the benzyl radical.

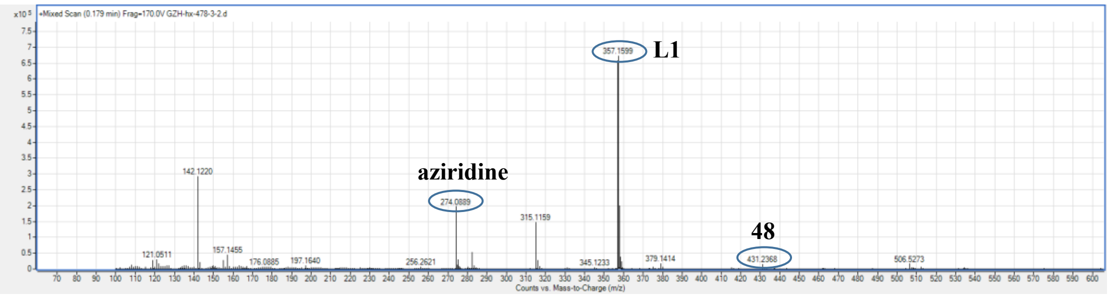

Figure S-11. HR-MS for radical formation studies using **49** with stoichiometric Ni(COD)$_2$/**L1**.

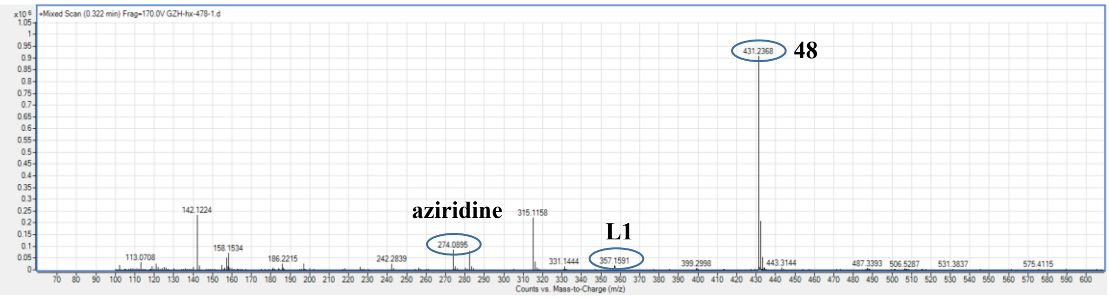

Figure S-12. HR-MS for radical formation studies using **50** with stoichiometric Ni(COD)$_2$/**L1**.

7-4. Cyclic voltammetry studies

Cyclic voltammetry experiments were performed in a three-electrode cell connected under nitrogen at room temperature. A working glass carbon electrode, platinum wire counter electrode, Ag|AgCl reference electrode were employed. Anhydrous degassed DMA (10 mL) containing 1.0 mmol $^{n}Bu_4NBF_4$ were poured into the electrochemical cell in all experiments. The concentration of compounds is 3 mM. The scan rate is 100 mV/s. All cyclic voltammograms were normalized by adding 1.0 equiv freshly-sublimed ferrocene and collecting a new voltammogram. The ½ wave penitential of the Fc/Fc+ peak was identified and set to 0.0 V. Data was analyzed by subtracting a background current prior to identifying the maximum current (Cp) and determining the potential (Ep/2) at half this value (Cp/2). [12]

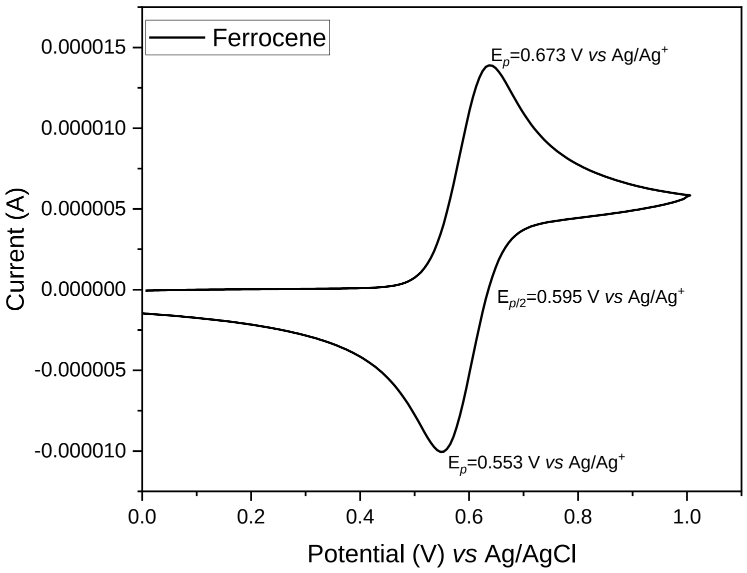

Figure S-13. Cyclic voltammograms of ferrocene.

As shown in Figure S-14 and Figure S-15, a reductive peak of **49** in DMA was observed at -2.74 V (*vs* Fc/Fc^+), and the reductive half-peak potential of **49** was measured as -2.61 V (*vs* Fc/Fc^+). A reductive peak of **50** in DMA was observed at -2.22 V (*vs* Fc/Fc^+), and the reductive half-peak potential of **50** was measured as -2.06 V (*vs* Fc/Fc^+). A reductive peak of 2-phenyl-1-tosylaziridine in DMA was observed at -2.78 V (*vs* Fc/Fc^+), and the reductive half-peak potential of 2-phenyl-1-tosylaziridine was measured as -2.70 V (*vs* Fc/Fc^+). A reductive peak of β-bromostyrene in DMA was observed at -2.69 V (vs Fc/Fc^+), and the reductive half-peak potential of β-bromostyrene was measured as -2.60 V (vs Fc/Fc^+). Two reductive peaks of $NiBr_2$-**L1**[11] in DMA were observed at Ni^{II}/Ni^{I}=-1.35 V (*vs* Fc/Fc^+) and Ni^{I}/Ni^{0}=-2.98 V (*vs* Fc/Fc^+), and the reductive half-peak potential of $NiBr_2$-**L1** was measured as Ni^{II}/Ni^{I}=-1.23 V (*vs* Fc/Fc^+) and Ni^{I}/Ni^{0}=-2.62 V (*vs* Fc/Fc^+).

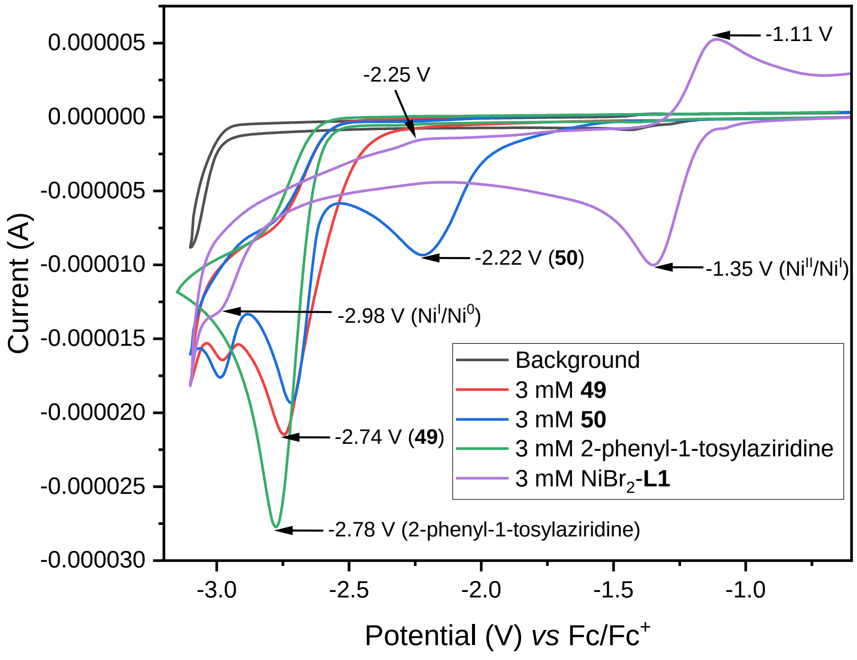

Figure S-14. Cyclic voltammograms of 2-phenyl-1-tosylaziridine, chlorosulfonamide **49**, bromosulfonamide **50**, and $NiBr_2$-**L1** in DMA at 100 mV/s scan rate.

In order to study whether Ni(I) can undergo oxidative addition with the alkenyl bromides, we measured the CV of $NiBr_2$-**L1** in the presence of 1.0 equivalent *β*-bromostyrene (Figure S-15). Some new reductive peaks appeared in the mixture of $NiBr_2$-**L1** and *β*-bromostyrene. This result suggested that the oxidative addition of alkenyl bromide to Ni(I) is also possible. Since the operating potential of Ni foam cathode (-3.05 *vs* Fc/Fc^+, -2.45 V *vs* Ag/AgCl, see section 7-8) is competent to reduce Ni(I) to Ni(0) ($E_{1/2}$ = -2.62 V *vs* Fc/Fc^+, -2.02 V *vs* Ag/AgCl), we hold that both $Ni^0/Ni^{II}/Ni^{III}/Ni^I$ and $Ni^I/Ni^{II}/Ni^{III}$ redox cycles are possible.

In order to study whether *β*-halo-sulfonamides can be activated by electrochemically generated Ni(I) species, we tested the reduction potential of $NiBr_2$-**L1** in the presence of 1.0 equivalent *β*-halo-sulfonamide **49** or **50** (Figure S-16 and Figure S-17). The cyclic voltammetry of the mixtures was different from the corresponding individual components. These results indirectly indicate that there may exist interaction between Ni(I) and *β*-halo-sulfonamides. Since the reduction potential of *β*-halo-sulfonamide **49** (-2.61 V *vs* Fc/Fc^+) or **50** (-2.06 V *vs* Fc/Fc^+) is more negative than that of Ni^{II}/Ni^I (-1.23 V *vs* Fc/Fc^+), they cannot be reduced by Ni(I), but they can be activated based on the cyclic voltammogram studies. We proposed that *β*-halo-sulfonamides might be activated through halogen atom abstraction (HAA) process.

The interaction between the Ni(I) and aziridines was also investigated. We tested the reduction potential of $NiBr_2$-**L1** in the presence of 1.0 equivalent 2-phenyl-1-tosylaziridine (Figure S-18). The cyclic voltammetry of the mixture did not change compared with the corresponding individual components. This result demonstrated that there is no interaction between the nickel catalyst and aziridine.

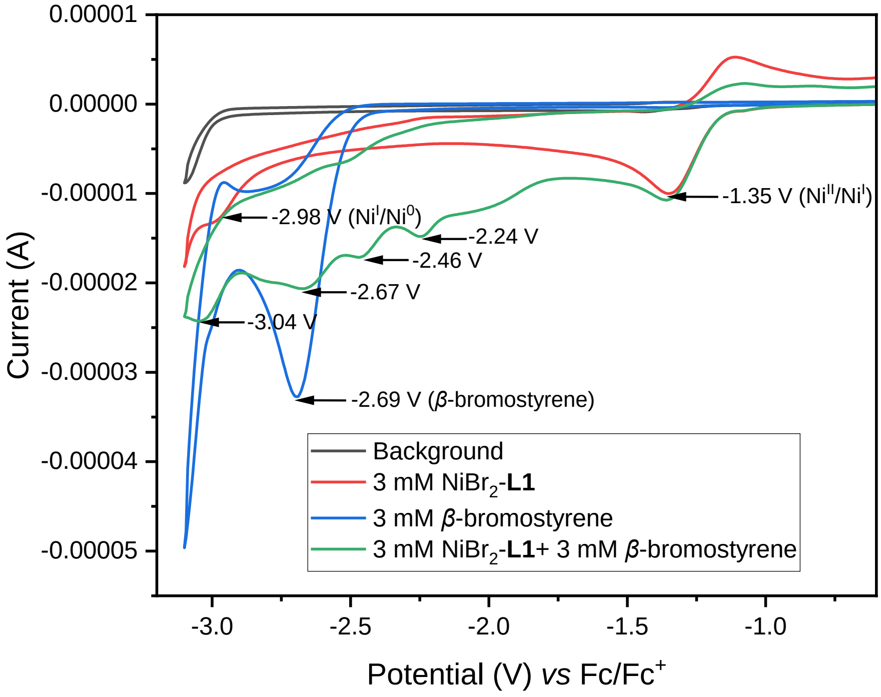

Figure S-15. Cyclic voltammograms of *β*-bromostyrene, $NiBr_2$-**L1**, the mixture of *β*-bromostyrene and $NiBr_2$-**L1** in DMA.

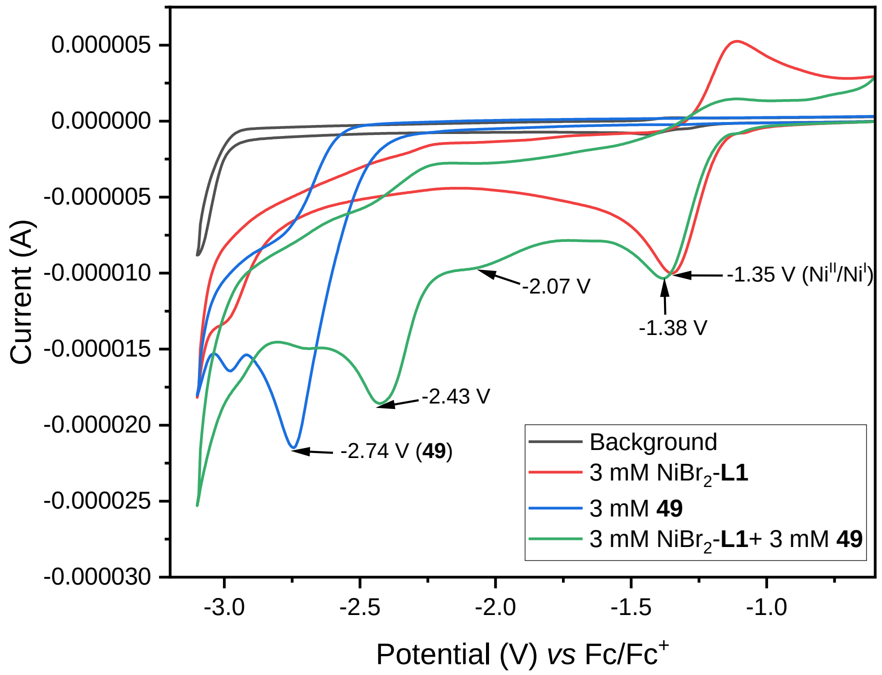

Figure S-16. Cyclic voltammograms of **49**, NiBr₂-**L1**, the mixture of **49** and NiBr₂-**L1** in DMA.

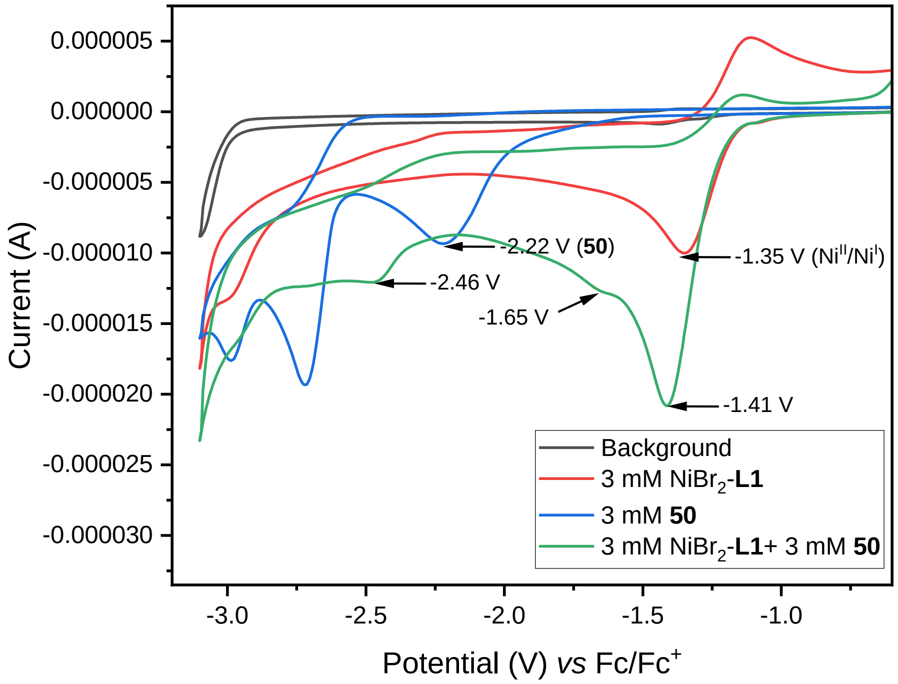

Figure S-17. Cyclic voltammograms of **50**, NiBr₂-**L1**, the mixture of **50** and NiBr₂-**L1** in DMA.

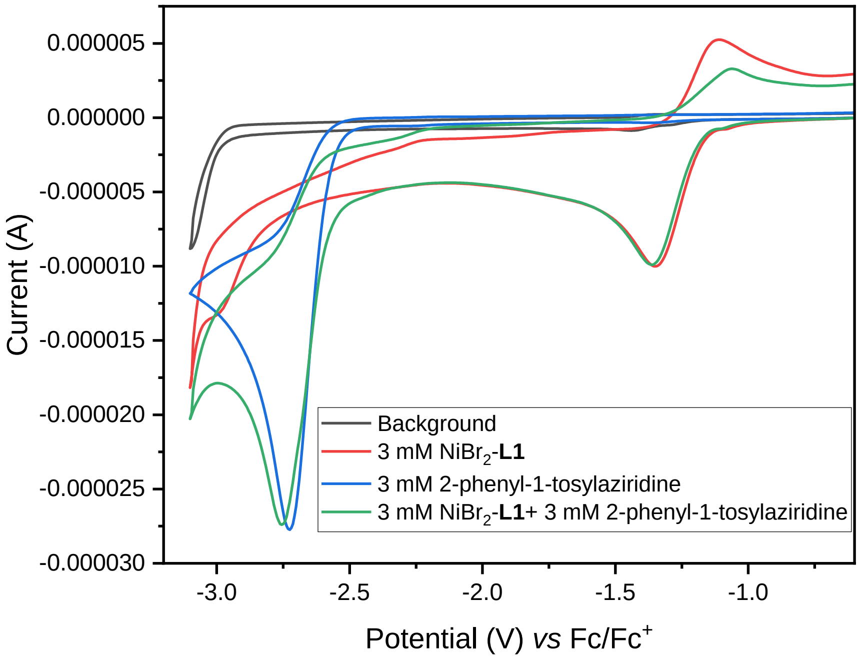

Figure S-18. Cyclic voltammograms of 2-phenyl-1-tosylaziridine, $NiBr_2$-**L1**, the mixture of 2-phenyl-1-tosylaziridine and $NiBr_2$-**L1** in DMA.

7-5. Intermediate confirmation experiment

All reactions were performed following the standard procedure **GP-A** and stopped after 2 hours. *β*-chloro-sulfonamide **49** can be isolated in 8% yield after 2 h of electrolysis in the absence of alkenyl bromide (Scheme S-17, entry 1) and in 18% yield when both $NiBr_2$•DME and alkenyl bromide are removed from the reaction mixture (Scheme S-17, entry 3), **49** was not detected in the absence of electric current (Scheme S-17, entry 2). *β*-Bromo-sulfonamide **49** can be isolated in 2% yield after 2 h of electrolysis in the absence of alkenyl bromide and $MgCl_2$ (Scheme S-17, entry 4). These results indicated that the aziridine can be activated through nucleophilic halide ring-opening mechanism, likely *in-situ* formed R_3N-HX (X = Cl or Br). Interestingly, neither $MgCl_2$ nor $NiBr_2$•DME seem to contribute to this activation process. Compound **50** is not detected under the standard conditions as a result of its facile reduction compared to **49** under the utilized electrochemical conditions.

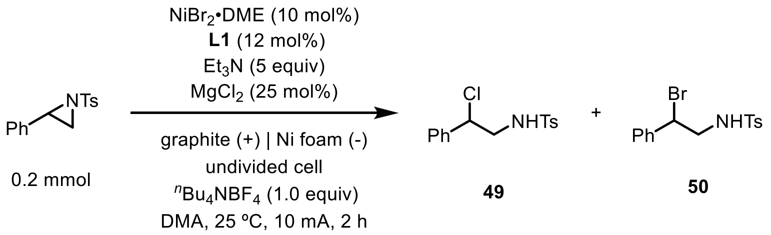

Entry	Variation from standard condition	Yield of **49**[a]	Yield of **50**[a]
1	None	8%	not detected[b]
2	w/o electric current	not detected[b]	not detected[b]
3	w/o $NiBr_2$•DME	18%	not detected[b]
4	w/o $MgCl_2$	not detected[b]	2%

[a]isolated yield, [b]detected by HR-MS.

Scheme S-17. Intermediate confirmation experiment.

7-6. Reactivity of *β*-halo-sulfonamide

All reactions were performed following the standard procedure **GP-A**. When *β*-chloro-sulfonamide (**49**) or *β*-bromo-sulfonamide (**50**) were subjected to the standard reaction set up, the cross-coupled product **1** was obtained in 53% and 68% yield (Scheme S-18), respectively. In addition, subjecting catalytic amounts of **49** or **50** (10-30%) to different quantities of 4-(1-tosylaziridin-2-yl)phenyl acetate(90-70%), generated the corresponding products **1** and **4** in consistent yields compared to those obtained when the independently used aziridine substrates were employed. The enantiomeric ratios were identical to those obtained when the aziridines were used as starting materials (Scheme S-19). Specifically, 0.1 equiv of **49** in combination with 0.9 equiv 4-(1-tosylaziridin-2-yl)phenyl acetate delivered product **1** in 7% yield (69% with respect to **49**) and product **4** in 59% yield (66% with respect to 4-(1-tosylaziridin-2-yl)phenyl acetate). Similar results were obtained for experiments using 0.2 or 0.3 equivalents of the alkyl halides, which support the idea of *β*-halo-sulfonamides as productive intermediates in these transformations.

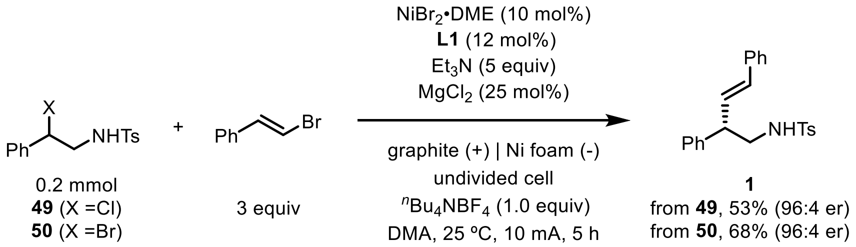

Scheme S-18. Reaction with stoichiometric amount of *β*-halo-sulfonamide.

A. Reaction with catalytic amount of **49**.

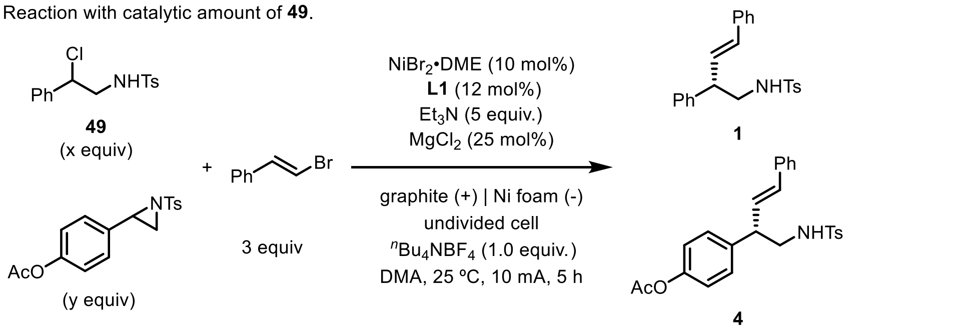

Entry	**49**	4-(1-tosylaziridin-2-yl)phenyl acetate	Yield and er of **1**	Yield and er of **4**
1	0.02 mmol (0.1 equiv)	0.18 mmol (0.9 equiv)	7%[a], 69%[b](96:4 er)	59%[c], 66%[d] (96:4 er)
2	0.04 mmol (0.2 equiv)	0.16 mmol (0.8 equiv)	14%[a], 68%[b] (96:4 er)	52%[c], 65%[d] (96:4 er)
3	0.06 mmol (0.3 equiv)	0.14 mmol (0.7 equiv)	20%[a], 68%[b] (96:4 er)	46%[c], 65%[d] (96:4 er)

[a]quantitative yield relative to **49**, [b]substrate **49** based yield, [c]quantitative yield relative to aziridine, [d]aziridine based yield.

B. Reaction with catalytic amount of **50**.

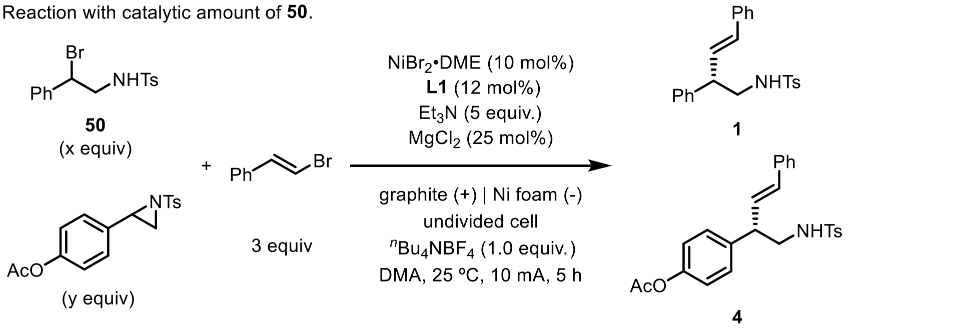

Entry	**50**	4-(1-tosylaziridin-2-yl)phenyl acetate	Yield and er of **1**	Yield and er of **4**
1	0.02 mmol (0.1 equiv)	0.18 mmol (0.9 equiv)	7%[a], 73%[b] (96:4 er)	59%[c], 66%[d] (96:4 er)
2	0.04 mmol (0.2 equiv)	0.16 mmol (0.8 equiv)	14%[a], 72%[b] (96:4 er)	52%[c], 66%[d] (96:4 er)
3	0.06 mmol (0.3 equiv)	0.14 mmol (0.7 equiv)	22%[a], 72%[b] (96:4 er)	46%[c], 66%[d] (96:4 er)

[a]quantitative yield relative to **50**, [b]substrate **50** based yield, [c]quantitative yield relative to aziridine, [d]aziridine based yield.

Scheme S-19. Reaction with catalytic amount of *β*-halo-sulfonamide.

7-7. Conversion of 2-phenyl-1-tosylaziridine to *β*-halo-sulfonamide with Et_3N-HX

In order to demonstrate that Et_3N-HX might be important for the activation of aziridines, the reactions of 2-phenyl-1-tosylaziridine with catalytic or stoichiometric amounts of Et_3N-HX were carried out (Scheme S-20). In a N_2-filled glovebox, a flame dried 5 mL Schlenk flask equipped with a stirring bar was charged with 2-phenyl-1-tosylaziridine (0.20 mmol, 1equiv), Et_3N-HCl or Et_3N-HBr (10 mol% or 1equiv) and DMA (1 mL). The resulting mixture was stirred for 5 hours at 25 ºC, and the reaction mixture was analyzed by NMR. We found that *β*-chloro-sulfonamide **49** can be detected in 5% (with 10 mol% Et_3N-HCl) and 23% yield (with 1.0 equiv Et_3N-HCl), respectively. By comparison, Et_3N-HBr showed a weaker activation ability than Et_3N-HCl, as *β*-bromo-sulfonamide **50** can be detected in 2% (with 10 mol% Et_3N-HBr) and 6% yield (with 1.0 equiv Et_3N-HBr), respectively. Further studies revealed that the conversion of 2-phenyl-1-tosylaziridine to *β*-halo-sulfonamides can be improved in the presence of catalytic amount of $NiBr_2$•DME/**L1**, as *β*-chloro-sulfonamide **49** can be detected in 30% yield (with 1.0 equiv Et_3N-HCl and 10 mol% $NiBr_2$•DME/12 mol% **L1**) and *β*-bromo-sulfonamide **50** can be detected in 14% yield (with 1.0

equiv Et_3N-HBr and 10 mol% $NiBr_2$•DME/12 mol% **L1**). These results indicated that Et_3N-HX play an important role in the ring-opening process of aziridines, and it also shows some degree of catalytic effect of Ni(II) complexes as Lewis acids in these transformations.

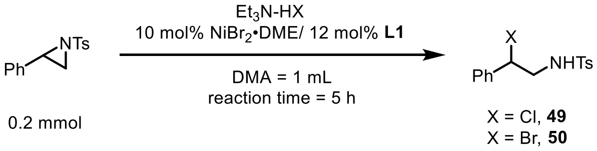

Entry	Et_3N-HX	10 mol% $NiBr_2$•DME/ 12 mol% **L1**	**49** or **50**
1	10 mol% Et_3N-HCl	without	5% **49**
2	1.0 equiv. Et_3N-HCl	without	23% **49**
3	1.0 equiv. Et_3N-HCl	with	30% **49**
4	10 mol% Et_3N-HBr	without	2% **50**
5	1.0 equiv. Et_3N-HBr	without	6% **50**
6	1.0 equiv. Et_3N-HBr	with	14% **50**
7	without	with	not detected

Scheme S-20. Conversion of 2-phenyl-1-tosylaziridine to β-halo-sulfonamide with Et_3N-HX.

7-8. Measurement of cathodic operating potential

The operating potential of cathode was measured by connecting a multimeter. It was found that the operating potential of the Ni foam cathode is -3.05 *vs* Fc/Fc$^+$, -2.45 V *vs* Ag/AgCl, using the same reference electrode for CV studies. The reductive potential of Ni^I/Ni^0 is $E_{1/2}$= -2.62 V *vs* Fc/Fc$^+$, -2.02 V *vs* Ag/AgCl. These results indicated that the cathode is competent to reduce Ni(I) to Ni(0) under the standard reaction conditions. In addition, we also tested the operating potential of Pt plate and Ni plate cathode. The operating potential of Pt plate cathode is -3.76 V *vs* Ag/AgCl, and the operating potential of Ni plate cathode is -4.59 V *vs* Ag/AgCl.

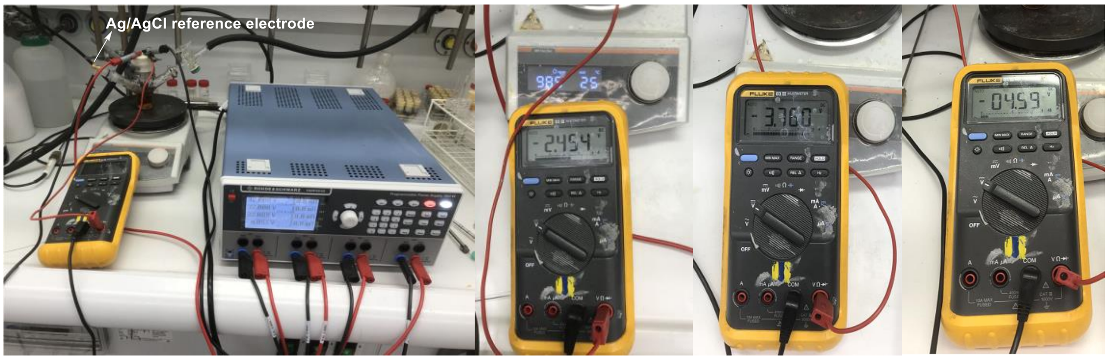

Device for measuring the operating potential of the cathode | Ni foam cathode | Pt plate cathode | Ni plate cathode

Scheme S-21. Measurement of cathodic operating potential with different electrode materials.

8. X-Ray Diffraction Analysis of Compound 1

(Deposition Number 2223639)

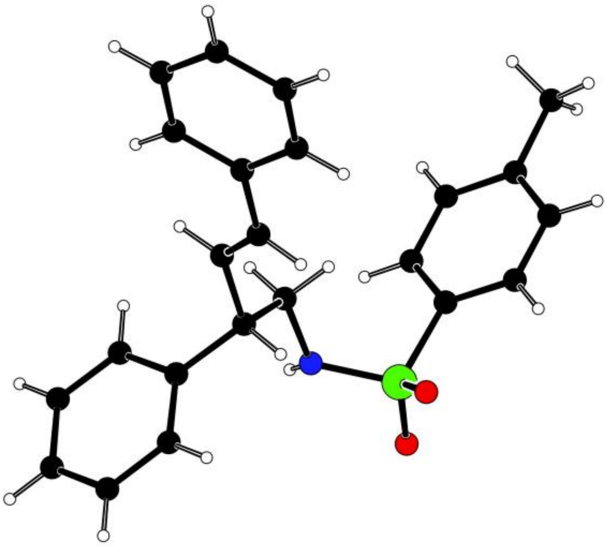

Crystallographic Data

Crystallised from		EtOAc / *n*-hexane
Empirical formula		$C_{23}H_{23}NO_2S$
Formula weight [g mol^{-1}]		377.48
Crystal colour, habit		colourless, needle
Crystal dimensions [mm]		0.05 □ 0.08 □ 0.19
Temperature [K]		160(1)
Crystal system		orthorhombic
Space group		$P2_12_12_1$ (#19)
Z		4
Reflections for cell determination		13469
2*q* range for cell determination [°]		5−154
Unit cell parameters	*a* [Å]	5.76504(5)
	b [Å]	11.37485(15)
	c [Å]	30.1375(3)
	a [°]	90
	b [°]	90
	g [°]	90
	V [Å^3]	1976.31(4)
F(000)		800
D_x [g cm^{-3}]		1.269
m(Cu *K*a) [mm^{-1}]		1.586
Scan type		*w*

$2q_{(max)}$ [°]	153.4
Transmission factors (min; max)	0.699; 1.000
Total reflections measured	20028
Symmetry independent reflections	4107
R_{int}	0.025
Reflections with $I > 2s(I)$	3859
Reflections used in refinement	4107
Parameters refined	249
Final $R(F)$ [$I > 2s(I)$ reflections]	0.0277
$wR(F^2)$ (all data)	0.0730
Weights:	$w = [s^2(F_o^2) + (0.0413P)^2 + 0.3408P]^{-1}$ where $P = (F_o^2 + 2F_c^2)/3$
Goodness of fit	1.034
Final D_{max}/s	0.000
Dr (max; min) [e Å^{-3}]	0.21; -0.18
$s(d_{(C–C)})$ [Å]	0.003 – 0.004

9. References

[1] Huang, C. Y.; Doyle, A. G. Nickel-catalyzed Negishi alkylations of styrenyl aziridines. *J. Am. Chem. Soc.* **2012**, *134*, 9541-9544.

[2] Woods, B. P.; Orlandi, M.; Huang, C. Y.; Sigman, M. S.; Doyle, A. G. Nickel-Catalyzed Enantioselective Reductive Cross-Coupling of Styrenyl Aziridines. *J. Am. Chem. Soc.* **2017**, 139, 5688-5691.

[3] McGhee, A.; Cochran, B. M.; Stenmark, T. A.; Michael, F. E. Stereoselective synthesis of 2,5-disubstituted morpholines using a palladium-catalyzed hydroamination reaction. *Chem. Commun.* **2013**, *49*, 6800-6802.

[4] Cherney, A. H.; Reisman, S. E. Nickel-catalyzed asymmetric reductive cross-coupling between vinyl and benzyl electrophiles. *J. Am. Chem. Soc.* **2014**, *136*, 14365-14368.

[5] Chang, D.; Gu, Y.; Shen, Q. Pd-catalyzed difluoromethylation of vinyl bromides, triflates, tosylates, and nonaflates. *Chem. Eur. J.* **2015**, *21*, 6074-6078.

[6] Pappula, V.; Donthiri, R. R.; Darapaneni, C. M.; Subbarayappa, A. H-β-zeolite catalyzed synthesis of β-bromostyrenes from styrene bromohydrins." *Tetrahedron Lett.* **2014**, *55*, 1793-1795.

[7] Li, K.; Weber, A. E.; Tseng, L.; Malcolmson, S. J. Diastereoselective and Enantiospecific Synthesis of 1,3-Diamines via 2-Azaallyl Anion Benzylic Ring-Opening of Aziridines. *Org. Lett.* **2017**, *19*, 4239-4242.

[8] DeLano, T. J.; Reisman, S. E. Enantioselective Electroreductive Coupling of Alkenyl and Benzyl Halides via Nickel Catalysis. *ACS Catal.* **2019**, *9*, 6751-6754.

[9] Nitelet, A.; Evano, G. A General Copper-Catalyzed Vinylic Halogen Exchange Reaction. *Org. Lett.* **2016**, *18*, 1904-1907.

[10] Ghorai, M. K.; Kumar, A.; Tiwari, D. P. BF_3-OEt_2 mediated highly regioselective S(N)2-type ring-opening of N-activated aziridines and N-activated azetidines by tetraalkylammonium halides. *J. Org. Chem.* **2010**, *75*, 137-151.

[11] Suzuki, N.; Hofstra, J. L.; Poremba, K. E.; Reisman, S. E. Nickel-Catalyzed Enantioselective Cross-Coupling of N-Hydroxyphthalimide Esters with Vinyl Bromides. *Org. Lett.* **2017**, 19 (8), 2150-2153.

[12] Espinoza, E. M; Clark, J. A.; Soliman, J.; Derr, J. B.; Morales, M.; Vullev, V. I. Practical Aspects of Cyclic Voltammetry: How to Estimate Reduction Potentials When Irreversibility Prevails. *J. Electrochem. Soc.* **2019**, *166* (5), H3175-H3187.

10. NMR Spectra

tert-Butyl 5-(1-tosylaziridin-2-yl)-1H-indole-1-carboxylate (S14)

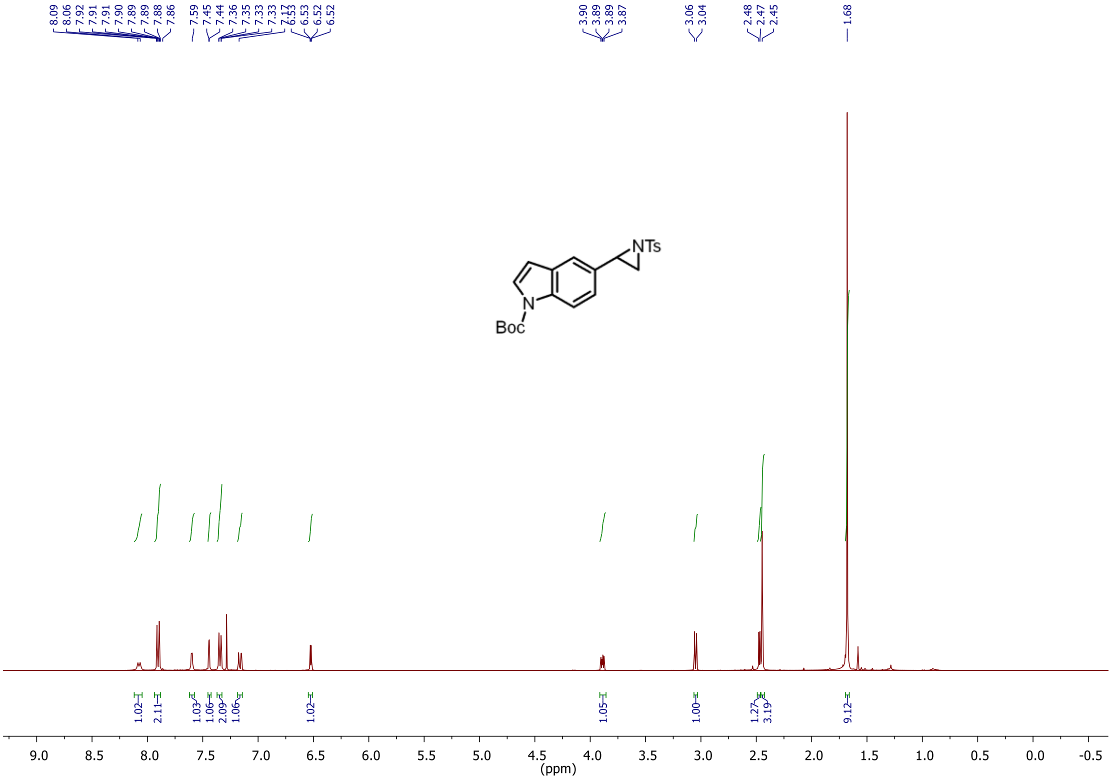

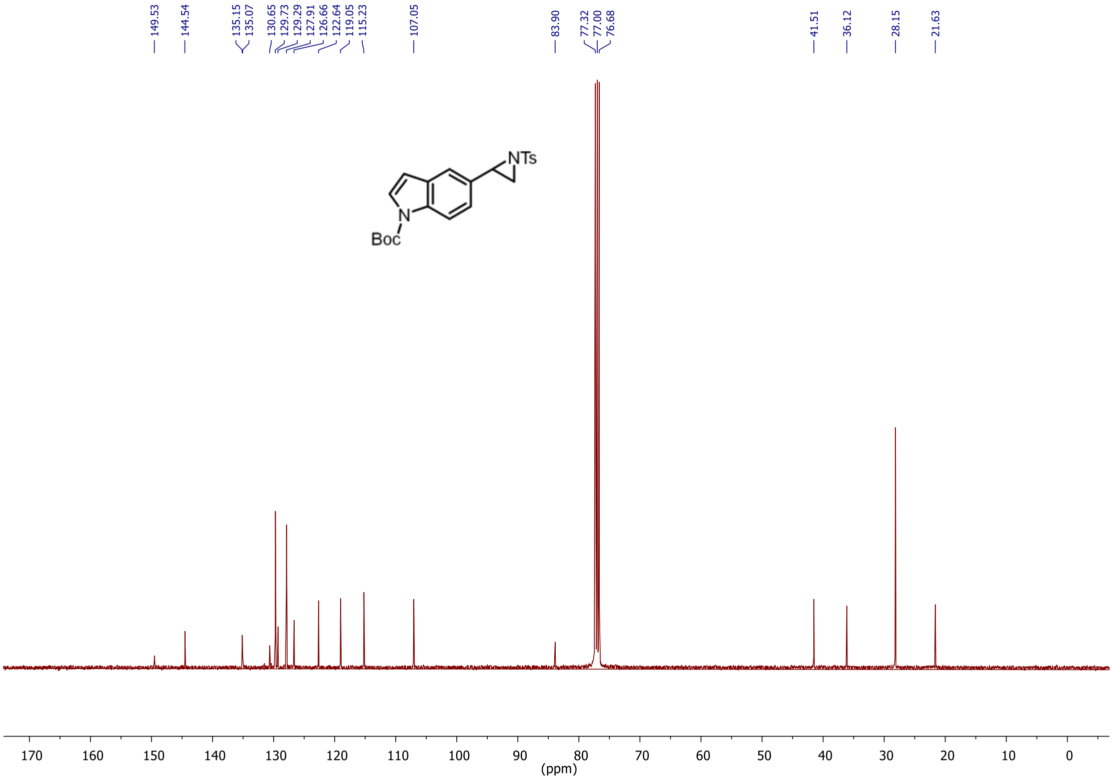

(*R*)-2,5,7,8-Tetramethyl-2-((4R,8R)-4,8,12-trimethyltridecyl)chroman-6-yl 4-(1-tosylaziridin-2-yl)benzoate (S16)

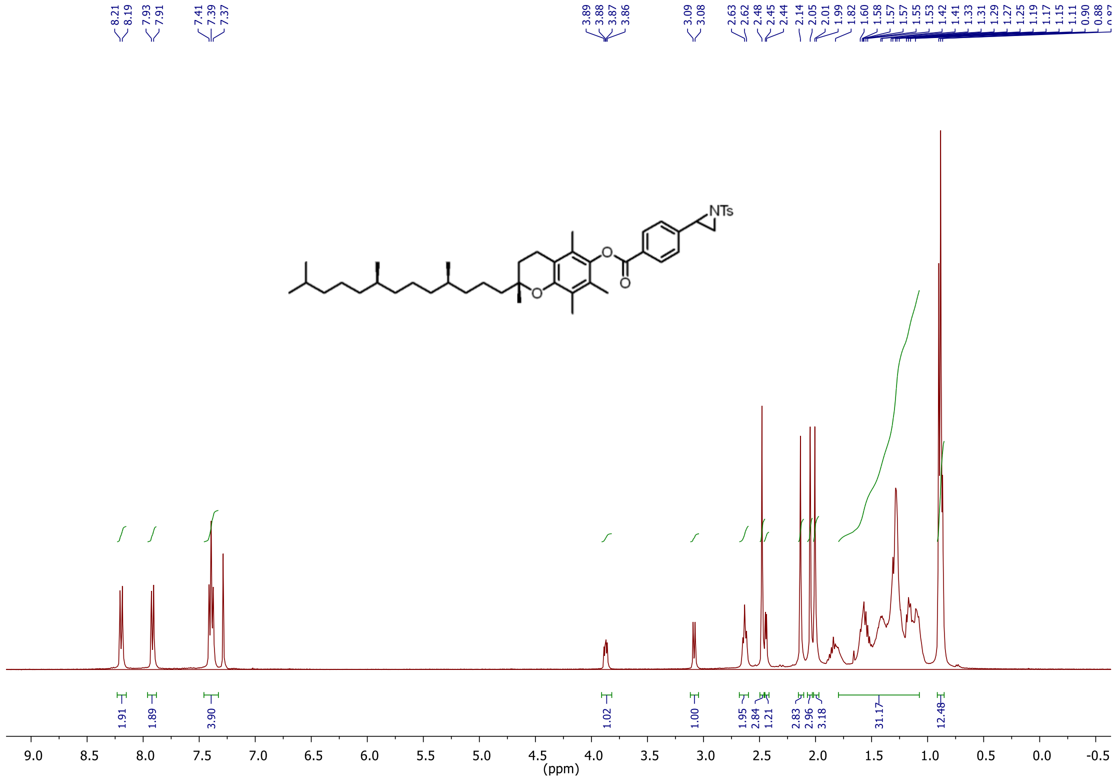

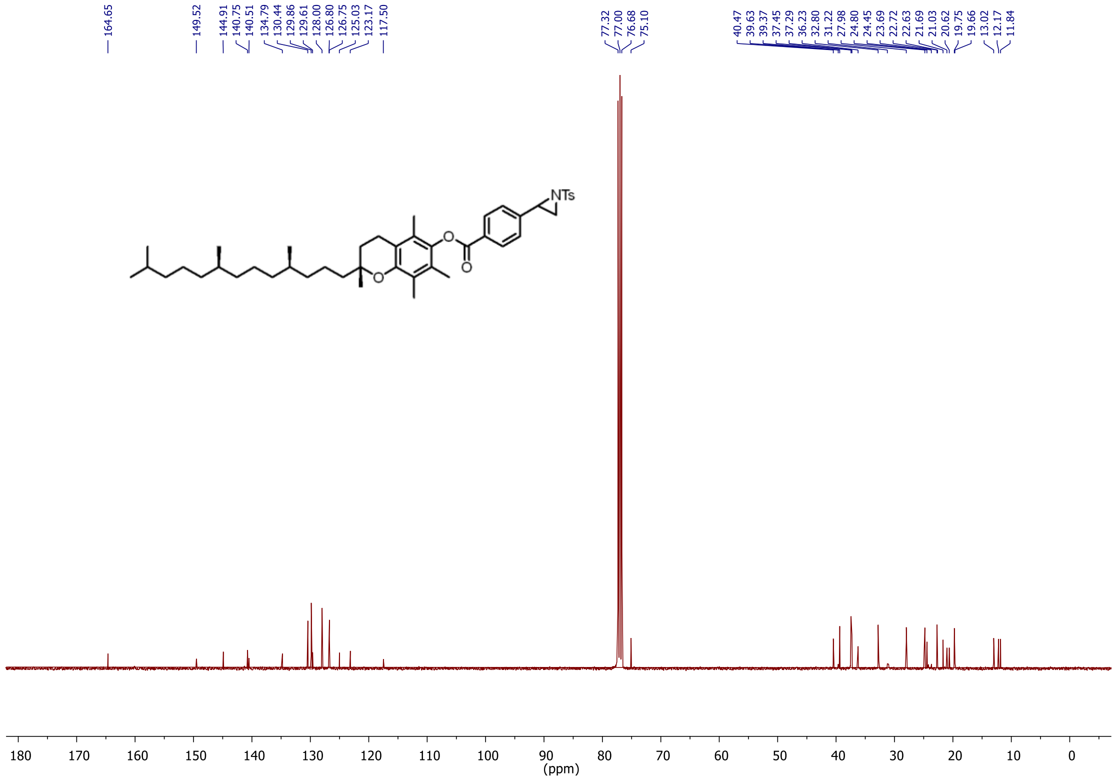

(*E*)-4-(2-Bromovinyl)phenyl (S)-2-(6-methoxynaphthalen-2-yl)propanoate (S34)

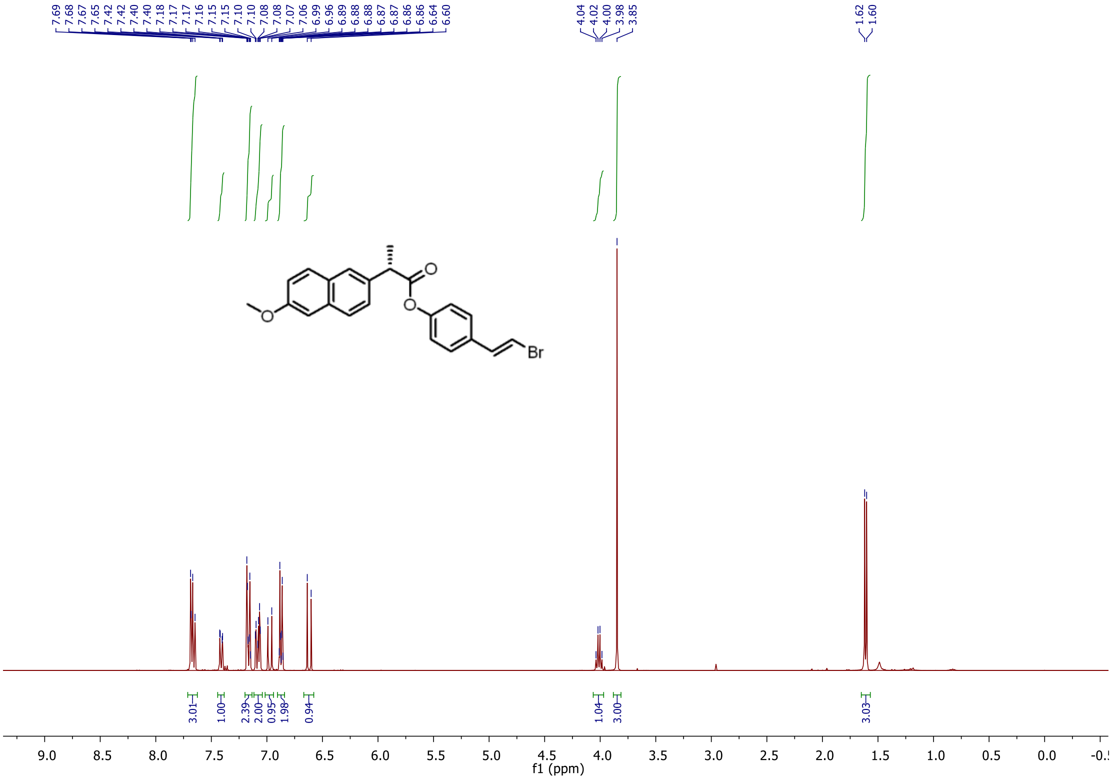

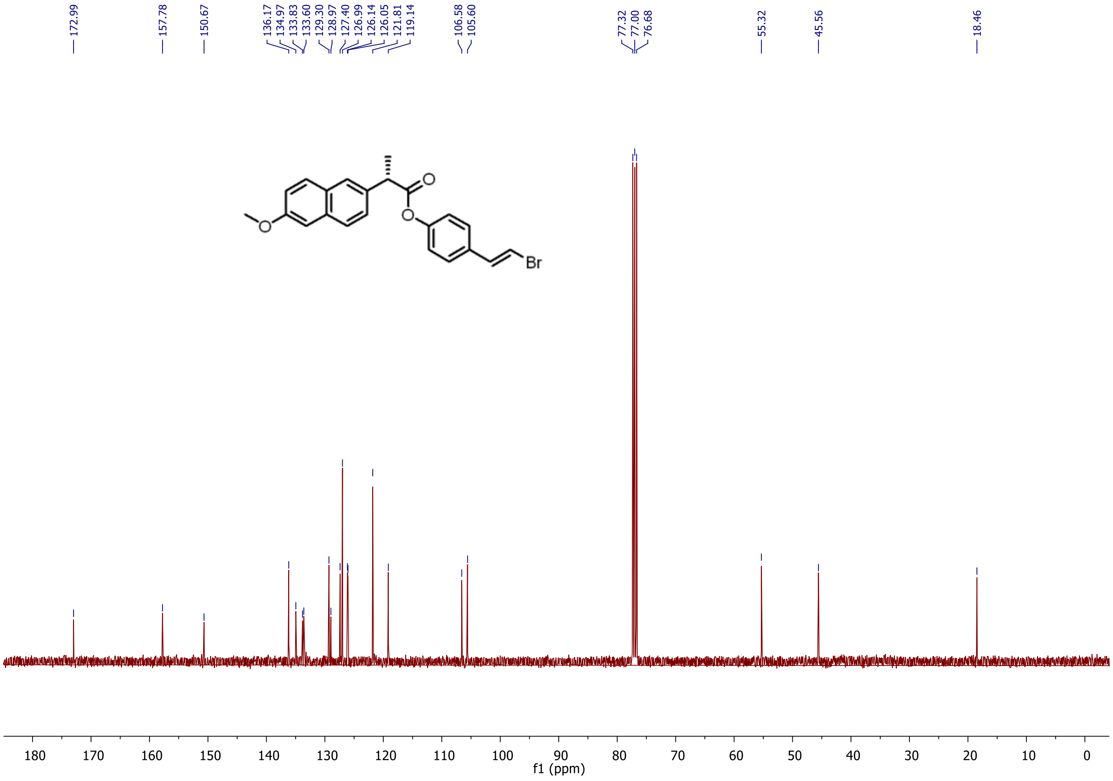

(*E*)-4-(2-Bromovinyl)phenyl 2-(4-(4-chlorobenzoyl)phenoxy)-2-methylpropanoate (S35)

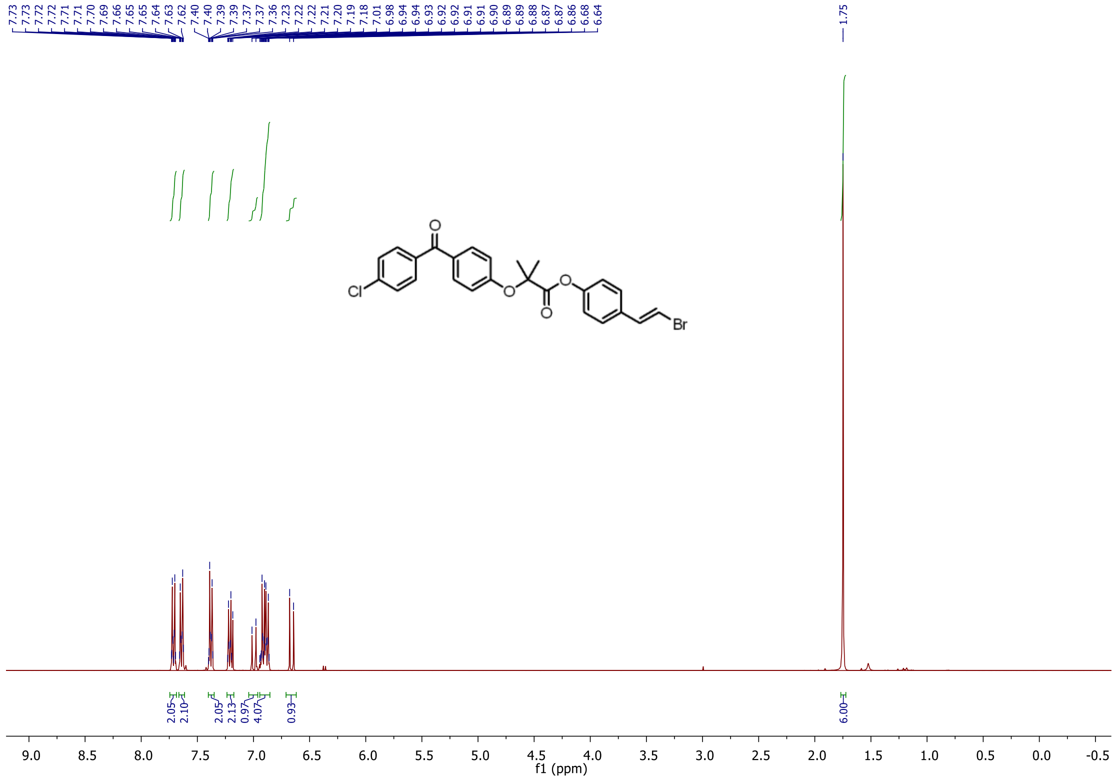

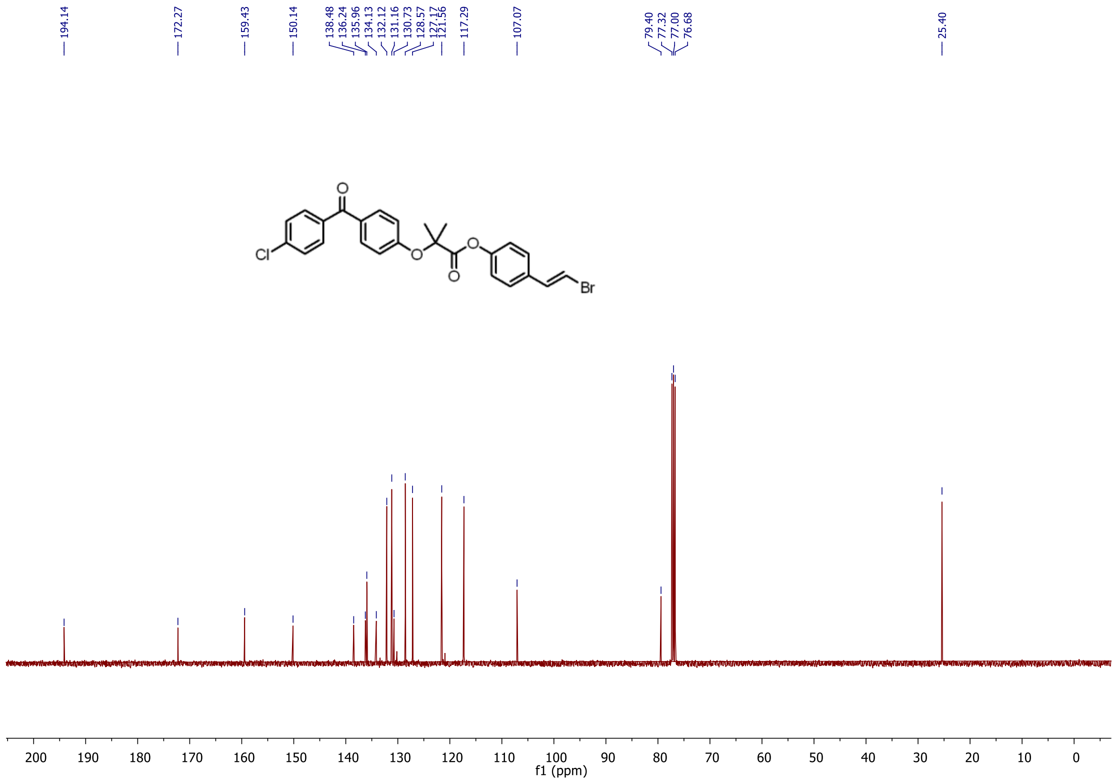

(*E*)-4-(2-Bromovinyl)phenyl 5-(2,5-dimethylphenoxy)-2,2-dimethylpentanoate (S36)

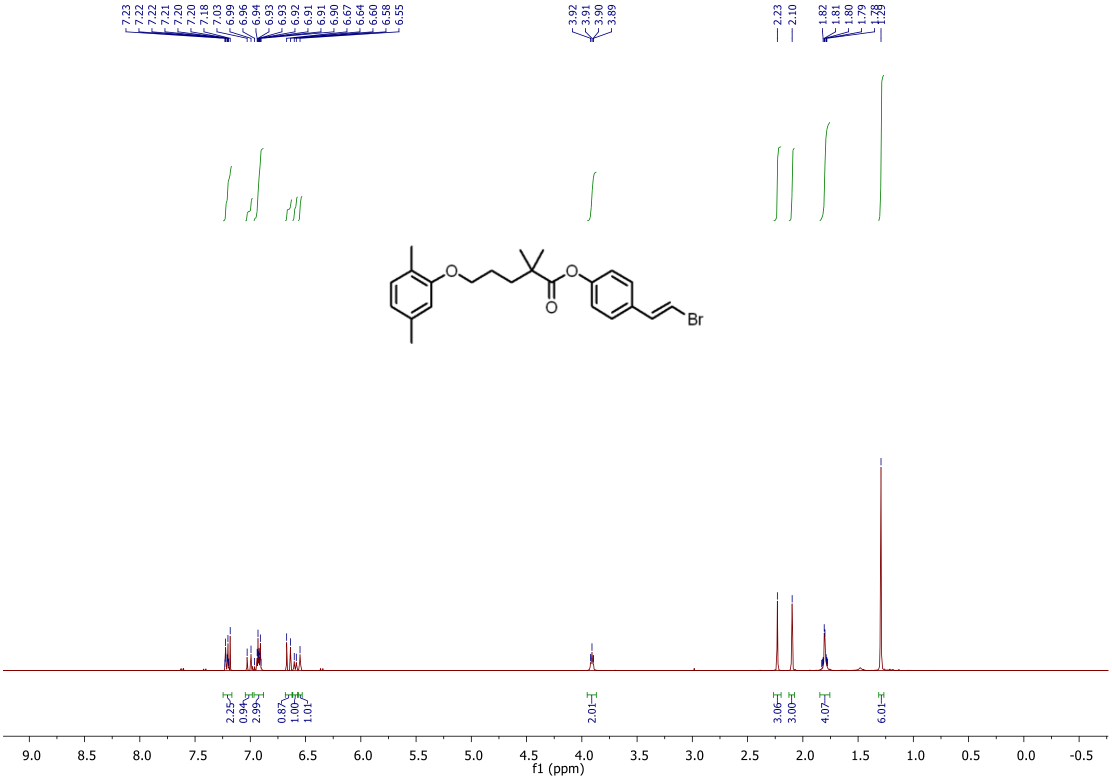

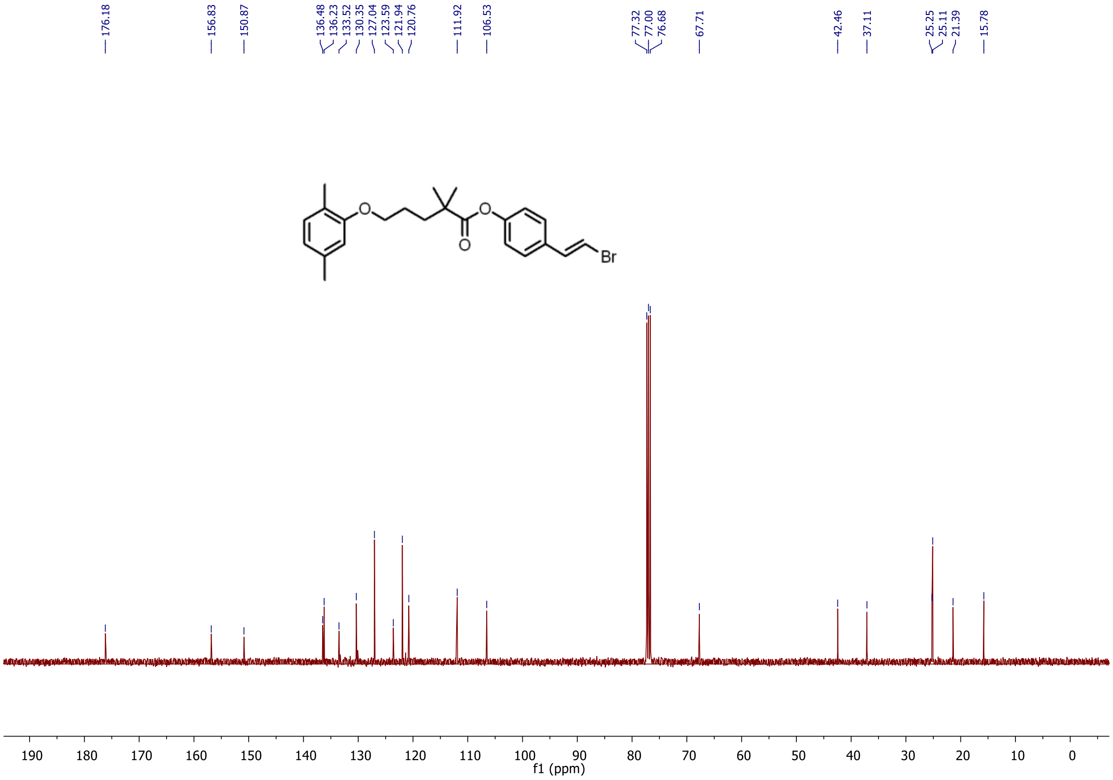

(*E*)-4-(2-Bromovinyl)phenyl (tert-butoxycarbonyl)-D-alaninate (S37)

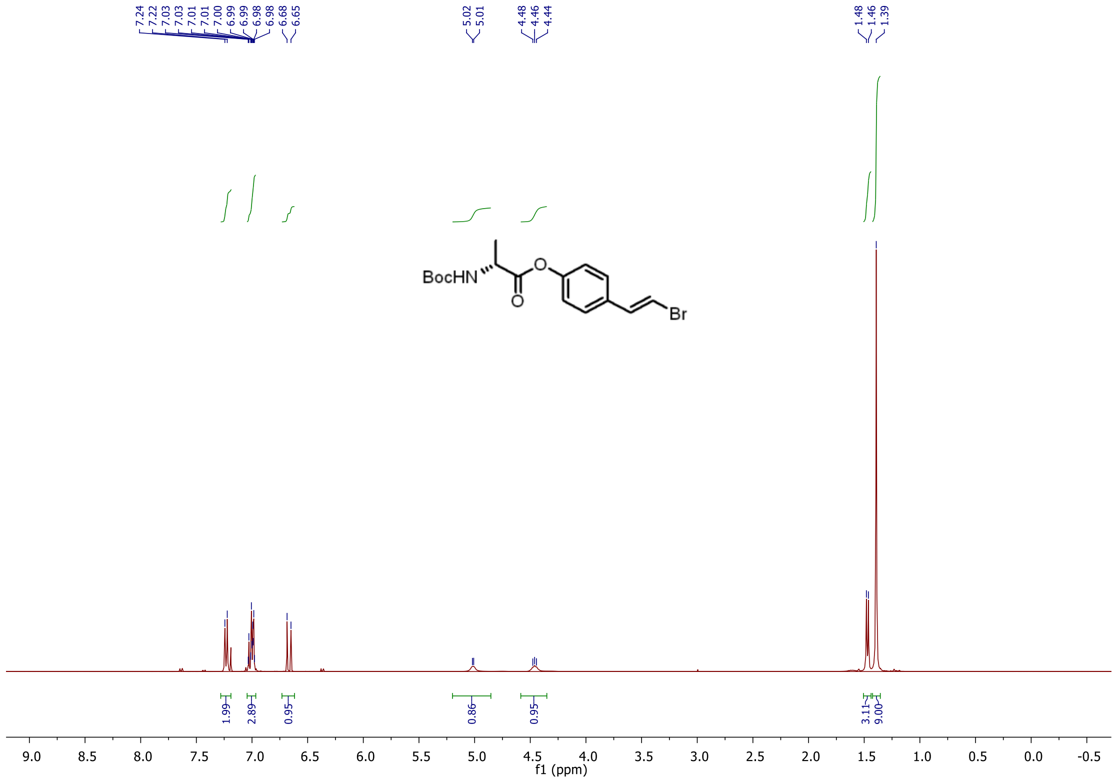

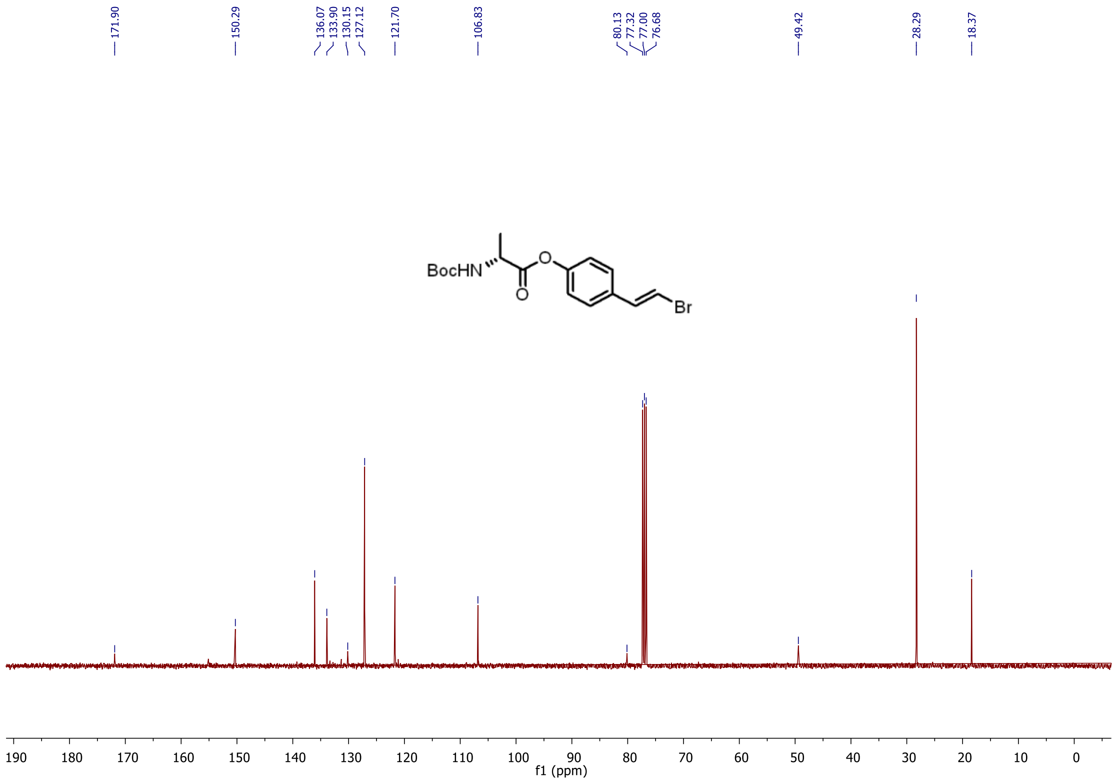

(*S*, *E*)-*N*-(2, 4-Diphenylbut-3-en-1-yl)-4-methylbenzenesulfonamide (1)

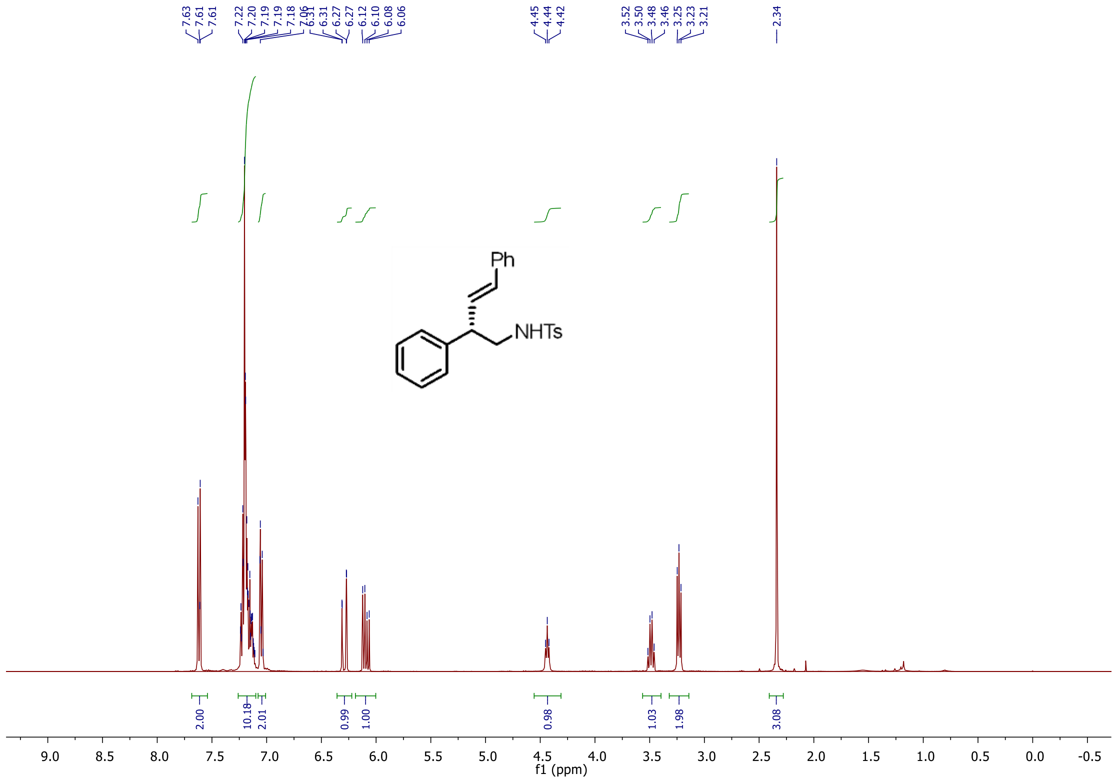

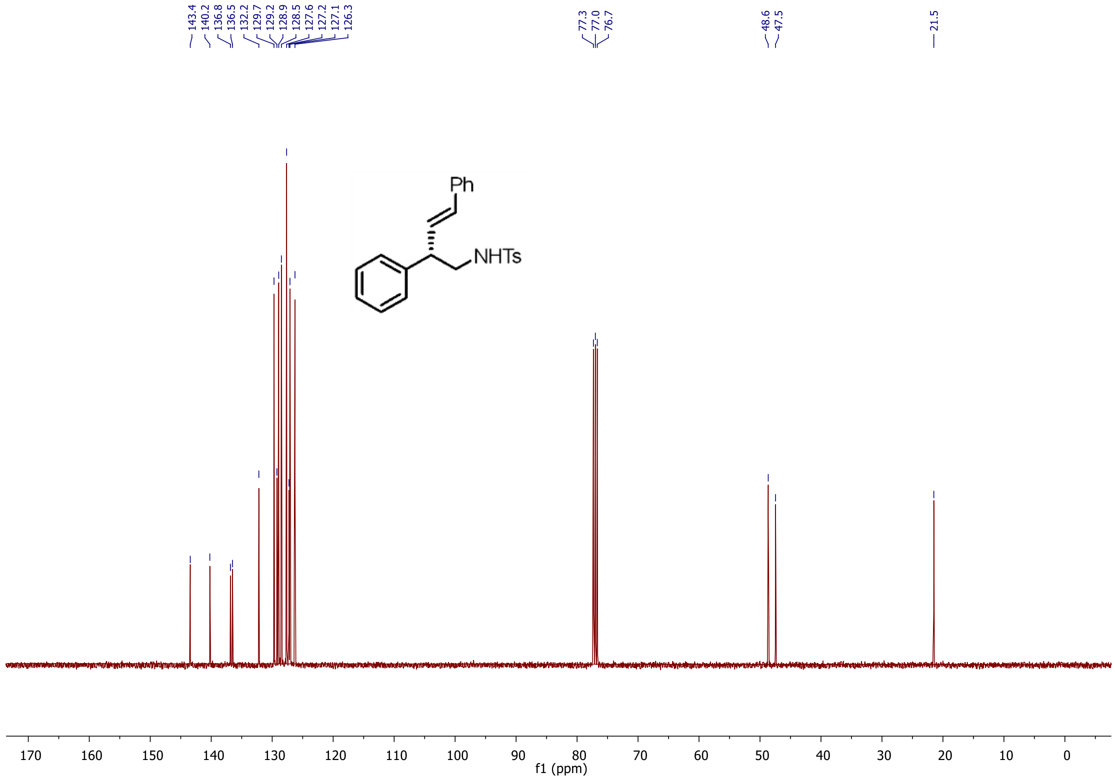

(*S, E*)-4-Methyl-*N*-(4-phenyl-2-(*p*-tolyl)but-3-en-1-yl)benzenesulfonamide (2)

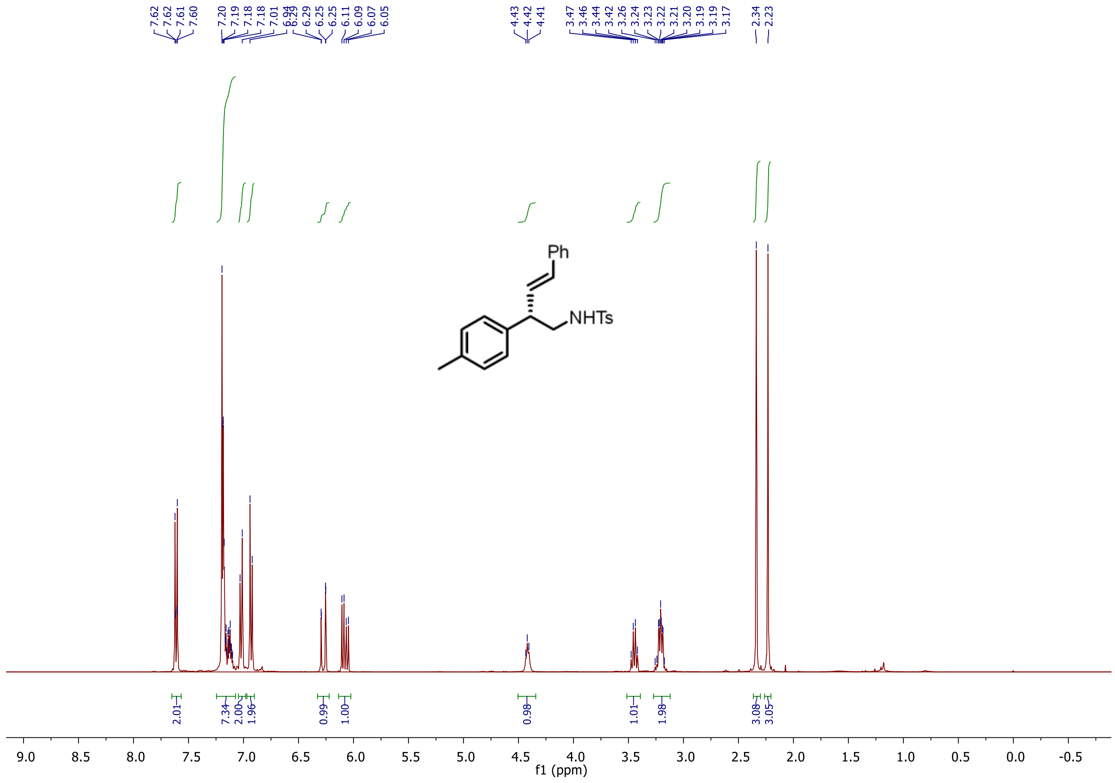

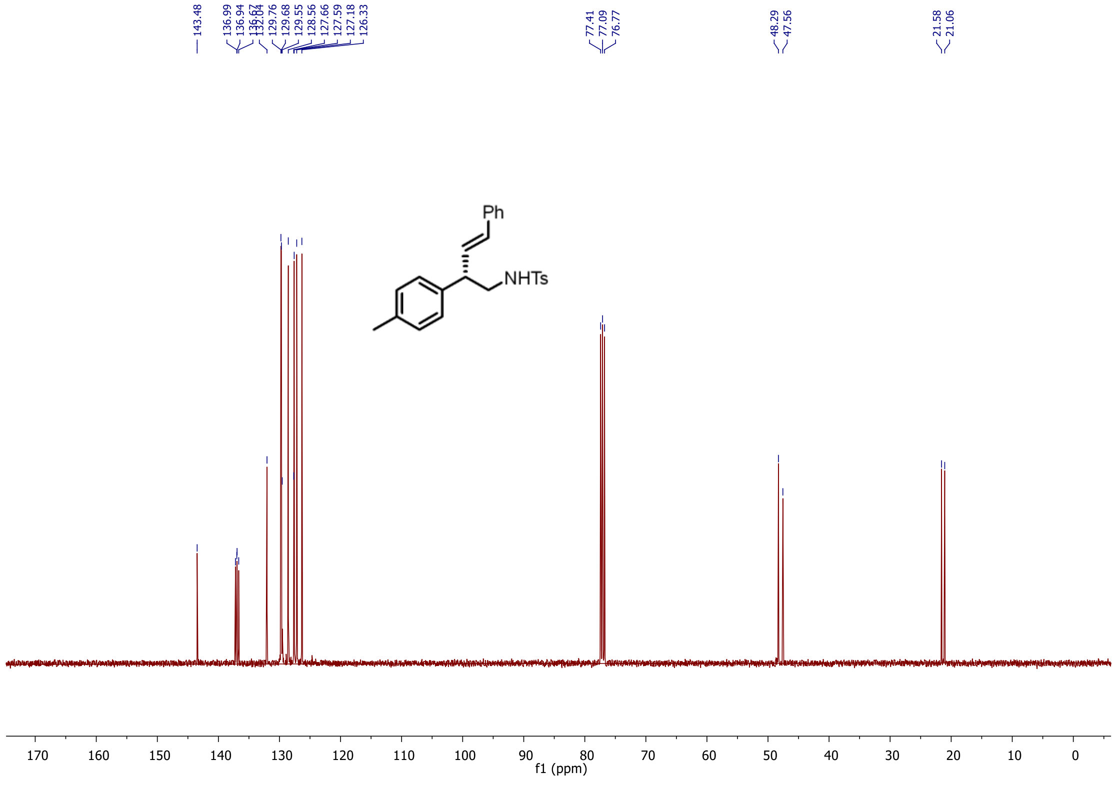

(*S*, 2*E*)-*N*-(2-(4-(*tert*-Butyl)phenyl)-4-phenylbut-3-en-1-yl)-4-methylbenzenesulfonamide (3)

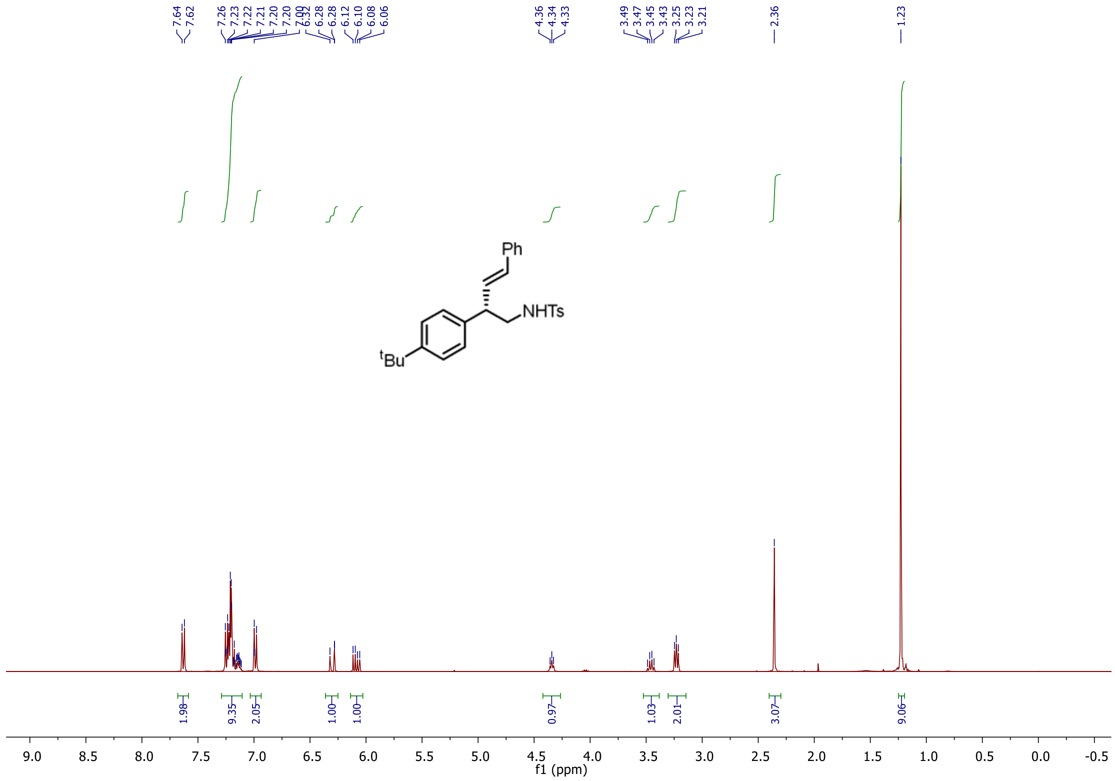

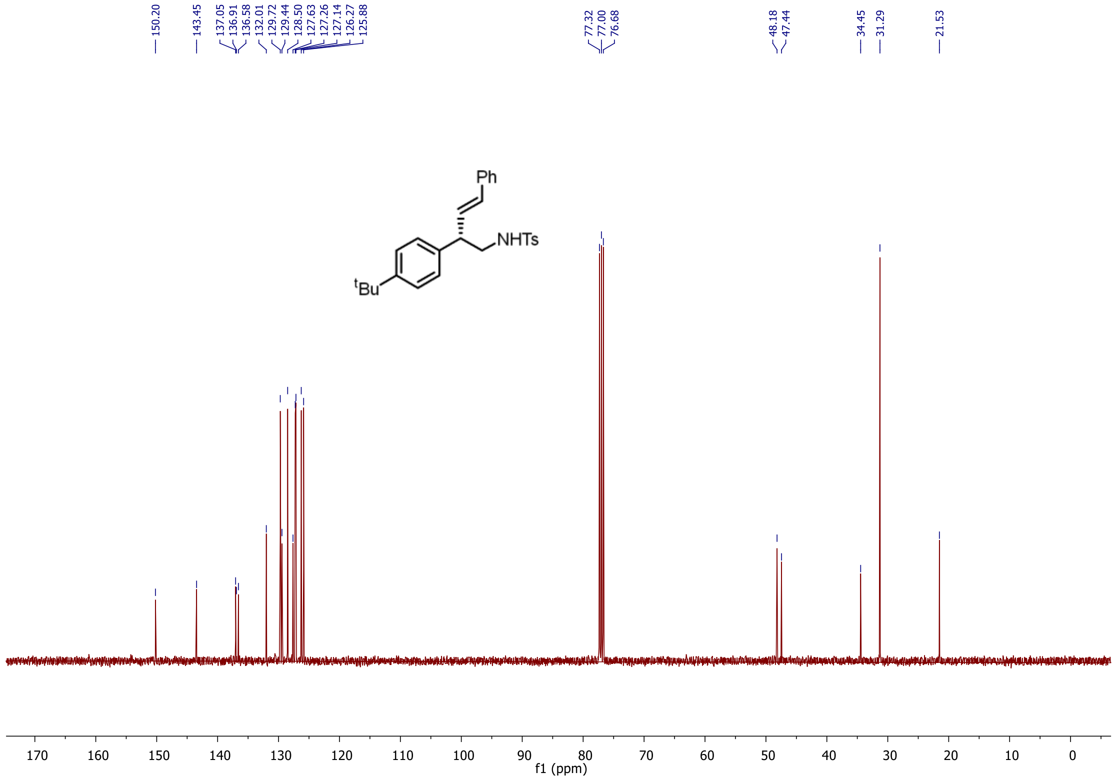

(*S*, *E*)-4-(1-((4-Methylphenyl)sulfonamido)-4-phenylbut-3-en-2-yl)phenyl acetate (4)

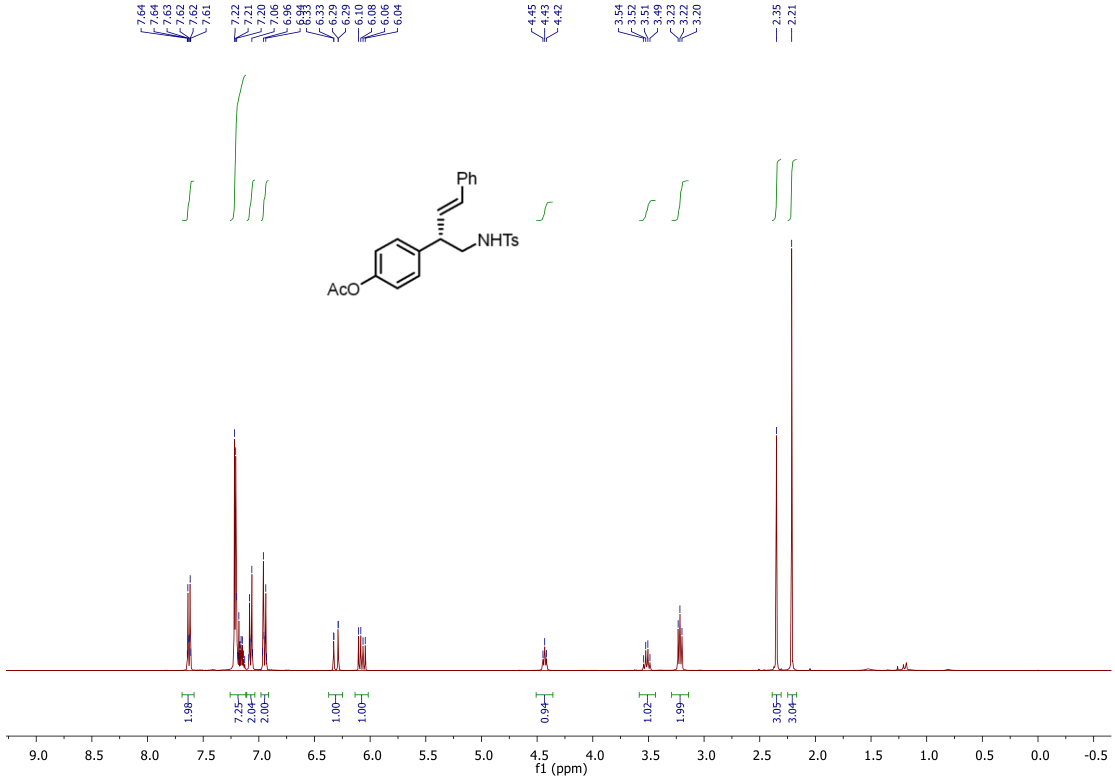

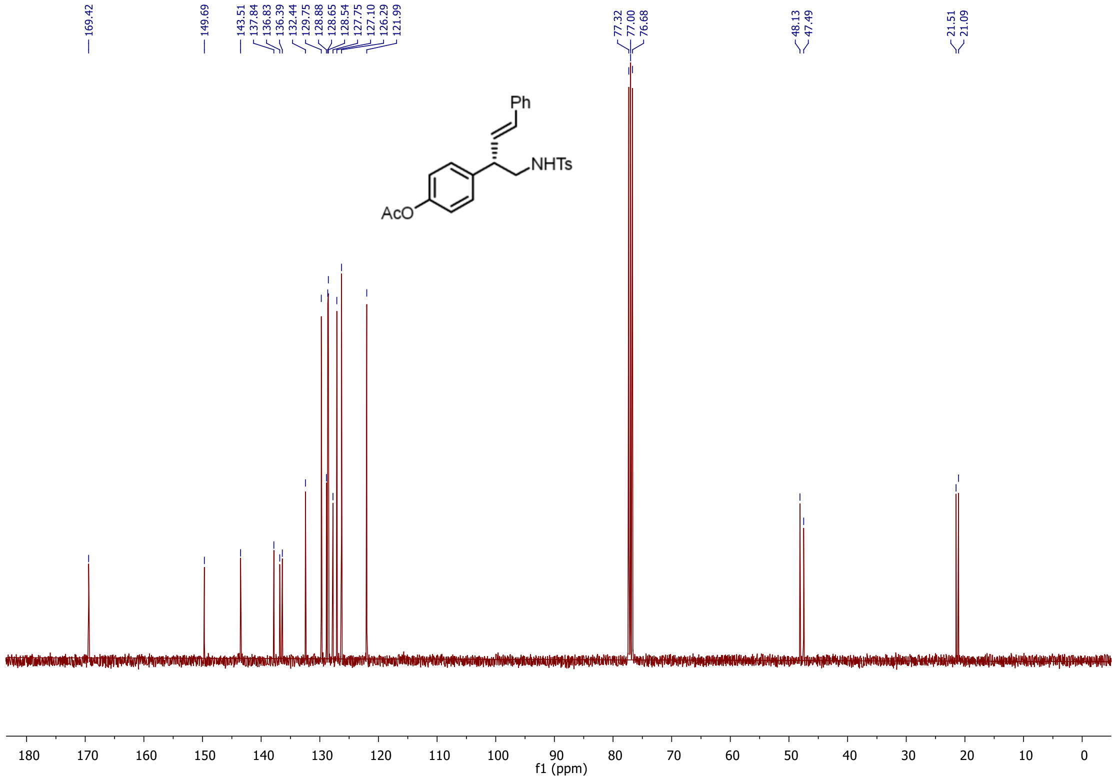

(*S*, *E*)-*N*-(2-(4-Fluorophenyl)-4-phenylbut-3-en-1-yl)-4-methylbenzenesulfonamide (5)

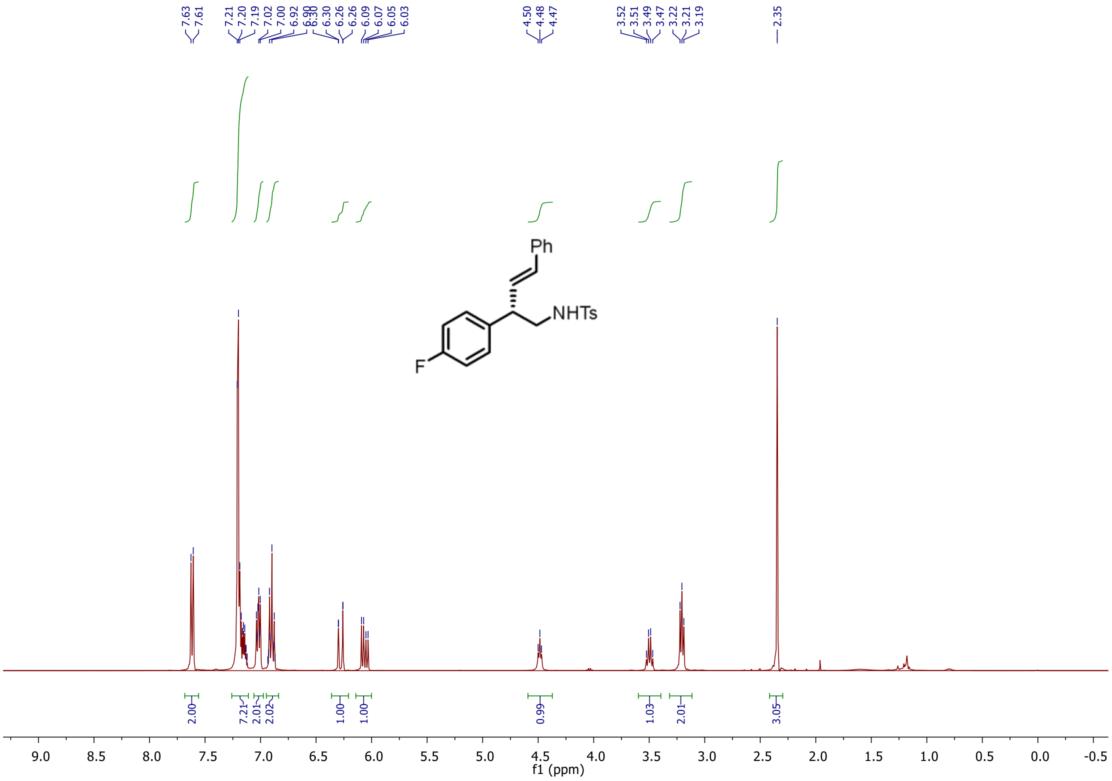

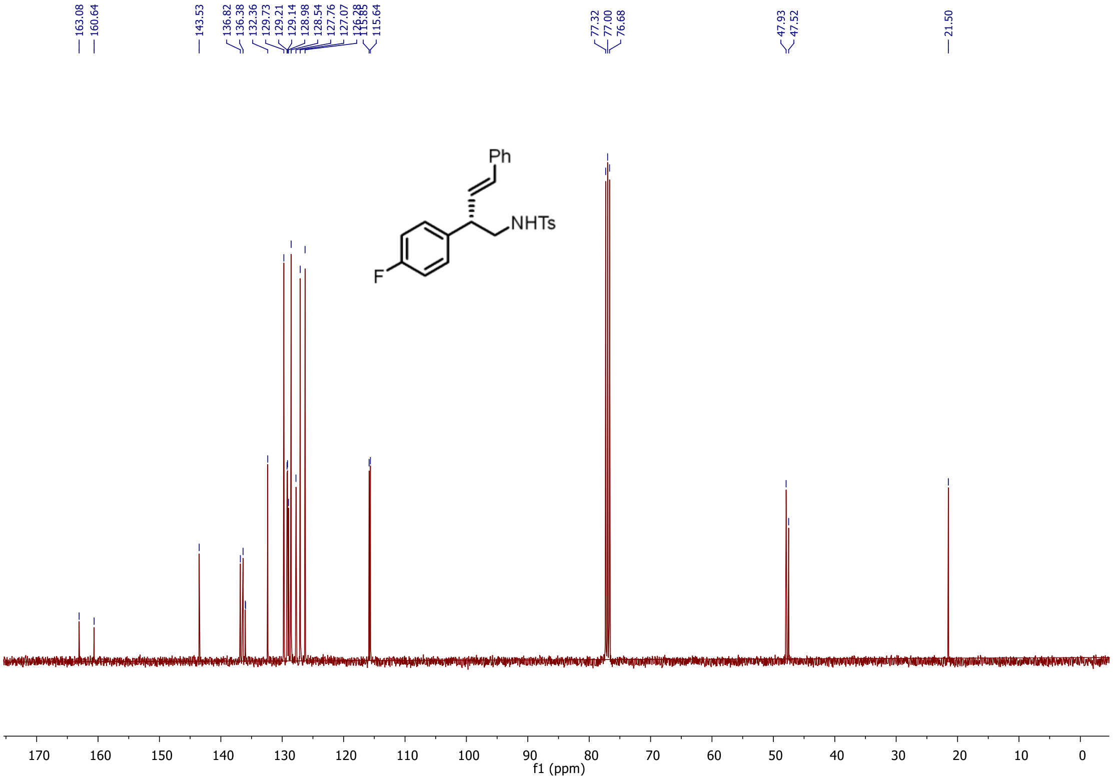

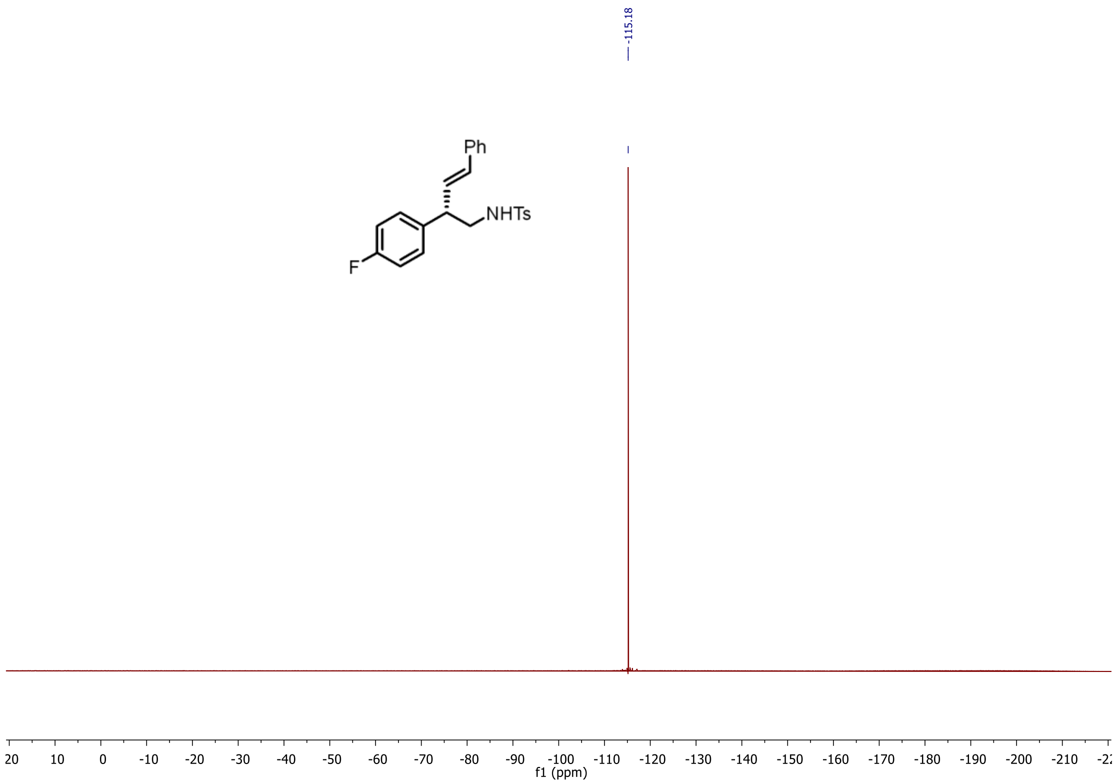

(*S*, *E*)-*N*-(2-(4-Chlorophenyl)-4-phenylbut-3-en-1-yl)-4-methylbenzenesulfonamide (6)

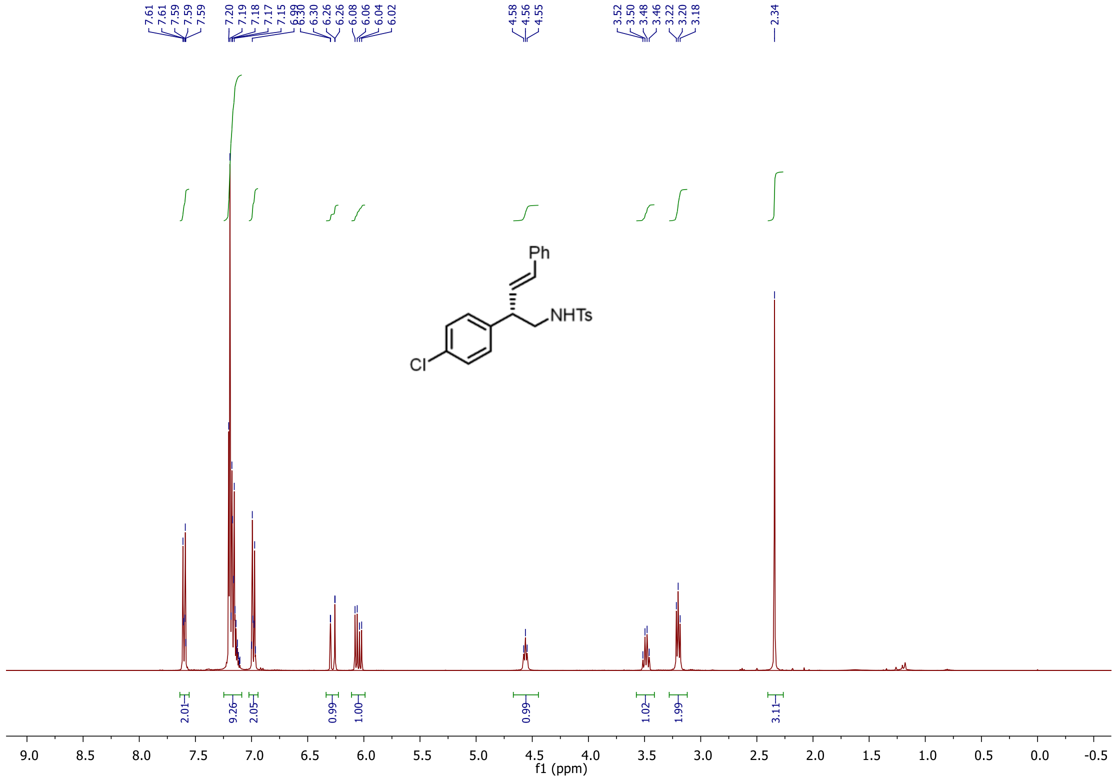

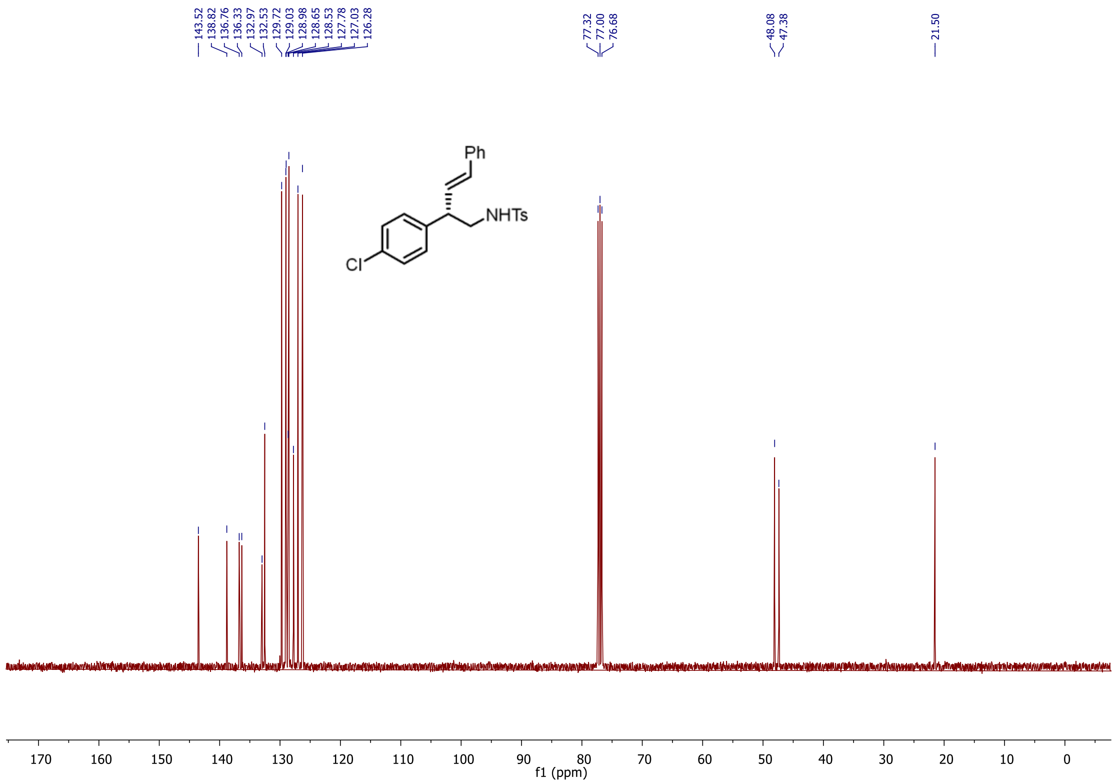

(*S*, *E*)-*N*-(2-(4-Bromophenyl)-4-phenylbut-3-en-1-yl)-4-methylbenzenesulfonamide (7)

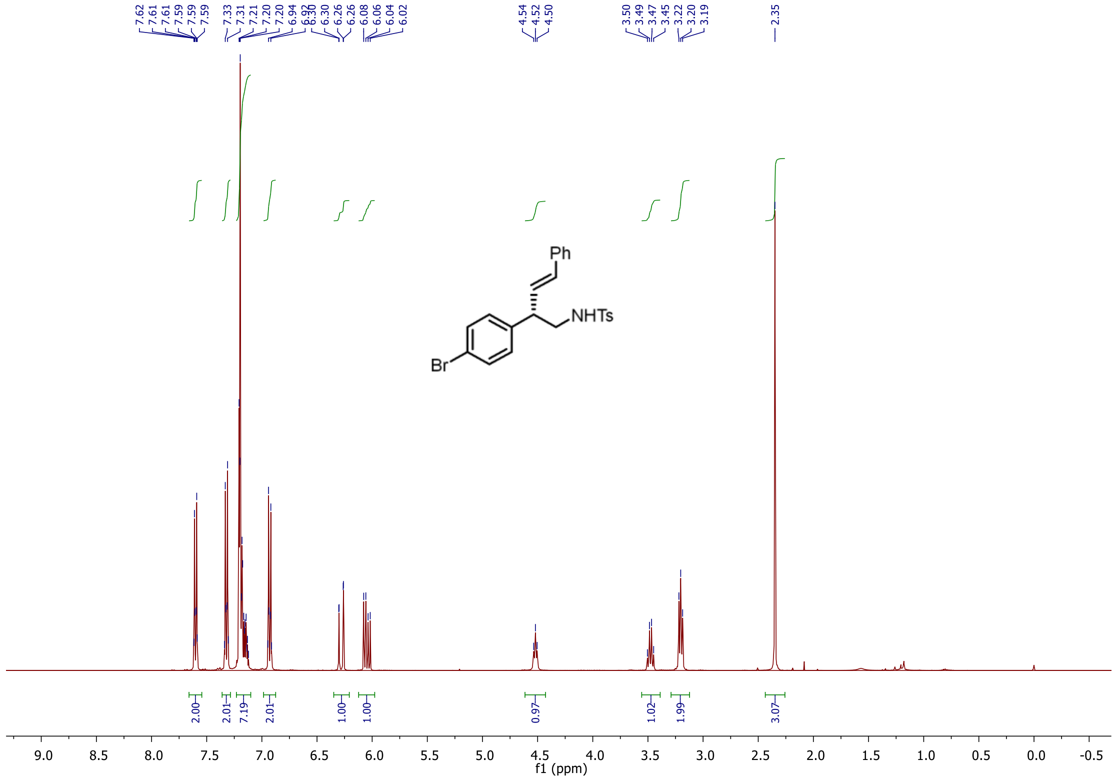

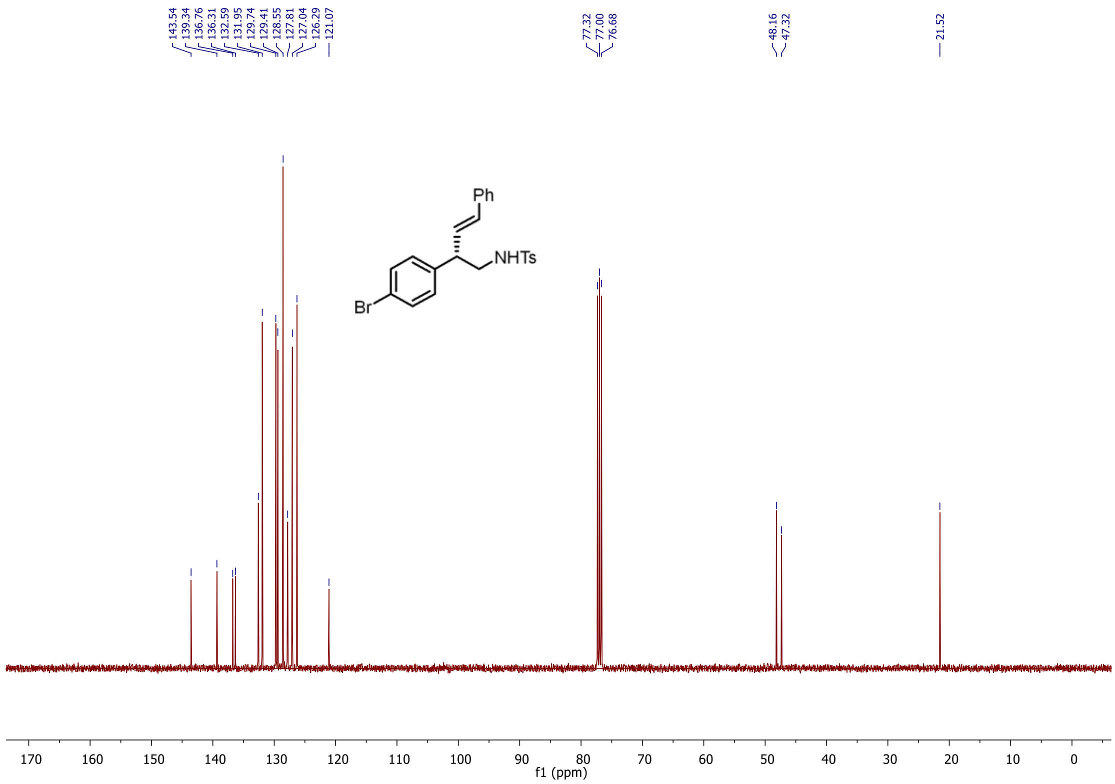

(*S*, *E*)-4-Methyl-*N*-(4-phenyl-2-(4-(trifluoromethyl)phenyl)but-3-en-1-yl)benzenesulfonamide (8)

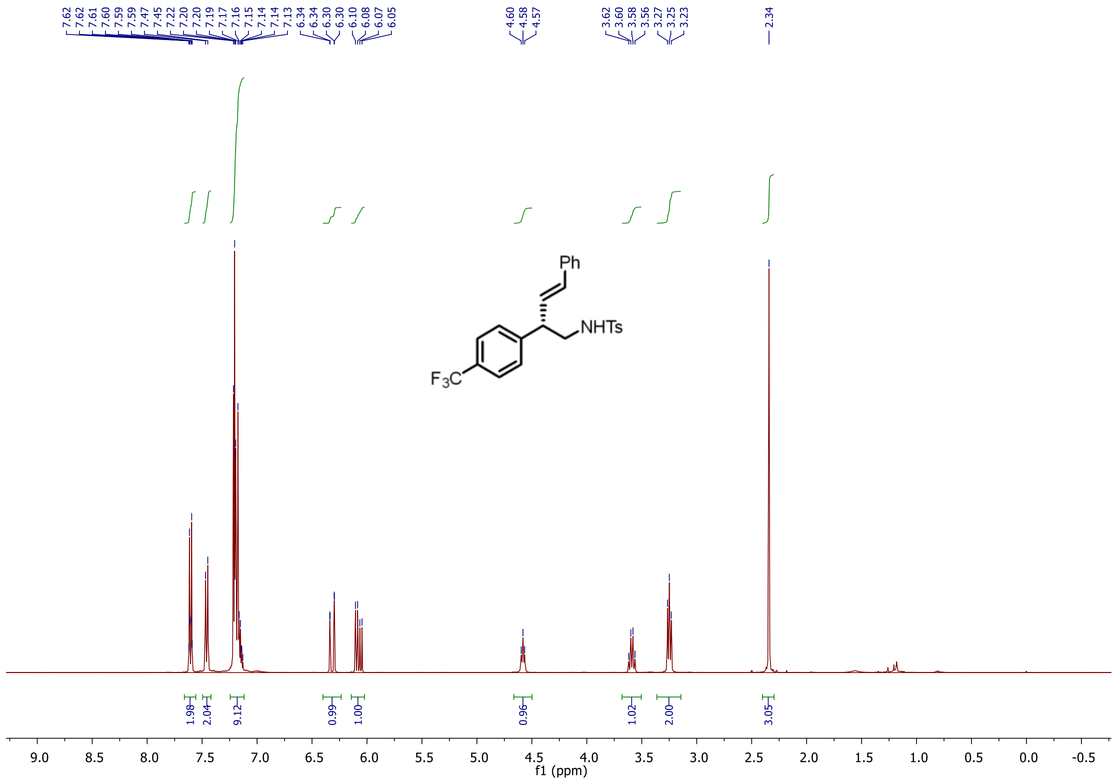

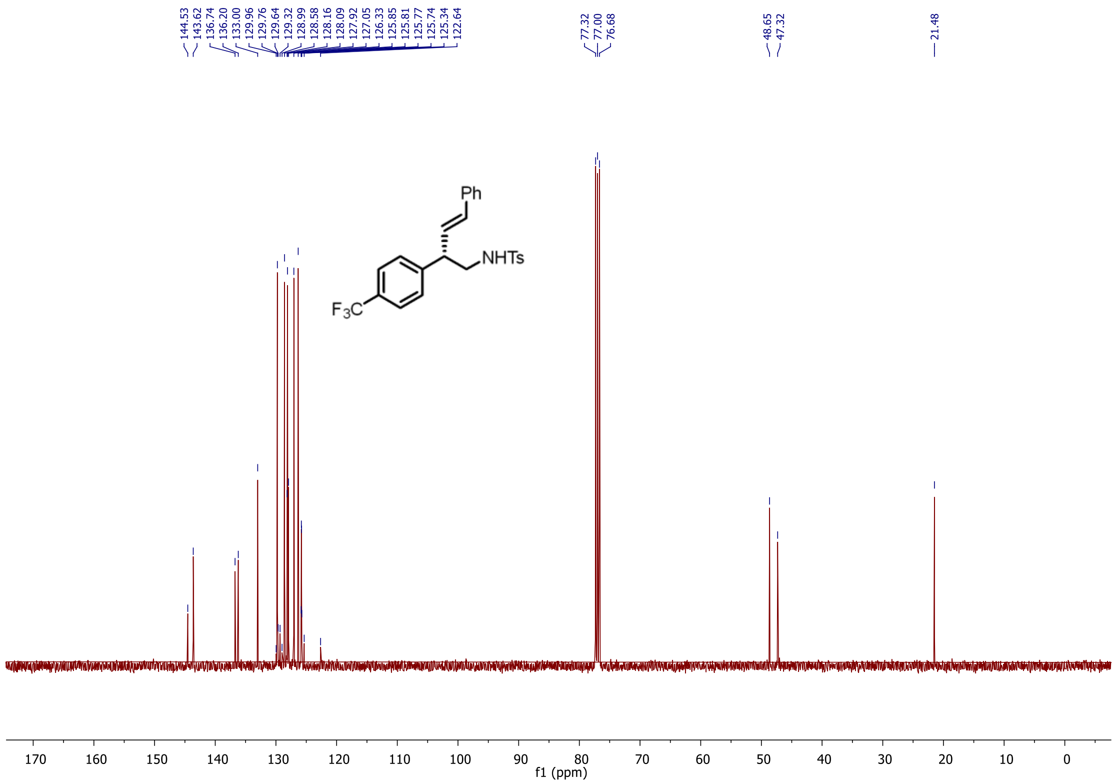
144.53
143.62
136.74
136.20
133.00
129.96
129.76
129.64
129.32
128.99
128.58
128.16
128.09
127.92
127.05
126.33
125.85
125.81
125.77
125.74
125.34
122.64
77.32
77.00
76.68
48.65
47.32
21.48
Ph
NHTs
F3C
f1 (ppm)

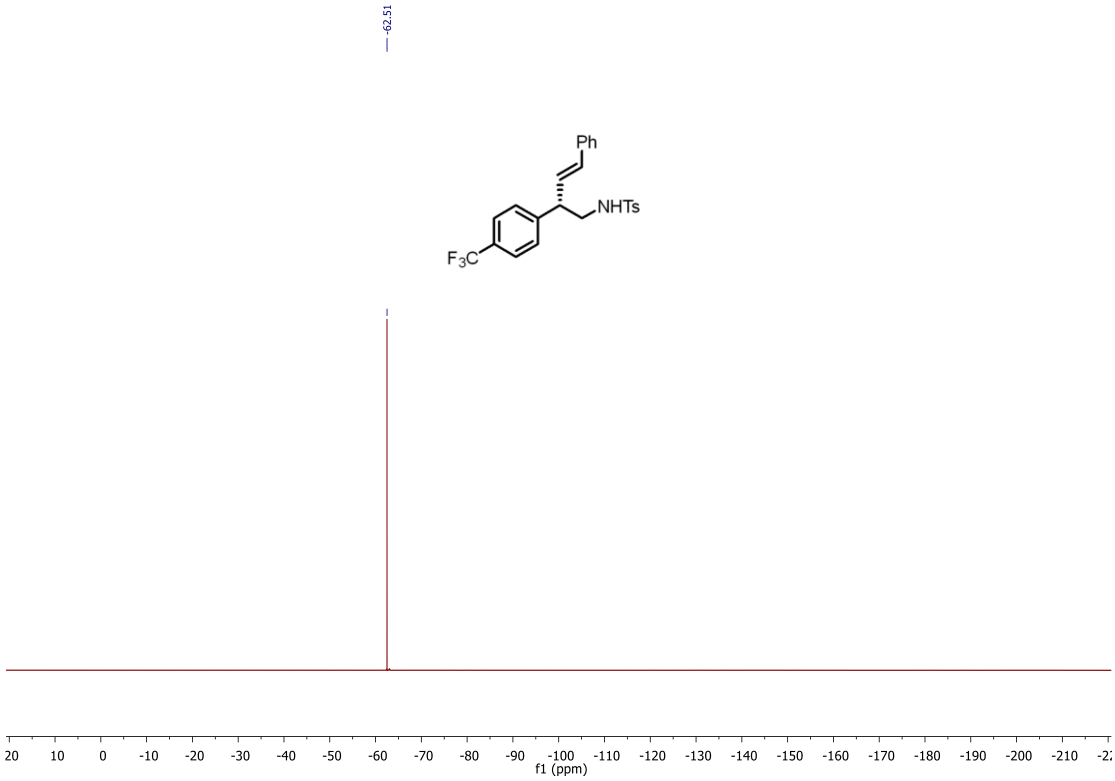
-62.51
Ph
NHTs
F3C
f1 (ppm)

Methyl (*S*, *E*)-4-(1-((4-methylphenyl)sulfonamido)-4-phenylbut-3-en-2-yl)benzoate (9)

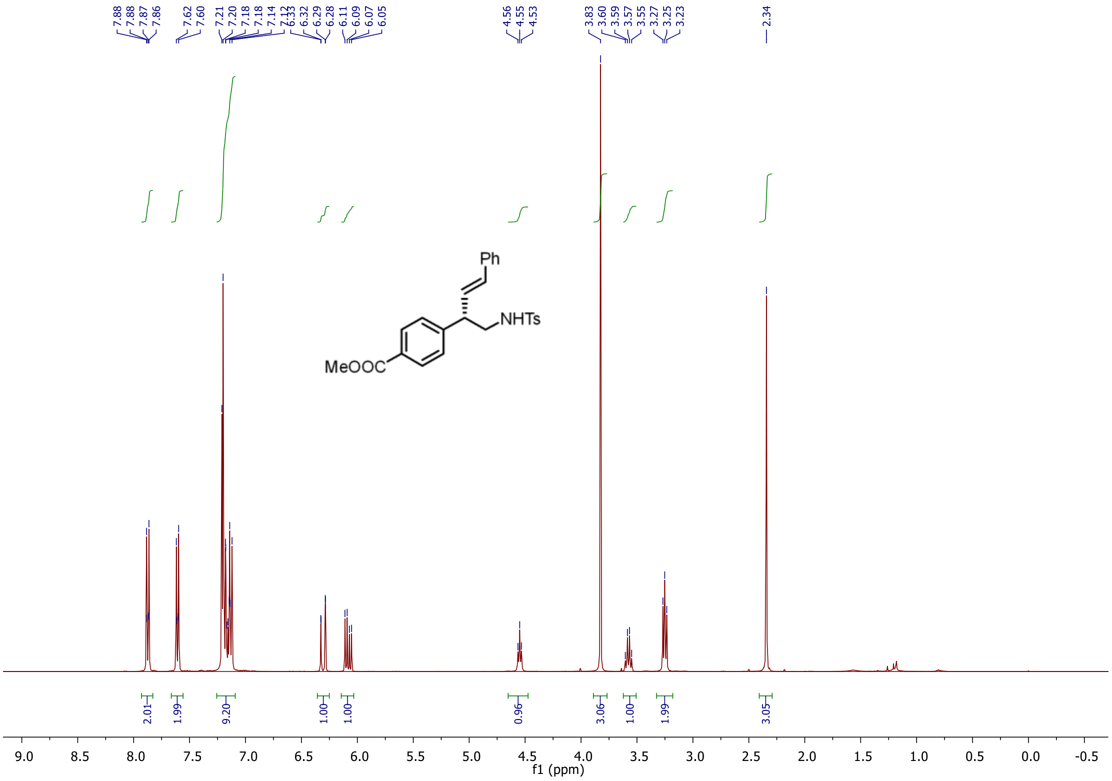

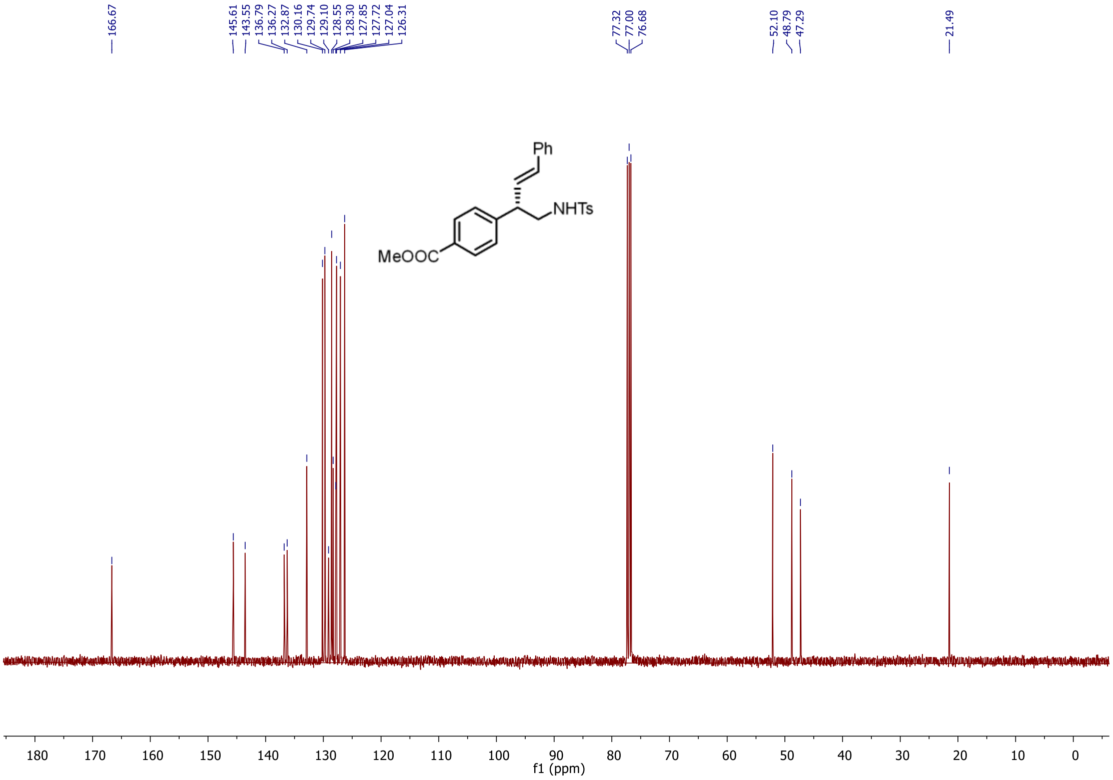

(*S*, *E*)-*N*-(2-(4-Cyanophenyl)-4-phenylbut-3-en-1-yl)-4-methylbenzenesulfonamide (10)

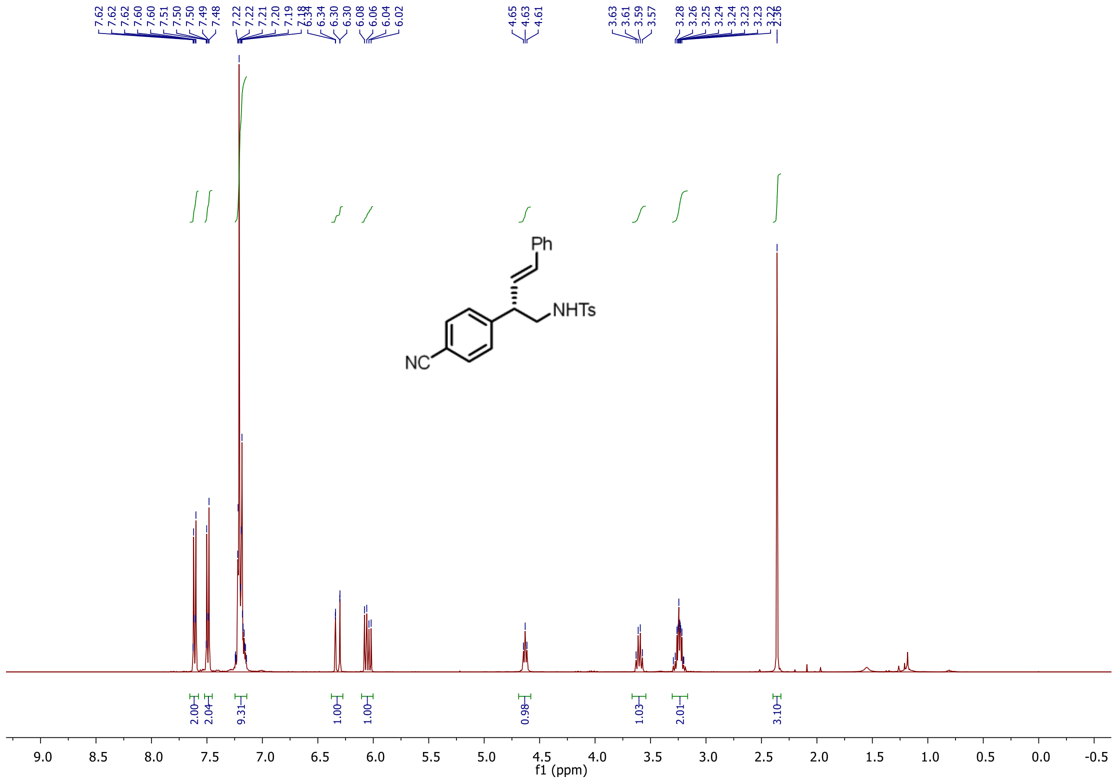

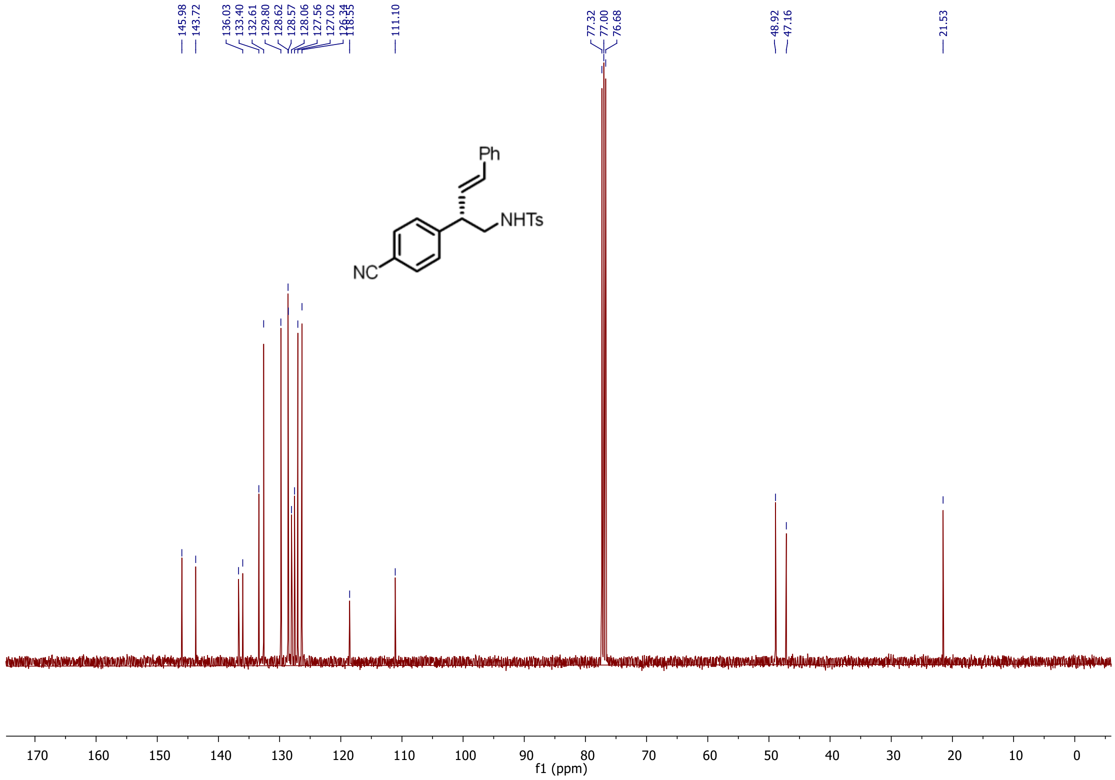

(*S, E*)-4-Methyl-*N*-(4-phenyl-2-(*o*-tolyl)but-3-en-1-yl)benzenesulfonamide (11)

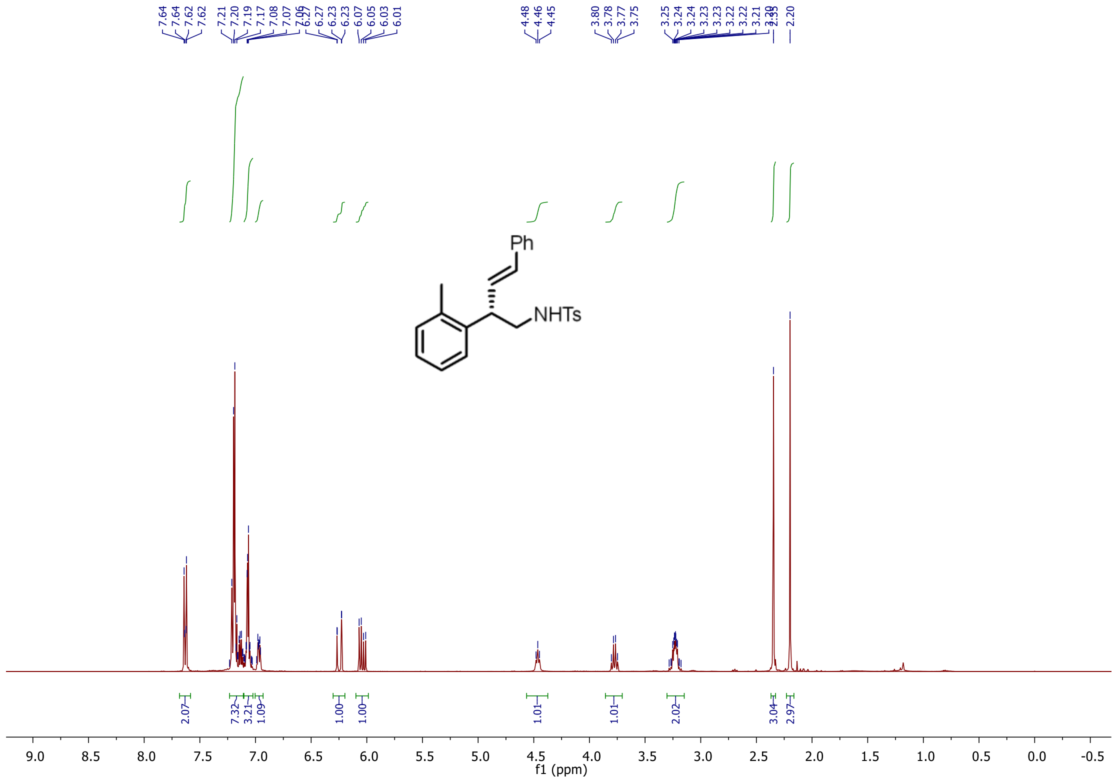

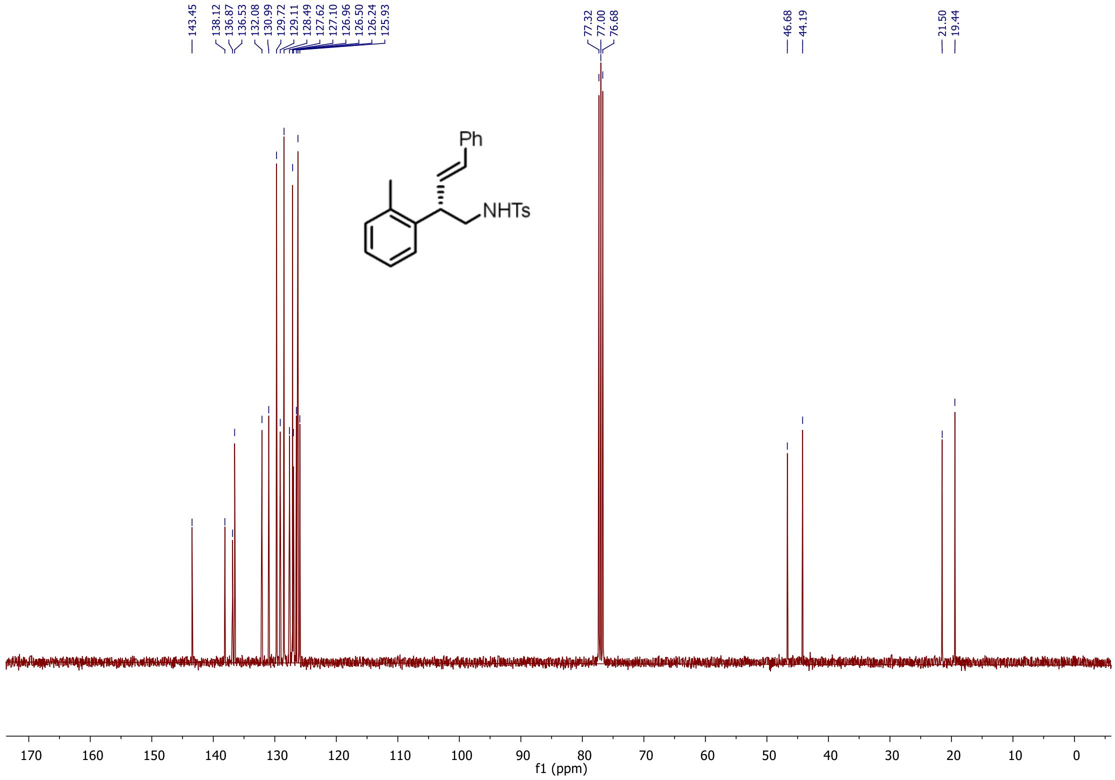

(*S, E*)-4-Methyl-*N*-(4-phenyl-2-(*m*-tolyl)but-3-en-1-yl)benzenesulfonamide (12)

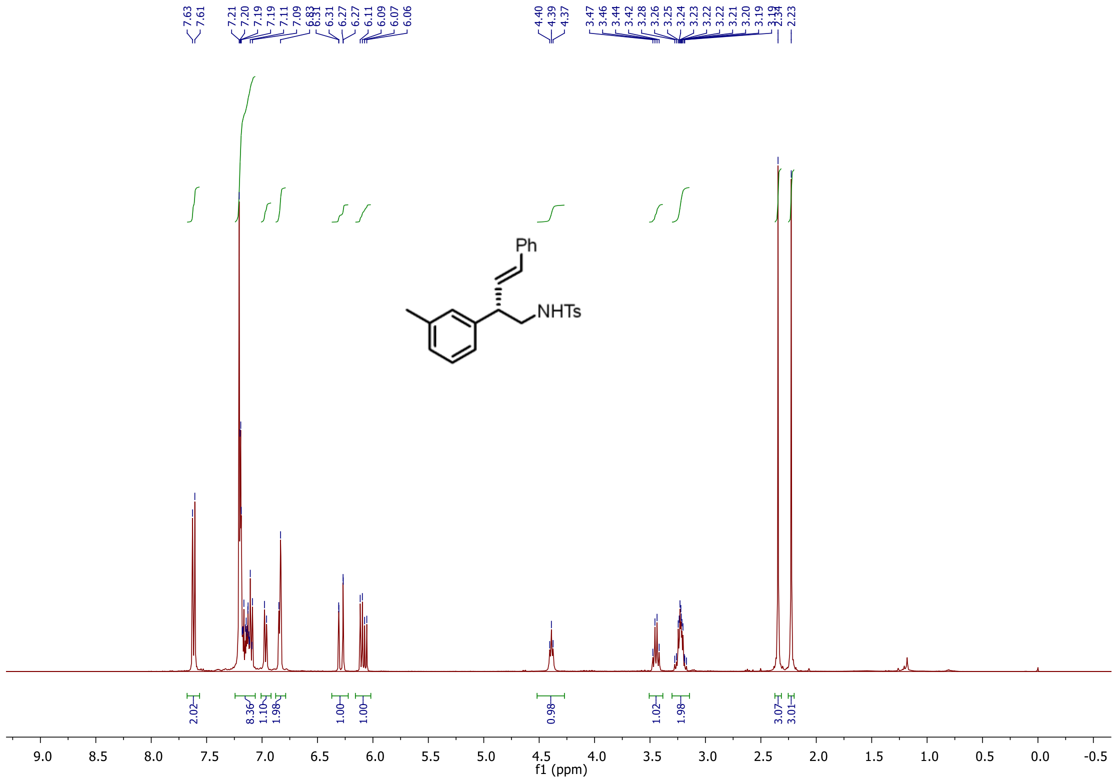

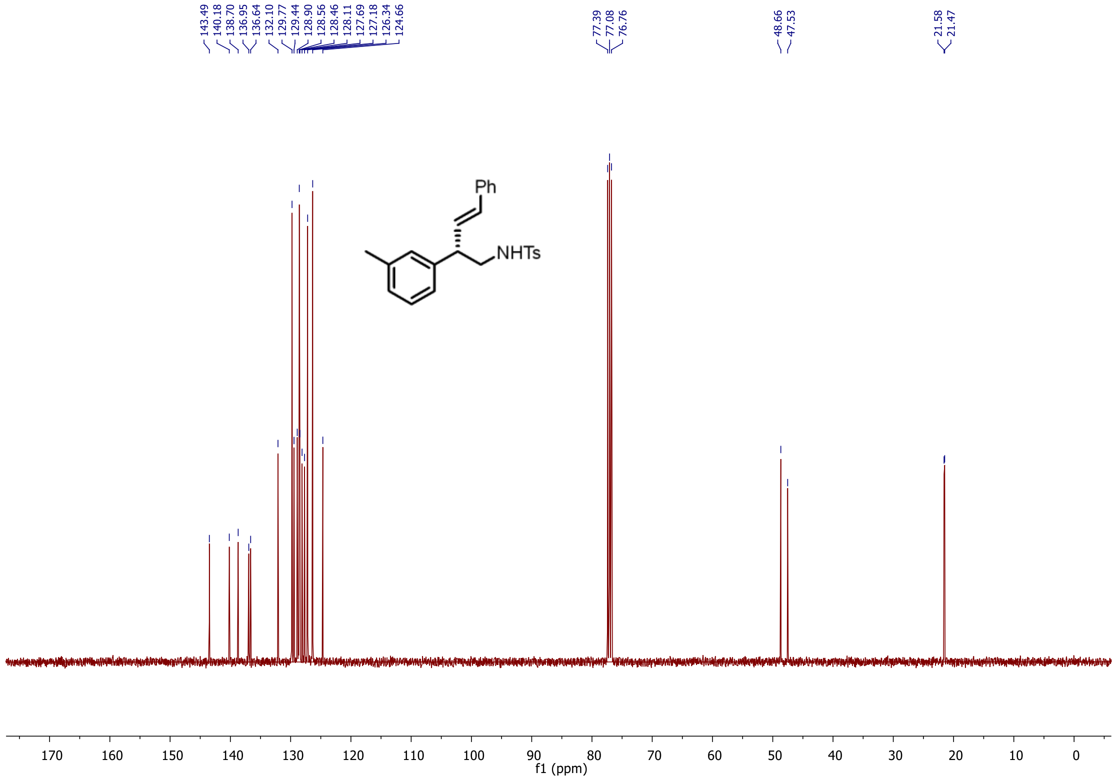

(*S*, *E*)-4-Methyl-*N*-(2-(naphthalen-2-yl)-4-phenylbut-3-en-1-yl)benzenesulfonamide (13)

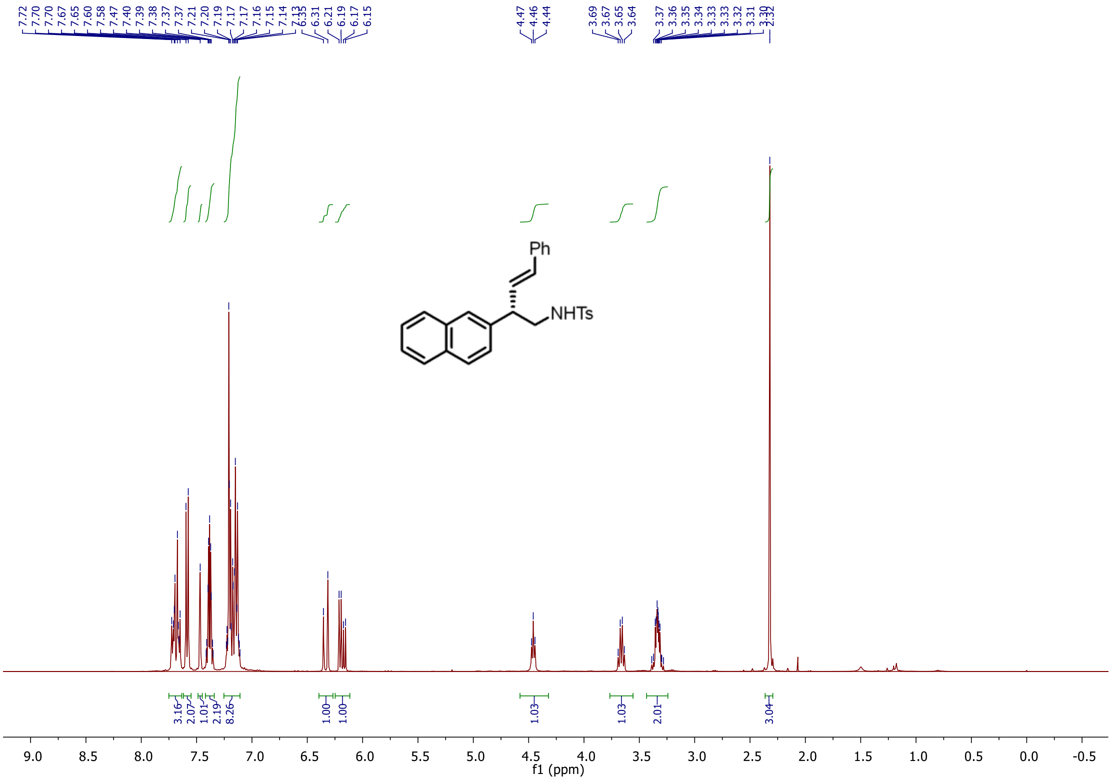

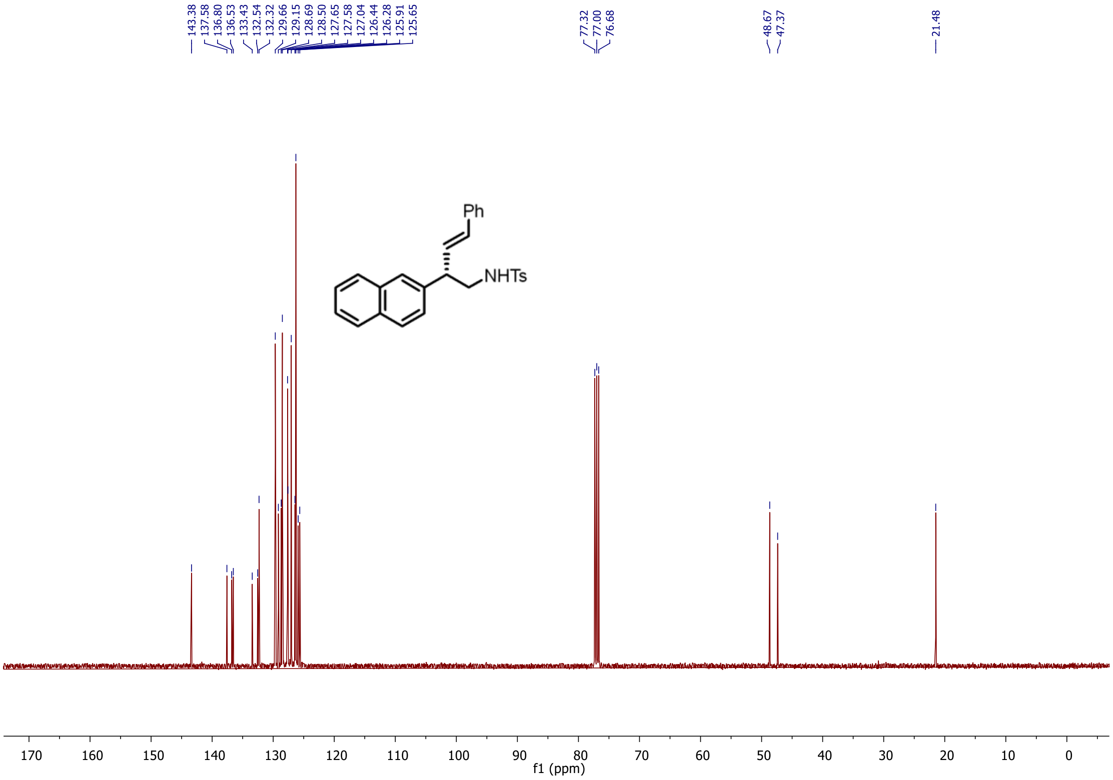

***tert*-Butyl (*S*, *E*)-5-(1-((4-methylphenyl)sulfonamido)-4-phenylbut-3-en-2-yl)-1H-indole-1-carboxylate (14)**

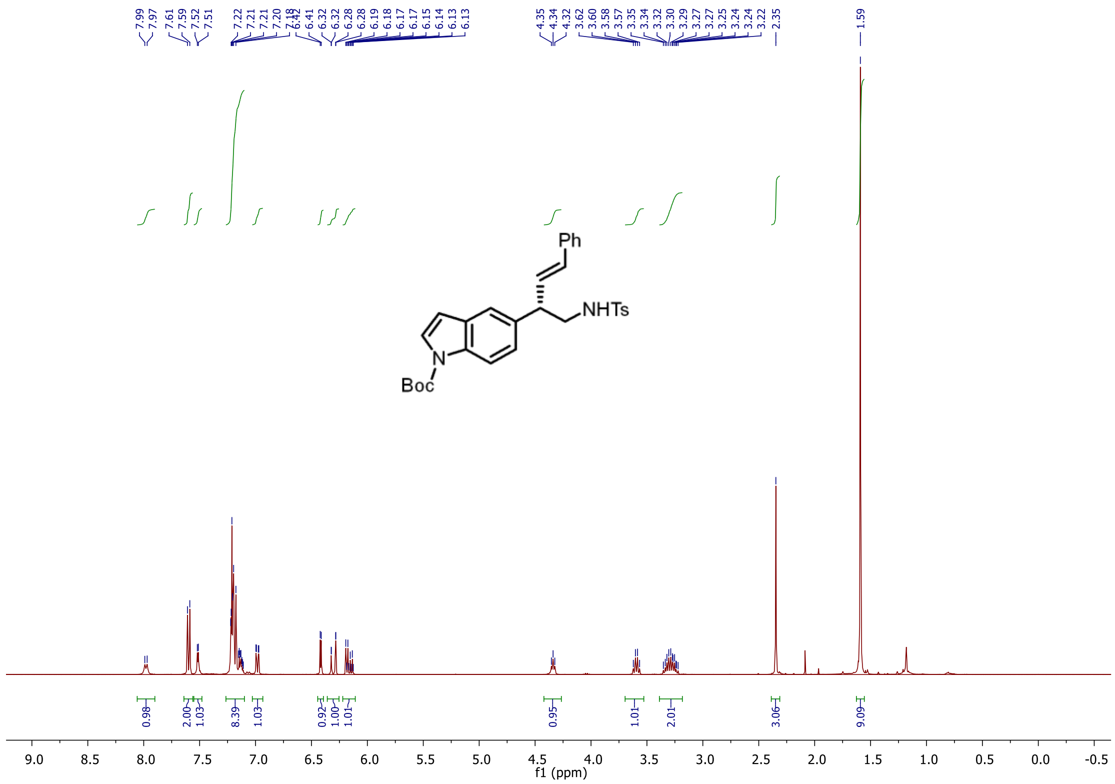

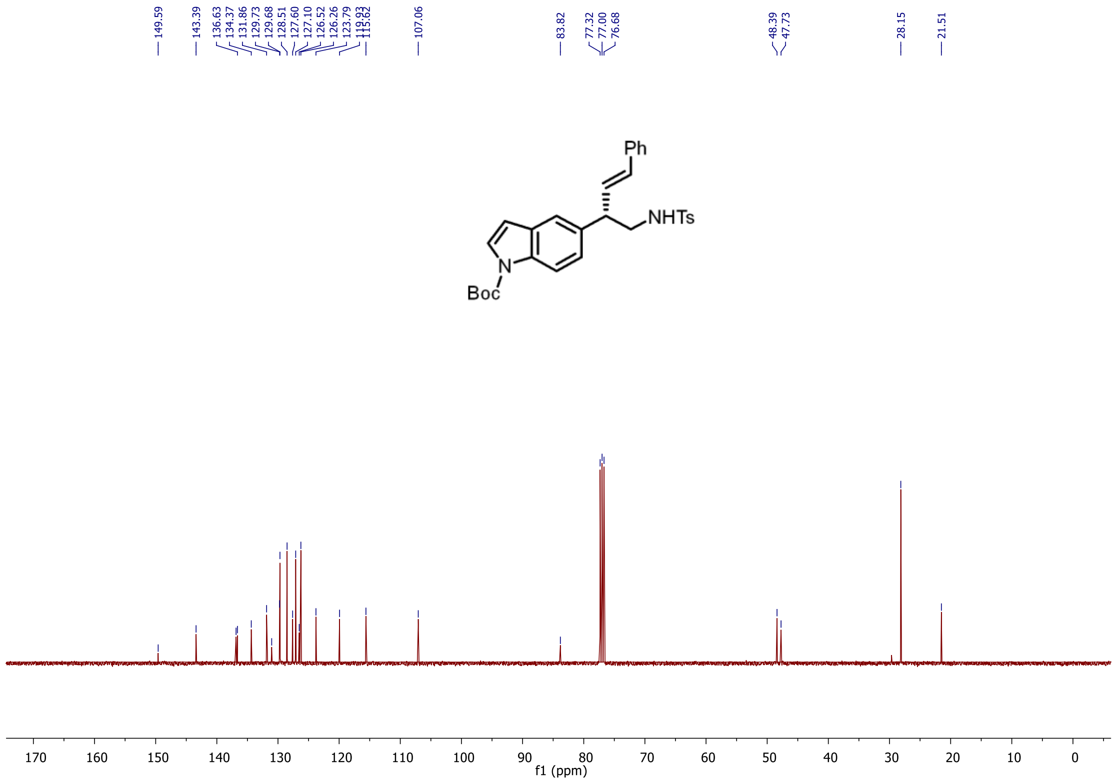

(*S*, *E*)-4-Methyl-N-(2-phenyl-4-(p-tolyl)but-3-en-1-yl)benzenesulfonamide (15)

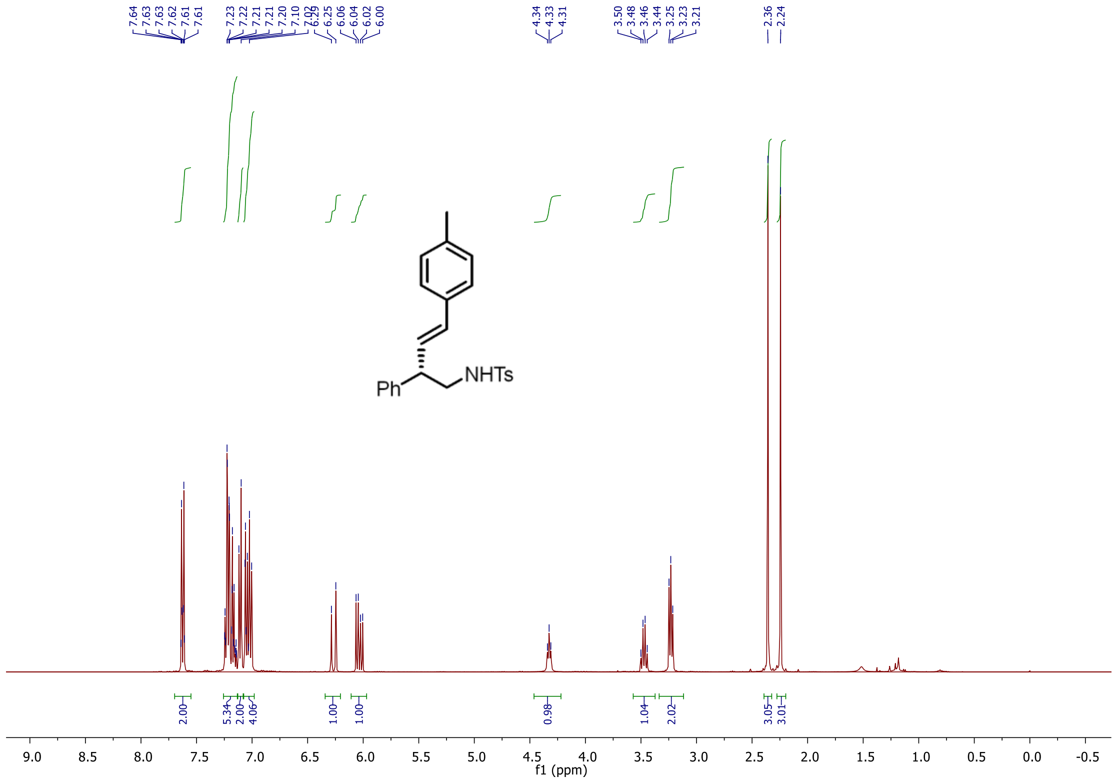

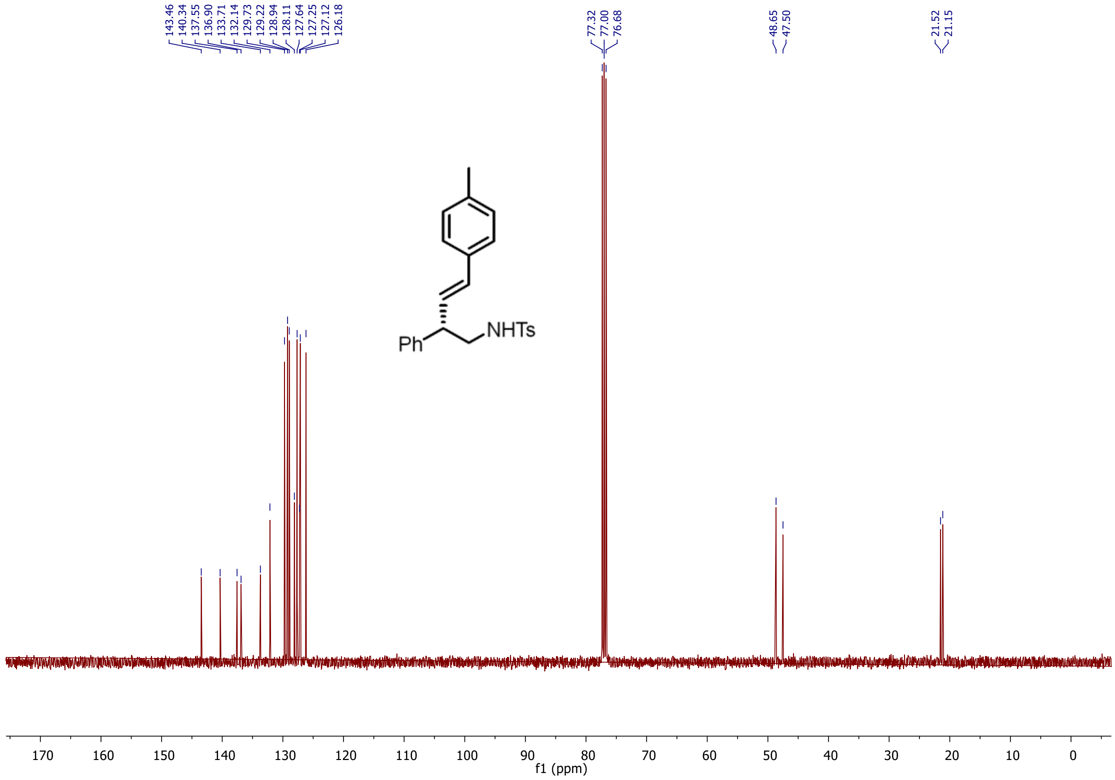

(*S*, *E*)-*N*-(4-(4-Methoxyphenyl)-2-phenylbut-3-en-1-yl)-4-methylbenzenesulfonamide (16)

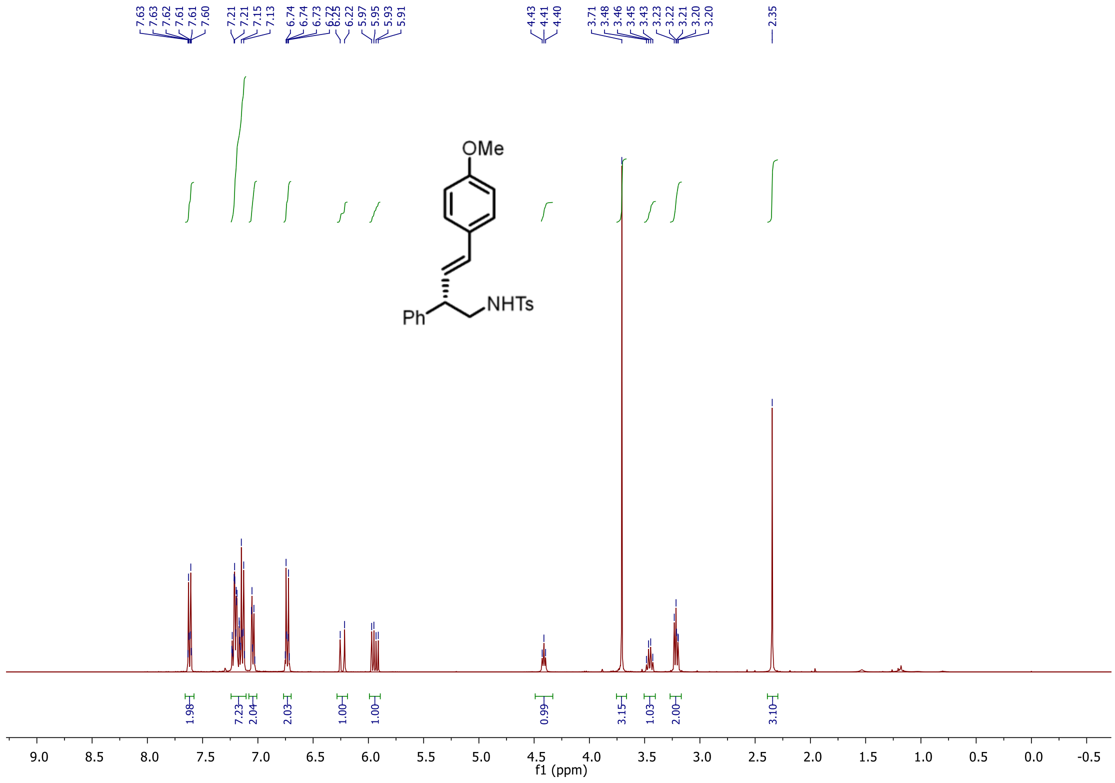

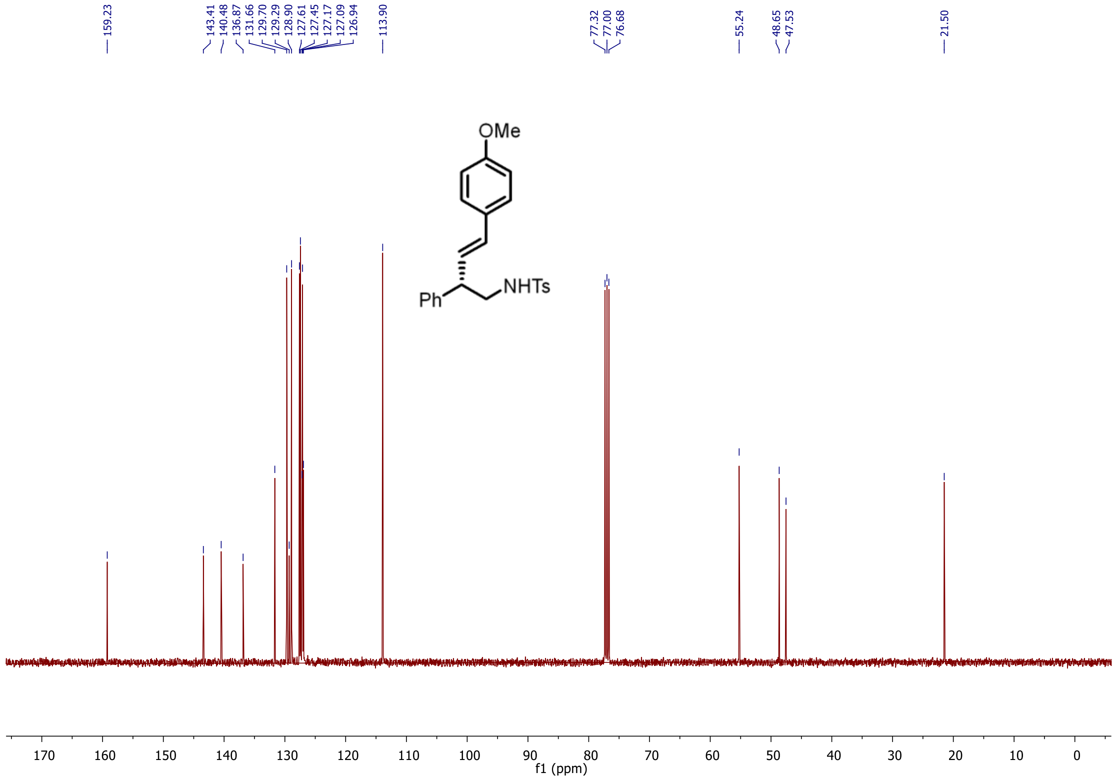

(*S*, *E*)-*N*-(4-(4-((*tert*-Butyldimethylsilyl)oxy)phenyl)-2-phenylbut-3-en-1-yl)-4-methylbenzenesulfonamide (17)

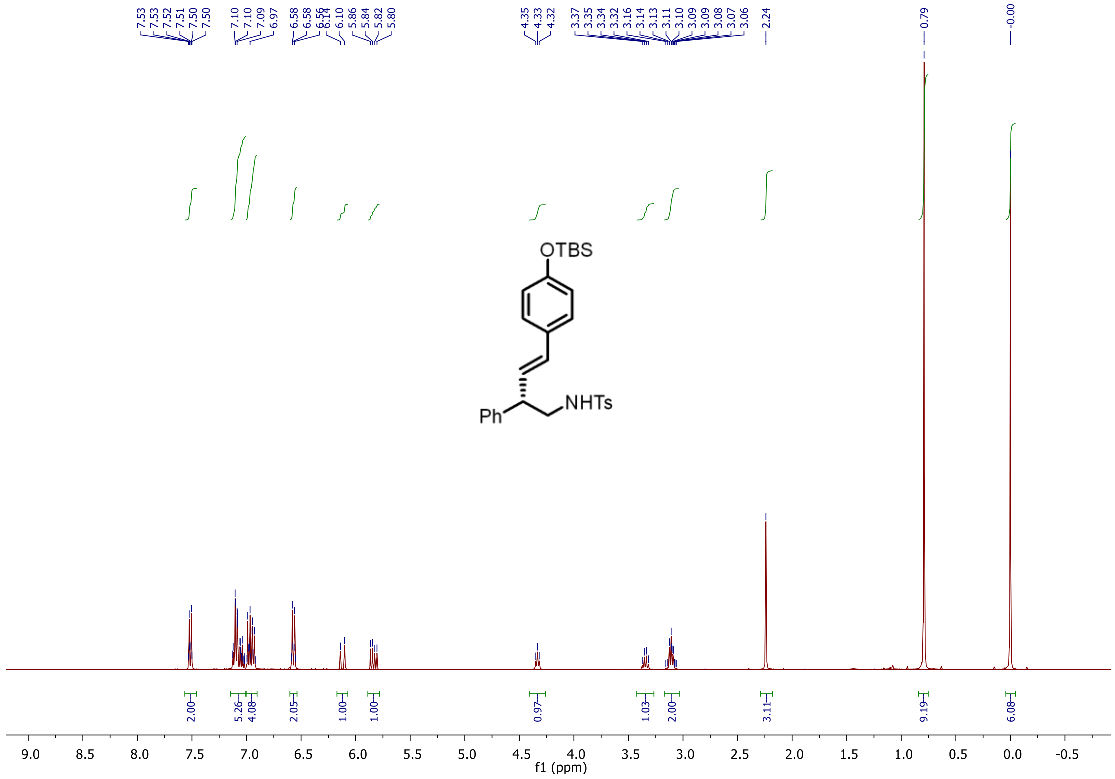

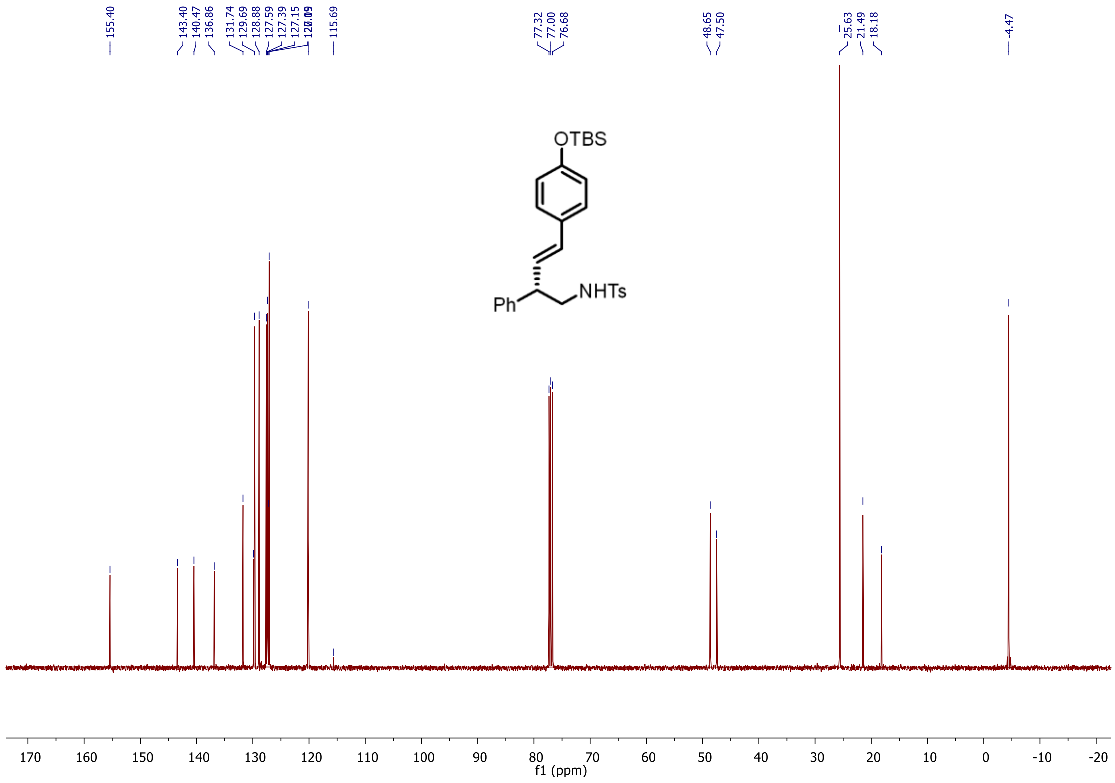

(*S*, *E*)-4-(4-((4-Methylphenyl)sulfonamido)-3-phenylbut-1-en-1-yl)phenyl acetate (18)

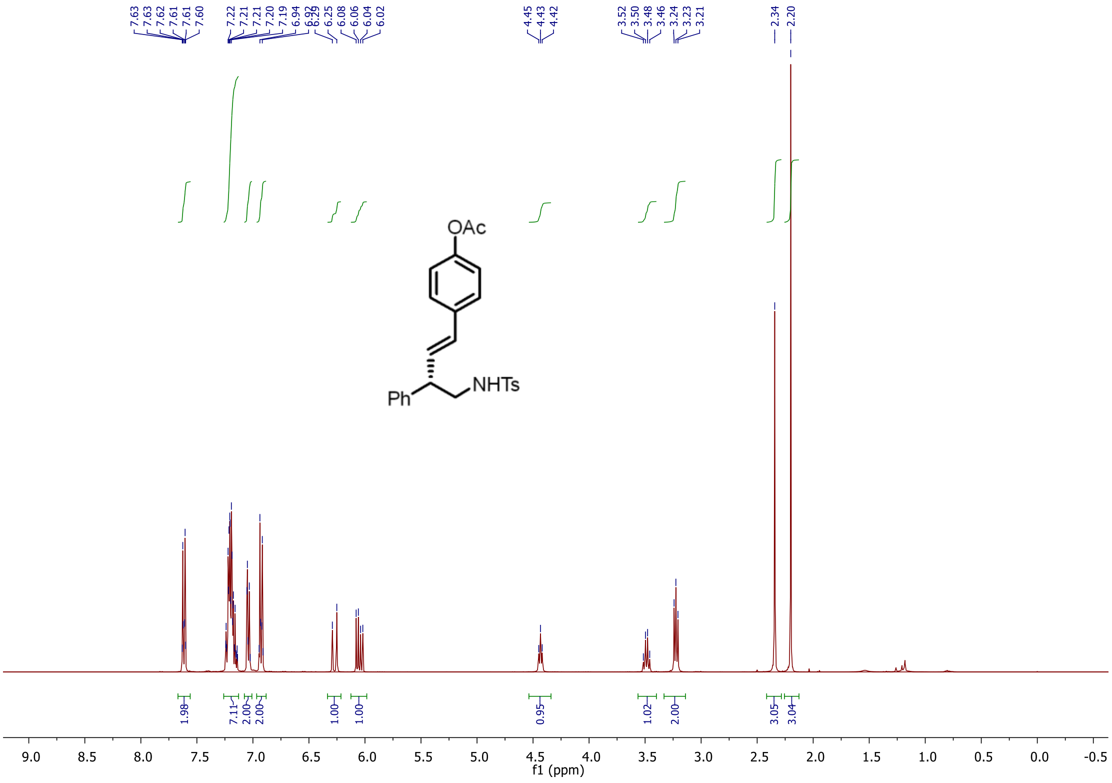

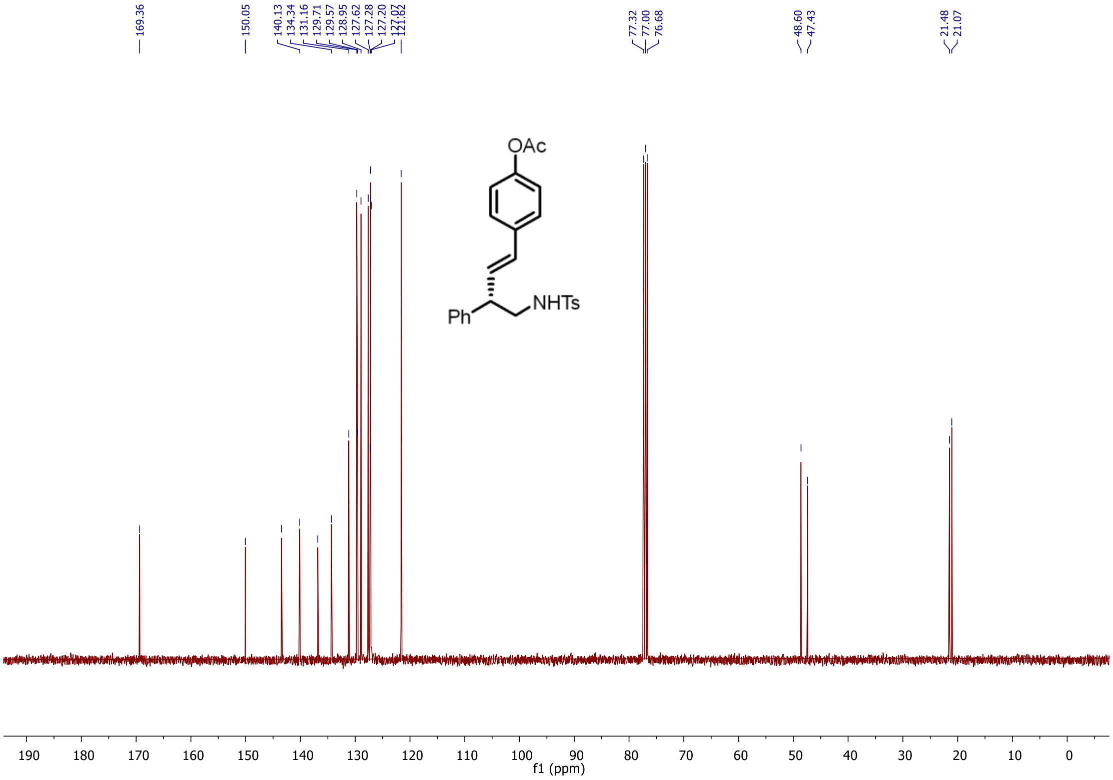

(*S*, *E*)-4-Methyl-*N*-(2-phenyl-4-(4-(trifluoromethoxy)phenyl)but-3-en-1-yl)benzenesulfonamide (19)

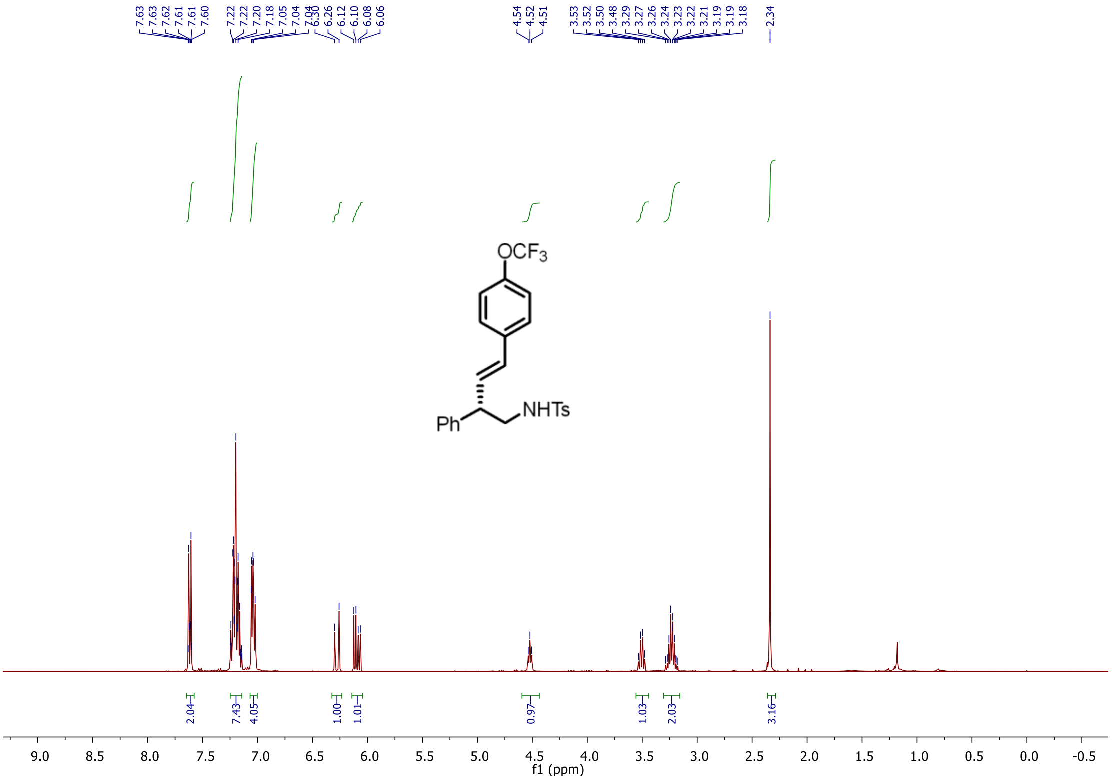

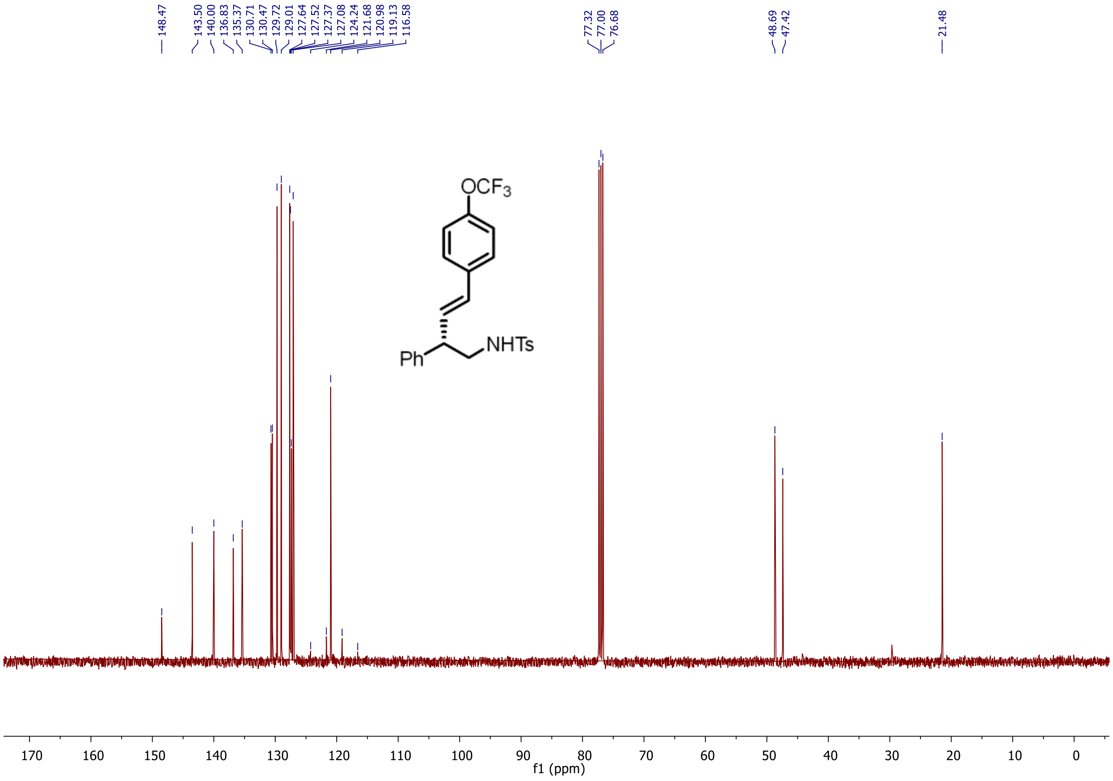

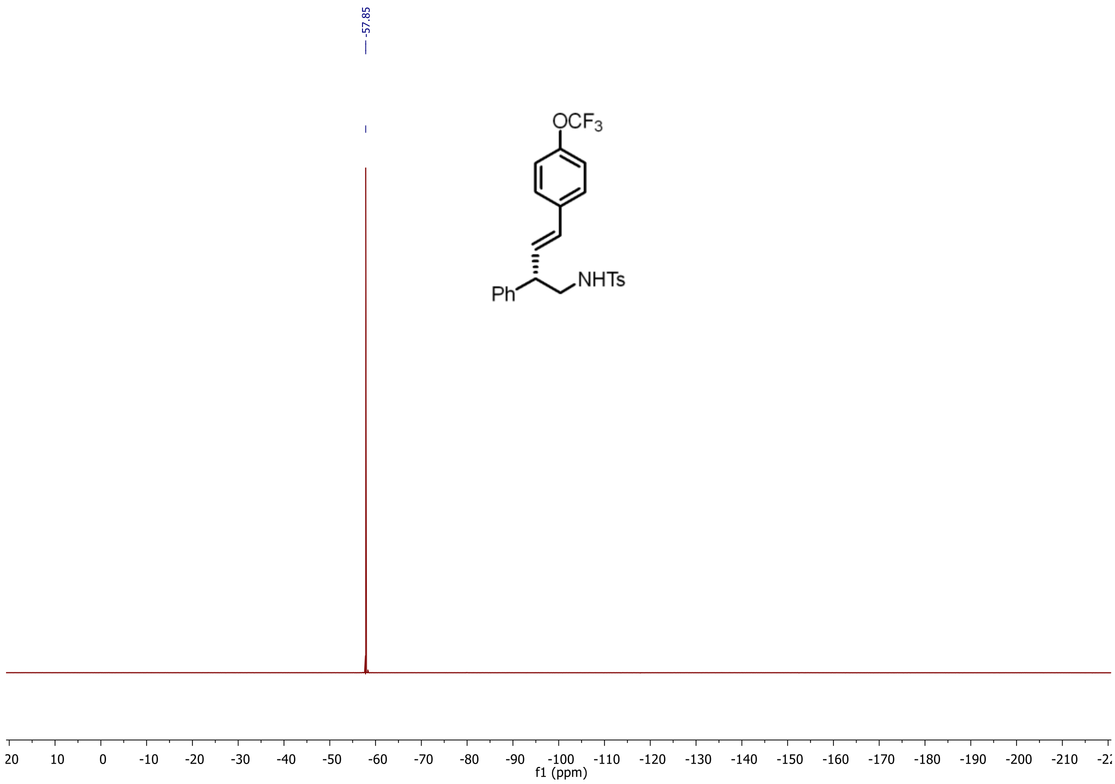

(*S*, *E*)-*N*-(4-(4-Fluorophenyl)-2-phenylbut-3-en-1-yl)-4-methylbenzenesulfonamide (20)

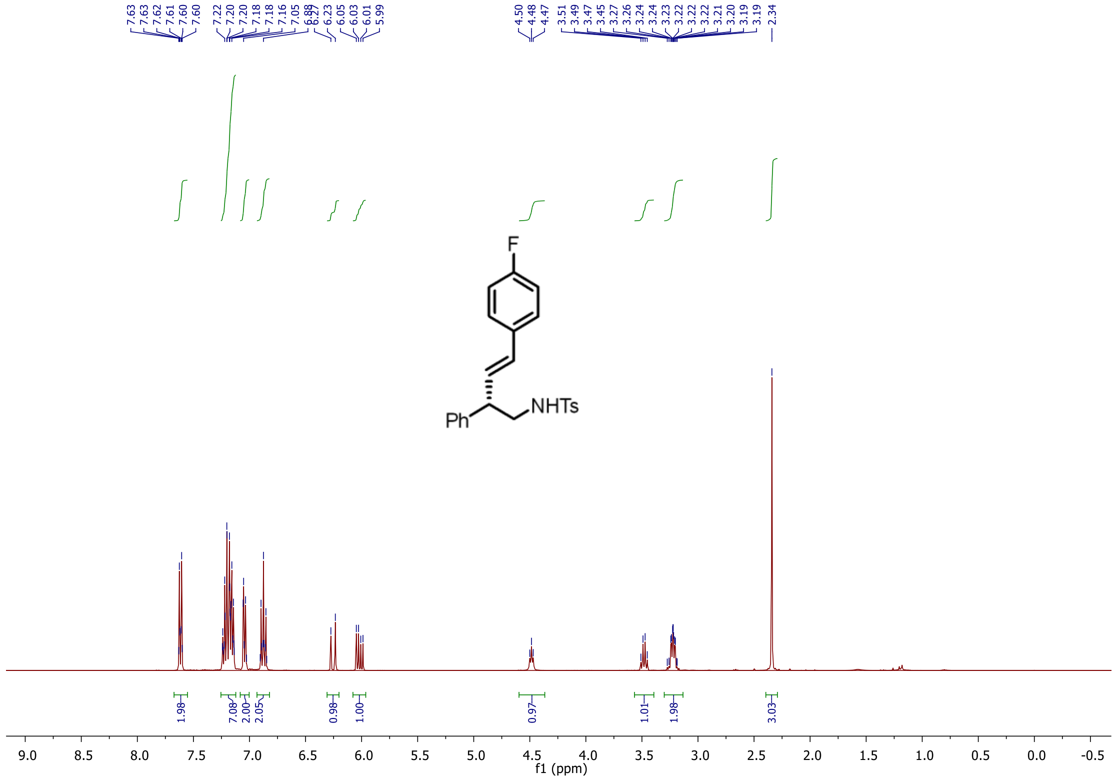

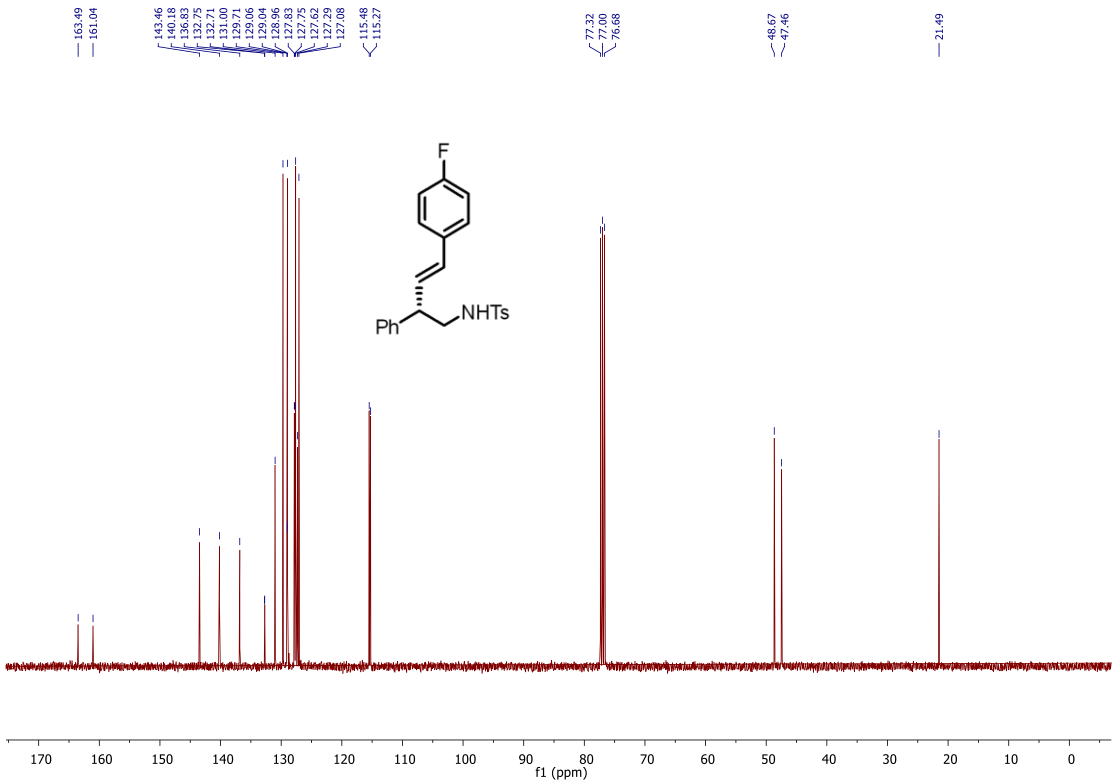
163.49
161.04
143.46
140.18
136.83
132.75
132.71
131.00
129.71
129.06
129.04
128.96
127.83
127.75
127.62
127.29
127.08
115.48
115.27
77.32
77.00
76.68
48.67
47.46
21.49
F
Ph
NHTs
f1 (ppm)

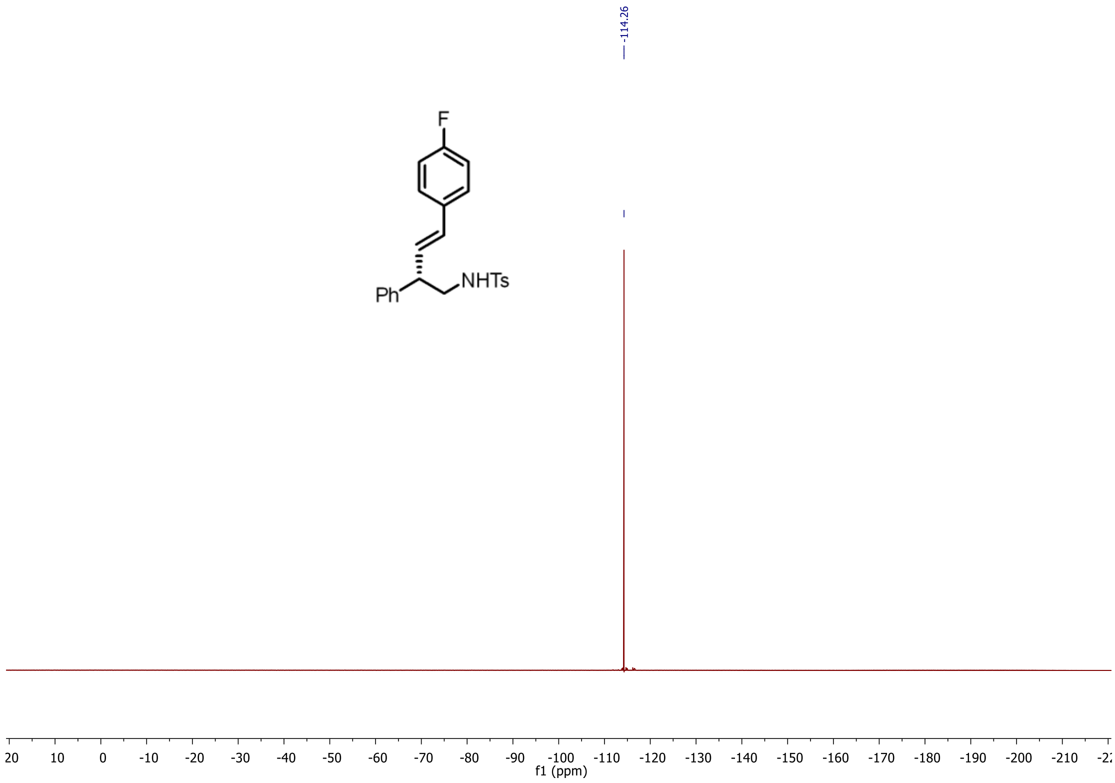
-114.26
F
Ph
NHTs
f1 (ppm)

(*S*, *E*)-*N*-(4-(4-Chlorophenyl)-2-phenylbut-3-en-1-yl)-4-methylbenzenesulfonamide (21)

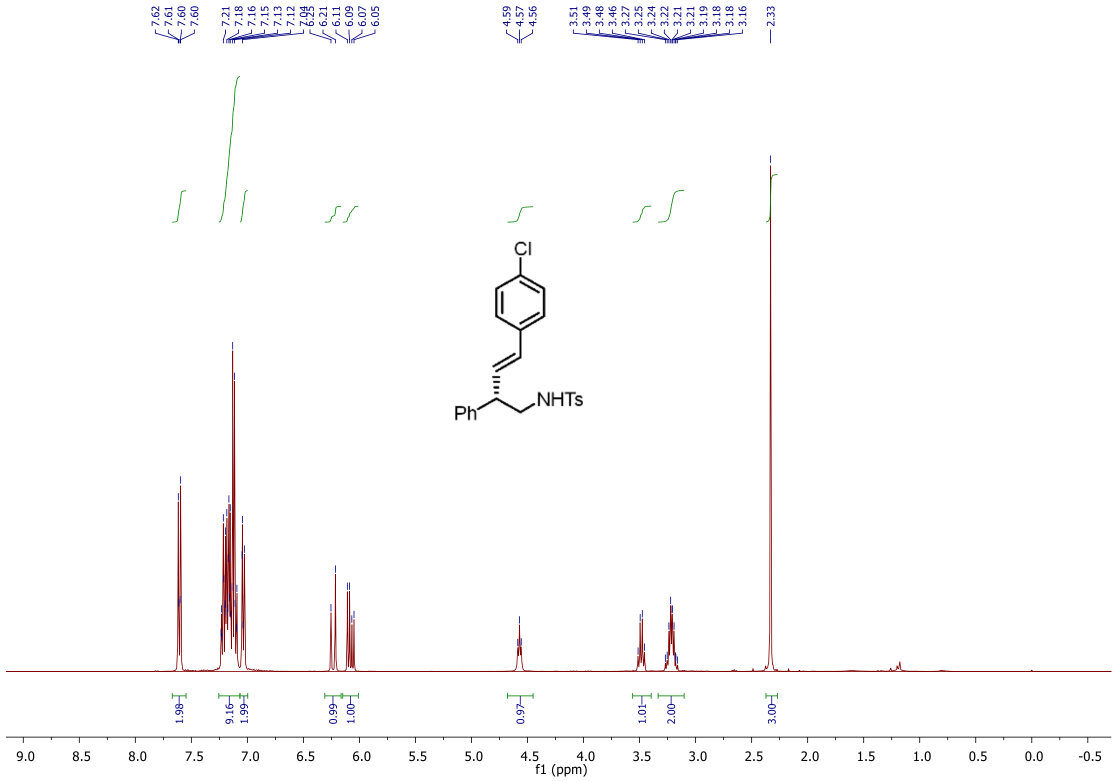

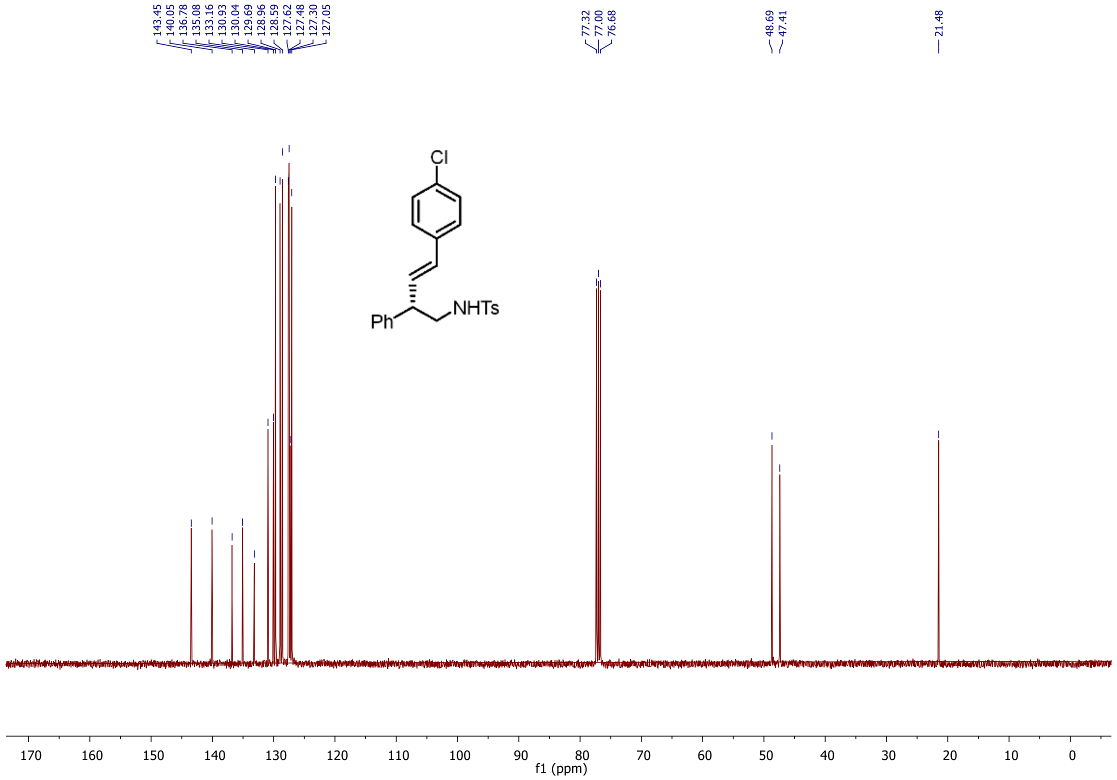

(*S*, *E*)-4-Methyl-*N*-(2-phenyl-4-(4-(trifluoromethyl)phenyl)but-3-en-1-yl)benzenesulfonamide (22)

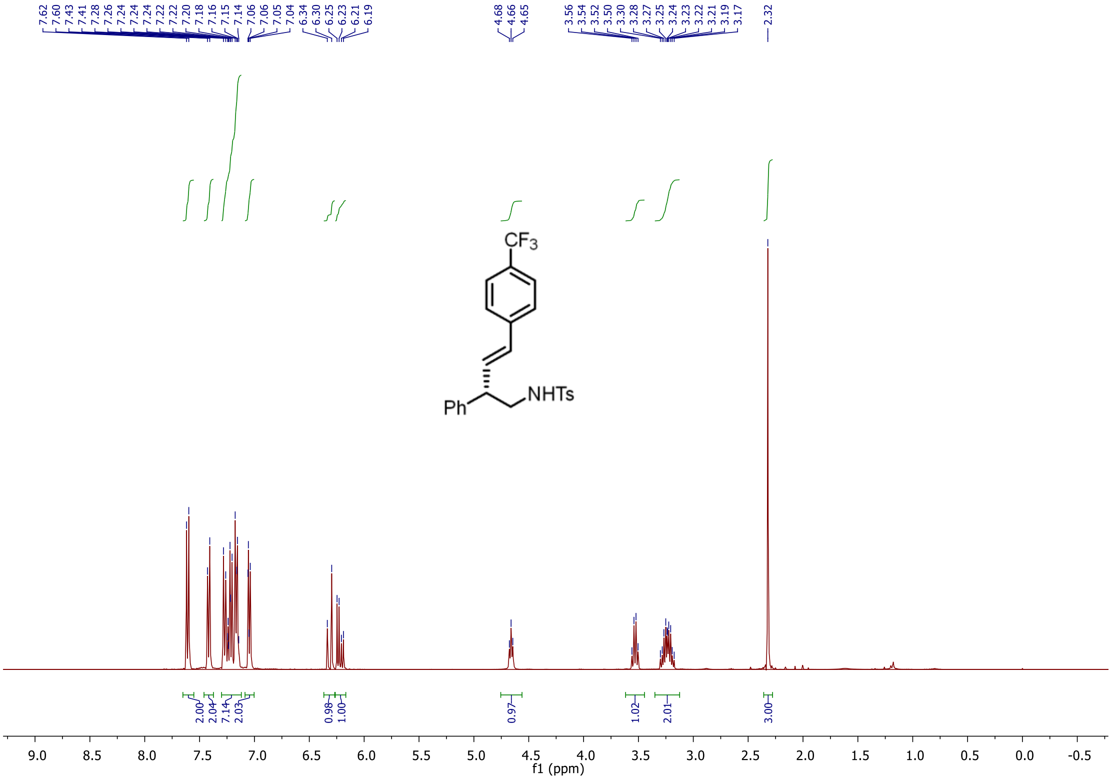

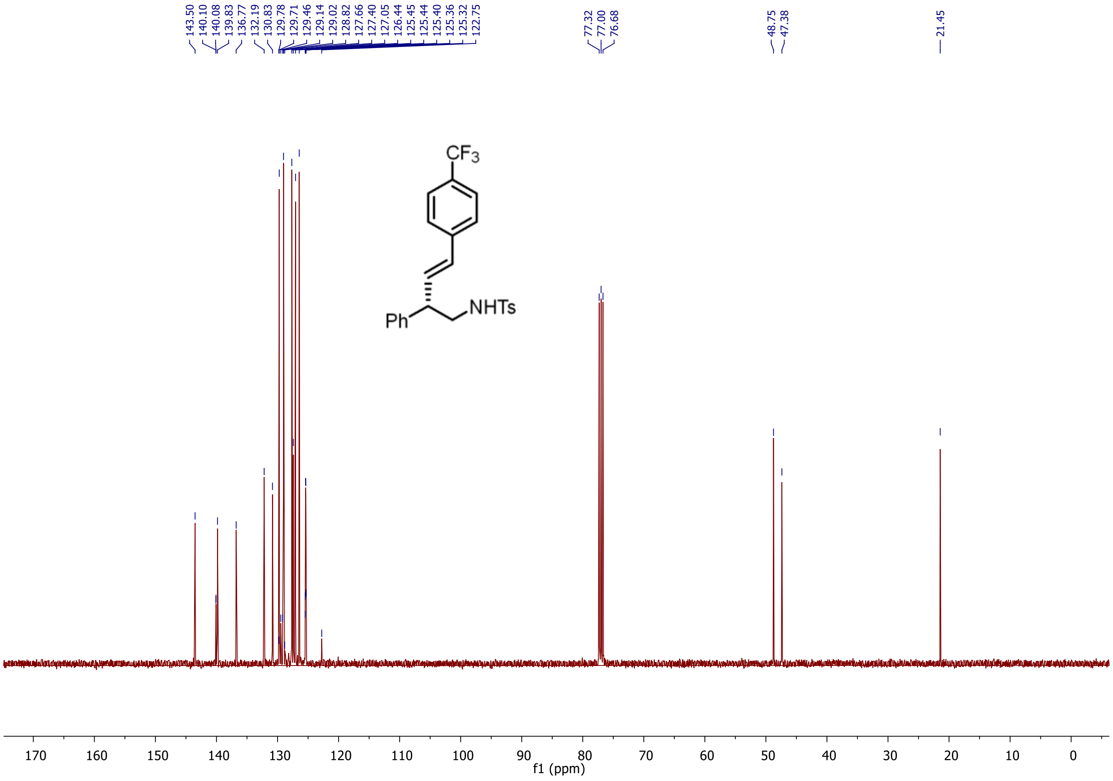

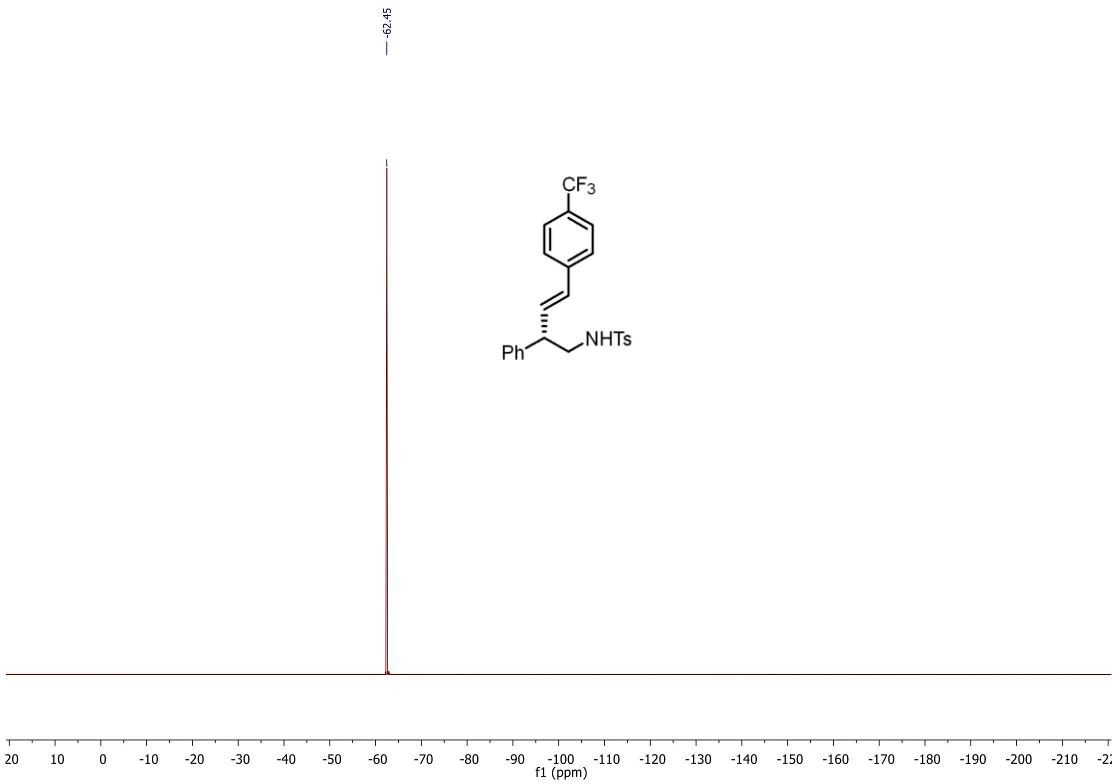

(*S*, *E*)-4-Methyl-*N*-(2-phenyl-4-(4-(4,4,5,5-tetramethyl-1,3,2-dioxaborolan-2-yl)phenyl)but-3-en-1-yl)benzenesulfonamide (23)

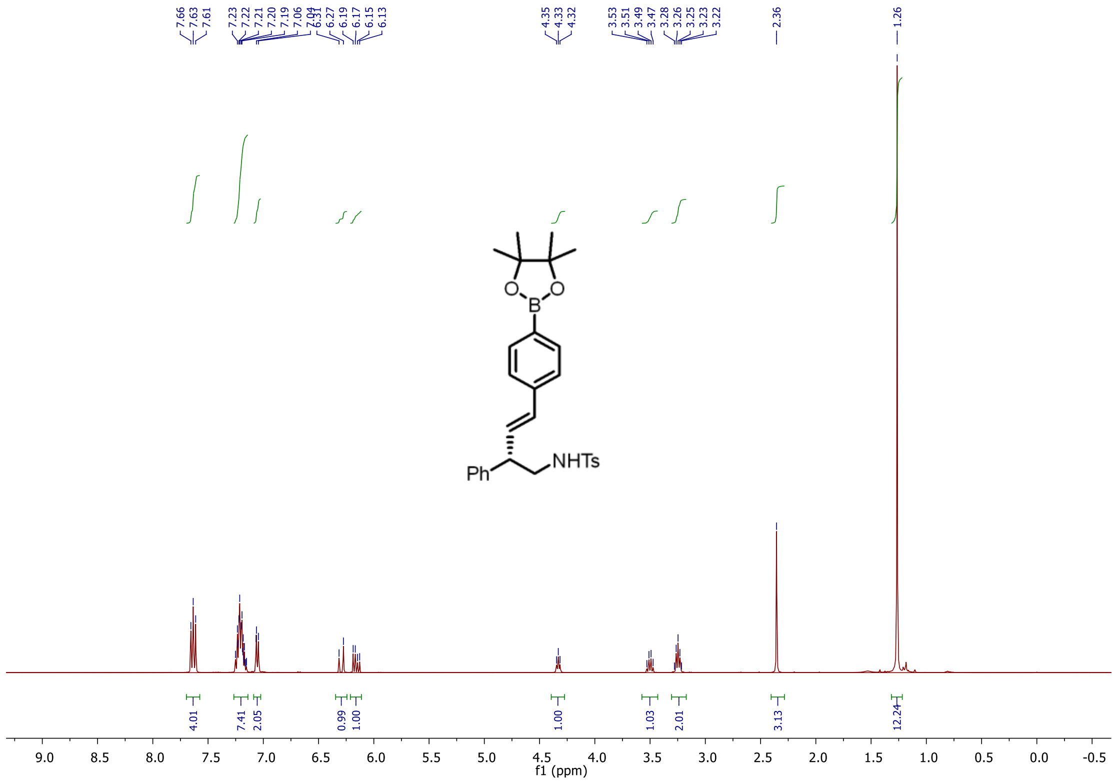

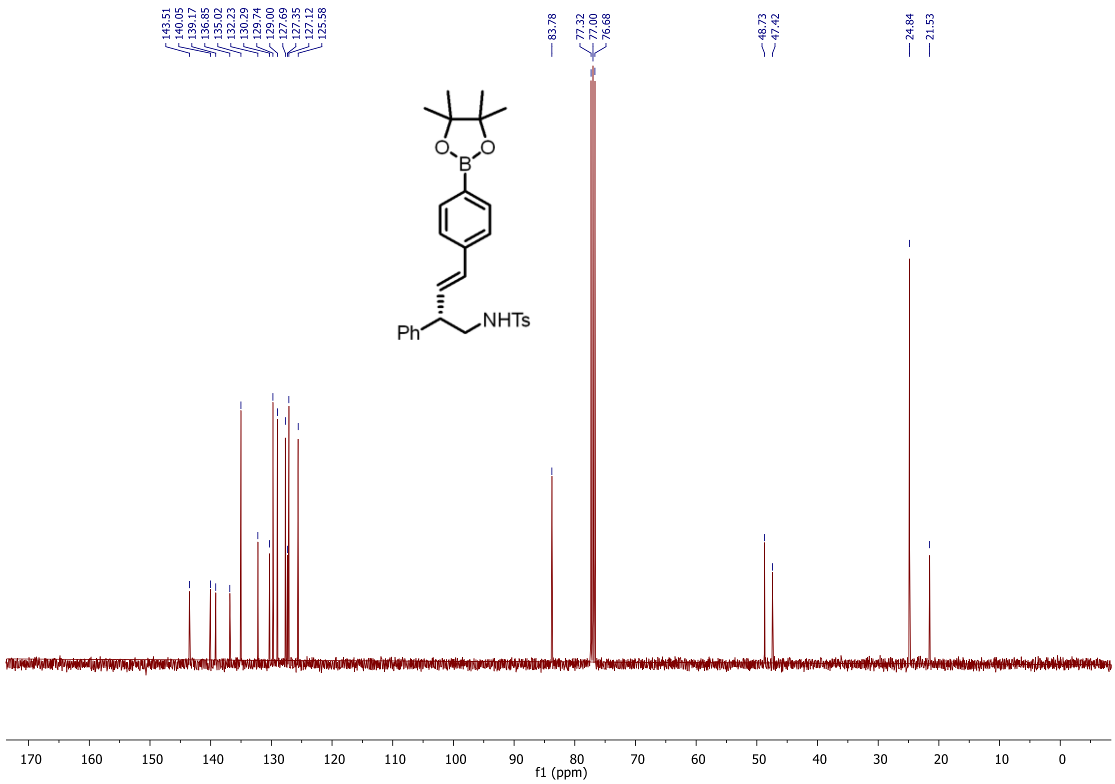

(*S*, *E*)-*N*-(4-(2-Fluorophenyl)-2-phenylbut-3-en-1-yl)-4-methylbenzenesulfonamide (24)

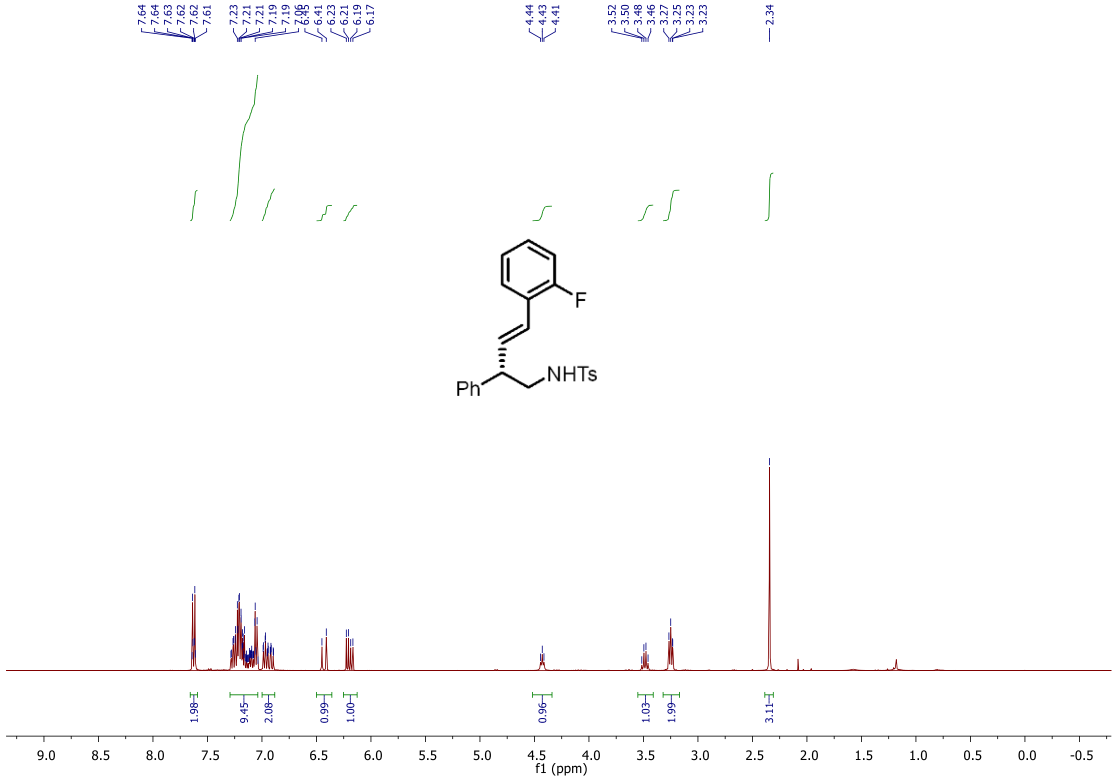

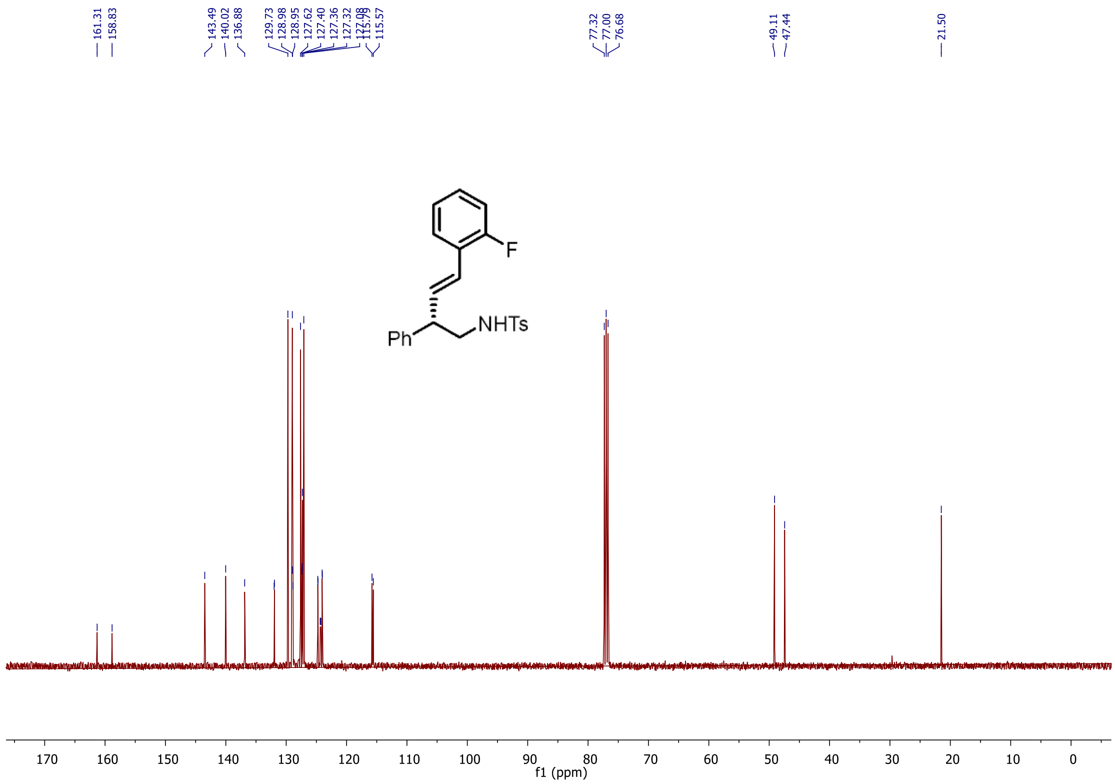
161.31
158.83
143.49
140.02
136.88
129.73
128.98
128.95
127.62
127.40
127.36
127.32
115.57
77.32
77.00
76.68
49.11
47.44
21.50
F
NHTs
Ph
f1 (ppm)

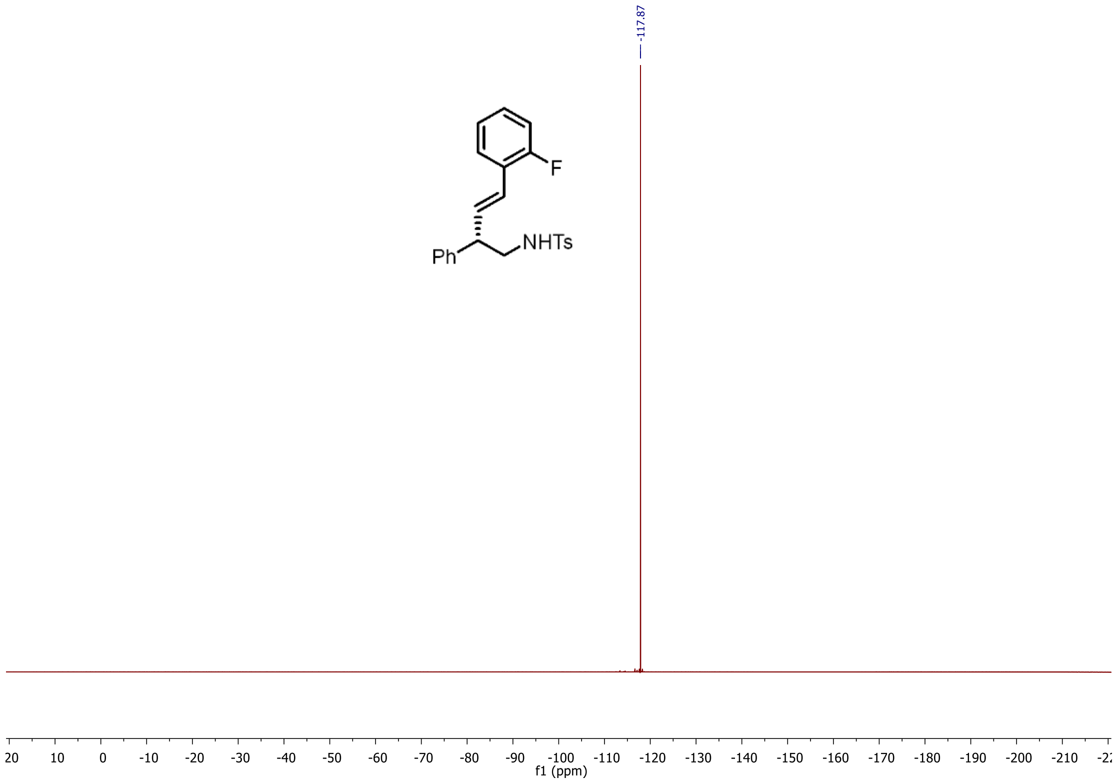
-117.87
F
NHTs
Ph
f1 (ppm)

(*S*, *E*)-*N*-(4-(3-Fluorophenyl)-2-phenylbut-3-en-1-yl)-4-methylbenzenesulfonamide (25)

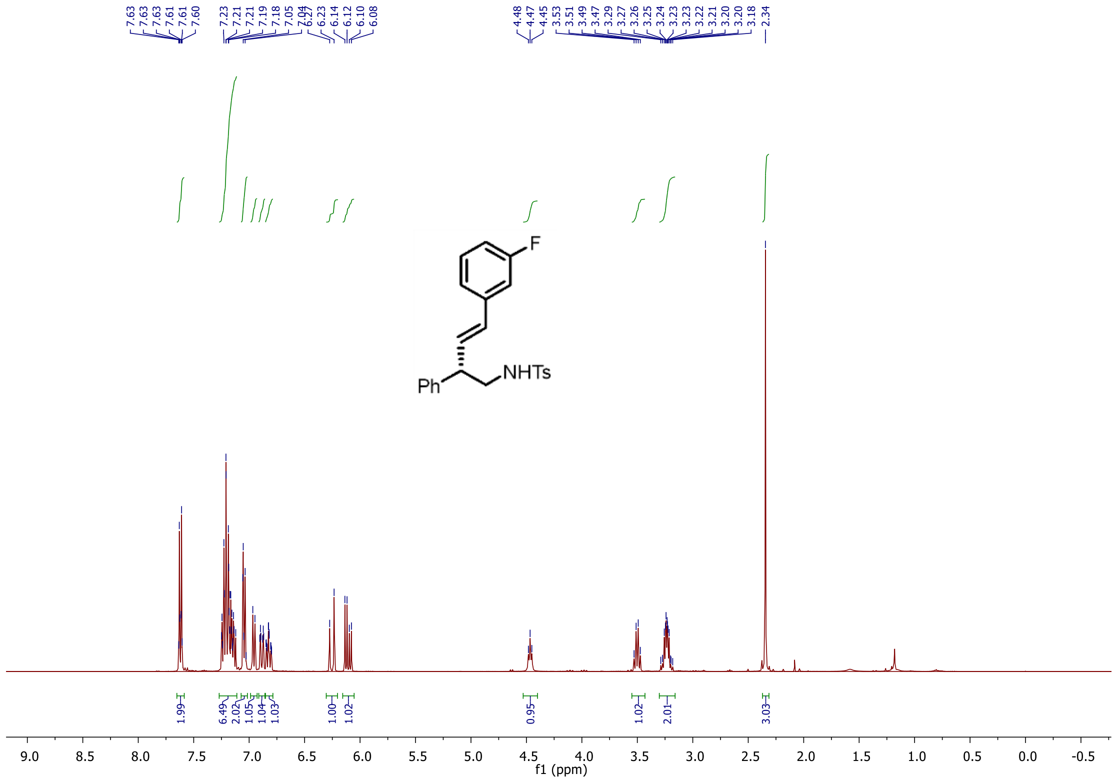

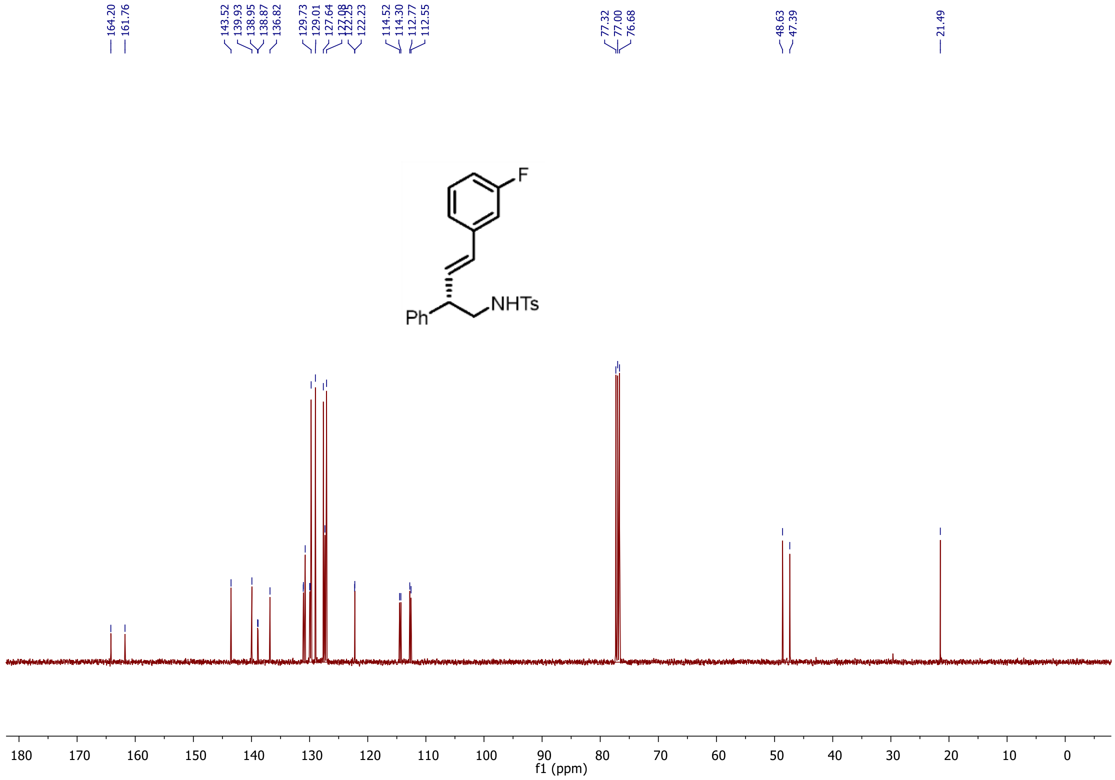

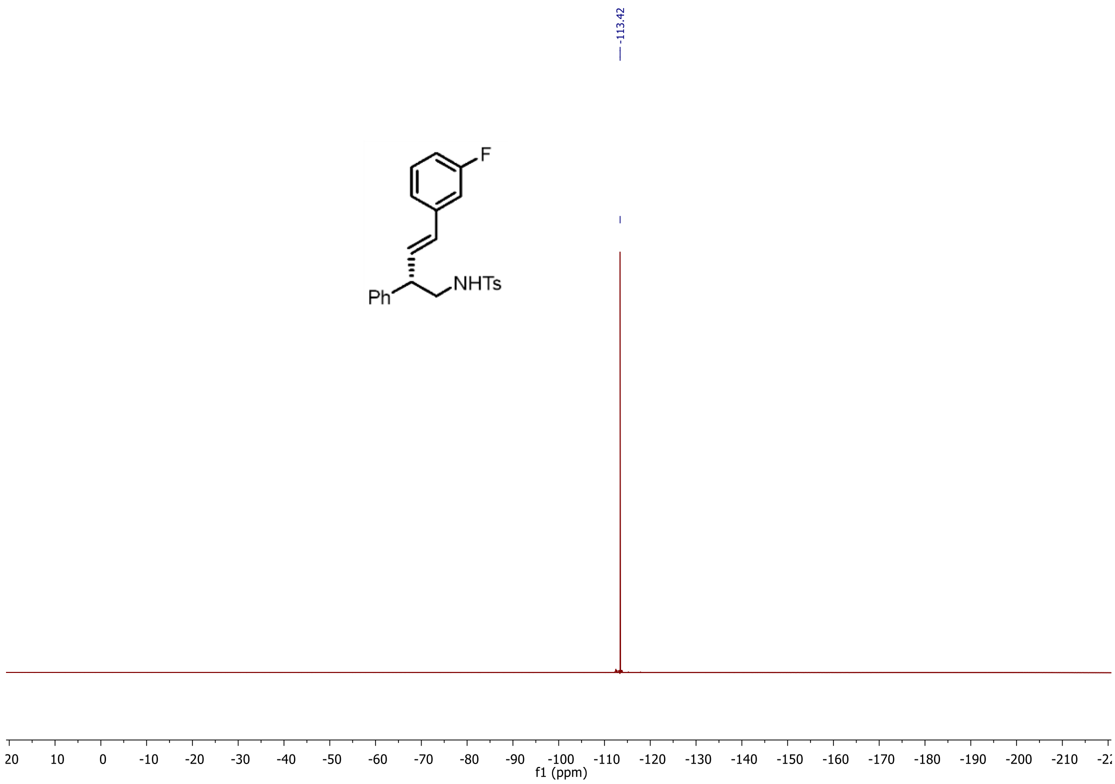

(*S*, *E*)-4-Methyl-*N*-(4-(naphthalen-2-yl)-2-phenylbut-3-en-1-yl)benzenesulfonamide (26)

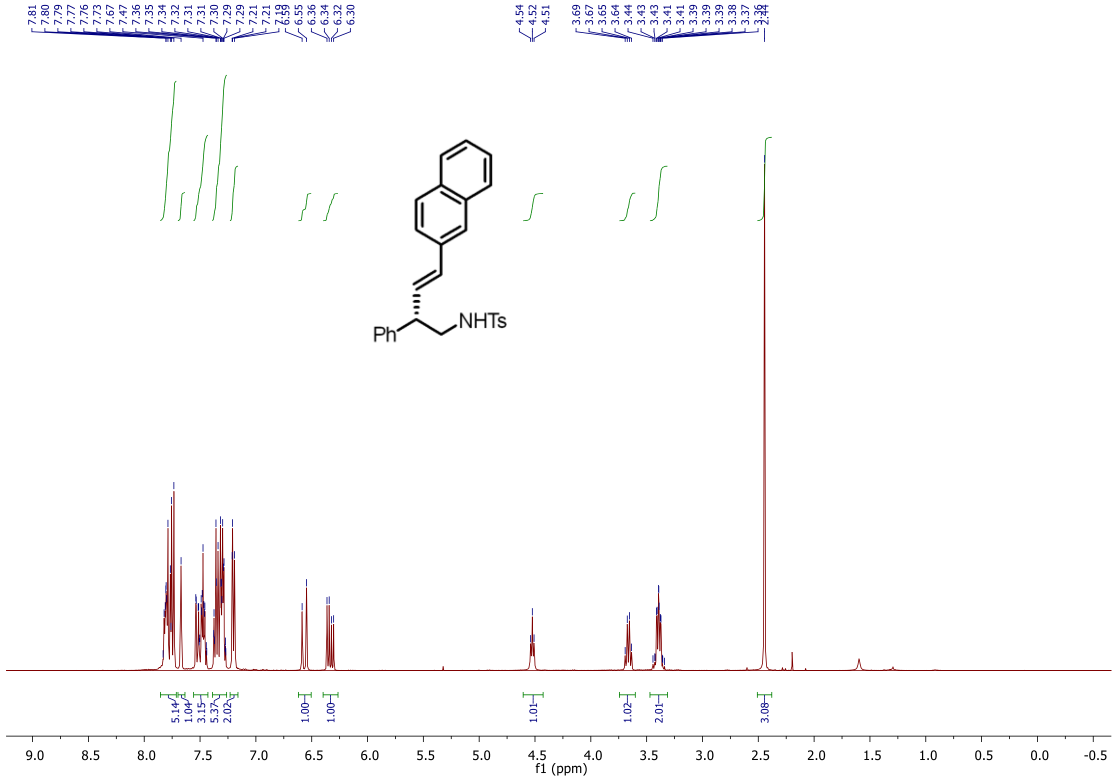

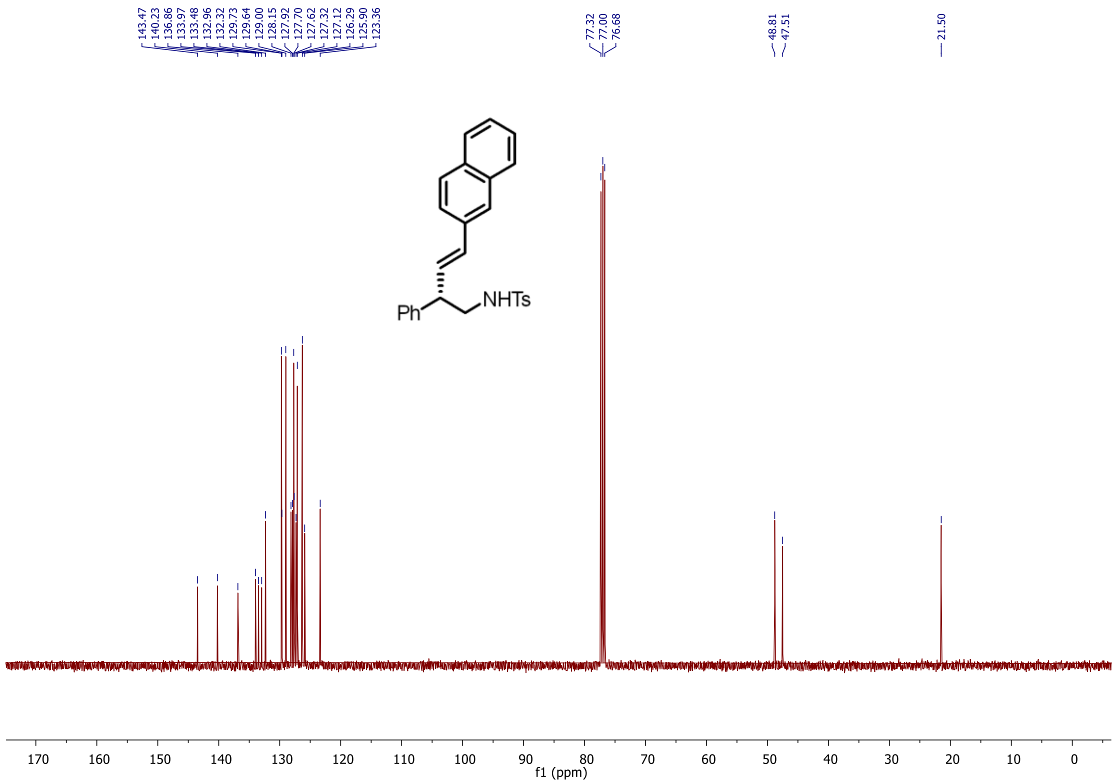

(*S*, *E*)-*N*-(4-(6-Methoxypyridin-3-yl)-2-phenylbut-3-en-1-yl)-4-methylbenzenesulfonamide (27)

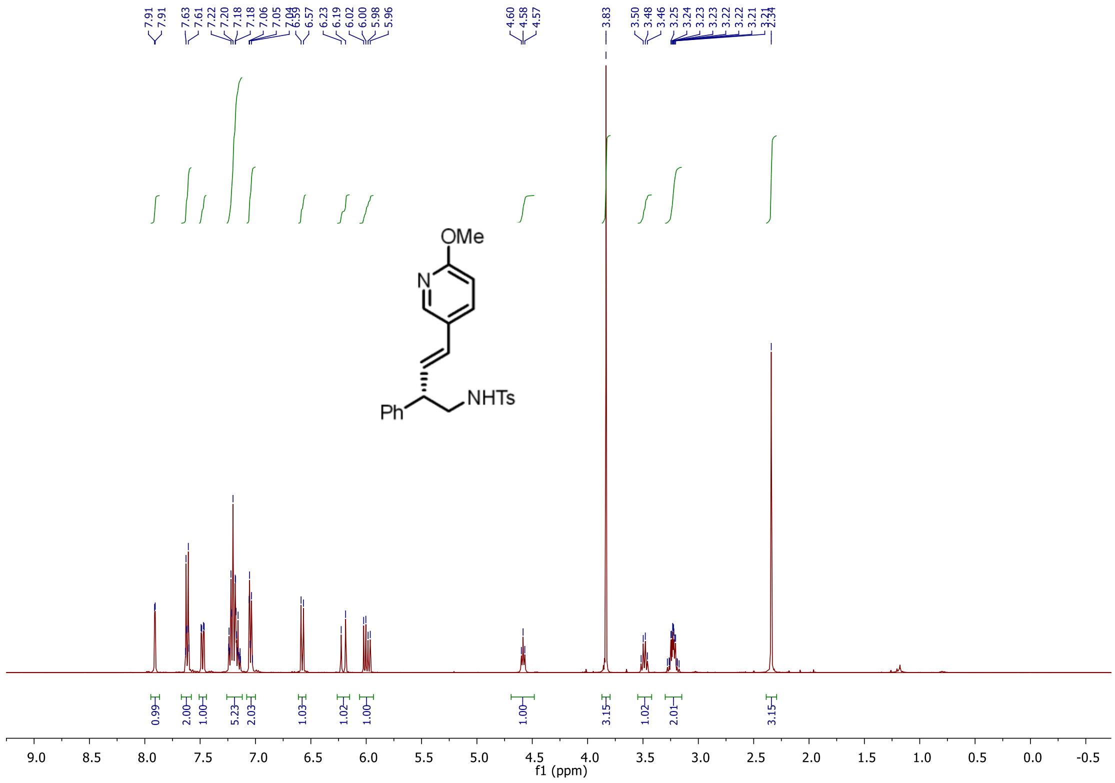

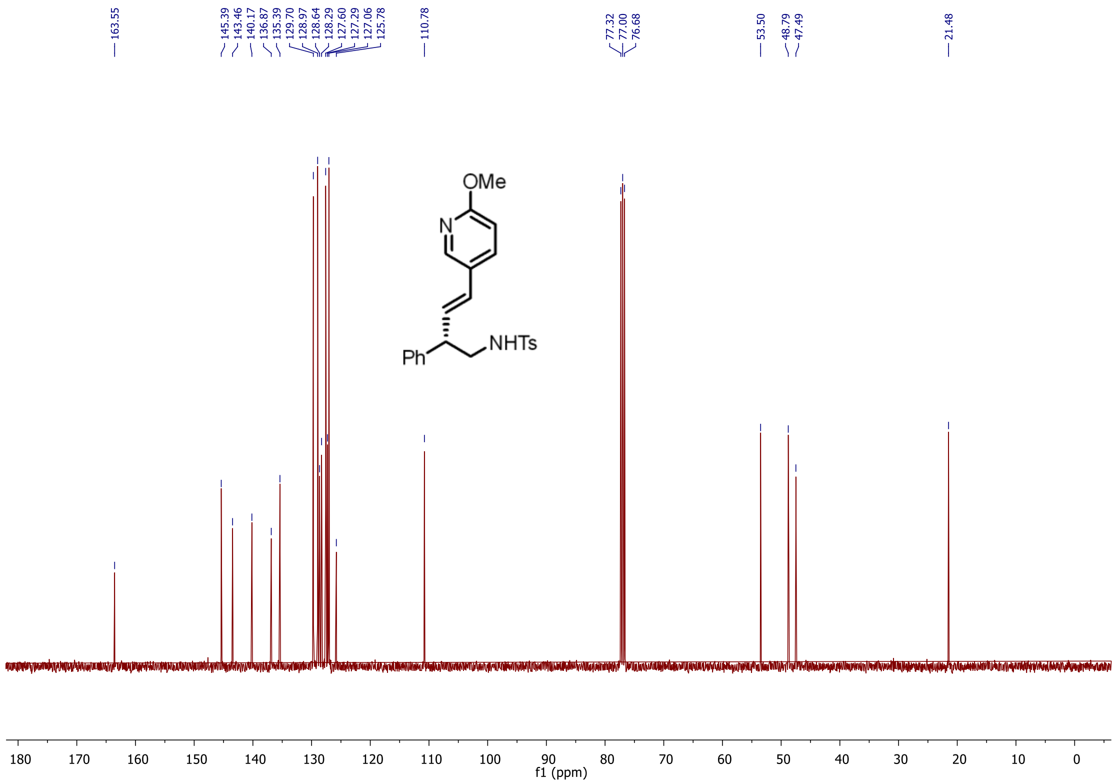

(*S*, *E*)-*N*-(4-(2-Methoxypyrimidin-5-yl)-2-phenylbut-3-en-1-yl)-4-methylbenzenesulfonamide (28)

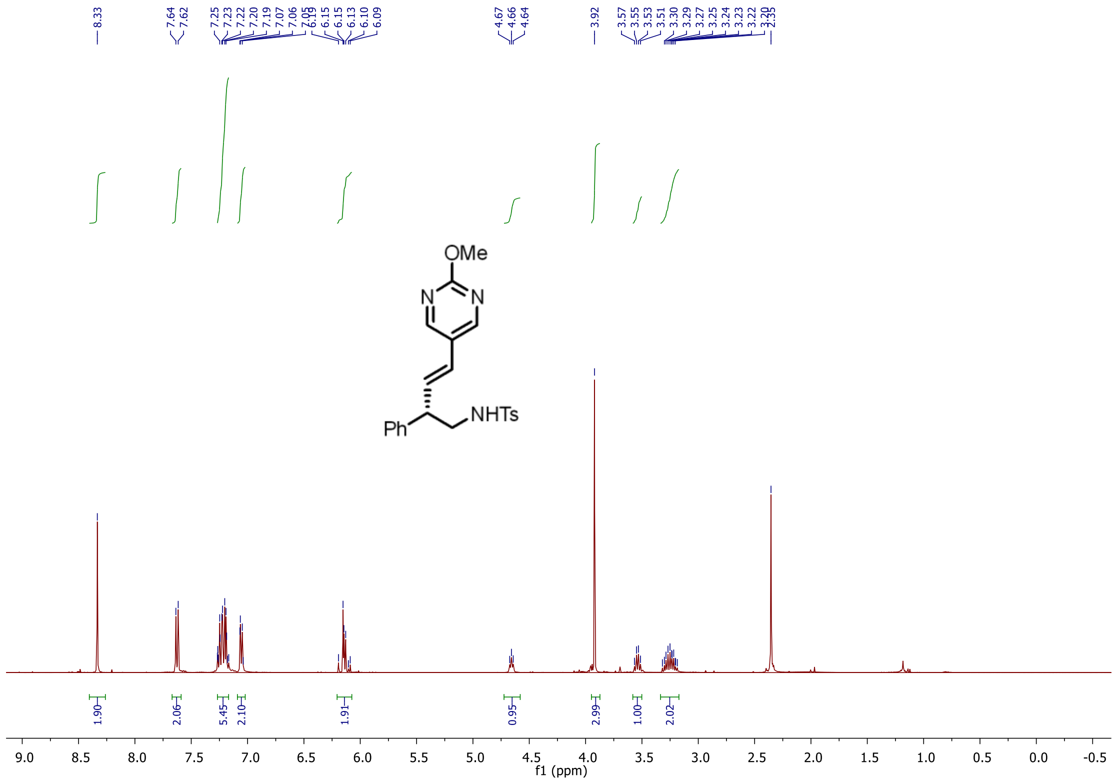

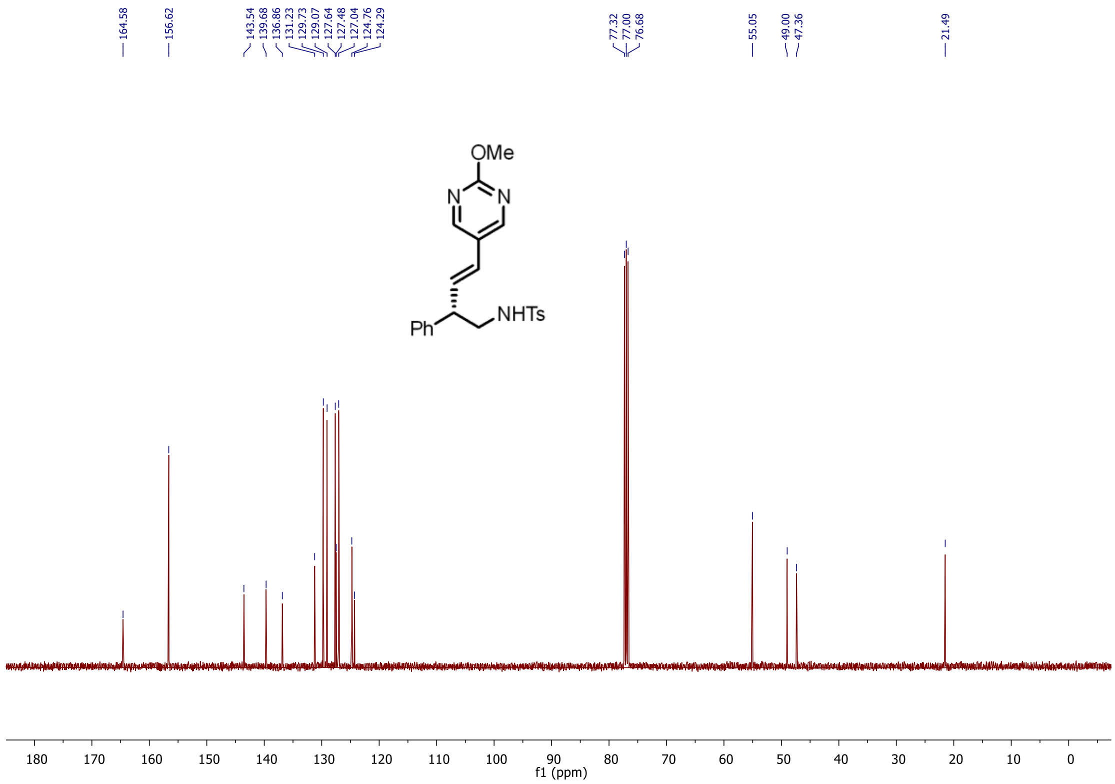

Benzyl (*S*, *E*)-(2, 4-diphenylbut-3-en-1-yl)carbamate (29)

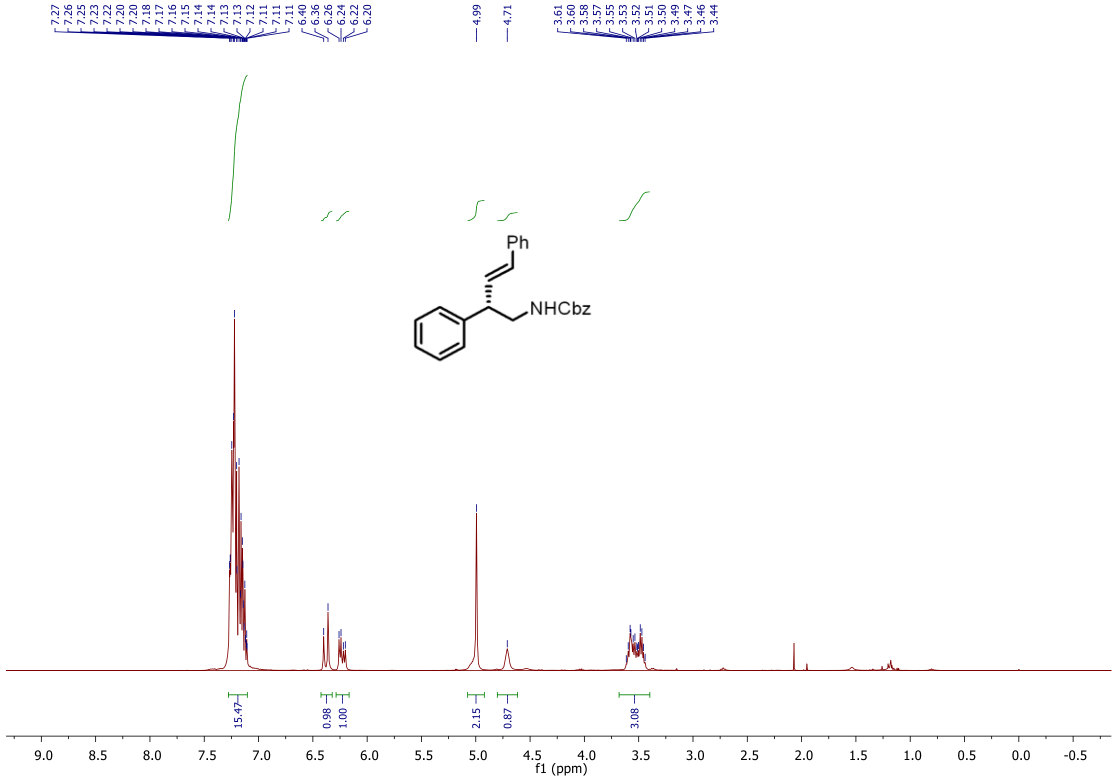

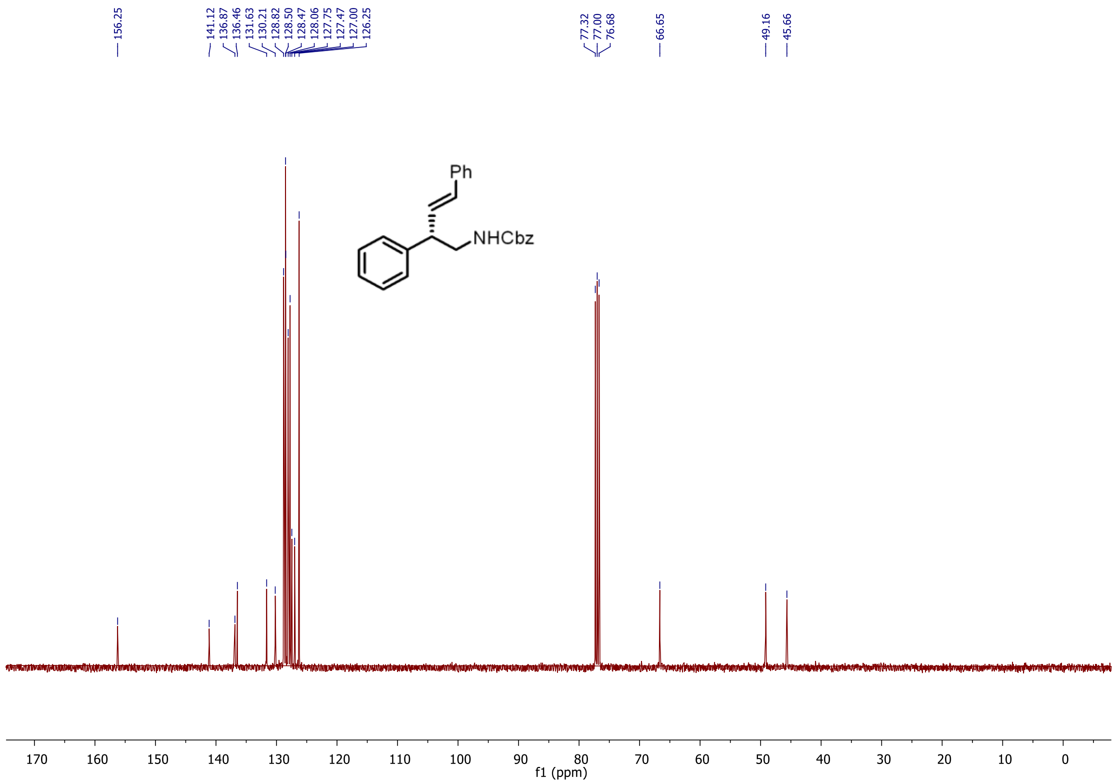

Benzyl (*S*, *E*)-(4-cyclohexyl-2-phenylbut-3-en-1-yl)carbamate (30)

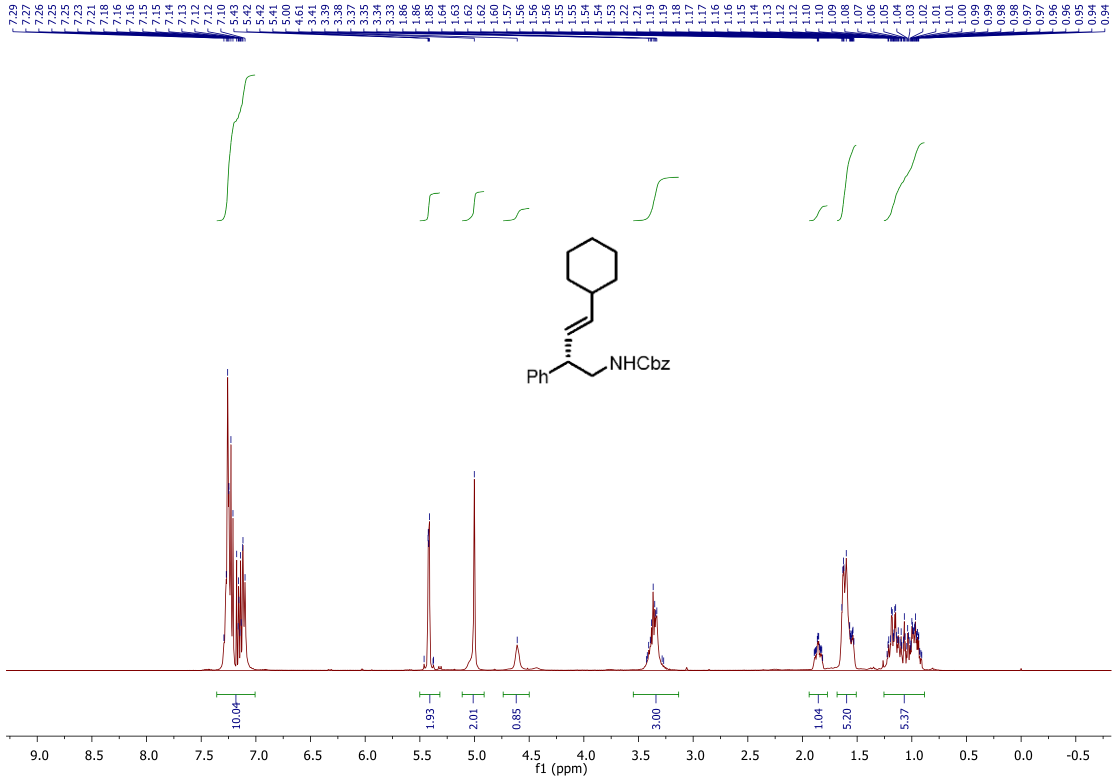

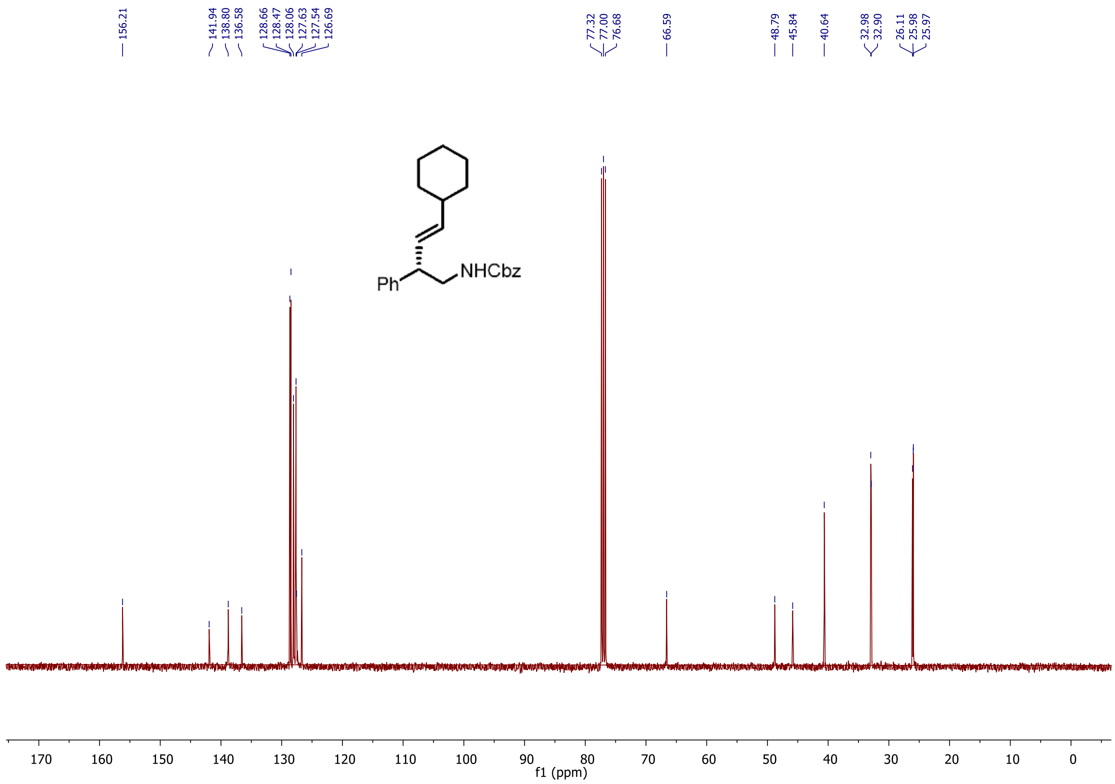

(*S*, *E*)-6-(((Benzyloxy)carbonyl)amino)-5-phenylhex-3-en-1-yl benzoate (31)

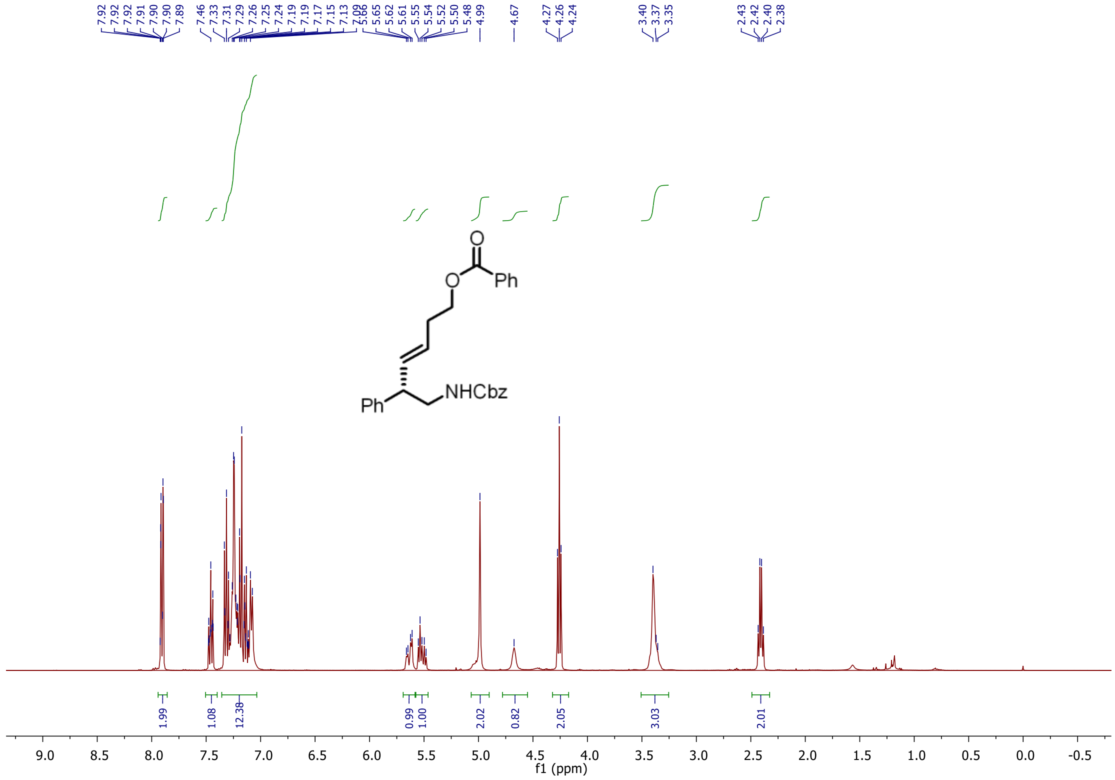

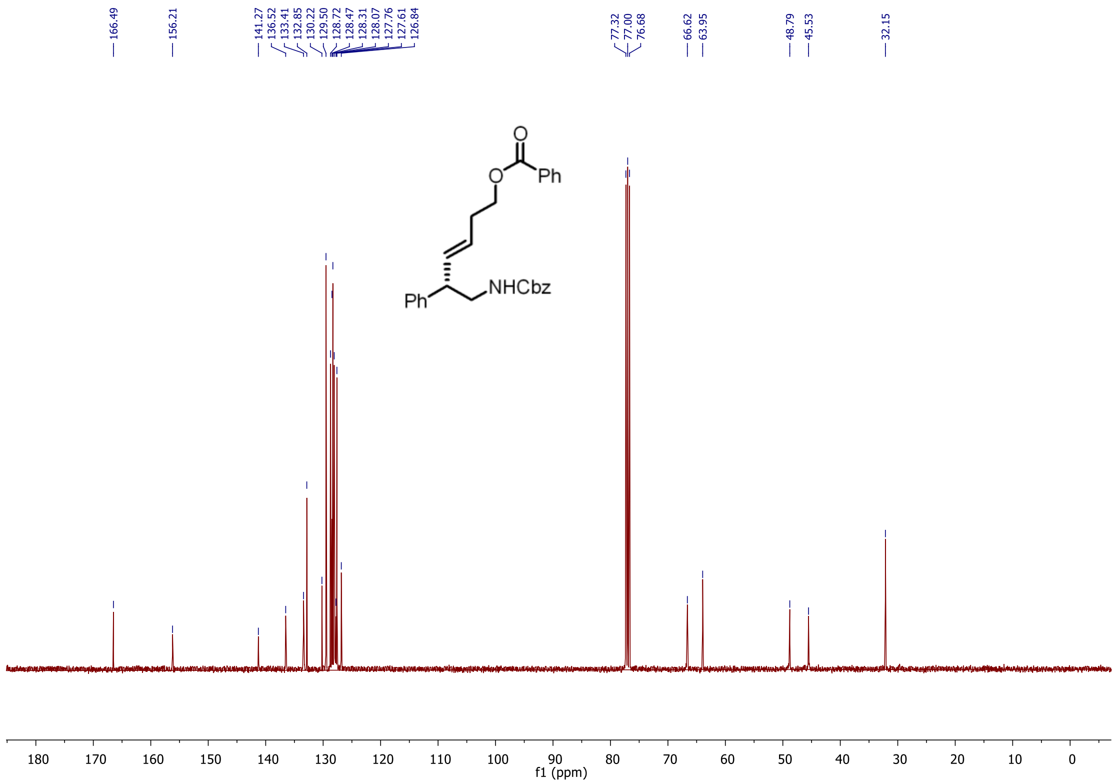

Benzyl (*S*, *E*)-(2-phenyl-4-(trimethylsilyl)but-3-en-1-yl)carbamate (32)

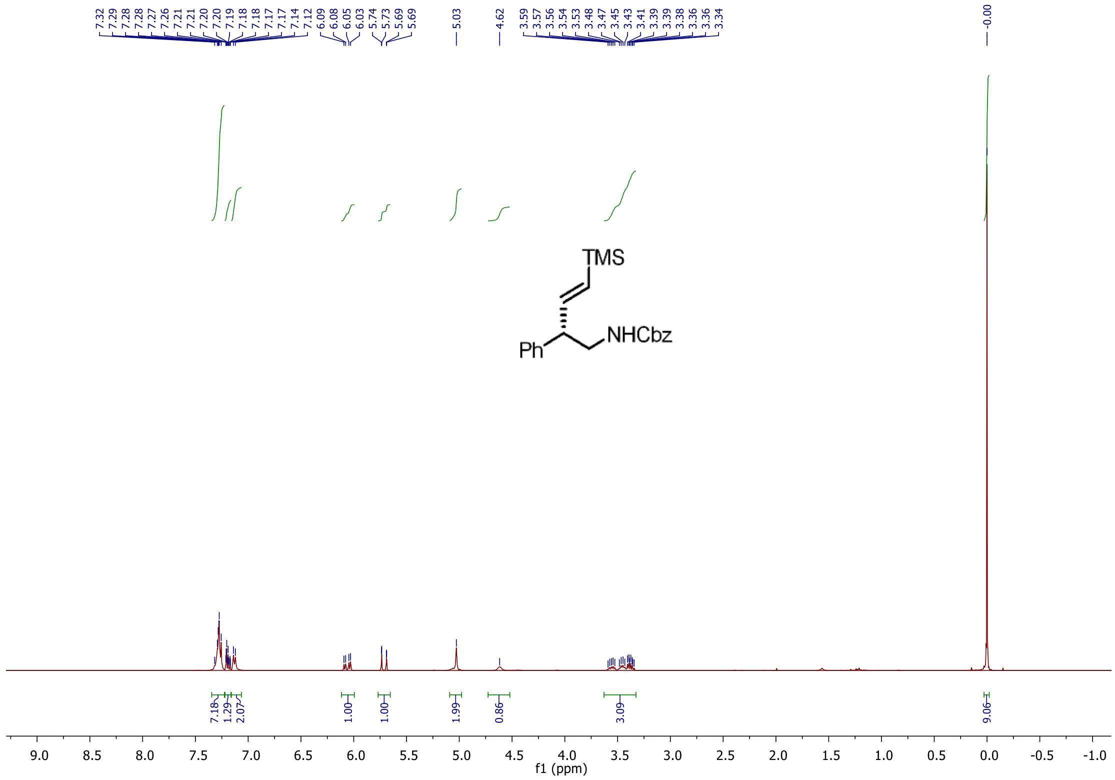

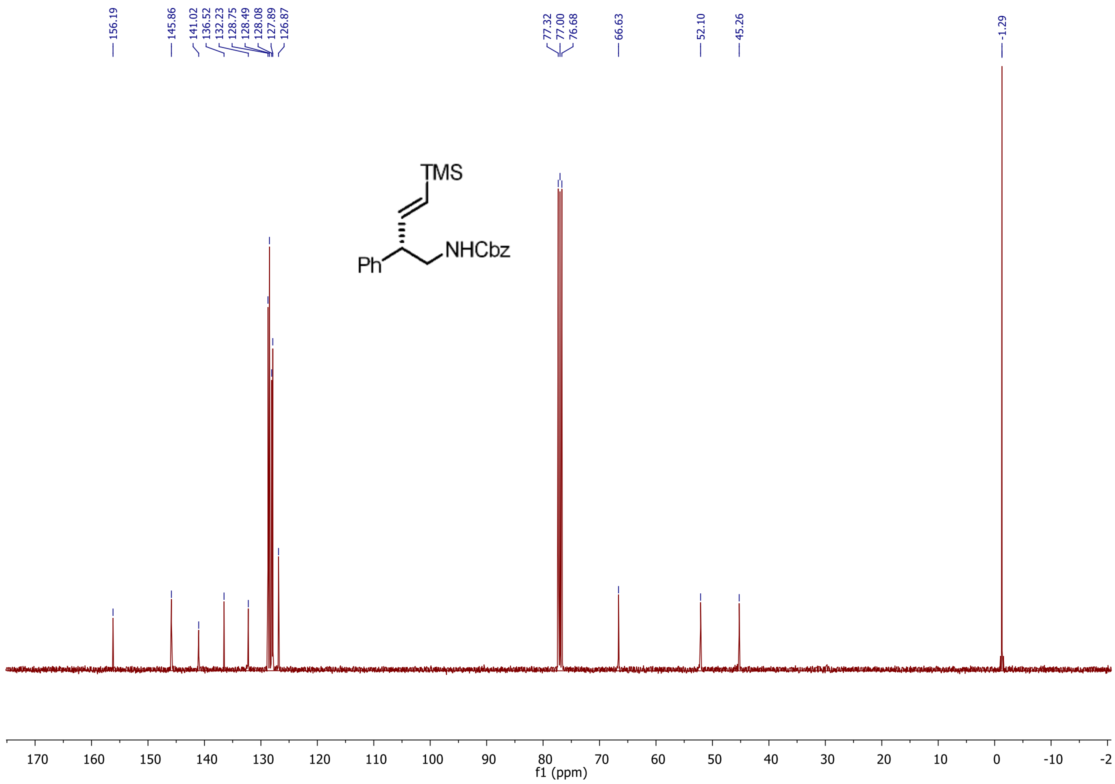

Benzyl ((S, 3*E*, 5*E*)-2,6-diphenylhexa-3,5-dien-1-yl)carbamate (33)

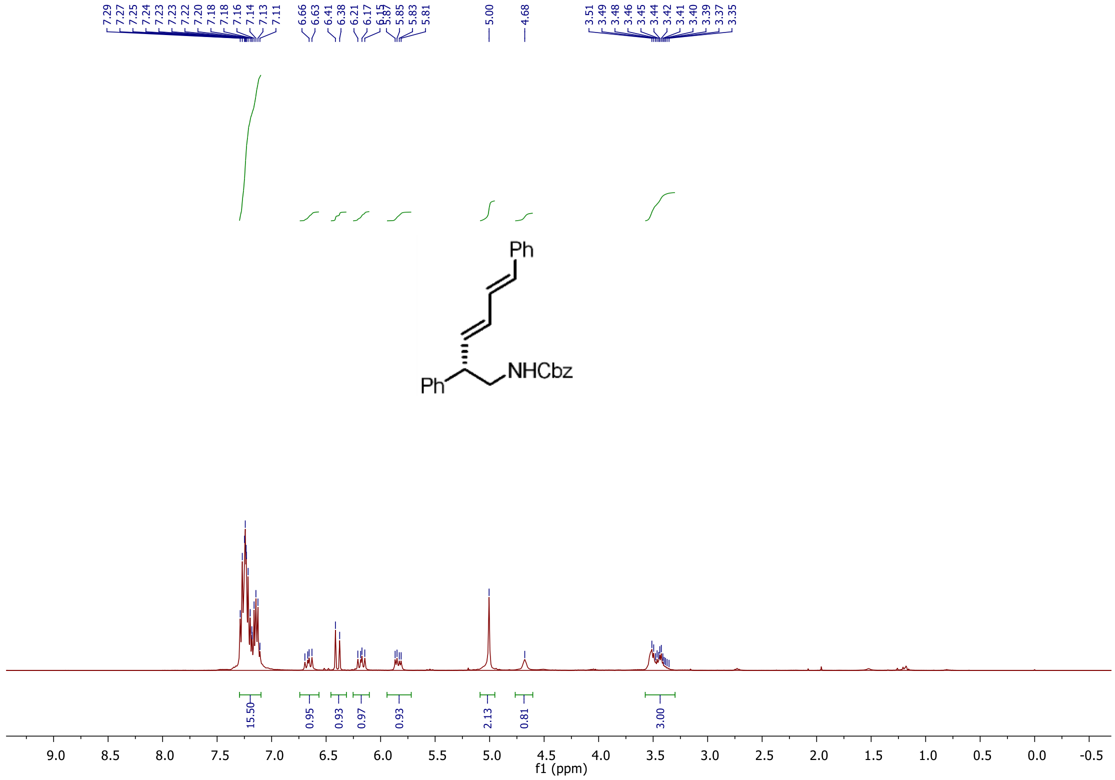

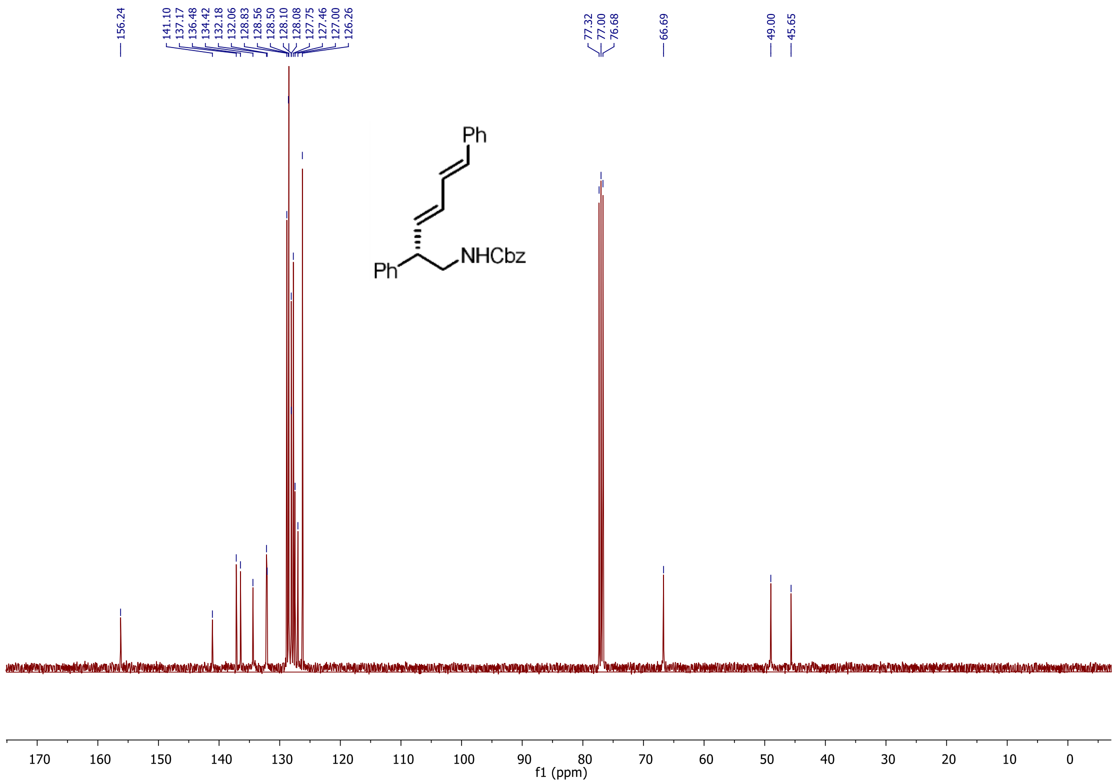

(*R*)-*N*-(2-(1H-Inden-2-yl)-2-phenylethyl)-4-methylbenzenesulfonamide (34)

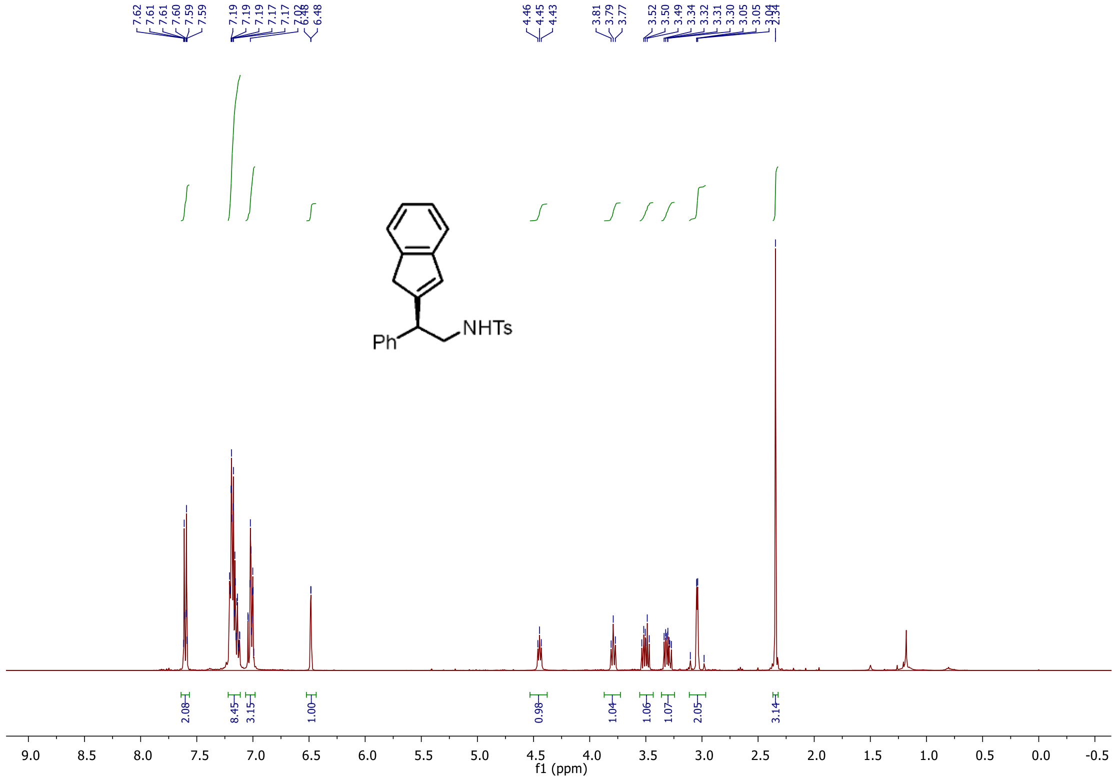

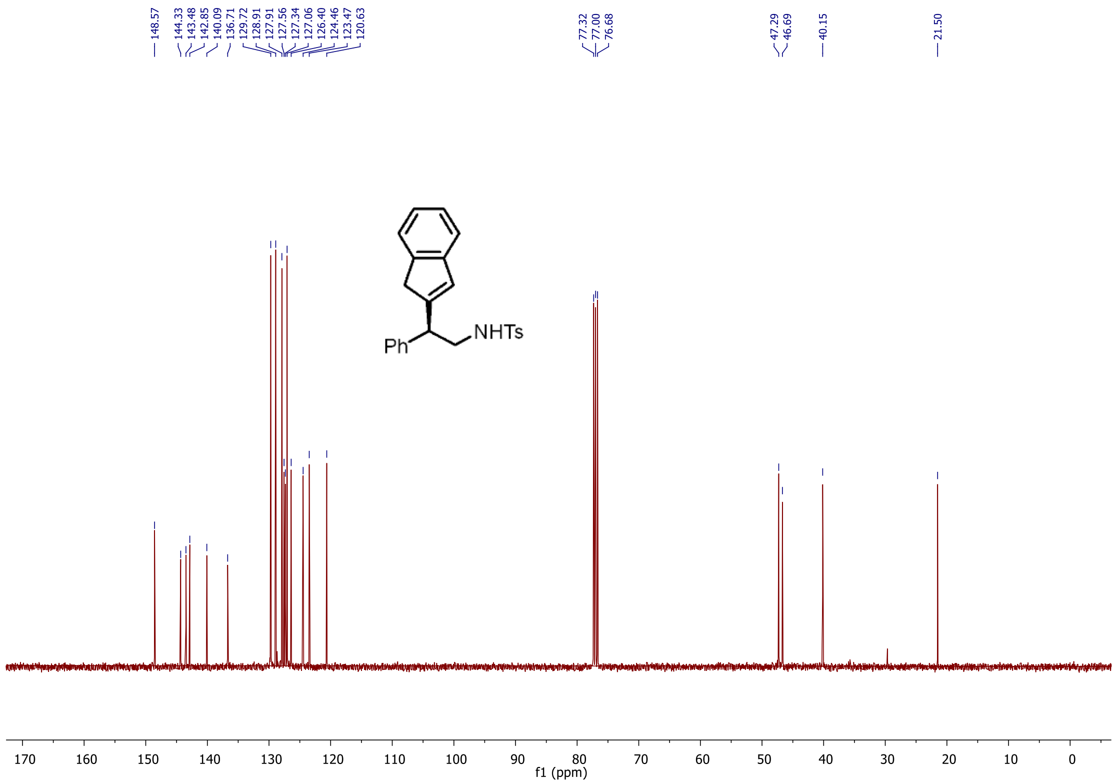

4-Methyl-*N*-((*S*, E)-2-((8*R*, 9*S*, 13*S*, 14*S*)-13-methyl-17-oxo-7, 8, 9, 11, 12, 13, 14, 15, 16, 17-decahydro-6H-cyclopenta[a]phenanthren-3-yl)-4-phenylbut-3-en-1-yl)benzenesulfonamide (35)

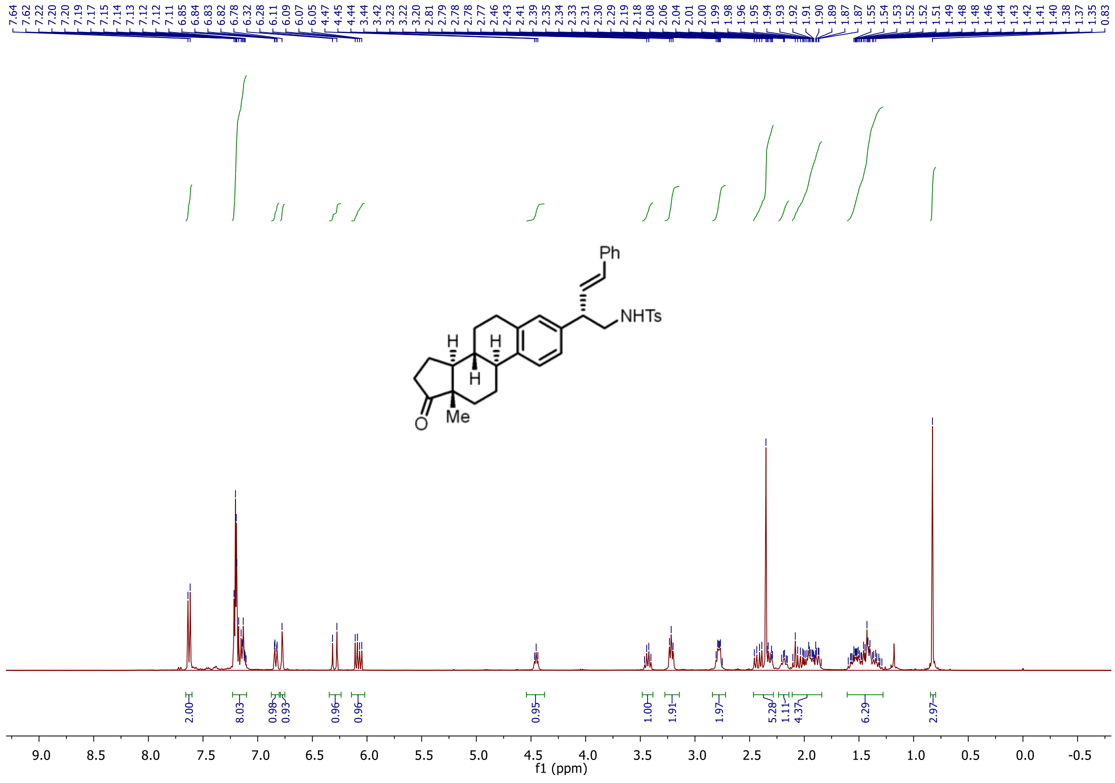

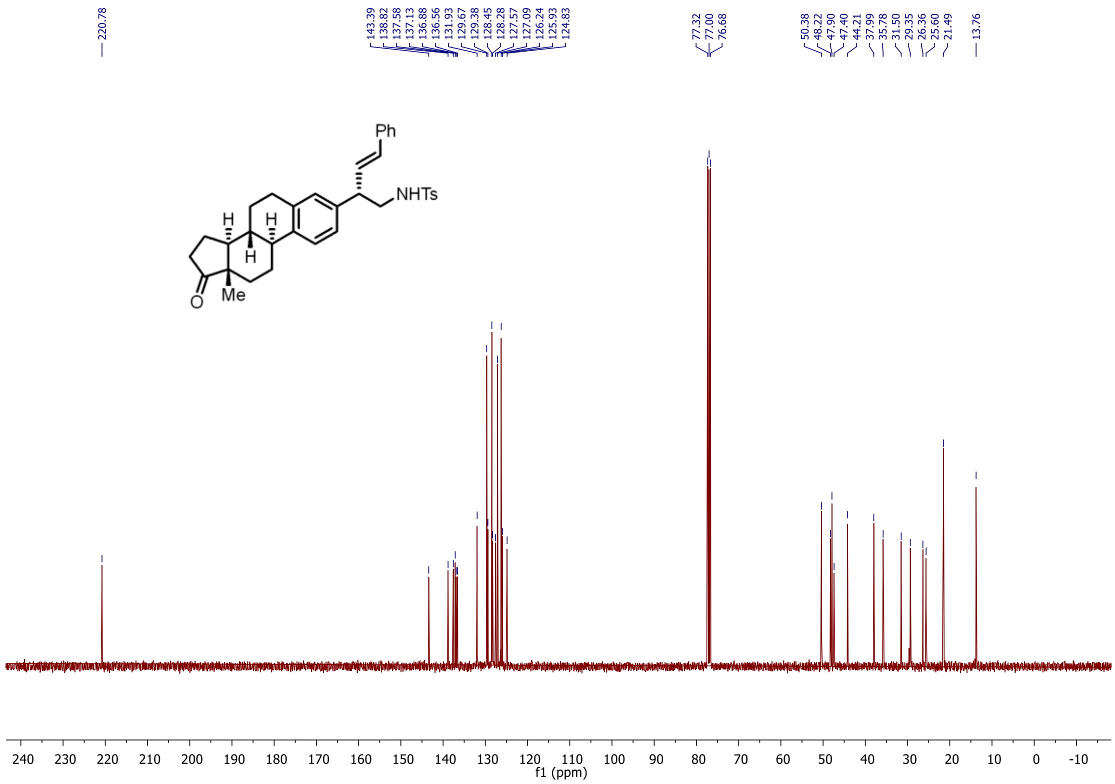

4-Methyl-*N*-((*R*, E)-2-((8*R*, 9*S*, 13*S*, 14*S*)-13-methyl-17-oxo-7, 8, 9, 11, 12, 13, 14, 15, 16, 17-decahydro-6H-cyclopenta[a]phenanthren-3-yl)-4-phenylbut-3-en-1-yl)benzenesulfonamide (*epi*-35)

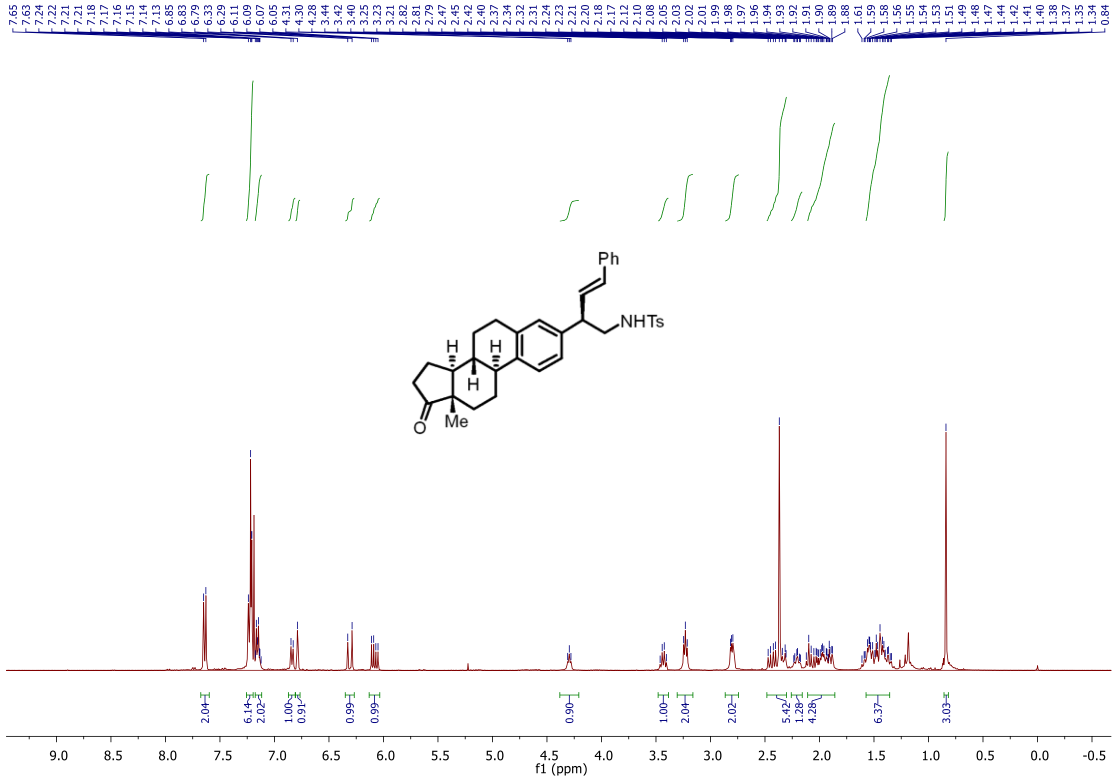

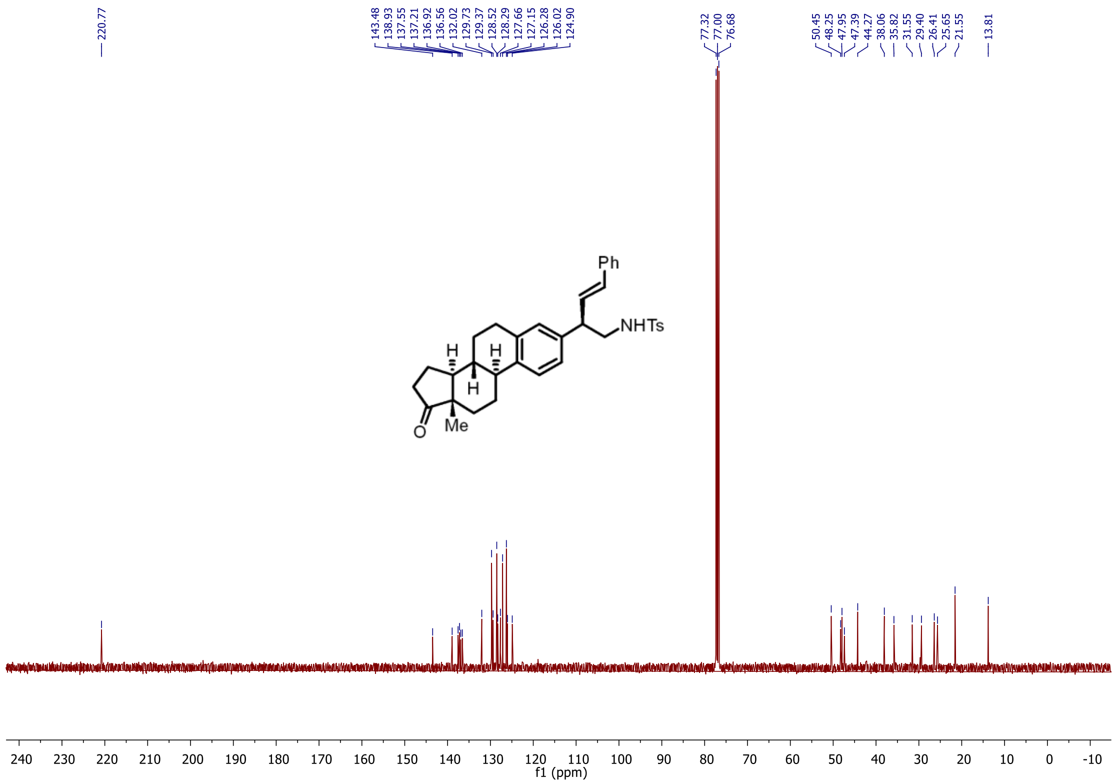

(*R*)-2, 5, 7, 8-Tetramethyl-2-((4R, 8R)-4, 8, 12-trimethyltridecyl)chroman-6-yl 4-((*S*, *E*)-1-((4-methylphenyl)sulfonamido)-4-phenylbut-3-en-2-yl)benzoate (36)

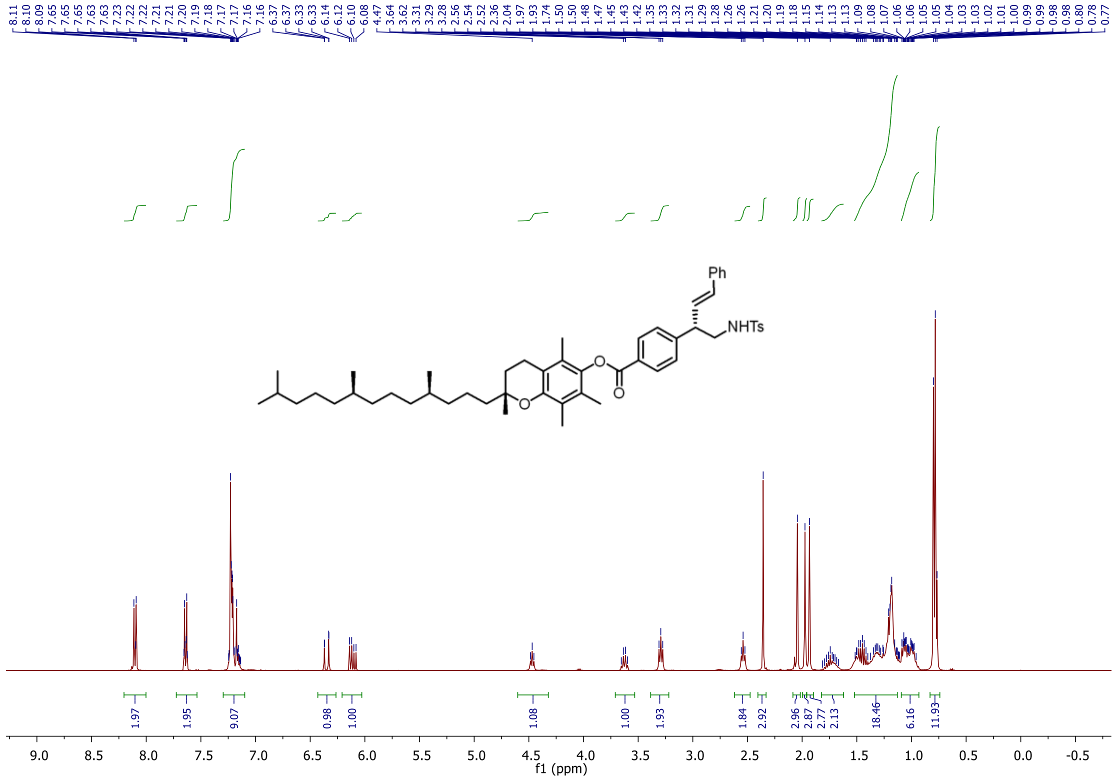

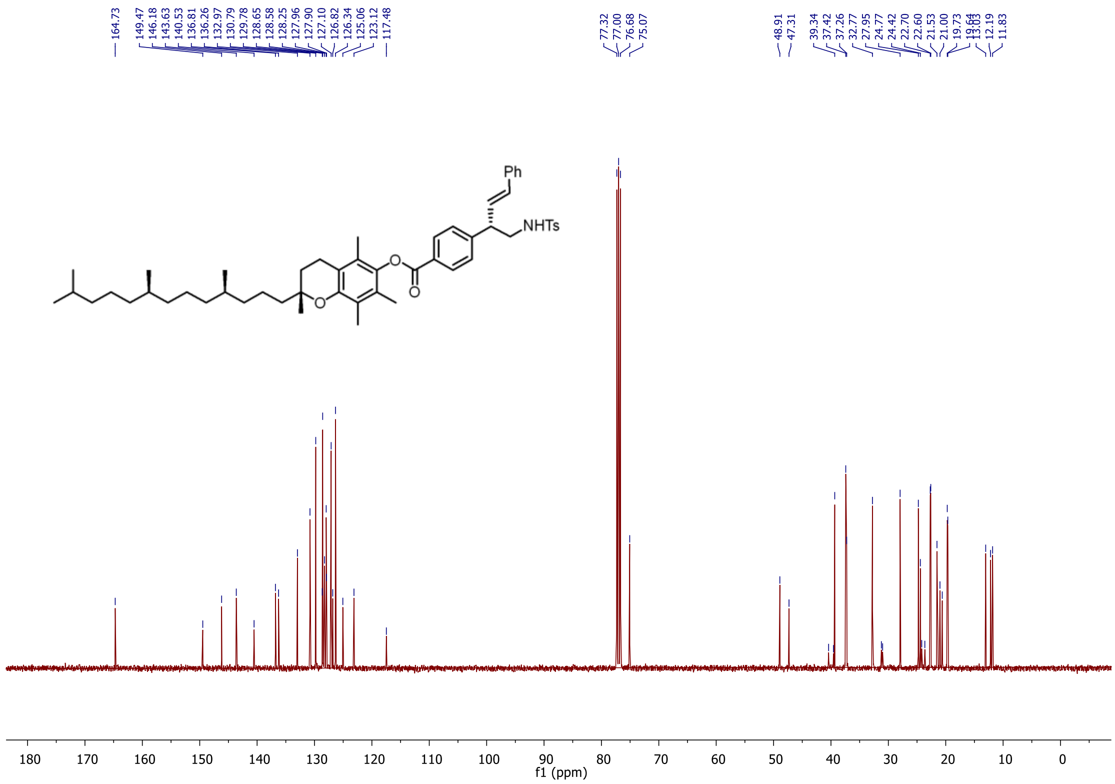

(*R*)-2, 5, 7, 8-Tetramethyl-2-((4R, 8R)-4, 8, 12-trimethyltridecyl)chroman-6-yl 4-((*R*, *E*)-1-((4-methylphenyl)sulfonamido)-4-phenylbut-3-en-2-yl)benzoate (*epi*-36)

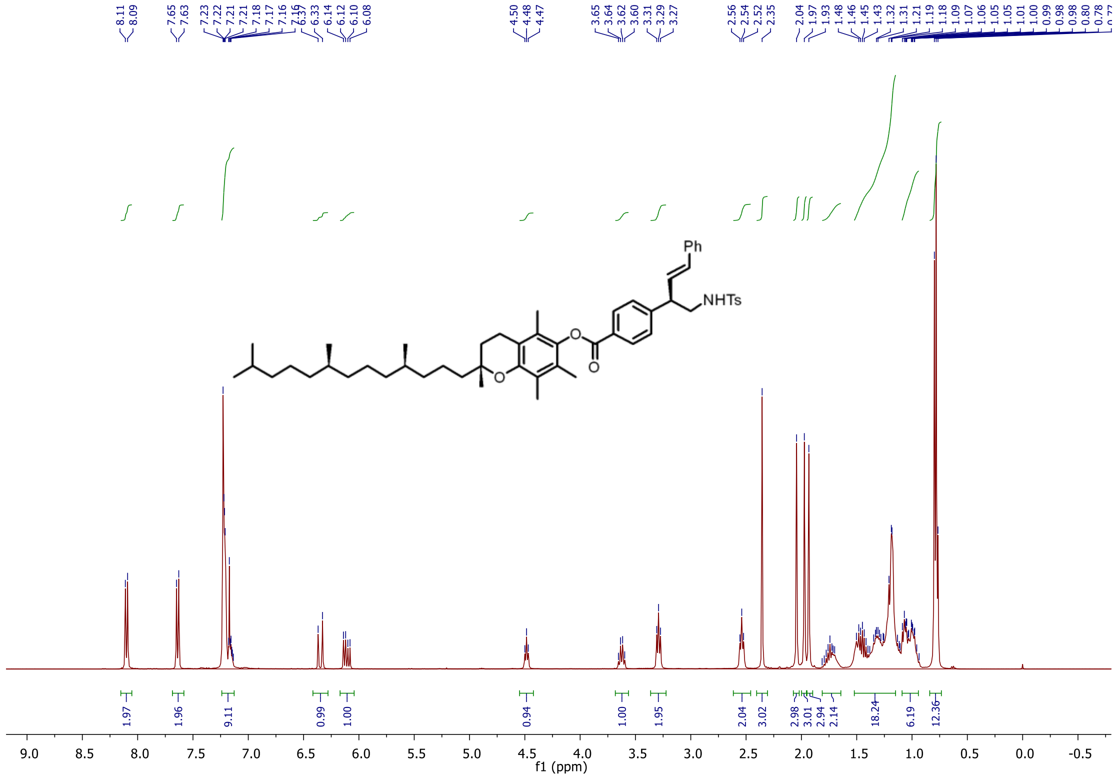

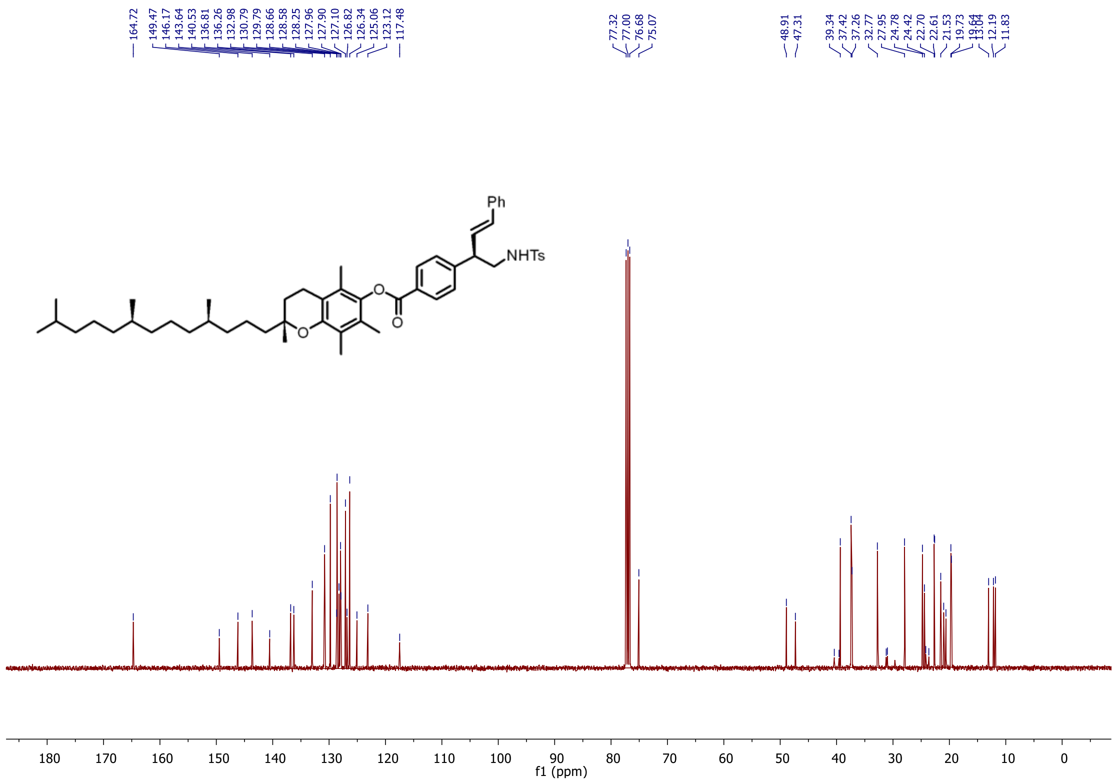

4-((*S*, *E*)-4-((4-Methylphenyl)sulfonamido)-3-phenylbut-1-en-1-yl)phenyl (*S*)-2-(6-methoxynaphthalen-2-yl)propanoate (37)

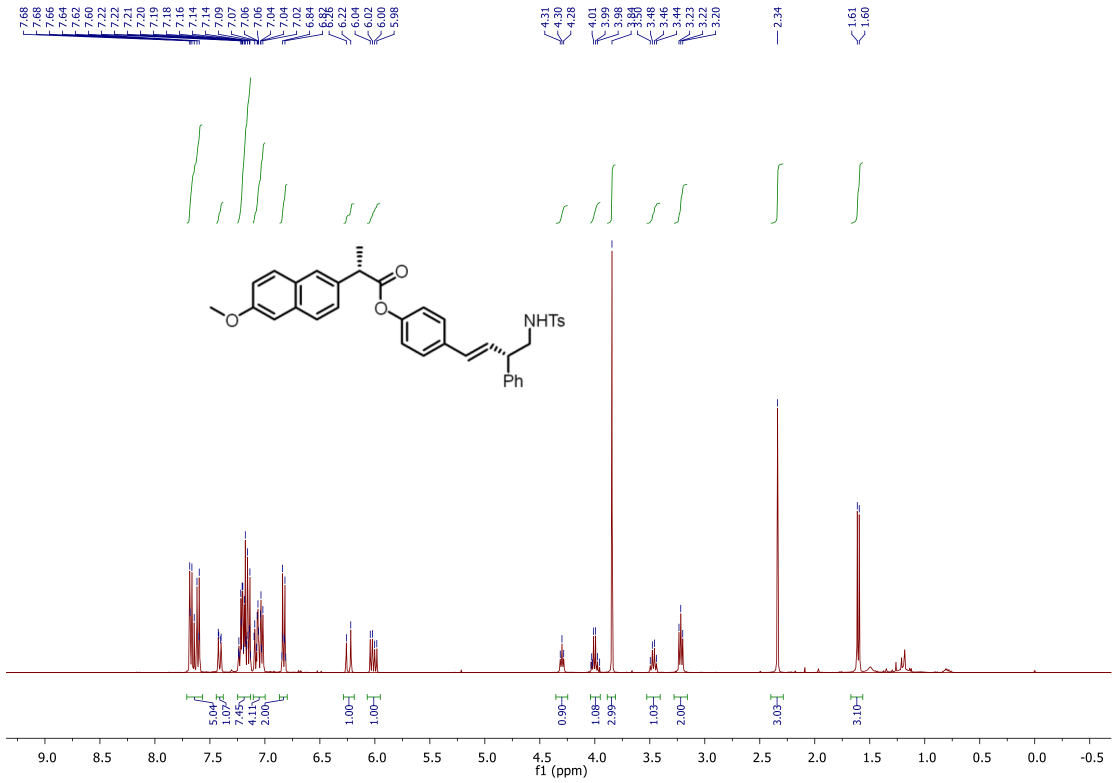

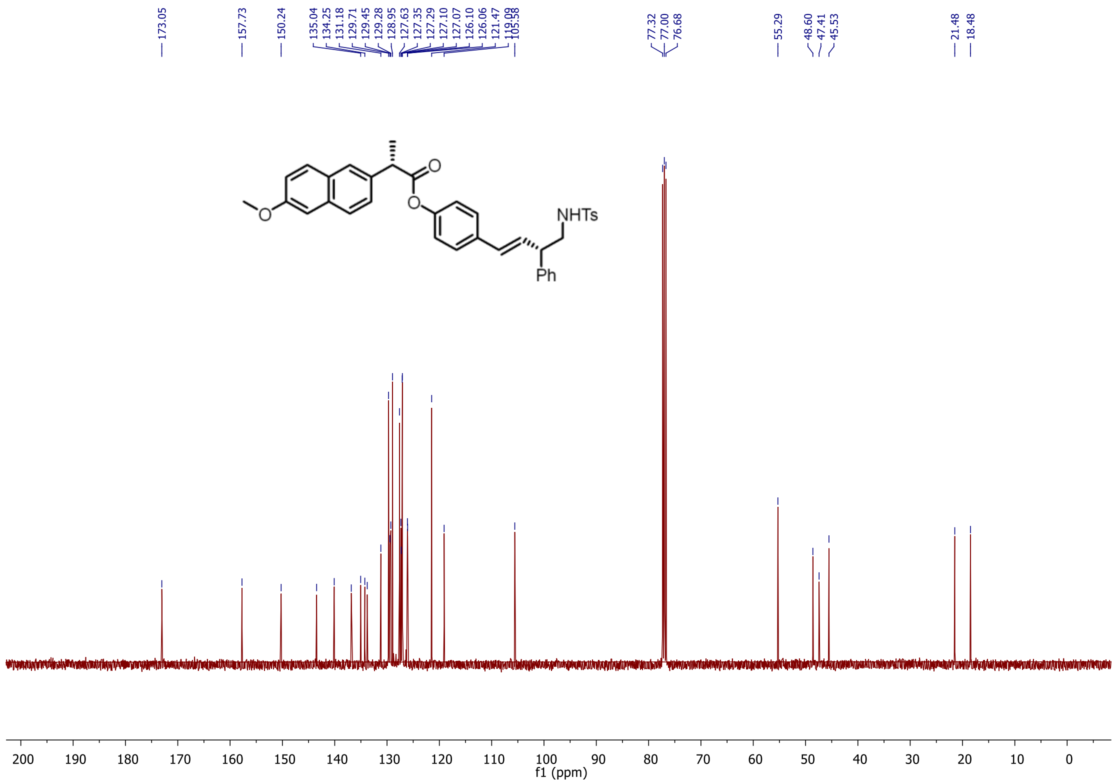

4-((*R*, *E*)-4-((4-Methylphenyl)sulfonamido)-3-phenylbut-1-en-1-yl)phenyl (*S*)-2-(6-methoxynaphthalen-2-yl)propanoate (*epi*-37)

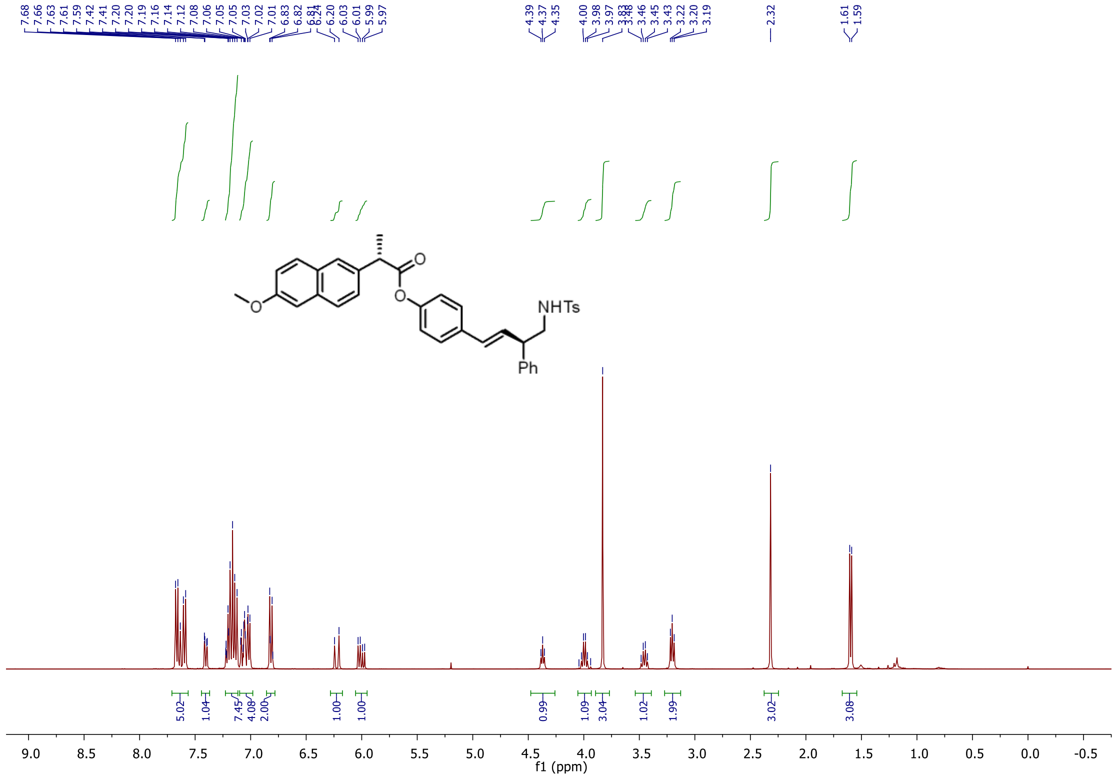

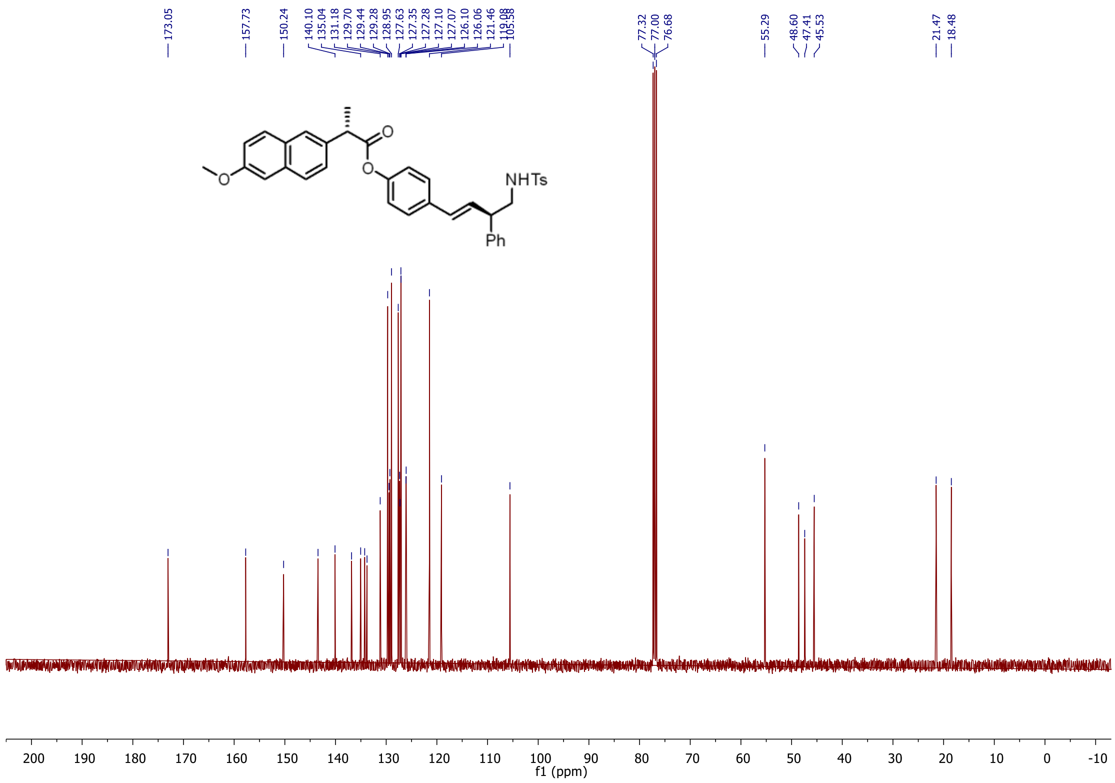

(*S*, *E*)-4-(4-((4-Methylphenyl)sulfonamido)-3-phenylbut-1-en-1-yl)phenyl 2-(4-(4-chlorobenzoyl)phenoxy)-2-methylpropanoate (38)

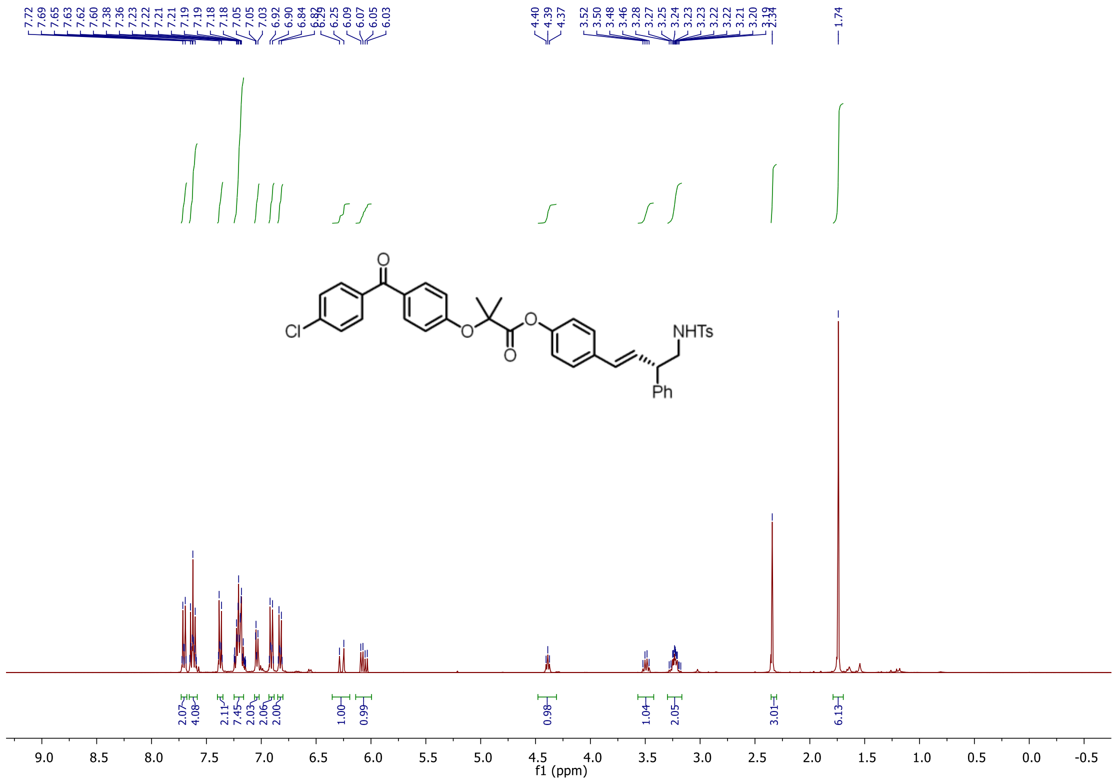

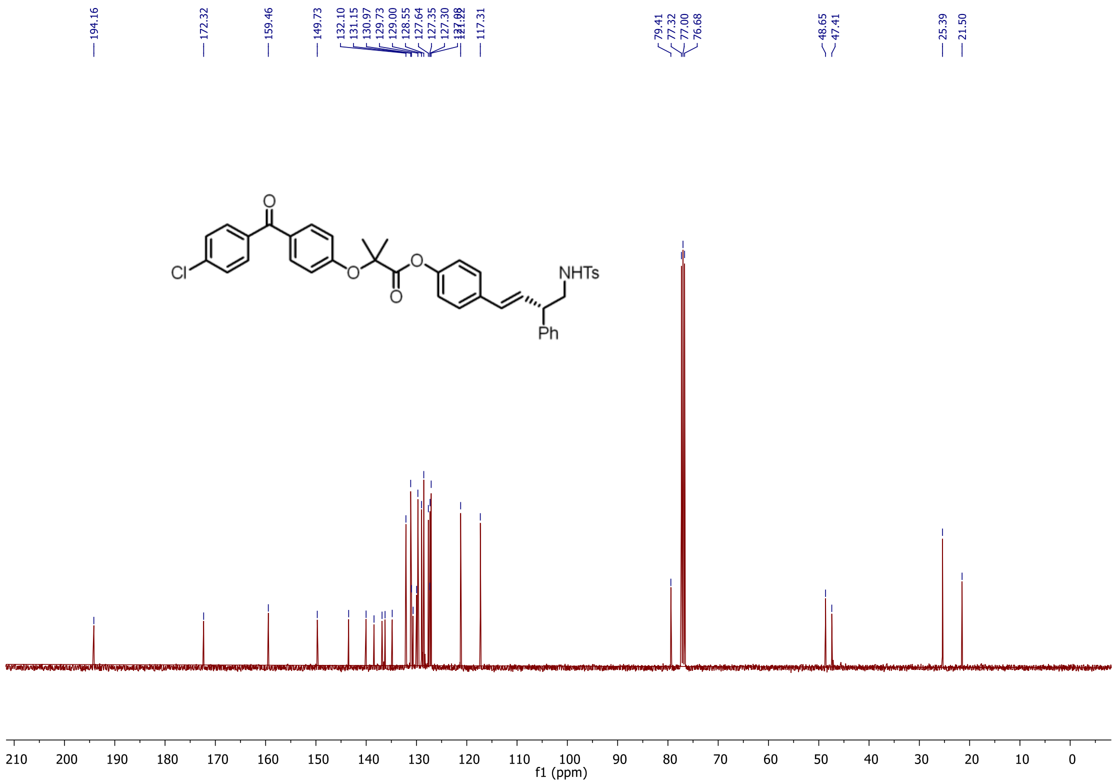

(*S*, *E*)-4-(4-((4-Methylphenyl)sulfonamido)-3-phenylbut-1-en-1-yl)phenyl 5-(2, 5-dimethylphenoxy)-2,2-dimethylpentanoate (39)

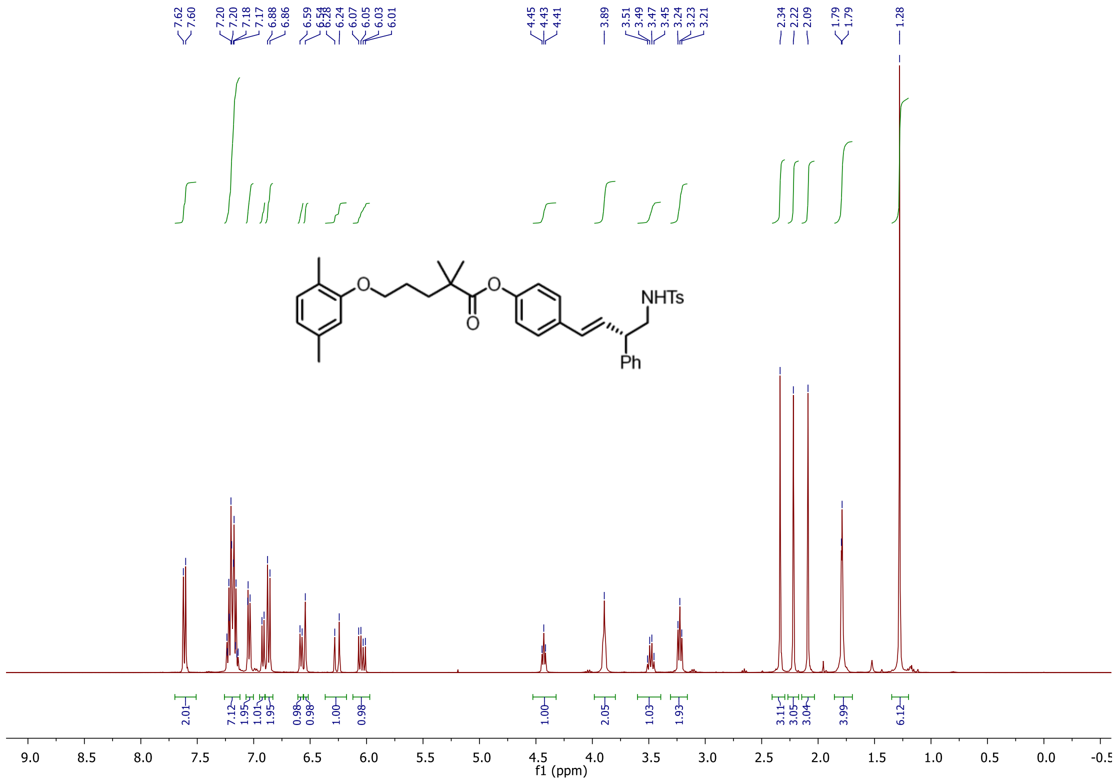

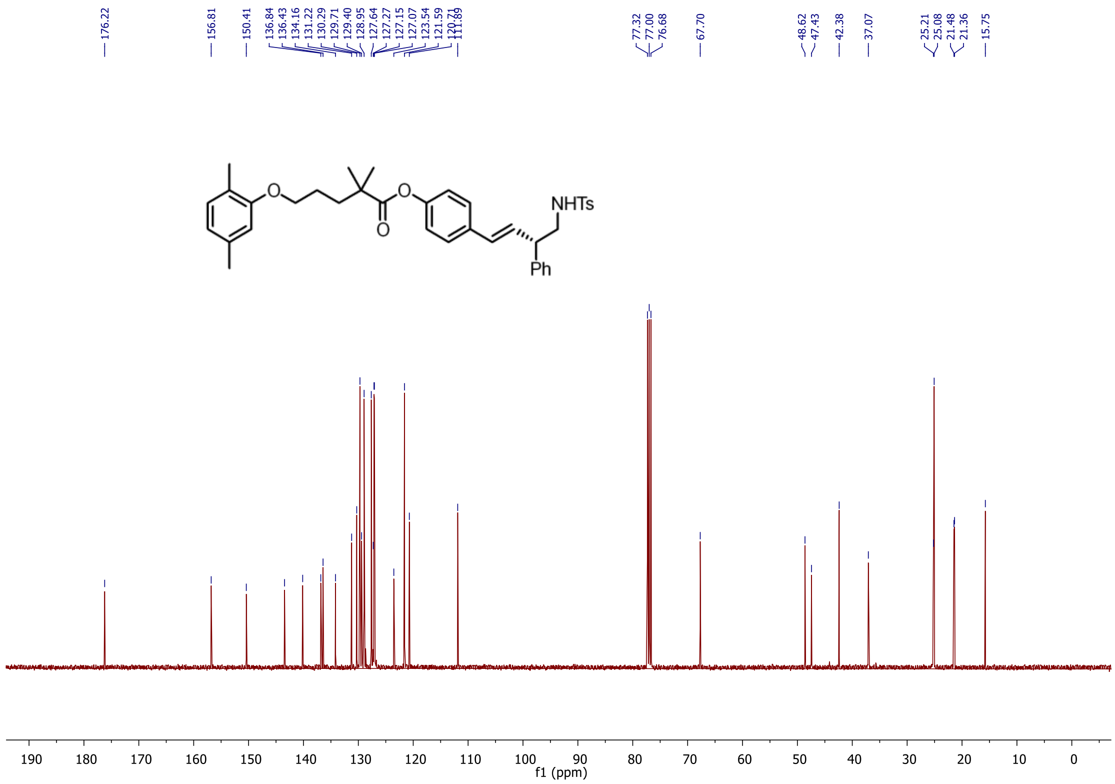

4-((*S*, *E*)-4-((4-Methylphenyl)sulfonamido)-3-phenylbut-1-en-1-yl)phenyl (*tert*-butoxycarbonyl)-D-alaninate (40)

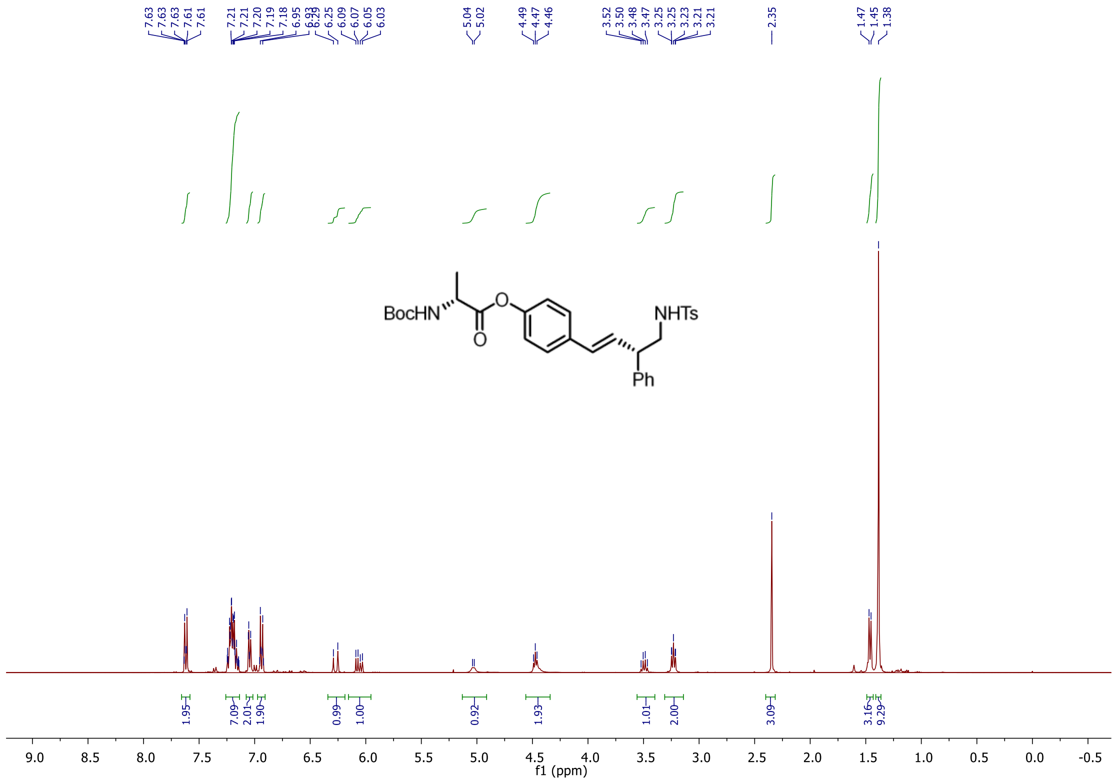

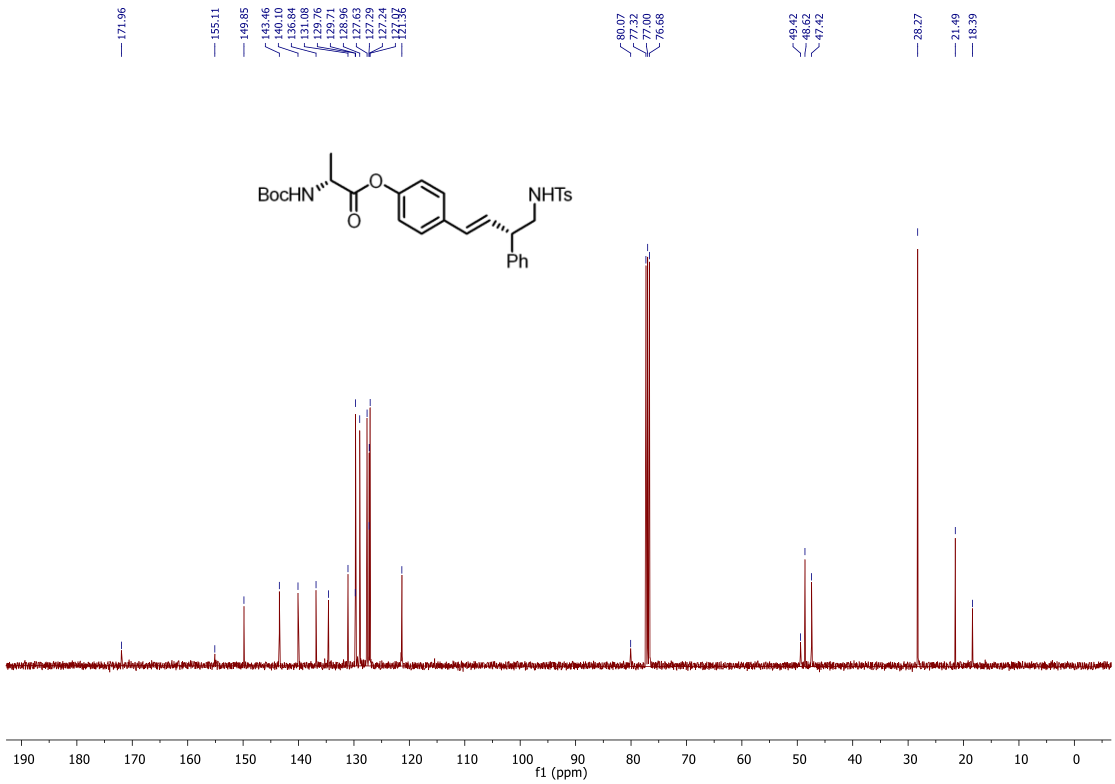

4-((*R*, *E*)-4-((4-Methylphenyl)sulfonamido)-3-phenylbut-1-en-1-yl)phenyl (*tert*-butoxycarbonyl)-D-alaninate (*epi*-40)

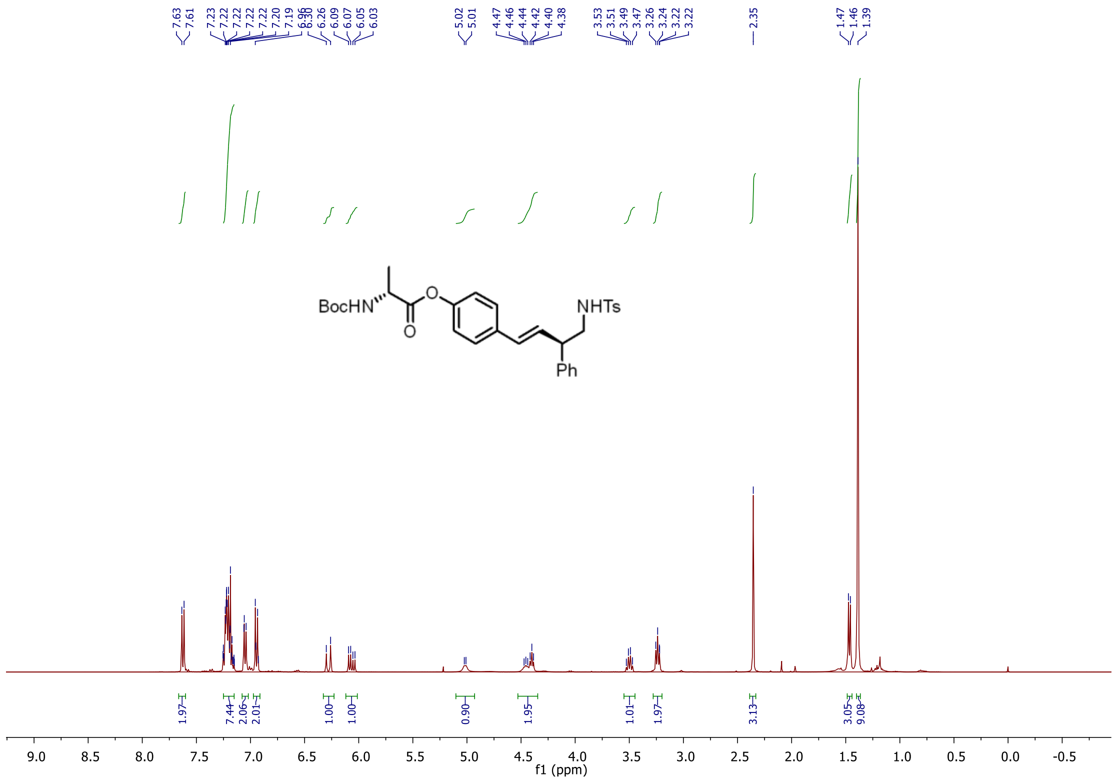

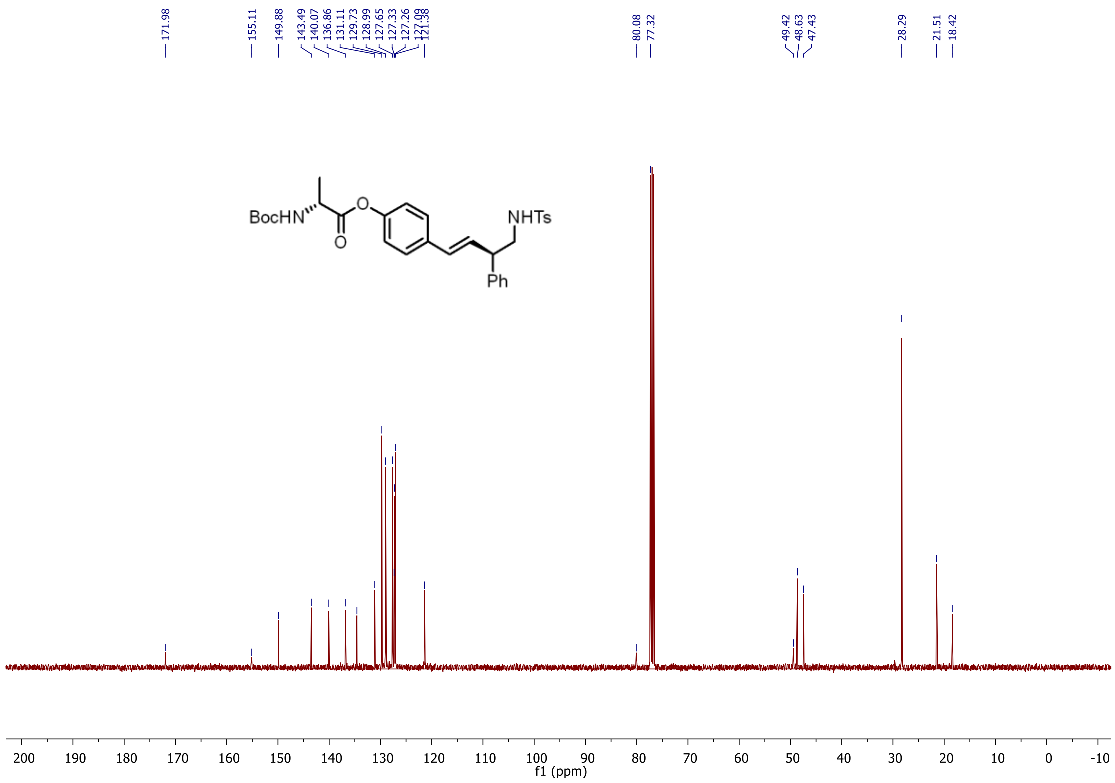

(*S*)-*N*-(2-(4-(*tert*-Butyl)phenyl)-2-phenylethyl)-4-methylbenzenesulfonamide (41)

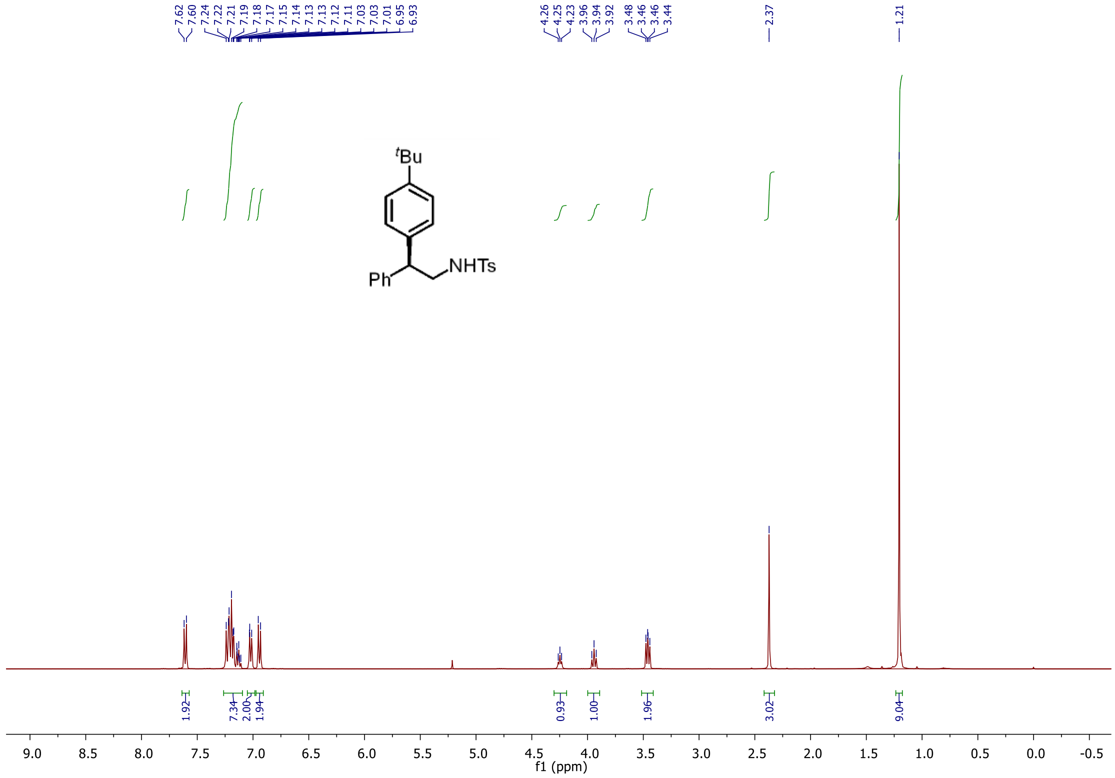

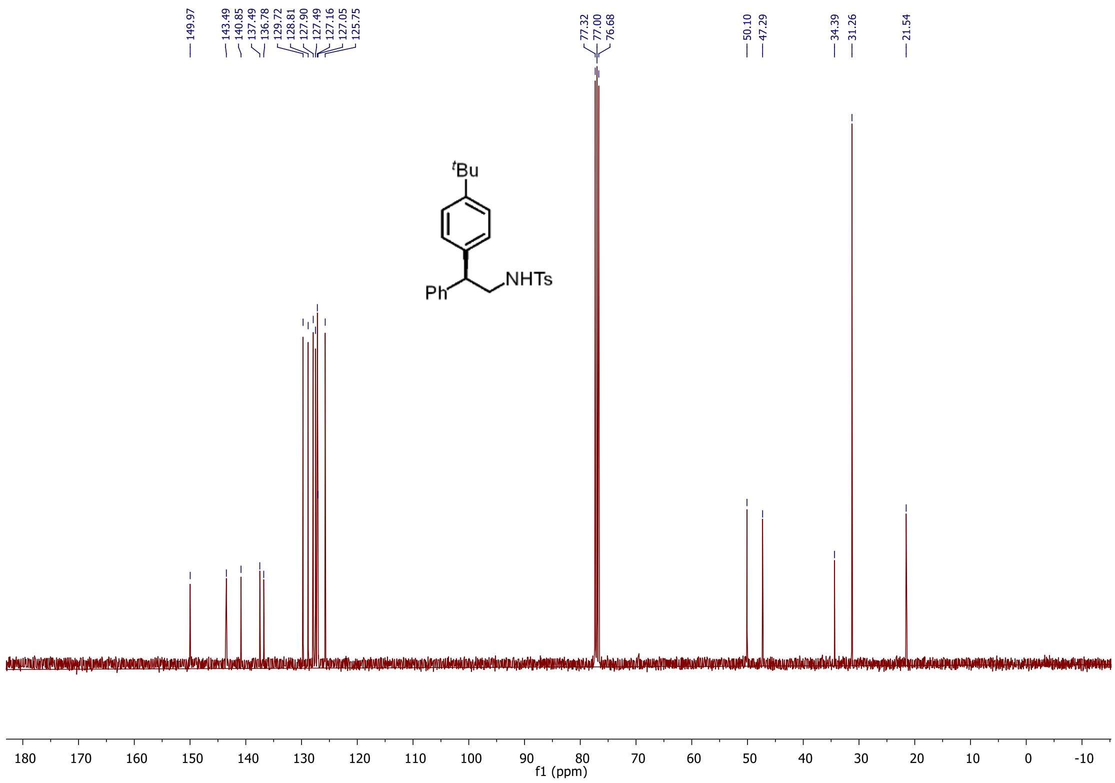

(*S*, *E*)-1-Methoxy-4-(3-phenylbut-1-en-1-yl)benzene (42)

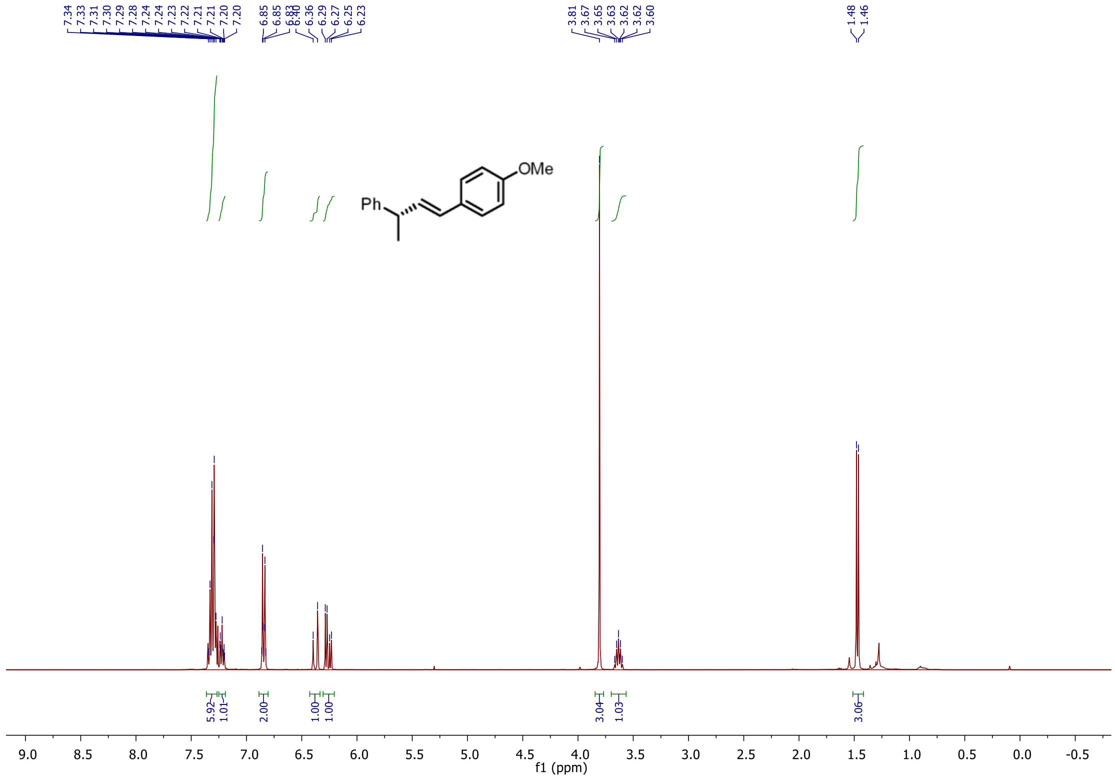

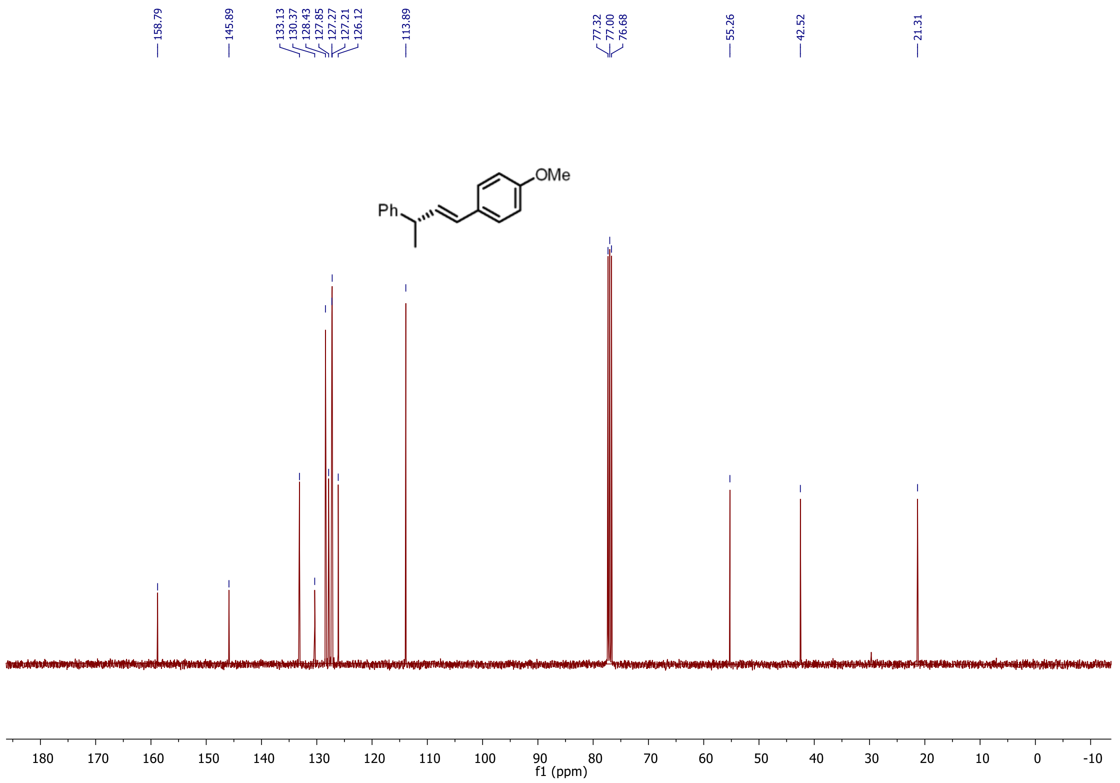

(*S*)-*N*-(2, 4-Diphenylbutyl)-4-methylbenzenesulfonamide (43)

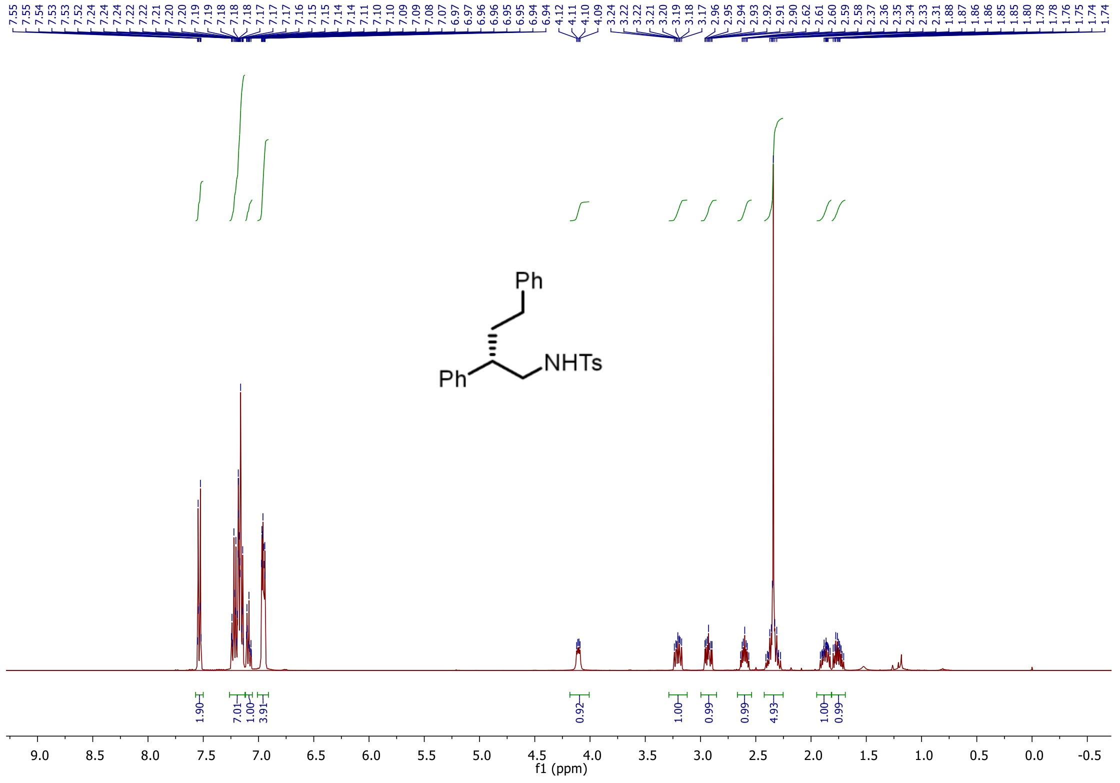

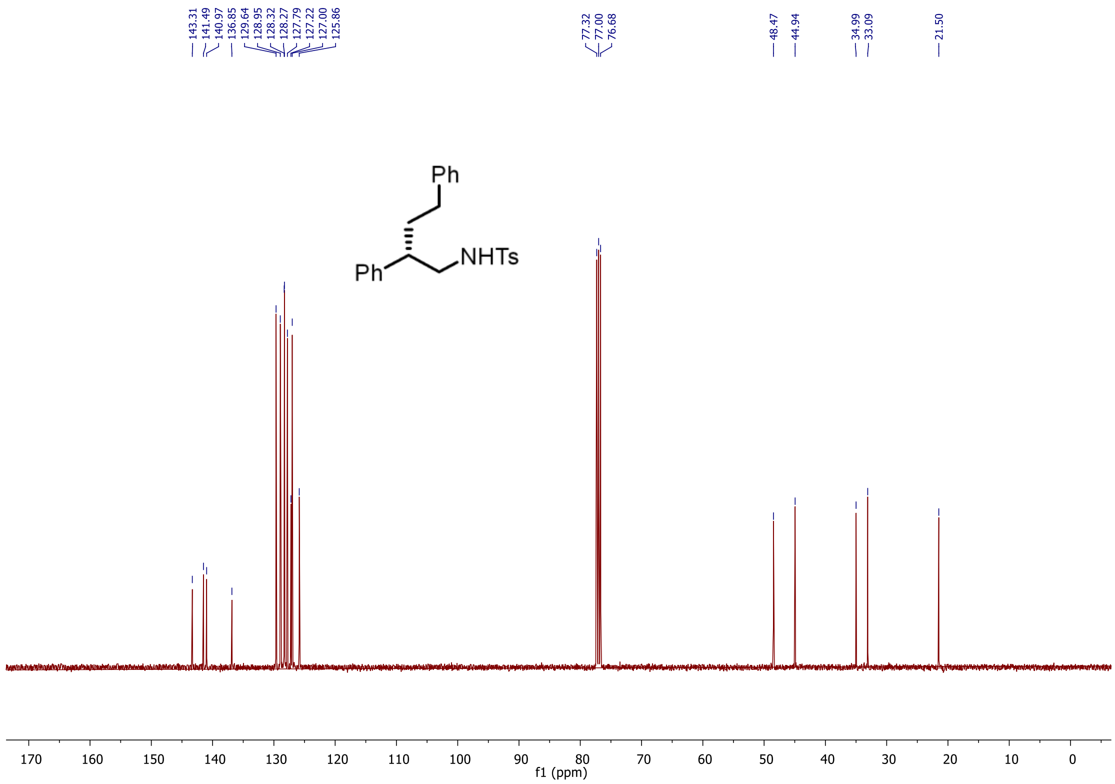

(2*S*, 3*R*, 4*S*)-3-iodo-2, 4-diphenyl-1-tosylpyrrolidine (44)

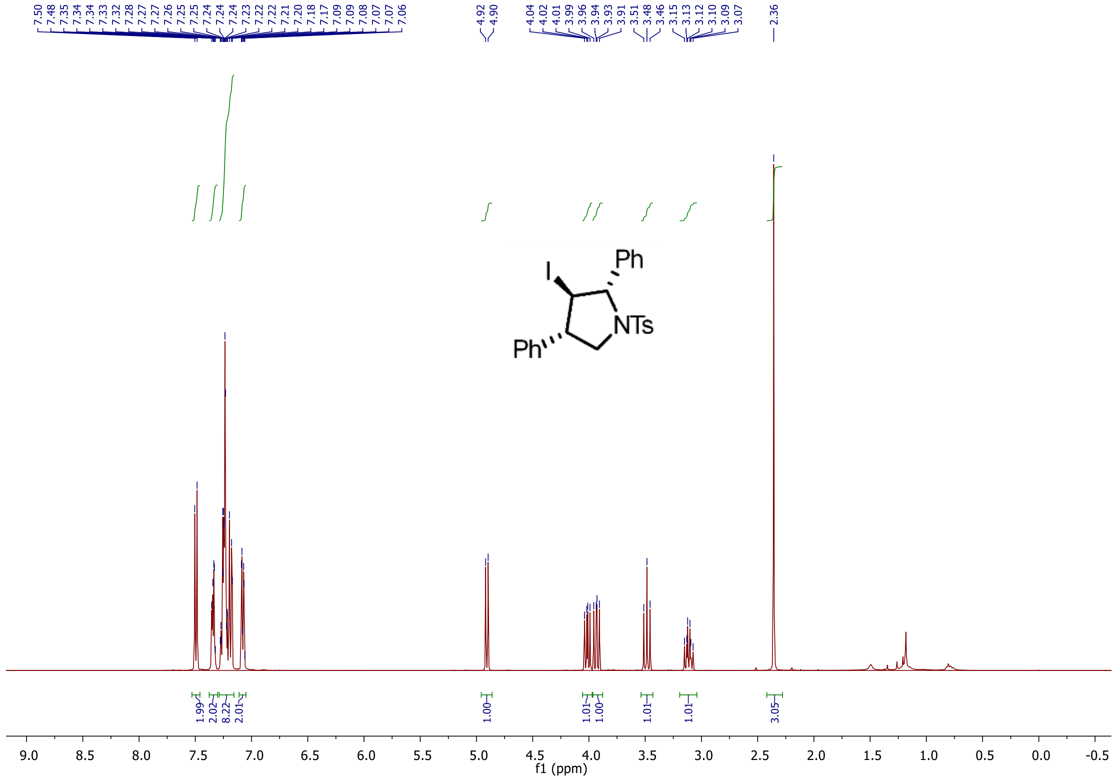

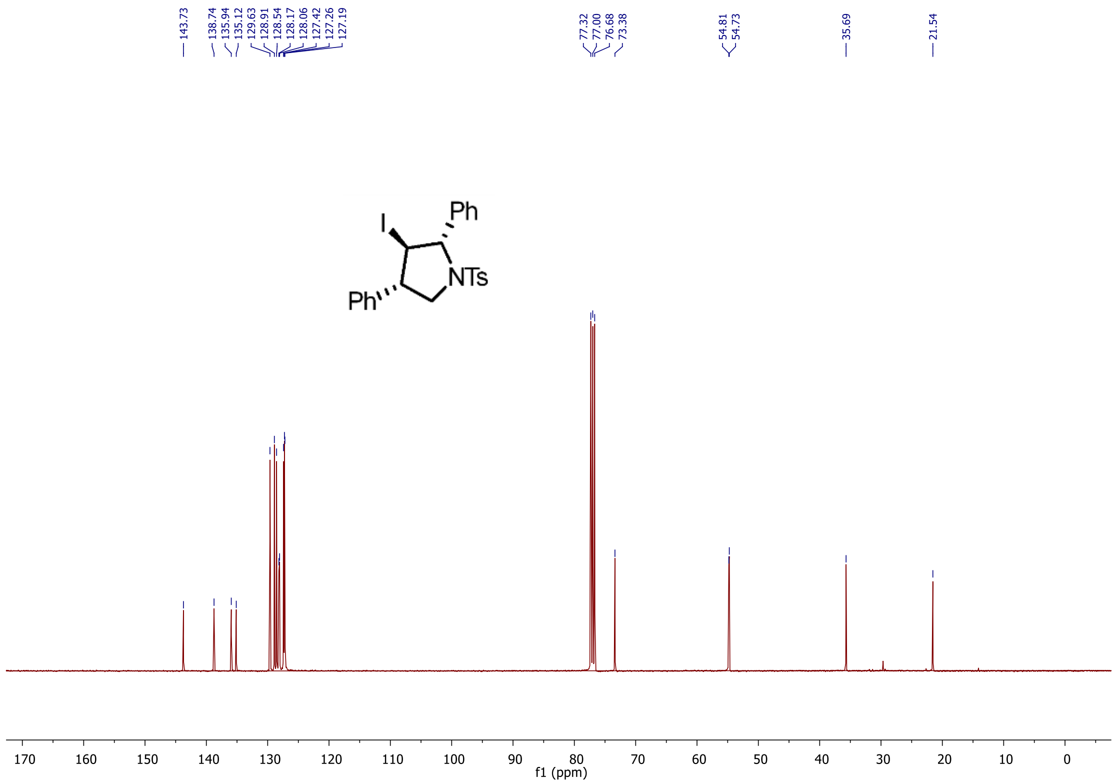

(2*R*, 4*S*)-2, 4-diphenyl-1-tosylpyrrolidine (45)

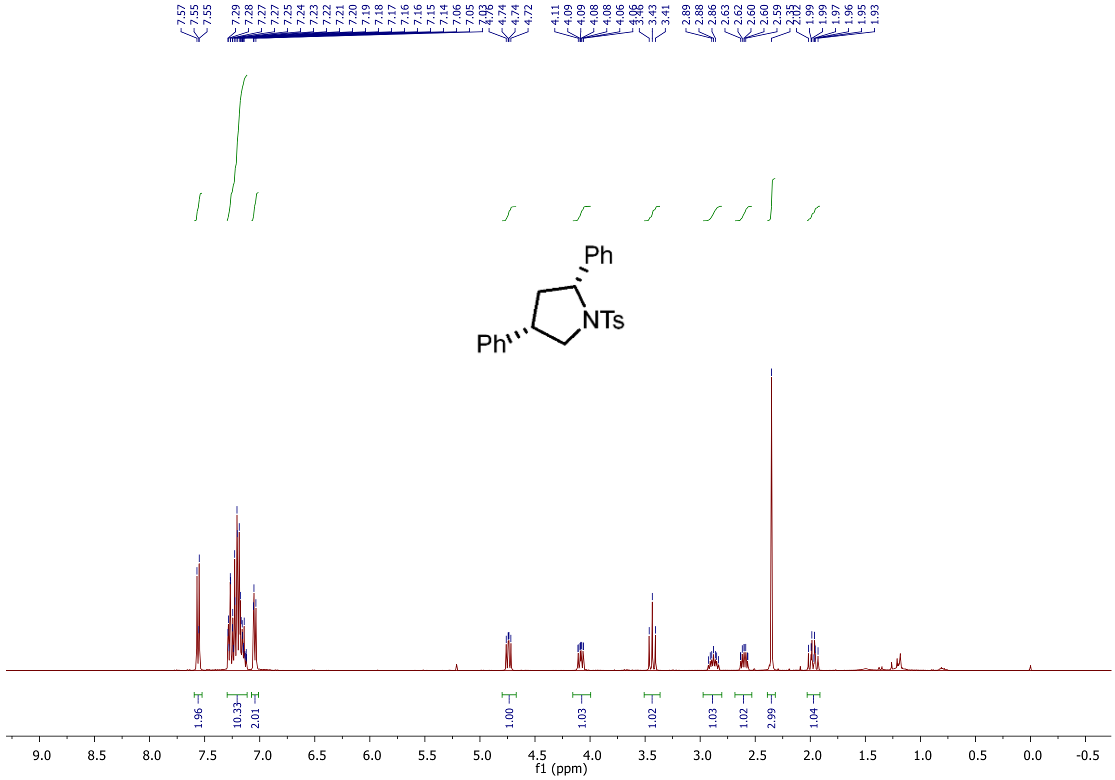

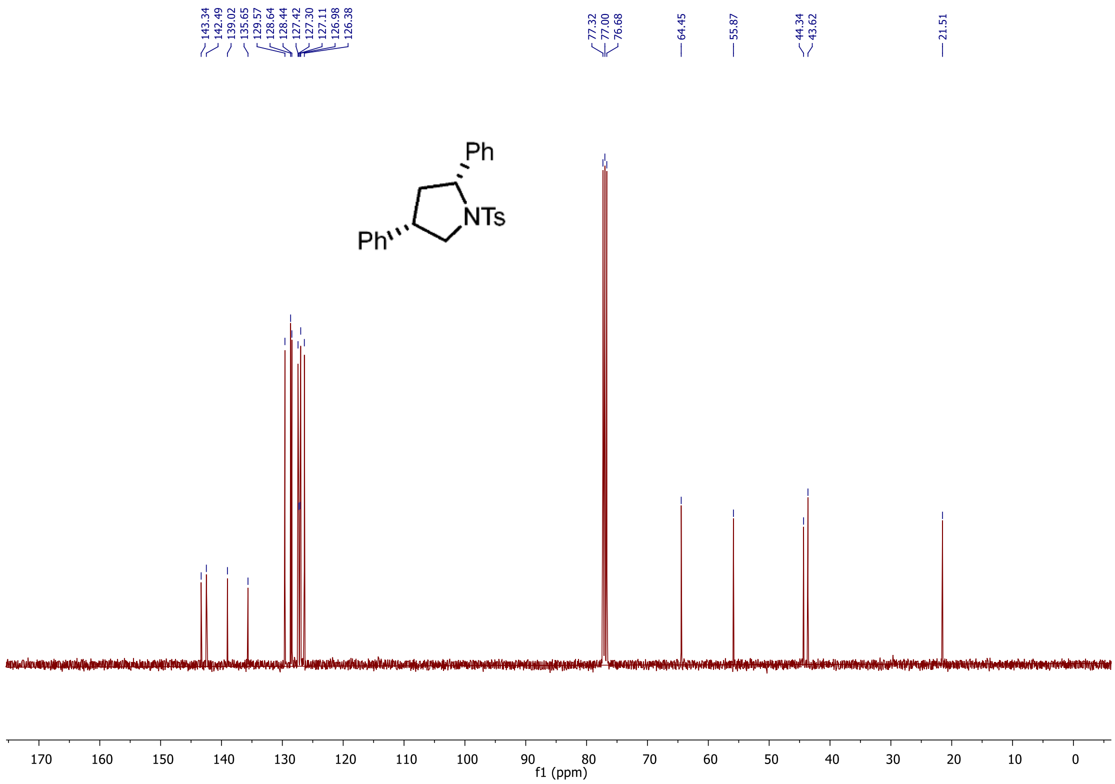

(*S*, *E*)-2, 4-Diphenylbut-3-en-1-amine (46)

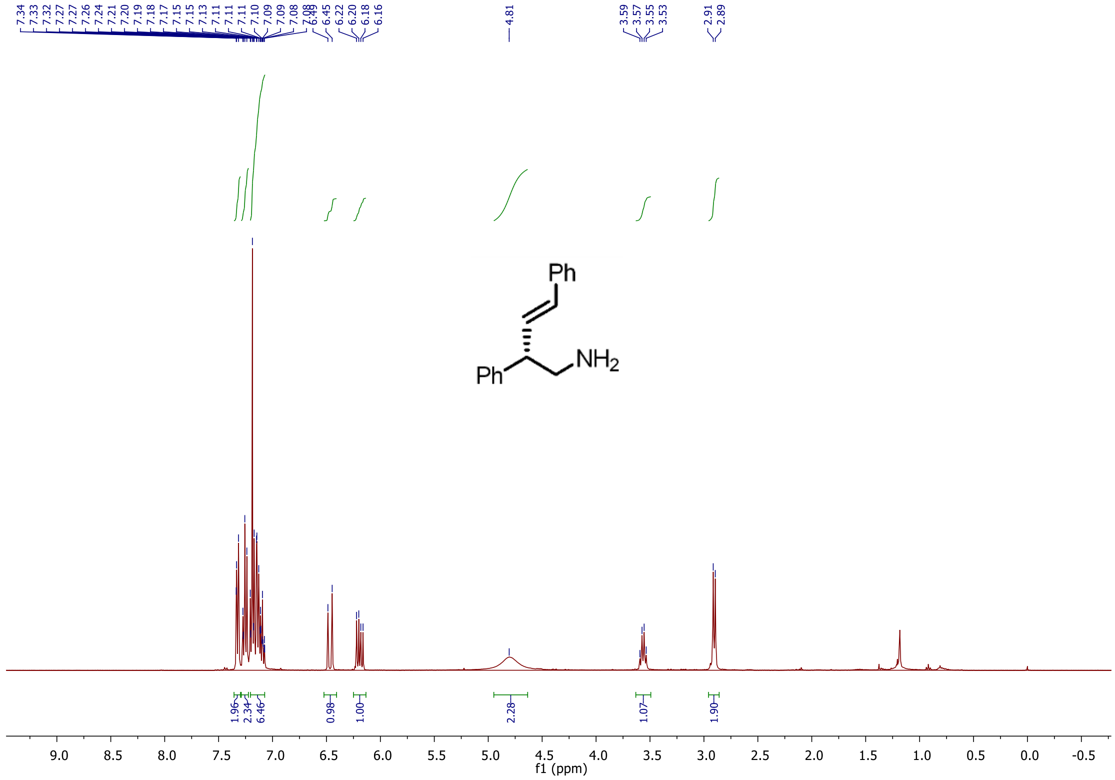

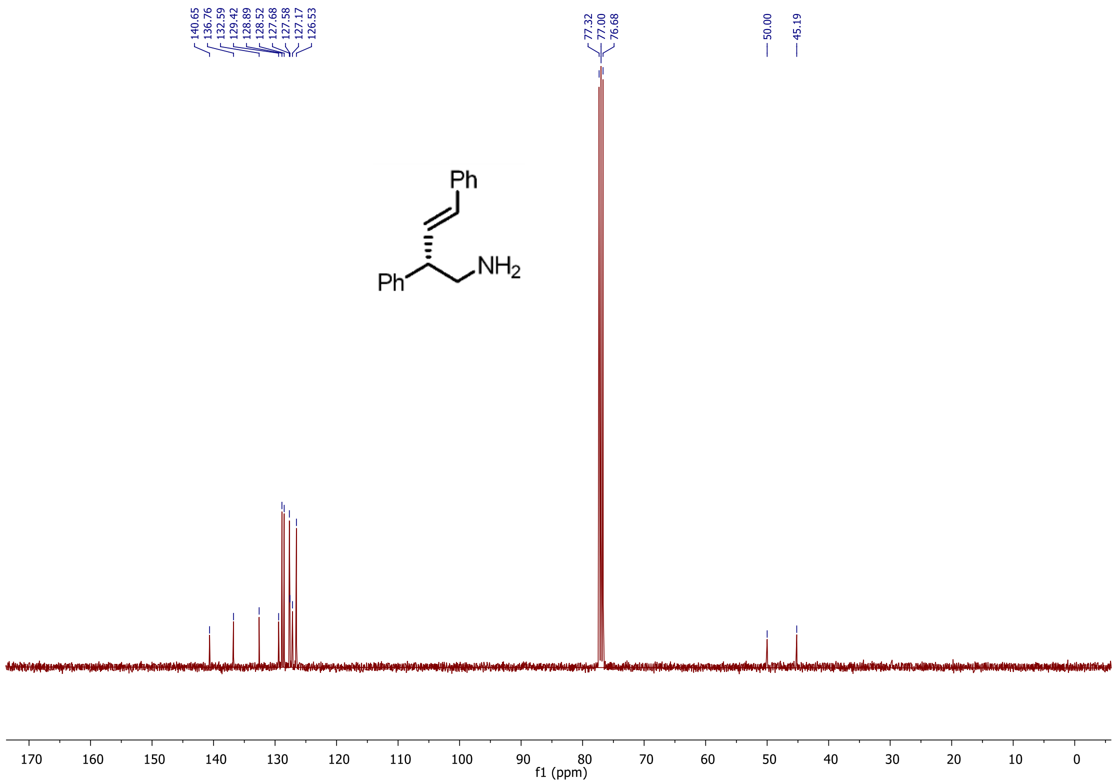
140.65
136.76
132.59
129.42
128.89
128.52
127.68
127.58
127.17
126.53
77.32
77.00
76.68
50.00
45.19
Ph
Ph
NH2
170
160
150
140
130
120
110
100
90
80
70
60
50
40
30
20
10
0
f1 (ppm)
